# Supplementary material for: Project20: maternity care mechanisms that improve access and engagement for women with social risk factors in the UK – a mixed-methods, realist evaluation
Source: BMJ Open. 2023 Feb 7;13(2):e064291. doi: 10.1136/bmjopen-2022-064291 (PMC9906302; doi:10.1136/bmjopen-2022-064291)
Supplement: Supplementary data [file bmjopen-2022-064291supp001.pdf]

Appendix 1: Definitions

Table 1: Definitions of the different models of care received by women at the two service providers evaluated

**Service A**	**Standard Care:** Depending on medical risk factors women receive antenatal and postnatal care as set by NICE guidance [2] in either the community setting or hospital setting. For low risk women care is usually provided by a community midwife in a GP surgery or local children's centre. For women at high medical risk care is provided at the hospital and shared between midwives and obstetricians. Although women may be assigned a 'named' healthcare professional, there is no emphasis on the provision of continuity of care.<br><br>**Group Practice:** Women are seen in either the community or hospital setting depending on their medical risk status. There is an aim to provide antenatal and postnatal continuity of care. Women have a named midwife who aims to see them for most of their antenatal and postnatal appointments. Intrapartum care is covered by the hospitals labour ward or birth centre staff. Women planning a homebirth will be looked after by on-call midwives, but this may be from a team not known to the woman.<br><br>**Specialist model: - Community-based Model [CBM]**<br>A team of 6 midwives provide continuity of care to women located in an area of social deprivation. Not all women under their care will have social risk factors. Each woman is assigned a named midwife who coordinates all care, multi-disciplinary communication, and referrals. The named midwife aims to provide most of the clinical care, with others in the team providing care when s/he is not on duty. The midwives are based in a local community health centre and offer antenatal, intrapartum, and postnatal care in the home, community, or hospital setting.
**Service B**	**Standard Care:** Depending on medical risk factors women receive antenatal and postnatal care as set by NICE guidance [2]. If women do not live within the geographical catchment areas of the group practices (see definition below), care is usually provided at the hospital and shared between midwives and obstetricians. Although women may be assigned a 'named' healthcare professional, there is no emphasis on the provision of continuity of care.<br><br>**Group practice** Women are seen in different settings depending on their medical risk status but there is an aim to provide antenatal and postnatal continuity of care. For most women who live within the hospital's geographical catchment area, care is provided in the community setting, often out of children's centres to prevent women from having to travel to the hospital for appointments. Postnatal care is provided at home and in postnatal clinics in the community. Women have a named midwife who aims to see them for most of their antenatal and postnatal appointments. Intrapartum care is covered by the hospitals labour ward or birth centre staff. Women planning a homebirth are looked after by a team of midwives providing on call care, with the aim for the midwives to have met the woman before.<br><br>**Specialist model: - Hospital-based Model [HBM]**<br>A team of 6 midwives provide continuity of care to women with social risk factors only. Women with one or more significant social risk factors (see Appendix A for inclusion criteria) are referred to the team and assigned a named midwife who coordinates all care, multi-disciplinary communication, and referrals. The named midwife aims to provide most of the clinical care, with others in the team providing care when she/he is not on duty. The midwives are based at the hospital site and offer antenatal, intrapartum, and postnatal care in the home or hospital setting.

Table 2: General definitions

Term	Definition
Deprivation score	A composite measure using routine data from the seven domains of deprivation to identify the most disadvantaged areas in England, UK [2]
Ethnicity	Using the ONS 18+ categories of ethnicity
Medical risk status	As recorded by healthcare professional entering routinely collected maternity record data. High medical risk status leads to obstetric-led care although many women will continue to see both obstetricians and midwives during their pregnancy. Low medical risk status leads to midwife-led care throughout pregnancy, birth and the postnatal period if the pregnancy remains low risk/uncomplicated.
Continuity of Care	The midwife is the lead professional in the planning, organisation and delivery of care given to a woman from initial booking to the postnatal period
Social Risk Factors	As recorded by healthcare professional entering routinely collected maternity record data* and includes:<br>Domestic abuse- an incident or pattern of incidents of controlling, coercive, threatening, degrading and violent behaviour, including sexual violence, in the majority of cases by a partner or ex-partner, but also by a family member or carer.<br>Common mental health- Includes depression, anxiety, post-traumatic stress disorder, and obsessive-compulsive disorder.<br>Severe mental health-Includes psychological problems that are so debilitating that they effect peoples ability to engage in functional and occupational activities. Includes schizophrenia, bipolar disorder and psychosis<br>Non-English speaking<br>Social care involvement- previous or current involvement with social care<br>Drug/alcohol abuse<br>Unsupported/single<br>Financial/housing<br>Learning disability<br>Sexual abuse/trafficked<br>AS/Refugee<br>FGM- Female genital mutilation<br>No recourse to public funds<br>*These risk factors could be ascertained through self-reporting
Demographics	**Level of education (completed)**<br>Secondary school only<br>Completed college<br>Completed university<br>**Occupation Status (NS- SEC)**<br>8 (long term unemployed)

	7 (routine occupations)<br>6-3 (semi-routine)
**Number of antenatal appointments**	Recorded as an episode of care each time a woman has a face-to-face appointment with an obstetrician or midwife during their pregnancy
**Number of antenatal appointments with a known professional**	Recorded as an episode of care each time a woman has a face-to-face appointment with an obstetrician or midwife that they have previously met during their pregnancy. The maximum number of appointments with one healthcare professional is reported for the purpose of this paper.
**Looked after in labour by known midwife**	Looked after at some point during intrapartum care by a healthcare professional seen at least once during the antenatal period.
**Number of missed appointments**	Recorded as an appointment that was scheduled but not carried out or attended.
