# Supplementary material for: Project20: maternity care mechanisms that improve access and engagement for women with social risk factors in the UK – a mixed-methods, realist evaluation
Source: BMJ Open. 2023 Feb 7;13(2):e064291. doi: 10.1136/bmjopen-2022-064291 (PMC9906302; doi:10.1136/bmjopen-2022-064291)
Supplement: Supplementary data [file bmjopen-2022-064291supp002.pdf]

Supplementary file 2: Programme theory refinement and supplementary data (Adjusted and unadjusted data analysis tables and qualitative data)

This file will detail the process of theory refinement. Initial programme theories and CMO configurations constructed by Rayment-Jones et al [1,2] to gain further insight into how and why specialist models of care work, or do not work, for women with low socioeconomic status and social risk factors. Each initial CMO configuration was tested through a three-step process- see Figure 1below for an explanatory diagram of the process:

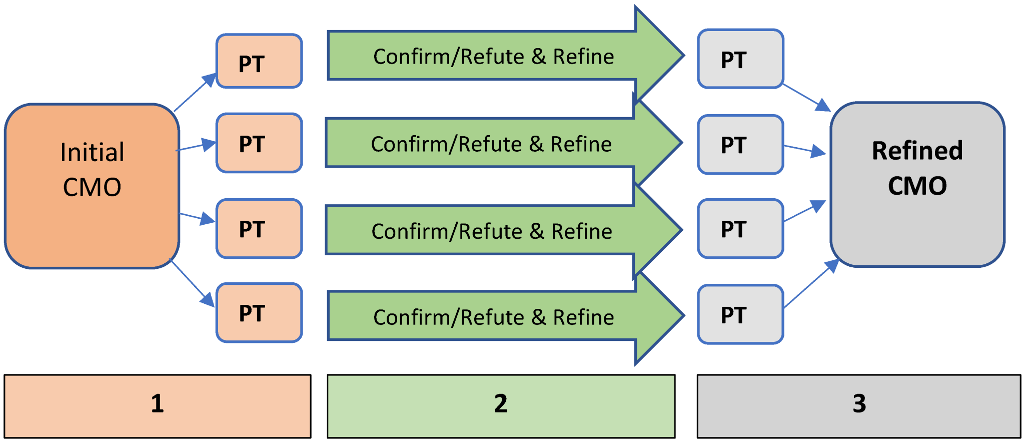

Figure 1: Programme theory refinement CMO configuration development process

This process is described below with the numbers correlating to the order of the diagram:

1) The initial CMO configuration is presented and broken down into smaller testable programme theories. Rival, or conflicting theories, and new theories are presented alongside these programme theories. These rival theories were identified throughout the project including the realist synthesis, focus groups with service providers, patient and public involvement group liaison, supervisory and advisory group discussions.
2) The programme theories are individually tested by drawing on the relevant quantitative data presented in Chapter 7, and qualitative longitudinal interview data to confirm or refute the programme theory before refining them to better reflect the findings.
3) The refined programme theories relating to the initial CMO configuration are compiled in a new, refined CMO configuration that represents the findings of the research.

The above three step process gives way to middle-range theories, indicating how the specialist model of care activates mechanisms amongst whom and in what conditions to bring about different outcomes. The refined CMO configurations summarised in Table 13 of the main manuscript will inform the development of a theoretically informed model of maternity care for women with social risk factors.

Analysis 1: Model of care accessed by deprivation score and risk factors

Initial Programme Theory: If women living in areas of deprivation are prioritised to receive continuity of care through community-based models then services are likely to identify women with social risk factors who have not previously disclosed these issues with professionals, and care is less likely to be disrupted during pregnancy when a disclosure is made. This may also avoid women feeling discriminated against due to specific social risk factors that they may feel do not place them at higher risk or may place them at a higher risk of increased surveillance.

Rival Theory: Placing specialist models of care that include increased levels of continuity in deprived communities does not protect those services from becoming used by more affluent women who are not at such high risk of poor outcomes and experiences and are able to coordinate the system. This would result in those women with low SES and social risk factors having to seek care elsewhere, perhaps outside of their local communities.

Testing using quantitative data:
Firstly, the quantitative data was used to test the hypothesis that women with low socioeconomic status and social risk factors are most likely to receive specialist models of care. The data presented in Table 1 shows that women receiving specialist models of care were more likely to be in the more deprived deciles even after adjusting for women's characteristics, the service provider attended and the place of antenatal care. These findings suggest that the aims of the specialist models- to reach the most deprived women, are being met. Further analysis shows that the specialist models of care based in areas of deprivation were indeed caring for more women with low SES and social risk factors, therefore refuting the rival theory. However, Table 2 also highlighted a significant relationship between the place of antenatal care and the model of care received, with women in the highest deprivation deciles attending hospital based antenatal care being less likely to be cared for by the specialist model. This supports the initial programme theory that community-based models are more likely to identify women with low SES who are more likely to be experiencing social risk factors.

Table 1: Maternity care received by deprivation score

```
Multinomial logistic regression                 Number of obs     =        765
                                                LR chi2(57)       =     153.19
                                                Prob > chi2       =     0.0000
Log likelihood = -948.68392                     Pseudo R2         =     0.0747
```

imd_3_score	RRR	Std. Err.	z	P>|z|	[95% Conf.	Interval]
**most_deprived**						
mod_care_4						
standard	.291514	.1930548	-1.86	0.063	.0796072	1.067497
partial CoC	.1270207	.0863271	-3.04	0.002	.0335253	.481257
ethnicity						
BA	8.688427	6.039025	3.11	0.002	2.224848	33.92985
BC	4.741514	3.985545	1.85	0.064	.91291	24.6267
BO	3.775191	4.386607	1.14	0.253	.3871507	36.81272
M	1.711281	1.391649	0.66	0.509	.3476215	8.424344
U	2.078345	.9214765	1.65	0.099	.8716061	4.955816
WB	.6403515	.2711355	-1.05	0.292	.279258	1.468356
WO	1.002714	.3973854	0.01	0.995	.4611452	2.180302
WP	2.391133	13830.83	0.00	1.000	0	.
age_cat						
20-24	1.37e-06	.0012548	-0.01	0.988	0	.
25-29	1.19e-06	.0010827	-0.01	0.988	0	.
30-34	4.47e-07	.0004081	-0.02	0.987	0	.
greater than 34	2.79e-07	.0002551	-0.02	0.987	0	.
0.parity	.6508242	.1653202	-1.69	0.091	.395589	1.070738
1.any_risk	1.684199	.5775913	1.52	0.129	.8599561	3.298455
2.high_risk_num	2.372965	.9909666	2.07	0.039	1.046704	5.379712
place_hosp_comm						
hospital	.4068348	.1188187	-3.08	0.002	.2295193	.7211356
imp_gstt						
imp	2.764661	.8701099	3.23	0.001	1.491925	5.123148
_cons	1.15e+07	1.05e+10	0.02	0.986	0	.
**3rd_and_4th_deciles**						
mod_care_4						
standard	.2897059	.1877638	-1.91	0.056	.081335	1.031898
partial CoC	.1905813	.1257227	-2.51	0.012	.052307	.6943858
ethnicity						
BA	5.239074	3.616894	2.40	0.016	1.353978	20.27205
BC	2.414152	2.031332	1.05	0.295	.4640247	12.55996
BO	7.223335	7.936432	1.80	0.072	.8385027	62.22588
M	1.297728	1.029289	0.33	0.742	.2741926	6.142027
U	2.248795	.9372376	1.94	0.052	.9935552	5.089884
WB	.689703	.2667646	-0.96	0.337	.3231713	1.471945
WO	1.130883	.4187918	0.33	0.740	.5472657	2.336883
WP	3.09e+07	1.41e+11	0.00	0.997	0	.
age_cat						
20-24	1.58e-06	.001445	-0.01	0.988	0	.
25-29	9.24e-07	.0008435	-0.02	0.988	0	.
30-34	5.30e-07	.0004843	-0.02	0.987	0	.
greater than 34	2.87e-07	.0002617	-0.02	0.987	0	.
0.parity	.66367	.1575855	-1.73	0.084	.4167141	1.056979
1.any_risk	1.480933	.4932381	1.18	0.238	.7709656	2.844695
2.high_risk_num	2.558484	.9732096	2.47	0.014	1.213942	5.392215
place_hosp_comm						
hospital	.4245959	.1120848	-3.25	0.001	.2530904	.7123212
imp_gstt						
imp	1.547321	.4430837	1.52	0.127	.8827429	2.71223
_cons	1.86e+07	1.70e+10	0.02	0.985	0	.
**5th_and_6th_deciles**						
mod_care_4						
standard	.2513724	.1692147	-2.05	0.040	.0671924	.9404055
partial CoC	.1490418	.1026833	-2.76	0.006	.0386249	.575107
ethnicity						
BA	5.558974	4.138049	2.30	0.021	1.292314	23.9123
BC	4.761389	4.20312	1.77	0.077	.843985	26.86164
BO	2.572342	3.305336	0.74	0.462	.2072886	31.92139
M	.9059086	.9060892	-0.10	0.921	.1275596	6.433623
U	2.623069	1.22996	2.06	0.040	1.046359	6.575653
WB	1.151676	.5021643	0.32	0.746	.4899841	2.706942
WO	1.536157	.6463339	1.02	0.308	.6734338	3.504099
WP	1.570376	9622.35	0.00	1.000	0	.
age_cat						
20-24	1.05e-06	.0009586	-0.02	0.988	0	.
25-29	1.12e-06	.0010216	-0.02	0.988	0	.
30-34	6.55e-07	.0005984	-0.02	0.988	0	.
greater than 34	4.74e-07	.0004326	-0.02	0.987	0	.
0.parity	1.061601	.2752384	0.23	0.818	.6386644	1.764615
1.any_risk	.9115363	.3385852	-0.25	0.803	.4401491	1.887766
2.high_risk_num	1.397186	.6138501	0.76	0.446	.5905828	3.305426
place_hosp_comm						
hospital	.6201716	.1818529	-1.63	0.103	.3490713	1.101817
imp_gstt						
imp	2.111408	.6563113	2.40	0.016	1.148112	3.882935
_cons	4769809	4.36e+09	0.02	0.987	0	.
**least_deprived**	(base outcome)					

Table 2: Place of antenatal care recorded by deprivation score

```
Multinomial logistic regression                 Number of obs     =        765
                                                LR chi2(57)       =     153.19
                                                Prob > chi2       =     0.0000
Log likelihood = -948.68392                     Pseudo R2         =     0.0747
```

imd_3_score	RRR	Std. Err.	z	P>|z|	[95% Conf.	Interval]
**most_deprived**						
place_hosp_comm						
hospital	.4068348	.1188187	-3.08	0.002	.2295193	.7211356
ethnicity						
BA	8.688427	6.039025	3.11	0.002	2.224848	33.92985
BC	4.741514	3.985545	1.85	0.064	.91291	24.6267
BO	3.775191	4.386607	1.14	0.253	.3871507	36.81272
M	1.711281	1.391649	0.66	0.509	.3476215	8.424344
U	2.078345	.9214765	1.65	0.099	.8716061	4.955816
WB	.6403515	.2711355	-1.05	0.292	.279258	1.468356
WO	1.002714	.3973854	0.01	0.995	.4611452	2.180302
WP	2.391133	13830.83	0.00	1.000	0	.
age_cat						
20-24	1.37e-06	.0012548	-0.01	0.988	0	.
25-29	1.19e-06	.0010827	-0.01	0.988	0	.
30-34	4.47e-07	.0004081	-0.02	0.987	0	.
greater than 34	2.79e-07	.0002551	-0.02	0.987	0	.
0.parity	.6508242	.1653202	-1.69	0.091	.395589	1.070738
1.any_risk	1.684199	.5775913	1.52	0.129	.8599561	3.298455
2.high_risk_num	2.372965	.9909666	2.07	0.039	1.046704	5.379712
mod_care_4						
standard	.291514	.1930548	-1.86	0.063	.0796072	1.067497
partial CoC	.1270207	.0863271	-3.04	0.002	.0335253	.481257
imp_gstt						
imp	2.764661	.8701099	3.23	0.001	1.491925	5.123148
_cons	1.15e+07	1.05e+10	0.02	0.986	0	.
**3rd_and_4th_deciles**						
place_hosp_comm						
hospital	.4245959	.1120848	-3.25	0.001	.2530904	.7123212
ethnicity						
BA	5.239074	3.616894	2.40	0.016	1.353978	20.27205
BC	2.414152	2.031332	1.05	0.295	.4640247	12.55996
BO	7.223335	7.936432	1.80	0.072	.8385027	62.22588
M	1.297728	1.029289	0.33	0.742	.2741926	6.142027
U	2.248795	.9372376	1.94	0.052	.9935552	5.089884
WB	.689703	.2667646	-0.96	0.337	.3231713	1.471945
WO	1.130883	.4187918	0.33	0.740	.5472657	2.336883
WP	3.09e+07	1.41e+11	0.00	0.997	0	.
age_cat						
20-24	1.58e-06	.001445	-0.01	0.988	0	.
25-29	9.24e-07	.0008435	-0.02	0.988	0	.
30-34	5.30e-07	.0004843	-0.02	0.987	0	.
greater than 34	2.87e-07	.0002617	-0.02	0.987	0	.
0.parity	.66367	.1575855	-1.73	0.084	.4167141	1.056979
1.any_risk	1.480933	.4932381	1.18	0.238	.7709656	2.844695
2.high_risk_num	2.558484	.9732096	2.47	0.014	1.213942	5.392215
mod_care_4						
standard	.2897059	.1877638	-1.91	0.056	.081335	1.031898
partial CoC	.1905813	.1257227	-2.51	0.012	.052307	.6943858
imp_gstt						
imp	1.547321	.4430837	1.52	0.127	.8827429	2.71223
_cons	1.86e+07	1.70e+10	0.02	0.985	0	.
**5th_and_6th_deciles**						
place_hosp_comm						
hospital	.6201716	.1818529	-1.63	0.103	.3490713	1.101817
ethnicity						
BA	5.558974	4.138049	2.30	0.021	1.292314	23.9123
BC	4.761389	4.20312	1.77	0.077	.843985	26.86164
BO	2.572342	3.305336	0.74	0.462	.2072886	31.92139
M	.9059086	.9060892	-0.10	0.921	.1275596	6.433623
U	2.623069	1.22996	2.06	0.040	1.046359	6.575653
WB	1.151676	.5021643	0.32	0.746	.4899841	2.706942
WO	1.536157	.6463339	1.02	0.308	.6734338	3.504099
WP	1.570376	9622.35	0.00	1.000	0	.
age_cat						
20-24	1.05e-06	.0009586	-0.02	0.988	0	.
25-29	1.12e-06	.0010216	-0.02	0.988	0	.
30-34	6.55e-07	.0005984	-0.02	0.988	0	.
greater than 34	4.74e-07	.0004326	-0.02	0.987	0	.
0.parity	1.061601	.2752384	0.23	0.818	.6386644	1.764615
1.any_risk	.9115363	.3385852	-0.25	0.803	.4401491	1.887766
2.high_risk_num	1.397186	.6138501	0.76	0.446	.5905828	3.305426
mod_care_4						
standard	.2513724	.1692147	-2.05	0.040	.0671924	.9404055
partial CoC	.1490418	.1026833	-2.76	0.006	.0386249	.575107
imp_gstt						
imp	2.111408	.6563113	2.40	0.016	1.148112	3.882935
_cons	4769809	4.36e+09	0.02	0.987	0	.
**least_deprived**	(base outcome)					

The qualitative data was then used to explore women's experiences of being referred to continuity models of care, focusing on the 'how' and 'why' women with low SES were more likely to receive specialist models

at the two services evaluated, and why those with low SES were less likely to receive antenatal care in the hospital setting, despite model of care received.

Testing using qualitative data: Women reported different pathways into the specialist models including direct referrals from their GP, midwives working in other models, sexual health clinics, social workers, accident and emergency departments, and self-referral. This indicates the specialist models are known to local services and have open referral pathways. However, the qualitative findings suggest that most women in the hospital based model were not aware the model existed. When women were referred to the model of care by other healthcare professionals, they were often not aware why they had been referred, or what the aim of the model of care was.

Table 3: Qualitative quotations relating to women's experiences of referral to the specialist model of care

'I was just sent here. I think it's because of, I have specific needs, but I think he [GP] must have known what he was doing because…this is more like a special care for me rather than just normal midwife care, so I assume he did that knowingly…I do not recall a question, I mean like him asking me if this is what I wanted or not wanted.' (CBM1)
'you always have your own midwife. No I don't think I had any choice, it's just based on your location, you will have your GP, and based on your GP you will have, er, nearby hospital based on your location…I don't think there's any choice out there, it's not given.' (HBM3)
*' I think is because of my vulnerability, the mental health issues that I have…. I was actually quite taken aback that, um, because I didn't have to do anything, I didn't have to chase anyone I didn't have to fight with anyone, it [*the referral*] was just done…so obviously my doctor had done that. There must have been some sort of … I don't know, either procedure on the system or they liaise with each other. But, I thought, thank God, because I felt like it was a moment of euphoria where I myself didn't have to fight. Didn't have to run around. It was just done. (HBM8)*
'I mean the fact that I'm seeing, um, you know [HBM], I don't know if you want to class that as discrimination [laughs], because [midwife name]'s deals with so-called 'vulnerable women'. So you can look at that two ways. I was just referred. I received a letter, um, saying you know, 'You'll be seeing, um, er, a member of the [HBM],' it wasn't kind of like disclosed on the paper you know. It's just something that I kind of gathered myself.' (HBM9)

The qualitative and quantitative data appear to come to the same conclusion that community-based models of care in areas of deprivation are likely to identify women who are experiencing social risk factors that increase their chances of poor birth outcomes. The rival theory is therefore refuted in the context of this evaluation. Although the hospital-based model cared for women with at least once social risk factor, the data suggests that only those women who have a known social risk factor are cared for by the team, and these women are not necessarily in the highest deprivation centile. This may mean that the service is not identifying women who are at increased risk but are yet to disclose sensitive, often difficult social risk factors.

Analysis 2: Timing of access to maternity services

Initial programme theory: If maternity care incorporated early pregnancy care (from conception/confirmation of pregnancy), then women would not view it as a package of care for viable and continuing pregnancies and therefore see value of accessing care early in pregnancy to seek support and advice regardless of whether or not they intend to continue the pregnancy

Rival PT: If maternity care incorporated early pregnancy care (from conception/confirmation of pregnancy), then women might perceive this as added surveillance and increased importance placed on the wellbeing of the fetus, this may result in less women accessing appropriately timed abortion services.

Testing using quantitative data: Table 4 and 5 show that the majority of women booked with maternity services later than 10 weeks' gestation, the recommended time at which to book, particularly those with social and medical risk factors. No significant relationship was found between the model of care and the gestation at which women attended the booking appointment. Women attending their booking appointment after 12 weeks were significantly more likely to be receiving hospital based care.

This data contributes to the testing of the theory by revealing the context that women with social and medical risk factors are not accessing maternity services early in pregnancy. The qualitative data will go on to explore if this is due to their preferences and behaviours or system barriers.

Table 4: Gestation at booking appointment in relation to the model of care received

Multinomial logistic regression — Number of obs = 764; LR chi2(66) = 118.84; Prob > chi2 = 0.0001; Pseudo R2 = 0.0641

Log likelihood = -867.49667

booking_by_weeks	RRR	Std. Err.	z	P>|z|	[95% Conf.	Interval]
**less_than_10**	(base outcome)					
**10_to_13**						
mod_care_4						
standard	.7668591	.2321091	-0.88	0.380	.4237193	1.387883
partial CoC	.8674172	.2802026	-0.44	0.660	.460536	1.633776
ethnicity						
BA	1.334442	.5151908	0.75	0.455	.6261447	2.843969
BC	.766018	.3445405	-0.59	0.553	.3172375	1.849667
BO	.6923595	.3854517	-0.66	0.509	.232514	2.061646
M	.3655942	.2340446	-1.57	0.116	.1042511	1.282088
U	1.279369	.399387	0.79	0.430	.6938601	2.358955
WB	.9397428	.2956228	-0.20	0.843	.5072702	1.740919
WO	.8957496	.2647394	-0.37	0.710	.5018957	1.598674
WP	5067080	1.20e+10	0.01	0.995	0	.
age_cat						
20-24	1.538972	1.460734	0.45	0.650	.239498	9.889157
25-29	1.753686	1.60286	0.61	0.539	.2923871	10.5183
30-34	1.280188	1.154429	0.27	0.784	.21862	7.496481
greater than 34	1.567427	1.417993	0.50	0.619	.2661565	9.230766
0.parity	.8088913	.1438613	-1.19	0.233	.5708264	1.146242
imd_3_score						
most deprived	.9860824	.2740039	-0.05	0.960	.5719905	1.699956
3rd and 4th deciles	.776939	.2004962	-0.98	0.328	.4685184	1.28839
5th and 6th deciles	.896944	.2518034	-0.39	0.698	.5173727	1.554989
1.any_risk	.7892282	.1783753	-1.05	0.295	.5067813	1.229093
2.high_risk_num	.7087357	.1948708	-1.25	0.211	.4134678	1.214862
place_hosp_comm						
hospital	1.00336	.2024533	0.02	0.987	.6756249	1.490076
imp_gstt						
imp	1.94413	.428977	3.01	0.003	1.261553	2.996022
_cons	.751263	.7661935	-0.28	0.779	.1017827	5.54511
**13_to_20**						
mod_care_4						
standard	.891767	.3978579	-0.26	0.797	.371956	2.138017
partial CoC	.7594591	.3653578	-0.57	0.567	.2958091	1.949832
ethnicity						
BA	1.880764	1.007965	1.18	0.239	.6578797	5.376777
BC	.879995	.6116616	-0.18	0.854	.2253368	3.436594
BO	.6772392	.5960294	-0.44	0.658	.1206731	3.800787
M	.5571623	.5041002	-0.65	0.518	.09459	3.281845
U	1.528332	.7273685	0.89	0.373	.601328	3.884402
WB	1.035115	.5188021	0.07	0.945	.3875826	2.764476
WO	.9787965	.4439433	-0.05	0.962	.4023659	2.381024
WP	.6953945	3549.102	-0.00	1.000	0	.
age_cat						
20-24	.3697443	.350147	-1.05	0.293	.0577851	2.365851
25-29	.3577783	.3203188	-1.15	0.251	.0618776	2.068686
30-34	.1970194	.1730667	-1.85	0.064	.0352202	1.102111
greater than 34	.4102757	.3572851	-1.02	0.306	.0744414	2.261192
0.parity	.7365051	.1987517	-1.13	0.257	.4339826	1.249911
imd_3_score						
most deprived	1.869325	.8781225	1.33	0.183	.7444399	4.693966
3rd and 4th deciles	1.450336	.6606416	0.82	0.414	.5939339	3.541595
5th and 6th deciles	1.924385	.9115327	1.38	0.167	.7605	4.869503
1.any_risk	1.406591	.4298377	1.12	0.264	.7727702	2.560265
2.high_risk_num	.5371098	.2690423	-1.24	0.215	.2012287	1.433628
place_hosp_comm						
hospital	1.05097	.3495657	0.15	0.881	.5476087	2.017022
imp_gstt						
imp	2.732355	.9649809	2.85	0.004	1.367477	5.45952
_cons	.3516654	.4063003	-0.90	0.366	.036533	3.385121
**3**						
mod_care_4						
standard	1.276589	.6689131	0.47	0.641	.4571242	3.565067
partial CoC	1.154034	.644423	0.26	0.798	.3862778	3.447762
ethnicity						
BA	2.23247	1.209553	1.48	0.138	.7719772	6.45605
BC	.3917793	.3365533	-1.09	0.275	.072749	2.109872
BO	6.47e-07	.0004433	-0.02	0.983	0	.
M	.3680627	.4184516	-0.88	0.379	.039645	3.417083
U	1.183814	.5736468	0.35	0.728	.4579438	3.060233
WB	.7463861	.37587	-0.58	0.561	.2781697	2.002706
WO	.8127787	.3744247	-0.45	0.653	.3294938	2.004922
WP	.7171062	3822.002	-0.00	1.000	0	.
age_cat						
20-24	2.274215	2.940942	0.64	0.525	.1803371	28.67993
25-29	1.210863	1.550069	0.15	0.881	.0985013	14.88499
30-34	1.303381	1.621172	0.21	0.831	.1138492	14.92152
greater than 34	1.273376	1.59059	0.19	0.847	.1100805	14.73002
0.parity	1.794035	.509178	2.06	0.039	1.028597	3.129079
imd_3_score						
most deprived	.9455481	.400684	-0.13	0.895	.4120783	2.169639
3rd and 4th deciles	.7027879	.2798035	-0.89	0.376	.3220567	1.533615
5th and 6th deciles	.7430273	.3252906	-0.68	0.497	.3150333	1.75248
1.any_risk	1.938461	.6191795	2.07	0.038	1.036497	3.625319
2.high_risk_num	2.498193	.9487996	2.41	0.016	1.18671	5.259053
place_hosp_comm						
hospital	2.510521	.8052141	2.87	0.004	1.338915	4.707331
imp_gstt						
imp	1.328284	.4706625	0.80	0.423	.6632503	2.660141
_cons	.0502035	.0742011	-2.02	0.043	.0027711	.9095269

Table 5: Gestation at booking appointment in relation to the place of antenatal care

```
Multinomial logistic regression                 Number of obs     =        764
                                                LR chi2(66)       =     118.84
                                                Prob > chi2       =     0.0001
Log likelihood = -867.49667                     Pseudo R2         =     0.0641
```

booking_by_weeks	RRR	Std. Err.	z	P>|z|	[95% Conf.	Interval]
**less_than_10**	(base outcome)					
**10_to_13**						
place_hosp_comm						
hospital	1.00336	.2024533	0.02	0.987	.6756249	1.490076
ethnicity						
BA	1.334442	.5151908	0.75	0.455	.6261447	2.843969
BC	.766018	.3445405	-0.59	0.553	.3172375	1.849667
BO	.6923595	.3854517	-0.66	0.509	.232514	2.061646
M	.3655942	.2340446	-1.57	0.116	.1042511	1.282088
U	1.279369	.399387	0.79	0.430	.6938601	2.358955
WB	.9397428	.2956228	-0.20	0.843	.5072702	1.740919
WO	.8957496	.2647394	-0.37	0.710	.5018957	1.598674
WP	5067080	1.20e+10	0.01	0.995	0	.
age_cat						
20-24	1.538972	1.460734	0.45	0.650	.239498	9.889157
25-29	1.753686	1.60286	0.61	0.539	.2923871	10.5183
30-34	1.280188	1.154429	0.27	0.784	.21862	7.496481
greater than 34	1.567427	1.417993	0.50	0.619	.2661565	9.230766
1.parity	1.23626	.2198688	1.19	0.233	.8724162	1.751846
imd_3_score						
3rd and 4th deciles	.7879048	.1724215	-1.09	0.276	.5130983	1.209893
5th and 6th deciles	.9096035	.2323215	-0.37	0.711	.5513749	1.500573
least deprived	1.014114	.2817931	0.05	0.960	.5882506	1.748281
1.any_risk	.7892282	.1783753	-1.05	0.295	.5067813	1.229093
2.high_risk_num	.7087357	.1948708	-1.25	0.211	.4134678	1.214862
mod_care_4						
standard	.7668591	.2321091	-0.88	0.380	.4237193	1.387883
partial CoC	.8674172	.2802026	-0.44	0.660	.460536	1.633776
imp_gstt						
imp	1.94413	.428977	3.01	0.003	1.261553	2.996022
_cons	.5992325	.5848482	-0.52	0.600	.0884762	4.058488
**13_to_20**						
place_hosp_comm						
hospital	1.05097	.3495657	0.15	0.881	.5476087	2.017022
ethnicity						
BA	1.880764	1.007965	1.18	0.239	.6578797	5.376777
BC	.879995	.6116616	-0.18	0.854	.2253368	3.436594
BO	.6772392	.5960294	-0.44	0.658	.1206731	3.800787
M	.5571623	.5041002	-0.65	0.518	.09459	3.281845
U	1.528332	.7273685	0.89	0.373	.601328	3.884402
WB	1.035115	.5188021	0.07	0.945	.3875826	2.764476
WO	.9787965	.4439433	-0.05	0.962	.4023659	2.381024
WP	.6953945	3549.102	-0.00	1.000	0	.
age_cat						
20-24	.3697443	.350147	-1.05	0.293	.0577851	2.365851
25-29	.3577783	.3203188	-1.15	0.251	.0618776	2.068686
30-34	.1970194	.1730667	-1.85	0.064	.0352202	1.102111
greater than 34	.4102757	.3572851	-1.02	0.306	.0744414	2.261192
1.parity	1.357764	.3664033	1.13	0.257	.8000567	2.30424
imd_3_score						
3rd and 4th deciles	.7758606	.2442081	-0.81	0.420	.41866	1.437825
5th and 6th deciles	1.029455	.3671188	0.08	0.935	.5117479	2.070896
least deprived	.5349525	.251296	-1.33	0.183	.2130395	1.343292
1.any_risk	1.406591	.4298377	1.12	0.264	.7727702	2.560265
2.high_risk_num	.5371098	.2690423	-1.24	0.215	.2012287	1.433628
mod_care_4						
standard	.891767	.3978579	-0.26	0.797	.371956	2.138017
partial CoC	.7594591	.3653578	-0.57	0.567	.2958091	1.949832
imp_gstt						
imp	2.732355	.9649809	2.85	0.004	1.367477	5.45952
_cons	.4841614	.5020639	-0.70	0.484	.0634329	3.695438
**3**						
place_hosp_comm						
hospital	2.510521	.8052141	2.87	0.004	1.338915	4.707331
ethnicity						
BA	2.23247	1.209553	1.48	0.138	.7719772	6.45605
BC	.3917793	.3365533	-1.09	0.275	.072749	2.109872
BO	6.47e-07	.0004433	-0.02	0.983	0	.
M	.3680627	.4184516	-0.88	0.379	.039645	3.417083
U	1.183814	.5736468	0.35	0.728	.4579438	3.060233
WB	.7463861	.37587	-0.58	0.561	.2781697	2.002706
WO	.8127787	.3744247	-0.45	0.653	.3294938	2.004922
WP	.7171062	3822.002	-0.00	1.000	0	.
age_cat						
20-24	2.274215	2.940942	0.64	0.525	.1803371	28.67993
25-29	1.210863	1.550069	0.15	0.881	.0985013	14.88499
30-34	1.303381	1.621172	0.21	0.831	.1138492	14.92152
greater than 34	1.273376	1.59059	0.19	0.847	.1100805	14.73002
1.parity	.5574027	.1582005	-2.06	0.039	.3195828	.9721981
imd_3_score						
3rd and 4th deciles	.7432598	.2534893	-0.87	0.384	.3809235	1.450252
5th and 6th deciles	.7858165	.3181927	-0.60	0.552	.3553471	1.737759
least deprived	1.057588	.4481618	0.13	0.895	.4609062	2.426723
1.any_risk	1.938461	.6191795	2.07	0.038	1.036497	3.625319
2.high_risk_num	2.498193	.9487996	2.41	0.016	1.18671	5.259053
mod_care_4						
standard	1.276589	.6689131	0.47	0.641	.4571242	3.565067
partial CoC	1.154034	.644423	0.26	0.798	.3862778	3.447762
imp_gstt						
imp	1.328284	.4706625	0.80	0.423	.6632503	2.660141
_cons	.0851625	.1209387	-1.73	0.083	.0052658	1.377316

Testing using qualitative data: When interviewing women about when they accessed maternity care many spoke about wanting to be seen earlier in pregnancy, and the impact that late booking, particularly after 12 weeks, had on their emotional wellbeing- see Table 6.

Table 6: Qualitative quotations relating to women's experiences of accessing maternity care

'*the main take-away that I have from the whole experience is…the first ten weeks. That you don't have any support from anyone until you have your booking appointment. Like even information on who to call, someone to talk to' (CBM1)*<br><br>Husband- *In the beginning she was unhappy because she was telling me like, no one is caring about her…first three months.* Woman - *Yeah, there is no one.* Husband- *there is no services, nothing. No one called her. She didn't do a scan…Before 20 weeks, it's different….you feel like they're not ready for someone who is pregnant before 20 weeks. (CBM6)*<br><br>*'they [maternity services] weren't really too bothered, it was like, 'well, it's not really important till the 12 weeks and that's when we can start doing what we need to do'.'(HBM9)*<br><br>*'Not easy. You near enough have to be dying to get a [GP] appointment. It is ridiculous to get an appointment there. Sometimes it's just not even worth trying to ring up to make an appointment … it's very hard work. [HBM9)*<br><br>*I couldn't get a GP appointment because I'm still registered at my old GP, I went to my local walk-in centre and they couldn't see me because I was pregnant… So I saw a couple of people at the, is it the maternity urgent care centre there? (CBM9)*<br><br>'*And then they [GP based at community health centre] send me to the [CBM] midwife, they send me a letter to contact the midwife…Very easy.' (CMB3)*<br><br>*'I went to the GP. Um, it was horrible…they gave me a very long [late] appointment like up to one month, so all these things were not done, like I wouldn't have done my scan or anything. Yeah, and then I talked to someone, they said about self-referral. And then I self-referred myself, and then I saw the midwife at around 14 weeks.' (HBM5)*

This woman, who had no recourse to public funds, described her experience of accessing standard, hospital-based maternity care in a previous pregnancy, and then her experience of accessing the hospital-based specialist model in her current pregnancy.

Previous pregnancy under standard hospital care: '*We went to the GP when I was about six weeks pregnant, and then we couldn't get [maternity] appointment until I was about four months. So what I did was we went to private hospital to do the three-months checks … the blood test and the scan checks. And then the first appointment I had here [at hospital] was when I was four months pregnant with a scan…I don't know maybe within GP to the hospital or the process maybe took long, but I remember phoning them and then asking them, 'When can I get an appointment and see a midwife?' and they said, 'No you can't see at the moment…there's no ready documentation and everything's not ready,' so and then we went to see private hospital. (HBM3)*

This pregnancy under specialist model: *I went to GP and they confirmed I was pregnant. And then I received call, or text, I can't remember maybe both, from [HBM midwife], saying that, 'I'm your midwife, can you come and see me on this day?'…Yes it was straightaway, easy process. (HBM3)*

The data confirms the initial programme theory and challenges the notion that women with social risk factors do not prioritise their maternity care. The qualitative data did not support the rival theory.

Refined PT (demonstrating CMO configuration): If maternity care provision commences when a woman accesses services (M) regardless of her gestation (C) , even if this is in the form of a phone number for advice (M), then women would not feel unsupported (O) , anxious (O), and that the service does not value them until they have a viable pregnancy (O). This might also improve early access to safe abortion and family planning services (O).

Analysis 3: Engagement - Number of antenatal appointments attended

Initial programme theory 1): If women can access a known midwife 24/7 via a phone call or text message, then they will be better able to engage with services, care will be more personalised, they will feel more cared for, and are less likely to have to repeat their history and experience a variation of responses/advice. Additionally, if those women who have few resources, such as no phone credit, have direct, easy access to a midwife through a free phone number, or free technology such as WhatsApp, skype, etc, then their anxieties will be allayed and engagement with services improved.

Rival theory 1): If women can access a known midwife throughout their pregnancy they might become overly reliant on the midwife and misuse the service, leading to inappropriate women-midwife relationships and overburdening the midwife.

Rival theory 2): If women feel they can contact a known midwife for every concern or question they have, then they may become more anxious and less able to seek out information of their own accord, leading to disempowerment and an inability to seek readily available information provided by reliable agencies when they are no longer cared for by their known midwife. Care could also become overmedicalized if midwives feel they have to investigate every concern that women might have prioritised less if they were under standard care and only had access to information through an unknown healthcare professional.

Testing using quantitative data: Before looking to the specific programme theories related to continuity of care, relationships and trust, it is important to test the hypothesis that the specialist models of care improve women's engagement with maternity care, and if so, to what extent the level of continuity and other factors such as the place of antenatal care have an impact. It is important to remember that increased engagement is not nesesarily a 'better' outcome, for example more antenatal appointments may be too many and burdensome for the woman, or lead to overmedicalisation or inappropriate intervention. The desired outcome here is a level of engagement that is approporiate to the womans needs.

Engagement with services is tested in tables 7 and 8 through the number of antenatal appointments women attended. Table 8 showed that women accessing the specialist model of care attended a similar number of appointments to those women in the standard model of care and group practices. Women with high medical risk and any social risk factor were more likely to have more than 15 antenatal

appointments. Given that the specialist models provide care for significantly more women with social risk factors, the similar findings across model of care groups suggests that specialist model might be mitigating the affects of inequality through improved engagement with maternity care for women with low socioeconomic status and social risk factors has been levelled by the specialist model of care. Women at Service A were less likely to have more than 15 antenatal appointments. A possible explanation for this could be due to the disproportionately high numbers of women at Service B having high medical risk status at the onset of labour and therefore requiring more appointments during pregnancy. This theory will be further explored in the qualitative data and discussion section.

When analysing the effect of place of antenatal care, Table 8 showed that women receiving hospital-based antenatal care were significantly less likely to attend the recommended number of appointments compared to those attending community based care, despite the model of care received.

Table 7: Number of antenatal appointments attended in relation to the model of care accessed

**1_6**	(base outcome)					
**7_9**						
mod_care_4						
standard	1.149272	.39513	0.40	0.686	.5858305	2.254621
partial CoC	1.812476	.6855162	1.57	0.116	.863635	3.803772
ethnicity						
BA	.5885425	.2427223	-1.29	0.199	.2622598	1.32076
BC	1.099803	.5676191	0.18	0.854	.3999463	3.024324
BO	4.060241	4.736953	1.20	0.230	.4125555	39.95962
M	1.171773	.7740172	0.24	0.810	.3210558	4.276678
U	.759139	.2641498	-0.79	0.428	.3838287	1.50143
WB	.5519485	.1999764	-1.64	0.101	.2713313	1.122787
WO	.7548208	.2487774	-0.85	0.393	.3956406	1.440081
WP	2.74e+10	5.45e+15	0.00	1.000	0	.
age_cat						
20-24	1.483199	1.397323	0.42	0.676	.2340356	9.399771
25-29	1.624311	1.465304	0.54	0.591	.2772002	9.517979
30-34	1.808432	1.601011	0.67	0.503	.3189559	10.25354
greater than 34	1.769831	1.573776	0.64	0.521	.3097586	10.11208
0.parity	.8018499	.1583715	-1.12	0.264	.5444704	1.180897
imd_3_score						
most deprived	.9755324	.2950882	-0.08	0.935	.5392154	1.764904
3rd and 4th deciles	.900557	.2580345	-0.37	0.715	.5135923	1.579079
5th and 6th deciles	1.030453	.3231764	0.10	0.924	.5572748	1.905402
1.any_risk	.7663696	.1894805	-1.08	0.282	.4720452	1.244208
2.high_risk_num	1.527345	.5028294	1.29	0.198	.8011361	2.911843
place_hosp_comm						
hospital	1.610698	.3915808	1.96	0.050	1.000176	2.593892
imp_gstt						
imp	1.778247	.4527273	2.26	0.024	1.07965	2.928877
_cons	.3449901	.3564553	-1.03	0.303	.045532	2.613946
**10_14**						
mod_care_4						
standard	.9440856	.3450886	-0.16	0.875	.4611841	1.932629
partial CoC	1.765937	.7013047	1.43	0.152	.8108462	3.846024
ethnicity						
BA	.7315004	.3275001	-0.70	0.485	.3041747	1.759163
BC	.9096089	.5294628	-0.16	0.871	.2906628	2.846557
BO	11.42446	12.8353	2.17	0.030	1.263331	103.3129
M	1.689606	1.169752	0.76	0.449	.43499	6.562836
U	.8829985	.3416791	-0.32	0.748	.4136041	1.885103
WB	.922301	.3626736	-0.21	0.837	.426734	1.993371
WO	.8191682	.3035382	-0.54	0.590	.3962462	1.693484
WP	.6818543	213248.4	-0.00	1.000	0	.
age_cat						
20-24	4.078474	4.889285	1.17	0.241	.3891123	42.74845
25-29	3.71801	4.365364	1.12	0.263	.3723091	37.12935
30-34	3.286795	3.815627	1.02	0.305	.3377661	31.98373
greater than 34	3.223814	3.749286	1.01	0.314	.3299325	31.50031
0.parity	.7469259	.1584339	-1.38	0.169	.492862	1.131957
imd_3_score						
most deprived	1.3555	.4647616	0.89	0.375	.6922249	2.654311
3rd and 4th deciles	1.424572	.4613422	1.09	0.275	.7551377	2.687465
5th and 6th deciles	1.681039	.5879565	1.49	0.138	.8469609	3.336508
1.any_risk	.9725235	.2515335	-0.11	0.914	.5857946	1.614562
2.high_risk_num	2.733911	.8926909	3.08	0.002	1.441603	5.184694
place_hosp_comm						
hospital	4.356843	1.119242	5.73	0.000	2.633324	7.208409
imp_gstt						
imp	1.158933	.3096098	0.55	0.581	.6865297	1.956399
_cons	.0613308	.0796822	-2.15	0.032	.004806	.7826666
**15**						
mod_care_4						
standard	.4947072	.2332588	-1.49	0.136	.1963356	1.246514
partial CoC	2.32004	1.123352	1.74	0.082	.8981479	5.992984
ethnicity						
BA	.3915834	.2406746	-1.53	0.127	.1173977	1.306137
BC	1.330121	.8859924	0.43	0.668	.3605009	4.907675
BO	14.03244	16.56205	2.24	0.025	1.388309	141.8339
M	.3621583	.4306236	-0.85	0.393	.0352192	3.724071
U	.4462132	.2238845	-1.61	0.108	.1669005	1.192963
WB	.4952449	.2435218	-1.43	0.153	.1889154	1.298293
WO	.3881658	.1850012	-1.99	0.047	.1525217	.9878774
WP	.6593021	273021.6	-0.00	1.000	0	.
age_cat						
20-24	.4101014	.4703021	-0.78	0.437	.0433249	3.881902
25-29	1.199888	1.214551	0.18	0.857	.1650205	8.724565
30-34	.9813159	.9625507	-0.02	0.985	.1435107	6.710166
greater than 34	1.388501	1.361578	0.33	0.738	.203165	9.489499
0.parity	1.135431	.3389281	0.43	0.670	.6325219	2.038196
imd_3_score						
most deprived	.8194501	.3920441	-0.42	0.677	.3208395	2.092942
3rd and 4th deciles	1.249745	.5321265	0.52	0.601	.5424875	2.879074
5th and 6th deciles	1.721278	.7826422	1.19	0.232	.7060268	4.19644
1.any_risk	1.971849	.670567	2.00	0.046	1.012524	3.840095
2.high_risk_num	4.129237	1.634349	3.58	0.000	1.900924	8.969637
place_hosp_comm						
hospital	7.886996	2.707958	6.01	0.000	4.023983	15.45849
imp_gstt						
imp	.5237601	.1927356	-1.76	0.079	.2546264	1.077361
_cons	.1135639	.1406873	-1.76	0.079	.010017	1.287483

Table 8: Number of antenatal appointments attended in relation to the place of antenatal care

ante_appt_cat	RRR	Std. Err.	z	P>|z|	[95% Conf.	Interval]
**1_6**						
place_hosp_comm						
hospital	.6193839	.1505954	-1.97	0.049	.3845926	.9975136
ethnicity						
BA	1.693631	.6982336	1.28	0.201	.7549079	3.799652
BC	.9099711	.469674	-0.18	0.855	.3308926	2.502466
BO	.2459387	.2869793	-1.20	0.229	.0249794	2.421427
M	.8530948	.5635031	-0.24	0.810	.2337463	3.113507
U	1.317993	.4586435	0.79	0.428	.6663566	2.606871
WB	1.817802	.658774	1.65	0.099	.8934492	3.698479
WO	1.325525	.4369186	0.85	0.393	.6947288	2.529067
WP	5.47e-06	.0028133	-0.02	0.981	0	.
age_cat						
20-24	.6736911	.6347487	-0.42	0.675	.1062827	4.270307
25-29	.6160015	.5557416	-0.54	0.591	.1051111	3.610066
30-34	.553214	.4898026	-0.67	0.504	.0975572	3.13709
greater than 34	.5657324	.5031052	-0.64	0.522	.0990007	3.232836
1.parity	.8019261	.1583226	-1.12	0.264	.5446073	1.180824
imd_3_score						
3rd and 4th deciles	1.0851	.2609095	0.34	0.734	.6773299	1.738357
5th and 6th deciles	.942631	.2663404	-0.21	0.834	.5417946	1.640019
least deprived	.9741613	.2946691	-0.09	0.931	.5384622	1.762408
1.any_risk	1.305822	.3228398	1.08	0.280	.8043415	2.119959
2.high_risk_num	.6512724	.2144145	-1.30	0.193	.3416069	1.241648
imp_gstt						
imp	.5628822	.1432485	-2.26	0.024	.3418175	.9269167
mod_care_4						
standard	.8709317	.2994596	-0.40	0.688	.4439239	1.708676
partial CoC	.551143	.2084863	-1.57	0.115	.2625861	1.156796
_cons	3.705105	3.624279	1.34	0.181	.5447133	25.20189
**7_9**	(base outcome)					
**10_14**						
place_hosp_comm						
hospital	2.706802	.7010945	3.84	0.000	1.629239	4.497053
ethnicity						
BA	1.250261	.5609235	0.50	0.619	.5189353	3.01223
BC	.8264228	.4639844	-0.34	0.734	.2749832	2.483696
BO	2.816899	1.91737	1.52	0.128	.7419706	10.69439
M	1.443782	.9667798	0.55	0.583	.3886181	5.363892
U	1.162341	.4419722	0.40	0.692	.5516585	2.449047
WB	1.664648	.6550648	1.30	0.195	.7697707	3.599842
WO	1.084449	.3876004	0.23	0.821	.5382388	2.184957
WP	3.74e-06	.0023281	-0.02	0.984	0	.
age_cat						
20-24	2.750417	3.611257	0.77	0.441	.2097893	36.059
25-29	2.286663	2.944347	0.64	0.521	.1833074	28.5249
30-34	1.815692	2.31376	0.47	0.640	.1493981	22.0668
greater than 34	1.819661	2.322906	0.47	0.639	.1490666	22.21266
1.parity	1.072536	.2310358	0.33	0.745	.7031594	1.635949
imd_3_score						
3rd and 4th deciles	1.137171	.2975716	0.49	0.623	.6809041	1.899178
5th and 6th deciles	1.179217	.3566926	0.54	0.586	.651809	2.133376
least deprived	.720963	.2481584	-0.95	0.342	.3672196	1.415468
1.any_risk	1.26802	.3372343	0.89	0.372	.7529157	2.135532
2.high_risk_num	1.801903	.6252472	1.70	0.090	.9127898	3.557068
imp_gstt						
imp	.6524645	.1786534	-1.56	0.119	.381493	1.115905
mod_care_4						
standard	.8213091	.3325921	-0.49	0.627	.3713723	1.816368
partial CoC	.9744801	.4157976	-0.06	0.952	.4222568	2.248895
_cons	.2299289	.3119432	-1.08	0.279	.0160981	3.28408
**15**						
place_hosp_comm						
hospital	4.907966	1.683372	4.64	0.000	2.50582	9.612873
ethnicity						
BA	.6804993	.4201494	-0.62	0.533	.2029011	2.282291
BC	1.210251	.7847556	0.29	0.769	.3395752	4.313351
BO	3.471839	2.712416	1.59	0.111	.7508414	16.05354
M	.3102073	.3671998	-0.99	0.323	.0304835	3.156746
U	.5870266	.2926949	-1.07	0.285	.2209244	1.559811
WB	.8915576	.4378813	-0.23	0.815	.3404777	2.334587
WO	.5134041	.2397176	-1.43	0.153	.2055976	1.282037
WP	3.62e-06	.0033946	-0.01	0.989	0	.
age_cat						
20-24	.2764452	.3510455	-1.01	0.311	.0229459	3.330521
25-29	.7364513	.8450313	-0.27	0.790	.0777041	6.979816
30-34	.5412187	.6054519	-0.55	0.583	.0604155	4.848384
greater than 34	.7835655	.876771	-0.22	0.827	.0874225	7.02308
1.parity	.7035639	.211548	-1.17	0.242	.3902687	1.268362
imd_3_score						
3rd and 4th deciles	1.649296	.6300238	1.31	0.190	.7800879	3.487016
5th and 6th deciles	2.010318	.8698524	1.61	0.107	.8609046	4.69434
least deprived	1.195039	.5724279	0.37	0.710	.4673623	3.055699
1.any_risk	2.56915	.8901076	2.72	0.006	1.302813	5.066368
2.high_risk_num	2.738223	1.123561	2.45	0.014	1.22518	6.119809
imp_gstt						
imp	.2950832	.1098794	-3.28	0.001	.1422278	.6122156
mod_care_4						
standard	.4299578	.2149768	-1.69	0.091	.1613724	1.145572
partial CoC	1.278626	.6479404	0.49	0.628	.4735869	3.452134
_cons	.3905271	.5050302	-0.73	0.467	.0309654	4.925227

The qualitative data was interrogated to explore mechanisms that may lead to increased engagement: for example, did women find the number of appointments appropriate for their individual, holistic needs? If they missed an appointment, did they feel they could rebook it without reproach? These were then explored in relation to the level of continuity of care received.

Testing using qualitative data:

Women from both specialist models of care expressed ease of contacting and booking appointments with their midwife or a midwife from the team, particularly when comparing this to their experiences of standard care or other healthcare services. This might be through phone call, text messaging or emailing. This was also valued by women's family members who felt able to contact the specialist model. This women's partner described the benefit of being able to contact a known midwife, and described how this interaction was enhanced by the fact the team communicated with one another and were knowledgeable about their situation. Women who do not speak English particularly valued the ability to text their midwife as it gave them the opportunity to use translation technology. Those who had multiple services involved in their care and needed to attend numerous appointments commented on how the regular care she received from the team made them feel looked after and that they could rely on them to remind her of her appointments:

Table 9: Qualitative quotations relating to women's experiences of engaging with the specialist model of care

'I will book my appointment with midwife through text messages like you're texting a friend, and then it's so informal but also very efficient…. sometimes I can't make the appointment, or my midwife can't make the appointment, then we text each other beforehand' (HBM3) <br>*'the absolute amazing thing about the [specialist model] is that I had a phone number I could call…by the end of the first week pretty much knew all of them on a first name basis. Whoever would answer the phone was reassuring, they were able to talk me through things… if we didn't have the like phone number that we could call the first few weeks it would have been a lot worse, a lot more difficult. We would have ended up in A&E a lot more often than we did… which is zero…it's just like every concern, it's kind of like calling a friend…regardless of which midwife was picking up at the time' (Partner of CBM6)* <br>*'the fact that I see someone regularly. I feel like I'm being looked after as well… I can rely on them to look after me, remind me of appointments and stuff like that as I really struggle … [the midwives] text, call, put it in my notes and*

*what-not so …I am remembering … or I do actually go to these appointments… whereas my other midwife appointments [*under standard care*] were just the normal basic appointments…I was visiting hospital more [because] when I did try to get in contact with someone it was impossible, so I just had to keep running to the hospital.'(CBM1)*

'I'll tell [CBM midwife] a convenient time slot for me. Last week it was at home. Sometimes if my husband is not there, it's better to be at home, because travelling with the kids is not easy.' (CBM2)

'it's been very flexible. Um, they have located a lot of time with my appointments because of my circumstances so … that's really really really helpful particularly with the midwives, it is easy.' (CMB5)

'at one point I was a bit worried, so I emailed them, they called me back, they got me in sort of within the day to do some blood tests' (CBM6)

[concerned she had a urine infection] I called up and, again because I've not had like any really serious problems there's always that hesitation like I don't want to bother anyone, but the team are so lovely they're like, 'You can literally just call us to chat.' and she was like 'Someone is in clinic…you could come in today between these hours and also tomorrow'… I literally got a sample, knocked on the door, gave them that, took a seat for five minutes…and when they had a gap came out and just chatted to me.' (CBM9)

'[an appointment] every two weeks…It's too much. Yeah, and um, after the baby's due date they started every week! It was too much. No medical reason, I think it's a requirement… I prefer, um, when I want to see them, like maybe every three weeks. And then if I need to see them then maybe I can call them and then make appointment before three weeks.' (HBM3)

I'm struggling a little bit. Just I think more, I'm feeling a bit low and irritable. I feel a bit, this week I do feel a bit emotional…it's just me, being on my own. I haven't seen [HBM midwife] in probably about two weeks, um, the last time I had an appointment was with a different midwife because she was on holiday. I was meant to see her today but she, I think she's not feeling very well….how I am feeling now I think more [appointments] would be good. I think [antenatal care] follows a schedule. (HBM2)

Given both the qualitative and quantitative findings above there is no evidence to support the rival theories put forward, therefore these have been refuted. The initial programme theory has been refined to provide more detailed contexts and mechanisms, and how they trigger outcomes.

Refined theory: If women have 24/7 access to a small team of midwives whom they have had the opportunity to meet during pregnancy and are encouraged to contact midwives via a phone call, text message or free technology, then their engagement with services will improve through needs-based communication and appropriately timed antenatal appointments. For women who have multiple social and medical needs, this open access can work both ways through midwives reminding them of appointments, this leads to women feeling more 'cared for'.

Analysis 4: The quality of relational continuity and missed appointments

Initial programme theory: If women feel they have a continued supportive presence throughout pregnancy and the perinatal period, either with a midwife, GP or other healthcare professional, then they will feel better supported and have reduced feelings of anxiety, increased sense of control, and enhanced self-beliefs and wellbeing.

Rival theory: If women have continued supportive presence from a known healthcare professional, then they will become overly reliant on that person and feel anxious when they are not on duty. it is more important that the whole service is perceived as safe, respectful, understanding, and kind, rather than one trusted HCP in a wider toxic environment.

Initial programme theory If women have a level of trust and confidence in their HCP's and do not fear judgement, for example their concerns are listened to on an individual level, they receive meaningful information, and they are able to rebook missed appointments with ease and without reproach, then they will perceive the maternity environment as a place of safety and their engagement with flexible services will improve.

Additional related programme theory identified in focus groups with healthcare professionals: If midwives are able to work flexibly, then they are able to meet women's individual needs and increase safety through spending time care planning and coordinating support that may not be available on demand (for example during an allocated appointment time in the standard maternity care model).

Rival theory: If women feel they can rebook appointments at will then they will not prioritise their maternity care appointments and have less engagement.

Testing using quantitative data: In order to test these programme theories the quality of continuity of care was analysed first through the number of antenatal appointments women received with a known healthcare professional, and whether women were supported in labour by a known healthcare professional. Table 10 shows a significant relationship between the model of care and the number of appointments with a known healthcare professional. The aim of both the group practice and specialist models of care appear to be being met with women more likely to receive more antenatal appointments with a known healthcare professional if they experienced one of these models. Conversely, the fully

adjusted results show that women accessing standard maternity care, Black women, and those in the most deprived deciles were the least likely to see a known healthcare professional for their antenatal appointments. Women receiving care in the specialist model were more likely to be looked after in labour by a known healthcare professional compared to the group practice model. After adjusting for the service provider attended, we found that women in the group practice models were the least likely group to know the person looking after them in labour. This is unsurprising given that these models of care are often set in the community with midwives not working in intrapartum settings. Table 11 shows that women receiving antenatal care based in the hospital were less likely to see a known healthcare professional for their antenatal appointments than those based in the community setting. Importantly, place of antenatal care made no difference the number of women who knew the person looking after them in labour.

No relationship was found between model of care, the place of antenatal care, and the number of missed appointments. Again, this suggests the specialist model of care, that is more likely to care for women with low SES who are known to struggle to enagage, could be mitigating the effects of inequality by reducing the number of appointments women miss

Table 10: Number of appointments and support in labour by known healthcare professional

noofapptswithknown~p	RRR	Std. Err.	z	P>|z|	[95% Conf.	Interval]
**0**	(base outcome)					
**2**						
mod_care_4						
standard	2.12497	1.42054	1.13	0.260	.5732255	7.877349
partial CoC	1.814651	1.307945	0.83	0.408	.4418483	7.452689
ethnicity						
BA	.6897641	.3674707	-0.70	0.486	.2427864	1.959642
BC	1.740153	1.212201	0.80	0.426	.4442582	6.816153
BO	1.674624	1.297217	0.67	0.506	.3669016	7.643372
M	3.514697	3.072361	1.44	0.150	.6335969	19.49678
U	1.185132	.5570245	0.36	0.718	.4717297	2.977422
WB	1.151437	.5773619	0.28	0.779	.4309476	3.076492
WO	1.175301	.507662	0.37	0.708	.5040567	2.740431
WP	2.09e+07	1.20e+11	0.00	0.998	0	.
age_cat						
20-24	2087928	2.43e+09	0.01	0.990	0	.
25-29	5006335	5.83e+09	0.01	0.989	0	.
30-34	5239593	6.10e+09	0.01	0.989	0	.
greater than 34	3618132	4.21e+09	0.01	0.990	0	.
0.parity	1.822294	.481216	2.27	0.023	1.086026	3.057712
imd_3_score						
most deprived	.9973948	.4150319	-0.01	0.995	.4412336	2.25458
3rd and 4th deciles	1.119732	.438289	0.29	0.773	.5199173	2.411538
5th and 6th deciles	1.117686	.4667524	0.27	0.790	.4930066	2.533883
1.any_risk	.8629611	.2652524	-0.48	0.632	.4724473	1.576264
2.high_risk_num	1.664787	1.116654	0.76	0.447	.4471117	6.198709
highriskatbirth						
Y	.8888816	.2524653	-0.41	0.678	.5094256	1.550983
place_hosp_comm						
hospital	.2463731	.11066	-3.12	0.002	.1021577	.5941768
imp_gstt						
imp	130.1612	68.8159	9.21	0.000	46.17993	366.8679
_cons	3.50e-09	4.07e-06	-0.02	0.987	0	.
**3**						
mod_care_4						
standard	.3452801	.1990772	-1.84	0.065	.1115313	1.068923
partial CoC	.8252684	.5081991	-0.31	0.755	.2468462	2.759078
ethnicity						
BA	.4432498	.2848524	-1.27	0.205	.1257847	1.561957
BC	.3192784	.3801943	-0.96	0.338	.0309432	3.294382
BO	.5940215	.7219469	-0.43	0.668	.0548643	6.431527
M	2.919087	2.948978	1.06	0.289	.4030225	21.1429
U	.9494037	.5401176	-0.09	0.927	.3113155	2.89535
WB	1.232423	.7186215	0.36	0.720	.393031	3.864492
WO	1.116556	.5604128	0.22	0.826	.4174958	2.98613
WP	.2176232	3289.857	-0.00	1.000	0	.
age_cat						
20-24	4070954	6.23e+09	0.01	0.992	0	.
25-29	1.60e+07	2.45e+10	0.01	0.991	0	.
30-34	8761015	1.34e+10	0.01	0.992	0	.
greater than 34	1.37e+07	2.10e+10	0.01	0.991	0	.
0.parity	1.645874	.5272247	1.56	0.120	.878477	3.083635
imd_3_score						
most deprived	2.243444	1.183107	1.53	0.125	.7980385	6.306765
3rd and 4th deciles	1.962508	.9973083	1.33	0.185	.724849	5.313436
5th and 6th deciles	1.874744	1.010673	1.17	0.244	.651718	5.392923
1.any_risk	1.249665	.4504208	0.62	0.536	.6165841	2.532765
2.high_risk_num	2.051514	1.692983	0.87	0.384	.4070358	10.3399
highriskatbirth						
Y	.9102559	.3097016	-0.28	0.782	.4672554	1.773261
place_hosp_comm						
hospital	.232326	.1178467	-2.88	0.004	.0859663	.6278666
imp_gstt						
imp	158.5037	106.957	7.51	0.000	42.23336	594.8719
_cons	1.50e-09	2.29e-06	-0.01	0.989	0	.
**4**						
mod_care_4						
standard	.1071422	.087908	-2.72	0.006	.0214569	.5350006
partial CoC	1.723088	1.290399	0.73	0.467	.3970573	7.47759
ethnicity						
BA	.303219	.2529282	-1.43	0.153	.0591197	1.55518
BC	1.391403	1.353061	0.34	0.734	.2068748	9.358329
BO	1.017385	1.394737	0.01	0.990	.0692739	14.94173
M	3.92e-06	.0041128	-0.01	0.991	0	.
U	1.080812	.7921639	0.11	0.916	.256966	4.545951
WB	1.750317	1.226933	0.80	0.425	.4430405	6.914965
WO	.6510023	.4259005	-0.66	0.512	.1805956	2.346702
WP	.8365924	16015.77	-0.00	1.000	0	.
age_cat						
20-24	1.13e+07	2.01e+10	0.01	0.993	0	.
25-29	9275790	1.65e+10	0.01	0.993	0	.
30-34	1.53e+07	2.72e+10	0.01	0.993	0	.
greater than 34	7097596	1.26e+10	0.01	0.993	0	.
0.parity	1.553959	.6429303	1.07	0.287	.6906625	3.496335
imd_3_score						
most deprived	2.797857	1.835835	1.57	0.117	.7732198	10.1239
3rd and 4th deciles	1.791035	1.137307	0.92	0.359	.5159303	6.217517
5th and 6th deciles	1.423641	.9797758	0.51	0.608	.3694775	5.485459
1.any_risk	.9706877	.4723698	-0.06	0.951	.3739863	2.519436
2.high_risk_num	5.072918	6.282963	1.31	0.190	.4477329	57.47735
highriskatbirth						
Y	1.834165	.8026277	1.39	0.166	.777954	4.32437
place_hosp_comm						
hospital	.306421	.1568288	-2.31	0.021	.1123742	.8355462
imp_gstt						
imp	293.5531	331.2947	5.03	0.000	32.14013	2681.178
_cons	3.35e-10	5.95e-07	-0.01	0.990	0	.
**5**						
mod_care_4						
standard	.0254159	.019587	-4.77	0.000	.005612	.1151047
partial CoC	.8291245	.5360572	-0.29	0.772	.2335012	2.944086
ethnicity						
BA	.1758425	.1385612	-2.21	0.027	.037531	.8238682
BC	.5001275	.5111954	-0.68	0.498	.0674593	3.707827
BO	1.5265	1.869733	0.35	0.730	.1383895	16.838
M	2.457489	3.421256	0.65	0.518	.1604981	37.6282
U	.8435105	.6012533	-0.24	0.811	.2086195	3.410562
WB	1.151793	.7796169	0.21	0.835	.3056468	4.340395
WO	.4221212	.2640412	-1.38	0.168	.1238801	1.438376
WP	1.229522	22017.75	0.00	1.000	0	.
age_cat						
20-24	2.161349	3.465587	0.48	0.631	.093299	50.06944
25-29	3.170843	5.019867	0.73	0.466	.1424326	70.58951
30-34	3.298027	5.106225	0.77	0.441	.1586235	68.57108
greater than 34	4.282206	6.658542	0.94	0.350	.2032856	90.20458
0.parity	.8574591	.3721807	-0.35	0.723	.3662257	2.007604
imd_3_score						
most deprived	3.896169	2.551882	2.08	0.038	1.079256	14.06537
3rd and 4th deciles	1.732054	1.141455	0.83	0.405	.475997	6.302582
5th and 6th deciles	1.696077	1.193329	0.75	0.453	.4271259	6.734965
1.any_risk	1.753116	.8077984	1.22	0.223	.7105512	4.325399
2.high_risk_num	3.22637	4.079044	0.93	0.354	.2707415	38.44798
highriskatbirth						
Y	2.248574	.9914991	1.84	0.066	.9474877	5.336308
place_hosp_comm						
hospital	.3523471	.1807112	-2.03	0.042	.128946	.9627944
imp_gstt						
imp	366.4902	422.7924	5.12	0.000	38.20274	3515.848
_cons	.0019683	.0042857	-2.86	0.004	.0000276	.1404198

Table 11: Number of appointments with a known healthcare professional in relation to place of care

deliver_named_mw	RRR	Std. Err.	z	P>|z|	[95% Conf.	Interval]
**yes**						
place_hosp_comm						
hospital	.8999399	.1918592	-0.49	0.621	.5925771	1.366728
ethnicity						
BA	1.107203	.4571936	0.25	0.805	.4928825	2.487202
BC	.8993323	.4435732	-0.22	0.830	.3420474	2.36458
BO	1.875314	1.157879	1.02	0.309	.5591325	6.289745
M	1.222475	.8163165	0.30	0.764	.3302511	4.525181
U	1.193273	.4079359	0.52	0.605	.6105841	2.33203
WB	1.595939	.5570766	1.34	0.181	.8051871	3.163266
WO	.8055546	.2648301	-0.66	0.511	.42292	1.534376
WP	.0000219	.0132068	-0.02	0.986	0	.
age_cat						
20-24	.094338	.0939308	-2.37	0.018	.0134017	.6640707
25-29	.1635071	.157408	-1.88	0.060	.0247791	1.078915
30-34	.1665161	.1580722	-1.89	0.059	.0259071	1.070272
greater than 34	.1411916	.1340099	-2.06	0.039	.0219737	.9072226
1.parity	1.07912	.212864	0.39	0.699	.7331014	1.588456
imd_3_score						
3rd and 4th deciles	.9407191	.2249157	-0.26	0.798	.5887716	1.503049
5th and 6th deciles	.8024294	.2263074	-0.78	0.435	.4616835	1.394663
least deprived	1.089825	.3345874	0.28	0.779	.5970758	1.989227
1.any_risk	1.142474	.2769763	0.55	0.583	.7103706	1.837418
2.high_risk_num	1.267832	.4231819	0.71	0.477	.6590892	2.438816
highriskatbirth						
Y	.4433954	.1015607	-3.55	0.000	.2830229	.6946416
mod_care_4						
standard	.5923736	.206196	-1.50	0.133	.2994372	1.171887
partial CoC	.4508229	.1653001	-2.17	0.030	.2197363	.9249327
imp_gstt						
imp	.0891558	.0219513	-9.82	0.000	.0550266	.1444528
_cons	44.86345	46.44374	3.67	0.000	5.898045	341.2536
**no**	(base outcome)					

noofapptswithknown~p	RRR	Std. Err.	z	P>|z|	[95% Conf.	Interval]
**0**	(base outcome)					
**2**						
place_hosp_comm						
hospital	.2467317	.1107829	-3.12	0.002	.1023374	.5948611
ethnicity						
BA	.6924964	.3690025	-0.69	0.490	.2436956	1.967829
BC	1.738724	1.210712	0.79	0.427	.4441403	6.806772
BO	1.67289	1.295196	0.66	0.506	.3668126	7.6294
M	3.508557	3.064964	1.44	0.151	.6332072	19.44067
U	1.182606	.5555711	0.36	0.721	.4709314	2.969765
WB	1.160279	.5821279	0.30	0.767	.4340132	3.101858
WO	1.174625	.5071569	0.37	0.709	.503946	2.737881
WP	2.05e+07	1.16e+11	0.00	0.998	0	.
age_cat						
20-24	2071967	2.40e+09	0.01	0.990	0	.
25-29	4946104	5.74e+09	0.01	0.989	0	.
30-34	5173676	6.00e+09	0.01	0.989	0	.
greater than 34	3587930	4.16e+09	0.01	0.990	0	.
1.parity	.5471574	.1444873	-2.28	0.022	.3260891	.9180962
imd_3_score						
3rd and 4th deciles	1.127121	.3644407	0.37	0.711	.5980605	2.124203
5th and 6th deciles	1.121483	.4141216	0.31	0.756	.5438459	2.312648
least deprived	1.000974	.4163176	0.00	0.998	.4429937	2.261769
1.any_risk	.8623928	.2650129	-0.48	0.630	.4722057	1.574994
2.high_risk_num	1.668212	1.119368	0.76	0.446	.4478124	6.214504
highriskatbirth						
Y	.8882196	.2522359	-0.42	0.676	.5090926	1.549687
mod_care_4						
standard	2.13658	1.428092	1.14	0.256	.5764682	7.918868
partial CoC	1.819327	1.310825	0.83	0.406	.4432208	7.467951
imp_gstt						
imp	129.3069	68.29544	9.21	0.000	45.92469	364.0801
_cons	6.43e-09	7.46e-06	-0.02	0.987	0	.
**3**						
place_hosp_comm						
hospital	.2324609	.117893	-2.88	0.004	.0860323	.6281138
ethnicity						
BA	.4448746	.2859349	-1.26	0.208	.1262245	1.567948
BC	.3192713	.3800974	-0.96	0.338	.0309593	3.292522
BO	.5933663	.7209856	-0.43	0.668	.0548337	6.420933
M	2.913797	2.942518	1.06	0.290	.4025942	21.08876
U	.9475622	.5389036	-0.09	0.925	.3108185	2.88874
WB	1.240831	.7237501	0.37	0.711	.3955715	3.892245
WO	1.116068	.5599861	0.22	0.827	.4174467	2.983873
WP	.2178736	3262.333	-0.00	1.000	0	.
age_cat						
20-24	4050570	6.18e+09	0.01	0.992	0	.
25-29	1.59e+07	2.43e+10	0.01	0.991	0	.
30-34	8673986	1.32e+10	0.01	0.992	0	.
greater than 34	1.36e+07	2.08e+10	0.01	0.991	0	.
1.parity	.6059389	.1940902	-1.56	0.118	.3234282	1.135219
imd_3_score						
3rd and 4th deciles	.8780057	.3331795	-0.34	0.732	.4173393	1.847164
5th and 6th deciles	.8363101	.3654243	-0.41	0.682	.35517	1.969239
least deprived	.445141	.2346754	-1.54	0.125	.158398	1.250966
1.any_risk	1.249047	.4500963	0.62	0.537	.6163776	2.531108
2.high_risk_num	2.048627	1.690593	0.87	0.385	.4064657	10.32528
highriskatbirth						
Y	.9095566	.3094149	-0.28	0.780	.4669455	1.771713
mod_care_4						
standard	.3472074	.2002005	-1.83	0.067	.1121461	1.074963
partial CoC	.8268466	.5089512	-0.31	0.757	.2474472	2.762914
imp_gstt						
imp	157.4338	106.1708	7.50	0.000	41.98182	590.384
_cons	5.57e-09	8.51e-06	-0.01	0.990	0	.
**4**						
place_hosp_comm						
hospital	.3067113	.1569143	-2.31	0.021	.112526	.8360008
ethnicity						
BA	.3042188	.2537595	-1.43	0.154	.0593156	1.560281
BC	1.390508	1.351873	0.34	0.735	.2068341	9.348128
BO	1.016634	1.393221	0.01	0.990	.0692878	14.9167
M	3.92e-06	.0041111	-0.01	0.991	0	.
U	1.078893	.7905951	0.10	0.917	.2565851	4.536542
WB	1.759525	1.233466	0.81	0.420	.4453323	6.951948
WO	.6506795	.4255932	-0.66	0.511	.1805583	2.344859
WP	.8377351	15882.32	-0.00	1.000	0	.
age_cat						
20-24	1.13e+07	2.00e+10	0.01	0.993	0	.
25-29	9189086	1.63e+10	0.01	0.993	0	.
30-34	1.52e+07	2.68e+10	0.01	0.993	0	.
greater than 34	7048785	1.25e+10	0.01	0.993	0	.
1.parity	.641973	.2655857	-1.07	0.284	.2853466	1.444311
imd_3_score						
3rd and 4th deciles	.6420809	.3098632	-0.92	0.359	.2493483	1.653381
5th and 6th deciles	.5092814	.2955374	-1.16	0.245	.1633064	1.588226
least deprived	.3570741	.2342331	-1.57	0.116	.0987161	1.291602
1.any_risk	.9700013	.4719995	-0.06	0.950	.3737493	2.51747
2.high_risk_num	5.082526	6.295067	1.31	0.189	.4485454	57.59076
highriskatbirth						
Y	1.833258	.8021245	1.39	0.166	.7776577	4.32174
mod_care_4						
standard	.1076106	.0883012	-2.72	0.007	.0215472	.5374261
partial CoC	1.724272	1.290989	0.73	0.467	.3974641	7.480207
imp_gstt						
imp	292.8011	330.4324	5.03	0.000	32.06074	2674.065
_cons	1.47e-09	2.60e-06	-0.01	0.991	0	.
**5**						
place_hosp_comm						
hospital	.3526896	.180827	-2.03	0.042	.1291142	.9634099
ethnicity						
BA	.1763719	.1389716	-2.20	0.028	.0376468	.8262865
BC	.4999369	.5108733	-0.68	0.497	.0674673	3.704565
BO	1.525588	1.867909	0.34	0.730	.1384323	16.81267
M	2.455025	3.417835	0.65	0.519	.1603359	37.59076
U	.8423045	.6002791	-0.24	0.810	.2083768	3.404778
WB	1.157315	.7833931	0.22	0.829	.3070921	4.361488
WO	.4219462	.2638713	-1.38	0.168	.1238636	1.437376
WP	1.23247	21857.73	0.00	1.000	0	.
age_cat						
20-24	2.160438	3.462691	0.48	0.631	.0933811	49.98325
25-29	3.158476	4.997976	0.73	0.467	.1420808	70.21337
30-34	3.283103	5.08069	0.77	0.442	.1581348	68.16189
greater than 34	4.27602	6.645841	0.93	0.350	.2032789	89.94712
1.parity	1.163698	.5050895	0.35	0.727	.4970344	2.724547
imd_3_score						
3rd and 4th deciles	.4457214	.2104513	-1.71	0.087	.1766693	1.124516
5th and 6th deciles	.4357005	.2474089	-1.46	0.143	.1431661	1.325976
least deprived	.256448	.1679203	-2.08	0.038	.0710622	.9254647
1.any_risk	1.752219	.8072836	1.22	0.223	.7102678	4.322695
2.high_risk_num	3.227525	4.080159	0.93	0.354	.2708952	38.45369
highriskatbirth						
Y	2.247512	.9909025	1.84	0.066	.9471462	5.333191
mod_care_4						
standard	.0255008	.0196506	-4.76	0.000	.0056316	.1154723
partial CoC	.8295332	.5361767	-0.29	0.772	.2336962	2.94453
imp_gstt						
imp	365.364	421.434	5.12	0.000	38.09746	3503.93
_cons	.0065914	.0132469	-2.50	0.012	.0001283	.3385662

Table 12: Number of missed appointments in relation to model of care received

miss_appt_4_cat	RRR	Std. Err.	z	P>|z|	[95% Conf.	Interval]
0	(base outcome)					
1						
mod_care_4						
standard	1.466618	.6491433	0.87	0.387	.6159775	3.491961
partial CoC	1.976347	.9047997	1.49	0.137	.8056957	4.847919
ethnicity						
BA	2.485515	1.125998	2.01	0.044	1.022826	6.03992
BC	1.94952	1.063413	1.22	0.221	.6692974	5.678536
BO	.8342489	.7124188	-0.21	0.832	.1564588	4.448272
M	.7729222	.6665585	-0.30	0.765	.1425838	4.189878
U	.9045829	.3983115	-0.23	0.820	.3816297	2.144147
WB	.909001	.4042185	-0.21	0.830	.3802318	2.173103
WO	1.45843	.5647411	0.97	0.330	.6827766	3.115247
WP	1.20e-06	.0036594	-0.00	0.996	0	.
age_cat						
20-24	.2842646	.2541574	-1.41	0.159	.0492804	1.639726
25-29	.2027736	.172672	-1.87	0.061	.0382094	1.0761
30-34	.2833981	.2325385	-1.54	0.124	.0567485	1.415269
greater than 34	.2004958	.1662139	-1.94	0.053	.0394864	1.018037
0.parity	.7442038	.1735291	-1.27	0.205	.4712092	1.175358
imd_3_score						
most deprived	1.779518	.6478759	1.58	0.113	.8717703	3.632474
3rd and 4th deciles	.7533867	.2805336	-0.76	0.447	.3631296	1.563055
5th and 6th deciles	1.253867	.4778692	0.59	0.553	.5940794	2.646417
1.any_risk	1.38075	.3691036	1.21	0.227	.8176568	2.331628
2.high_risk_num	.5591242	.2403068	-1.35	0.176	.2408071	1.298217
place_hosp_comm						
hospital	.9538957	.2571112	-0.18	0.861	.5624315	1.617827
imp_gstt						
imp	1.740019	.4995678	1.93	0.054	.99122	3.054485
_cons	.3266529	.3430343	-1.07	0.287	.0417059	2.558439
2						
mod_care_4						
standard	1.819736	1.136977	0.96	0.338	.5347799	6.192154
partial CoC	.8625367	.5949905	-0.21	0.830	.2231548	3.333872
ethnicity						
BA	11.34923	9.569888	2.88	0.004	2.173787	59.25374
BC	3.389846	3.648733	1.13	0.257	.4111224	27.95045
BO	7.72868	7.823851	2.02	0.043	1.062728	56.20676
M	6.162043	6.890638	1.63	0.104	.6884586	55.15332
U	6.547095	5.306048	2.32	0.020	1.337206	32.05524
WB	4.231173	3.552244	1.72	0.086	.8162852	21.93207
WO	3.399138	2.703933	1.54	0.124	.7149213	16.16141
WP	2.12e-06	.0091325	-0.00	0.998	0	.
age_cat						
20-24	.9444105	1.201994	-0.04	0.964	.0779468	11.44257
25-29	.8079029	1.015	-0.17	0.865	.0688591	9.478885
30-34	.3067858	.3842996	-0.94	0.346	.026337	3.573591
greater than 34	.2556753	.3218523	-1.08	0.279	.0216855	3.014454
0.parity	1.016398	.3258644	0.05	0.960	.5422039	1.905307
imd_3_score						
most deprived	.4342183	.2256039	-1.61	0.108	.1568393	1.202157
3rd and 4th deciles	.5644775	.2685964	-1.20	0.229	.2221354	1.434417
5th and 6th deciles	.6803869	.3456319	-0.76	0.448	.251392	1.841452
1.any_risk	2.112226	.7313112	2.16	0.031	1.071595	4.163417
2.high_risk_num	1.848433	1.227859	0.92	0.355	.5027771	6.795662
place_hosp_comm						
hospital	.6361429	.2928467	-0.98	0.326	.2580514	1.568206
imp_gstt						
imp	10.41672	5.60486	4.36	0.000	3.628525	29.90417
_cons	.0143837	.0240619	-2.54	0.011	.0005419	.3817767
3						
mod_care_4						
standard	2.029736	2.288317	0.63	0.530	.22274	18.49613
partial CoC	2.438196	2.827234	0.77	0.442	.2512174	23.66398
ethnicity						
BA	2.626047	2.073478	1.22	0.221	.5587396	12.34229
BC	1.201955	1.499941	0.15	0.883	.1041498	13.87132
BO	1.032836	1.324027	0.03	0.980	.0837234	12.74135
M	1.53e-06	.0016317	-0.01	0.990	0	.
U	.5783578	.5561831	-0.57	0.569	.0878276	3.808574
WB	1.778301	1.417697	0.72	0.470	.3727437	8.483993
WO	1.196887	.8684031	0.25	0.804	.2887104	4.96185
WP	1.60e-06	.0100035	-0.00	0.998	0	.
age_cat						
20-24	946768.2	2.68e+09	0.00	0.996	0	.
25-29	1081410	3.07e+09	0.00	0.996	0	.
30-34	1760311	4.99e+09	0.01	0.996	0	.
greater than 34	472766.8	1.34e+09	0.00	0.996	0	.
0.parity	.7531945	.3418027	-0.62	0.532	.309477	1.833098
imd_3_score						
most deprived	1.474234	1.08819	0.53	0.599	.3469455	6.264289
3rd and 4th deciles	.8275671	.6216565	-0.25	0.801	.1898423	3.60756
5th and 6th deciles	1.433326	1.086795	0.47	0.635	.3242916	6.335112
1.any_risk	2.895147	1.356331	2.27	0.023	1.155836	7.251788
2.high_risk_num	1.272324	1.592764	0.19	0.847	.1094001	14.79715
place_hosp_comm						
hospital	1.023997	.5942124	0.04	0.967	.3283651	3.193303
imp_gstt						
imp	9.324685	7.681576	2.71	0.007	1.85534	46.8646
_cons	2.80e-09	7.93e-06	-0.01	0.994	0	.
4						
mod_care_4						
standard	.2309158	.1969309	-1.72	0.086	.0434038	1.228514
partial CoC	.4939632	.4295315	-0.81	0.417	.0898509	2.715607
ethnicity						
BA	.6189749	.6022693	-0.49	0.622	.0919274	4.167747
BC	.3402144	.4352897	-0.84	0.399	.0277126	4.176653
BO	7.92e-07	.0011195	-0.01	0.992	0	.
M	4.08e-07	.0004059	-0.01	0.988	0	.
U	.7025714	.5593681	-0.44	0.657	.1475663	3.344983
WB	.373482	.3541876	-1.04	0.299	.0582159	2.396058
WO	.2917438	.2447006	-1.47	0.142	.0563709	1.509901
WP	3.24e-06	.0258196	-0.00	0.999	0	.
age_cat						
20-24	.1451364	.2109871	-1.33	0.184	.0084018	2.507158
25-29	.0522817	.0791982	-1.95	0.051	.0026848	1.018076
30-34	.040073	.059397	-2.17	0.030	.0021937	.7320142
greater than 34	.0252698	.0376016	-2.47	0.013	.0013678	.4668686
0.parity	.2194832	.1566993	-2.12	0.034	.0541613	.8894338
imd_3_score						
most deprived	1.402094	1.292939	0.37	0.714	.230061	8.544987
3rd and 4th deciles	.6948283	.6501306	-0.39	0.697	.1110281	4.348325
5th and 6th deciles	.8176601	.901942	-0.18	0.855	.0941092	7.104175
1.any_risk	1.932104	1.203268	1.06	0.290	.5700627	6.548446
2.high_risk_num	2.565955	2.56353	0.94	0.346	.3621195	18.18219
place_hosp_comm						
hospital	.3593408	.2686003	-1.37	0.171	.0830328	1.555118
imp_gstt						
imp	7.903945	7.665181	2.13	0.033	1.181288	52.88495
_cons	1.920439	3.991262	0.31	0.754	.0326846	112.8388

Table 13: Number of missed appointments in relation to place of care

miss_appt_4_cat	RRR	Std. Err.	z	P>|z|	[95% Conf.	Interval]
0	(base outcome)					
1						
place_hosp_comm						
hospital	.9569555	.2576891	-0.16	0.870	.564521	1.622196
ethnicity						
BA	2.517118	1.14186	2.03	0.042	1.034586	6.124078
BC	1.946001	1.061045	1.22	0.222	.6683913	5.665725
BO	.8344806	.7122503	-0.21	0.832	.1566369	4.445681
M	.7755741	.6686059	-0.29	0.768	.1431597	4.201709
U	.9020032	.397027	-0.23	0.815	.3806642	2.137342
WB	.9135987	.4062334	-0.20	0.839	.3821793	2.183956
WO	1.456576	.5637853	0.97	0.331	.6821274	3.110291
WP	1.19e-06	.0036291	-0.00	0.996	0	.
age_cat						
20-24	.287718	.2572229	-1.39	0.163	.0498866	1.659396
25-29	.202999	.1727676	-1.87	0.061	.0382875	1.076294
30-34	.2834254	.2324265	-1.54	0.124	.0568068	1.41409
greater than 34	.2015995	.1670383	-1.93	0.053	.0397387	1.02274
1.parity	1.333352	.3112723	1.23	0.218	.8437839	2.106972
imd_3_score						
3rd and 4th deciles	.425283	.1189227	-3.06	0.002	.2458414	.7357007
5th and 6th deciles	.7094975	.2181208	-1.12	0.264	.3883882	1.296092
least deprived	.5615893	.2044222	-1.59	0.113	.275154	1.146204
1.any_risk	1.376409	.3679088	1.20	0.232	.8151256	2.324183
2.high_risk_num	.5711377	.2460416	-1.30	0.194	.2454991	1.328715
mod_care_4						
standard	1.473125	.6518644	0.88	0.381	.6188412	3.506712
partial CoC	1.979531	.9058473	1.49	0.136	.8073215	4.853758
imp_gstt						
imp	1.736336	.4981058	1.92	0.054	.9895738	3.046629
_cons	.4301065	.4174436	-0.87	0.385	.0641852	2.882153
2						
place_hosp_comm						
hospital	.6444555	.296171	-0.96	0.339	.2618232	1.586272
ethnicity						
BA	11.49696	9.698098	2.90	0.004	2.200715	60.06232
BC	3.370232	3.627379	1.13	0.259	.4088011	27.78481
BO	7.69689	7.790642	2.02	0.044	1.058634	55.96093
M	6.204943	6.936276	1.63	0.102	.6937629	55.49636
U	6.504876	5.271353	2.31	0.021	1.328774	31.84394
WB	4.261376	3.577301	1.73	0.084	.8222254	22.08558
WO	3.390012	2.696209	1.53	0.125	.7131934	16.1137
WP	2.10e-06	.0090408	-0.00	0.998	0	.
age_cat						
20-24	.9640915	1.227325	-0.03	0.977	.0795255	11.68773
25-29	.8111337	1.018517	-0.17	0.868	.069225	9.504333
30-34	.3079009	.3854809	-0.94	0.347	.026469	3.581664
greater than 34	.2579331	.3245363	-1.08	0.281	.0219033	3.037422
1.parity	.9754309	.3129515	-0.08	0.938	.5201182	1.829325
imd_3_score						
3rd and 4th deciles	1.306187	.5227048	0.67	0.504	.5961767	2.861777
5th and 6th deciles	1.578365	.7246758	0.99	0.320	.6417928	3.881684
least deprived	2.29793	1.193699	1.60	0.109	.830167	6.360747
1.any_risk	2.099106	.7269789	2.14	0.032	1.06473	4.138368
2.high_risk_num	1.91287	1.273571	0.97	0.330	.5187563	7.053547
mod_care_4						
standard	1.829594	1.142745	0.97	0.333	.5379022	6.22309
partial CoC	.8694436	.5991082	-0.20	0.839	.22527	3.355672
imp_gstt						
imp	10.31898	5.539971	4.35	0.000	3.602889	29.55446
_cons	.0062786	.0099685	-3.19	0.001	.0002795	.1410296
3						
place_hosp_comm						
hospital	1.027215	.5956154	0.05	0.963	.3296895	3.200501
ethnicity						
BA	2.648138	2.091771	1.23	0.218	.5630855	12.45394
BC	1.198983	1.495906	0.15	0.884	.1039478	13.82964
BO	1.033182	1.324002	0.03	0.980	.0838262	12.73427
M	1.54e-06	.0016388	-0.01	0.990	0	.
U	.5769185	.5547314	-0.57	0.567	.0876291	3.798224
WB	1.78182	1.420415	0.72	0.469	.3735172	8.499963
WO	1.194865	.866795	0.25	0.806	.2882896	4.952322
WP	1.59e-06	.0099383	-0.00	0.998	0	.
age_cat						
20-24	941966.5	2.65e+09	0.00	0.996	0	.
25-29	1064254	2.99e+09	0.00	0.996	0	.
30-34	1731111	4.87e+09	0.01	0.996	0	.
greater than 34	466779.2	1.31e+09	0.00	0.996	0	.
1.parity	1.320394	.599513	0.61	0.540	.5422793	3.215022
imd_3_score						
3rd and 4th deciles	.563164	.3030157	-1.07	0.286	.1961723	1.616709
5th and 6th deciles	.976508	.5736932	-0.04	0.968	.3087447	3.088532
least deprived	.6780669	.500478	-0.53	0.599	.1595896	2.880981
1.any_risk	2.886719	1.35232	2.26	0.024	1.15252	7.230371
2.high_risk_num	1.296785	1.62442	0.21	0.836	.1113293	15.10521
mod_care_4						
standard	2.037241	2.296359	0.63	0.528	.2236537	18.55704
partial CoC	2.44362	2.832502	0.77	0.441	.2519827	23.69718
imp_gstt						
imp	9.301962	7.659612	2.71	0.007	1.852085	46.71844
_cons	3.15e-09	8.84e-06	-0.01	0.994	0	.
4						
place_hosp_comm						
hospital	.3605907	.2694494	-1.37	0.172	.0833602	1.559804
ethnicity						
BA	.6230185	.6064412	-0.49	0.627	.0924588	4.198108
BC	.3400471	.4350152	-0.84	0.399	.0277086	4.173147
BO	7.95e-07	.0011212	-0.01	0.992	0	.
M	4.11e-07	.0004077	-0.01	0.988	0	.
U	.70192	.5587515	-0.44	0.657	.1474698	3.340967
WB	.3747134	.3553621	-1.04	0.301	.0584059	2.404042
WO	.2916114	.244571	-1.47	0.142	.0563523	1.509027
WP	3.22e-06	.0256849	-0.00	0.999	0	.
age_cat						
20-24	.1464599	.2129082	-1.32	0.186	.0084787	2.52992
25-29	.052439	.0794202	-1.95	0.052	.0026946	1.020519
30-34	.0401708	.0595274	-2.17	0.030	.0022007	.733278
greater than 34	.025392	.0377773	-2.47	0.014	.001375	.4689034
1.parity	4.534777	3.238673	2.12	0.034	1.11851	18.38535
imd_3_score						
3rd and 4th deciles	.496867	.3204713	-1.08	0.278	.1403553	1.758942
5th and 6th deciles	.5848716	.510162	-0.61	0.539	.1058251	3.232454
least deprived	.7131029	.6575218	-0.37	0.714	.1170295	4.345191
1.any_risk	1.928033	1.200607	1.05	0.292	.5689343	6.533816
2.high_risk_num	2.581789	2.580332	0.95	0.343	.3640824	18.30804
mod_care_4						
standard	.2315544	.197482	-1.72	0.086	.0435214	1.231979
partial CoC	.494682	.4300472	-0.81	0.418	.0900206	2.718381
imp_gstt						
imp	7.884151	7.64327	2.13	0.033	1.179125	52.71693
_cons	.5883176	1.026983	-0.30	0.761	.019219	18.00909

Testing using qualitative data: Firstly we explored why women receiving care in the hospital-based model might have less appointments with a known healthcare professional than those in the community-based model. One explanation for this appeared to be that women in the hospital-based model were referred to the specialist model after disclosing social risk factors during their pregnancy. This would have an impact on the number of appointments they had with a known healthcare professional depending on their gestation at the time of referral to the specialist model. Although it was often seen as a positive referral, only women in the hospital-based model described care being disrupted through a referral to the specialist model. Flexibility was discussed though women's ability to rebook missed appointments easily and without reproach. Women in both models described feeling comfortable when rebooking appointments. This ability to reschedule may reduce the number of 'missed' appointments recorded, seen as a mitigating effect of the specialist model.

Table 14: Qualitative quotations relating to women's experiences of continuity of care, missing and rescheduling appointments

'I met all of them when I was going for my appointments at [Community centre]. I met all of them before I gave birth…and when I gave birth all of them came to see me at home one by one…. they was taking care of me. Every time when I'm at home I call them, they pick up my call, they know everything about me. Yeah but not the midwife at hospital.' (CBM7)

'There are six of them [CBM midwives]… I met all of them, when I have the baby there going to be one of them… in the hospital, which is I never heard this. Which is good, they know you and then they know all the, the problem or the issues you have' (CBM2)

'sometimes if I was getting anxious and worried, um, I felt like I didn't want to bother or inconvenience [named midwife] even though I had her mobile number and I think in the beginning I did try to utilise it but it, she wasn't either at work or someone else would pick up from the team and say, 'Oh she's not here.' And so that would kind of knock my confidence, so I wouldn't kind of ring her. I just get told that they're rushed off their feet, you have one midwife to six, seven ladies when they're all in active labour, so I don't, I wouldn't think that they would be there for 24 hours, I think the kind of contact is very minimal. the midwife kind of works like nine to five. And I know that she, um, isn't in some days, so any type of concerns like that I'll just rather ring the triage' (HBM8)

'because she [HBM midwife] says she will be off…so I didn't want to call another midwife and explain everything, so I just went straight… to the GP because I was feeling bad, yeah, the GP, yeah she say that I had to go back to the

hospital… in the Emergency they say because I was five days after the operation they cannot see me, so they send me to the labour ward. (HBM10)

'That is something that does worry me a little bit is like who's going to come [to provide care at homebirth]?...obviously you don't know when the baby's going to come so you don't know a hundred percent who's going to come round to help you. So that's a bit … daunting. (HBM2)

'she [midwife] cancelled a lot of my appointments….I understand that people have babies. but it meant that it was cancelled appointments again and again and again… I only had maybe one or two midwife appointments during my pregnancy…. it suffered me because I didn't go to antenatal classes, because she didn't really sit there and go, 'Well this is what's available'…. so because of me not having the right sort of preparation for the baby it kind of led to bigger things being involved because they [social care] were scared that I wasn't picking up cues and I wasn't doing this, and I wasn't doing that… It was an incredibly stressful time for me… And when I sat there and said, 'Look I didn't have any antenatal care,' they were a little bit more understanding of why I wasn't picking up on those cues'(HBM1)

'because of my situation I called them to change it…. if it's not a convenient time, I'm happy to change it.' (CMB1)

'because they say you have to come, I tried to squeeze my plan, to be attending as much as I can…just now because of my situation I called them to change it…. if it's not a convenient time, I'm happy to change it.' (CMB1)

'I like her [CBM midwife], I feel open to talk all to her, yeah. Because the first appointment I missed so she said, 'OK I can, if you are free on Saturday I come on Saturday,' And she came to see me... at home.' (CBM7)

'I think there was one week where, because I was going out of London back to [region].. I was like, 'I can't do this appointment, could we do it a week early?' And they did and it was fine. Um, which was obviously really helpful' (CBM9)

She's perfect when it comes to, um, appointments or anything like that, and if I'm running late or something comes up I just text her or give her a call and she'll muddle things around (HBM9)

'she [midwife] cancelled a lot of my appointments….I understand that people have babies. but it meant that it was cancelled appointments again and again and again… I only had maybe one or two midwife appointments during my pregnancy…. it suffered me because I didn't go to antenatal classes, because she didn't really sit there and go, 'Well this

is what's available'…. so because of me not having the right sort of preparation for the baby it kind of led to bigger things being involved because they [social care] were scared that I wasn't picking up cues and I wasn't doing this, and I wasn't doing that… It was an incredibly stressful time for me… And when I sat there and said, 'Look I didn't have any antenatal care,' they were a little bit more understanding of why I wasn't picking up on those cues…'(HBM1)

The data confirmed aspects of both the initial programme theories and the rival theories. Therefore the refined theory aims to reflect these conflicting ideas:

Refined theory: If a small team of midwives provide continued supportive presence throughout pregnancy and the perinatal period and prepare women for labour and birth through education and gaining familiarity of birth settings, then they will feel better supported and able to seek help and exercise choice, resulting in improved safety and reduced feelings of anxiety. When a named midwife is unable to be present for labour care, women with social risk factors should be cared for by a healthcare professional who is aware of their circumstances and individual needs to avoid disrespectful care and the risk of triggering previous experiences of trauma.

Women's insights around missed appointments confirmed the initial programme theory and enabled the development of a new programme theory relating to how midwives in specialist models share workloads.

Refined theory: If women are able to reschedule appointments easily, and do not fear judgement or reproach when they miss appointments then they will perceive the maternity environment as a place of safety and their engagement with flexible services will improve.

Refined theory: If women have the opportunity to get to know the all midwives in a small team throughout their pregnancy, then they will not feel disappointment or let down when their named midwife is unable to attend an appointment, and care, information and responsibility will be shared across the team, thus improving safety.
