# Supplementary material for: Project20: maternity care mechanisms that improve access and engagement for women with social risk factors in the UK – a mixed-methods, realist evaluation
Source: BMJ Open. 2023 Feb 7;13(2):e064291. doi: 10.1136/bmjopen-2022-064291 (PMC9906302; doi:10.1136/bmjopen-2022-064291)
Supplement: Author's manuscript [file bmjopen-2022-064291.draft_revisions.pdf]

BMJ Open

BMJ Open is committed to open peer review. As part of this commitment we make the peer review history of every article we publish publicly available.

When an article is published we post the peer reviewers' comments and the authors' responses online. We also post the versions of the paper that were used during peer review. These are the versions that the peer review comments apply to.

The versions of the paper that follow are the versions that were submitted during the peer review process. They are not the versions of record or the final published versions. They should not be cited or distributed as the published version of this manuscript.

BMJ Open is an open access journal and the full, final, typeset and author-corrected version of record of the manuscript is available on our site with no access controls, subscription charges or pay-per-view fees (http://bmjopen.bmj.com).

If you have any questions on BMJ Open's open peer review process please email info.bmjopen@bmj.com

BMJ Open

Project20: Maternity care mechanisms that improve access and engagement for women with social risk factors. A realist evaluation

Journal:	*BMJ Open*
Manuscript ID	bmjopen-2022-064291
Article Type:	Original research
Date Submitted by the Author:	27-Apr-2022
Complete List of Authors:	Rayment-Jones, Hannah; King's College London, Women and Children's Health<br>Dalrymple, Kathryn; King's College London, Women and Children's Health<br>Harris, James; University College London Hospitals NHS Foundation Trust, Centre for Nursing, Midwifery and Allied Health Professional Research<br>Harden, Angela; City University of London<br>Parslow, Elidh; North Middlesex University Hospital NHS Trust<br>Georgi, Thomas; King's College London<br>Sandall, Jane; King's College London, Health and Social Care Research Division
Keywords:	Organisation of health services < HEALTH SERVICES ADMINISTRATION & MANAGEMENT, Maternal medicine < OBSTETRICS, Community child health < PAEDIATRICS, PRIMARY CARE, PUBLIC HEALTH, SOCIAL MEDICINE

SCHOLARONE™
Manuscripts

I, the Submitting Author has the right to grant and does grant on behalf of all authors of the Work (as defined in the below author licence), an exclusive licence and/or a non-exclusive licence for contributions from authors who are: i) UK Crown employees; ii) where BMJ has agreed a CC-BY licence shall apply, and/or iii) in accordance with the terms applicable for US Federal Government officers or employees acting as part of their official duties; on a worldwide, perpetual, irrevocable, royalty-free basis to BMJ Publishing Group Ltd ("BMJ") its licensees and where the relevant Journal is co-owned by BMJ to the co-owners of the Journal, to publish the Work in this journal and any other BMJ products and to exploit all rights, as set out in our licence.

The Submitting Author accepts and understands that any supply made under these terms is made by BMJ to the Submitting Author unless you are acting as an employee on behalf of your employer or a postgraduate student of an affiliated institution which is paying any applicable article publishing charge ("APC") for Open Access articles. Where the Submitting Author wishes to make the Work available on an Open Access basis (and intends to pay the relevant APC), the terms of reuse of such Open Access shall be governed by a Creative Commons licence – details of these licences and which Creative Commons licence will apply to this Work are set out in our licence referred to above.

Other than as permitted in any relevant BMJ Author's Self Archiving Policies, I confirm this Work has not been accepted for publication elsewhere, is not being considered for publication elsewhere and does not duplicate material already published. I confirm all authors consent to publication of this Work and authorise the granting of this licence.

For peer review only

Project20: Maternity care mechanisms that improve access and engagement for women with social risk factors. A realist evaluation

Corresponding author:
Hannah Rayment-Jones RM, PhD, PGCE, FHEA
NIHR Post-Doctoral Research Fellow
Department of Women and Children's Health
Faculty of Life Sciences & Medicine, King's College London
10th Floor, North Wing St. Thomas' Hospital
Westminster Bridge Road, London, SE1 7EH
Hannah.rayment-jones@kcl.ac.uk

Kathryn Dalrymple PhD
Post-Doctoral Researcher
Department of Women and Children's Health
Faculty of Life Sciences & Medicine, King's College London
10th Floor, North Wing St. Thomas' Hospital
Westminster Bridge Road, London, SE1 7EH
kathryn.dalrymple@kcl.ac.uk

James Harris, RM, PhD, BSc, PGCE, FHEA
Senior Clinical Lecturer for Evidence Based Midwifery
Chelsea and Westminster NHS Foundation Trust
Clinical Research Facility, St Stephen's Centre
252 Fulham Road, London, SW109NA

For peer review only

james.harris4@nhs.net

Angela Harden
Professor of Health Sciences
School of Health Sciences
City, University of London
Northampton Square
London EC1V 0HB
angela.harden@city.ac.uk

Elidh Parslow
Birth Centre Matron
Imperial College NHS Trust
Birth Centre, St Mary's Hospital,
London W2 1NY
Elidh.parslow@nhs.net

Thomas Georgi, MBBS, BA
School of Population Health & Environmental Sciences
Faculty of Life Sciences & Medicine
King's College London
London, SE1 7EH
Tom.Georgi@nhs.net

Jane Sandall CBE PhD MSc BSc RM HV RN
Professor of Social Science and Women's Health
Department of Women and Children's Health
Faculty of Life Sciences & Medicine, King's College London
10th Floor, North Wing St. Thomas' Hospital
Westminster Bridge Road, London, SE1 7EH
jane.sandall@kcl.ac.uk

For peer review only

Abstract

Objectives: To evaluate how women access and engage with different models of maternity care, whether specialist models improve access and engagement for women with social risk factors, and if so, how?

Design: Realist evaluation

Setting: Two UK service providers

Population: Women accessing maternity care in 2019 (n=1020)

Methods: Prospective observational cohort with multinomial regression analysis to compare measures of access and engagement between models and place of antenatal care. Realist informed, longitudinal interviews with women accessing specialist models of care were analysed to identify mechanisms.

Main outcome measures: Measures of access and engagement, healthcare-seeking experiences.

Results: The number of social risk factors women were experiencing increased with deprivation score, with the most deprived more likely to receive a specialist model that provided continuity of care. Women attending hospital-based antenatal care were more likely to access maternity care late (RR 2.51 CI1.33-4.70), less likely to have the recommended number of antenatal appointments (RR0.61 CI0.38-0.99) and more likely to have over 15 appointments (RR4.90 CI2.50-9.61) compared to community-based care. Women accessing standard care (RR0.02, CI0.00-0.11) and Black women (RR0.02, CI0.00-0.11) were less likely to have appointments with a known healthcare professional compared to the specialist model. Qualitative data revealed mechanisms for improved access and engagement including self-referral, relational continuity with a small team of midwives, flexibility, and situating services within deprived community settings.

Conclusion: Inequalities in access and engagement with maternity care appears to have been mitigated by the community-based specialist model that provided continuity of care. The findings enabled the

For peer review only - http://bmjopen.bmj.com/site/about/guidelines.xhtml

refinement of a realist programme theory to inform those developing maternity services in line with current policy.

Keywords: Inequality, maternity care, continuity of care, access and engagement, social risk factors

Keywords: Inequality, maternity care, continuity of care, access and engagement, social risk factors

Strengths and limitations of this study

- The specialist models of care evaluated in this study are situated in the UK's complex maternity system and can be appropriately investigated using a realist evaluation design.
- Mixed methods are used to understand not only if access and engagement are improved by specialist models of care, but how and in what context.
- Women interviewed during the study may have perceived the study questions to be testing them about their willingness to engage with their care, although this may have been lessened through the trust built between the participant and the researcher over the course of the longitudinal interviews.
- The relatively small numbers in each quantitative data group should be taken into consideration due to the significant amount of multiple testing required to establish the separate effects of the model of care, place of care and service attended. This presents a potential limitation as the use of multiple testing can result in a substantial change in statistical power, reducing the probability of detecting effects when they do exist.
- The generalisability of the findings is limited by the urban location of both specialist models of care evaluated. This is particularly significant when reflecting on the outcomes relating to place-based care- what may have yielded significant outcomes in a densely populated, multicultural community, may yield very different results elsewhere. Research is needed to test the generalisability of the findings to rural and other community settings.

For peer review only

Introduction

As a core component of maternity care across the globe, the adequacy of antenatal care is measured by the timing of antenatal care access and the number of appointments attended [1]. The routine functions of antenatal care include support, health promotion, screening and diagnosis, disease prevention, and additional care for women at higher risk [2]. When these functions are of high quality and care is well attended, it makes a crucially important contribution to the reduction of health inequalities [3,4]. The World Health Organisation (WHO) [1] recommend a minimum of eight antenatal appointments to reduce perinatal mortality and improve women's experiences. In high-income countries such as the UK, antenatal care coverage is consistently high and correlates with relatively low maternal and infant mortality rates when compared to the LMIC's [5]. Despite this, there are marked inequalities in access, health outcomes and women's experiences with poor antenatal care uptake for women from lower socioeconomic and minority groups [6–10] . The most deprived women in the United Kingdom are 60% less likely to have received any antenatal care when compared to the least deprived women [6].

Factors associated with inequalities in maternal and neonatal outcomes include; Black and minority ethnicity, poverty, young motherhood, homelessness, difficulty speaking or understanding English, migrant or refugee status, domestic violence, mental illness and substance abuse [7,11–13]. It is hypothesised that a lack of access and engagement with maternity services is directly linked to health inequalities for women with these social risk factors [14–17]. Therefore policies to tackle inequalities often focus on improving access to maternity services and continuity of midwifery care [18 19]. There is a strong evidence base documenting improved maternity outcomes and experiences for women who receive continuity models of midwifery care [20] but less is known about what works to improve access and engagement for women with social risk factors [21]. The impact of place-based maternity care is also poorly understood. The specialist models of care being evaluated in this study provide continuity of care across the antenatal, intrapartum and postnatal periods from a one-to-one midwife or small team of midwives whom women have had the opportunity to meet during pregnancy. One model based is in the community setting and provides care to women in the local, deprived catchment area, the other is based in a large teaching hospital and women with significant social risk factors are referred to the model of care early in pregnancy. See Appendix A for full definitions of each type of maternity care women may experience, and those being compared in this study. As part of a wider evaluation these two UK-based

For peer review only - http://bmjopen.bmj.com/site/about/guidelines.xhtml

specialist models, this study aimed to describe and compare service use for women with and without social risk factors by asking:

1) Are women with social risk factors more likely to be offered the specialist models of care over those without? If so, do they find this acceptable?

2) Compared to standard maternity care and group practice models, do specialist models improve access and engagement with maternity services? If so, for whom, in what context, and how?

3) Do specialist models of care increase the quality of relational continuity and reduce the number of appointments women miss?

4) How does the location of a model of care- community or hospital, impact on women's access and engagement with maternity services?

Methods

Study design

Realist methodology is a theoretically informed approach to evaluating complex interventions and often uses mixed methods to understand *how* interventions, in this case models of maternity care, do or do not work in different contexts [22]. This allows those implementing services such as specialist models of care to refine, scale-up, or even withdraw the service [23]. The aims of this study were approached through the testing and refinement of initial programme theories constructed in an earlier synthesis of literature [24] and focus groups with midwives[25] relating to how women with social risk factors access and engage with maternity care.

Routinely collected pregnancy and birth outcome data for 1000 women accessing different maternity models, including standard care, group practice and specialist models of care at two large, inner-city maternity services were prospectively collected and analysed. Longitudinal interviews with 20 women with social risk factors were conducted to uncover the mechanisms for any associations found in the

For peer review only - http://bmjopen.bmj.com/site/about/guidelines.xhtml

For peer review only

cohort analysis. Appendix A provides definitions of the two service provider settings and describes the different models of care women might experience at each.

The research was approved by the London Brent Research Ethics Committee (HRA) REC Reference 18-LO-0701. All participants provided written consent by signing a consent form approved by the ethics committee. The quantitative data collected was anonymised before being made available to the research team. A service user panel contributed to the study through external peer review for funding, the research focus, design, and analysis of data.

Patient and Public Involvement

Multiple representative, diverse groups of service users were involved in the planning and development of this research. They provided feedback on the protocol, study materials, interview guides, and refinement of programme theories. They also prioritised outcome measures and reviewed the qualitative data analysis.

Data collection

Quantitative data collection

A power calculation was based on a previous analysis of UK antenatal care utilisation [6] and validated metrics for monitoring local inequalities in access to care at a service evaluated in this research [26]. We calculated that with 250 women in each group (those receiving standard maternity care and those receiving group practice or a specialist model of care), we would have 90% power to detect a 15% difference in timely access to antenatal care (before 12+6 weeks gestation) between the different models of care with 500 anonymised birth records accessed at each trust. Pregnancy and birth outcome data were extracted from computerised records at each service provider. Data collection was prospective; the demographics of the first 500 women booking for maternity care with each service provider in January 2019 were collected, with pregnancy and birth outcomes collected later in the year when all women had been discharged from maternity care. Deprivation deciles, calculated using the 2019 English Indices of Deprivation [27] were grouped into four groups of sufficient numbers to enable comparisons between groups of similar numbers.

For peer review only - http://bmjopen.bmj.com/site/about/guidelines.xhtml

Semi-structured, longitudinal interviews with 20 women with low socioeconomic status and social risk factors who were receiving specialist care from one of the two service providers were carried out throughout the pregnancy and postnatal period. Women were identified by the specialist model midwives providing their care if they met the following inclusion criteria: A deprivation score [28] of higher than 30 and/or secondary school as the highest level of education attained. An interview guide was developed using previous literature [24,25,29] to elicit specific mechanisms explaining how the specialist model might improve access and engagement.

Analysis

The quantitative data were analysed using Stata 16.0. Firstly, women's social risk factors, ethnicity, socioeconomic status and medical characteristics were described using descriptive statistics and stratified by the service provider attended to enable comparisons of differences in the samples between each service. Variables were tested for bivariate association using chi-square tests and *t*-tests, for dichotomous and continuous variables, respectively. Chi-square analyses were also performed to test for associations between socio-economic position by deprivation (IMD) decile [30], as well as social and medical risk factors. Secondly, three regression models were developed: Model 1 adjusted for ethnicity, age, parity, deprivation score, social risk factors and medical risk status, Model 2 included model 1, plus adjustment for the service provider that women attended to consider differences in organisation guidelines, processes and culture and Model 3 included model 2, however, the place of antenatal care (hospital versus community-based antenatal care) was treated as the independent variable. This structured model allowed us to explore the association between access and engagement depending on the model of care received, whilst accounting for interactions between independent variables to predict the dependent variable. Risk ratios and confidence intervals are used to demonstrate statistical significance as well as the direction and strength of the effect [31]. Definitions for all demographics and social risk factors are given in Appendix A.

The qualitative data were coded using NVivo v.12 and analysed using a thematic framework analysis [32]. This allowed for the organisation of a large qualitative dataset into a coding framework developed using previously constructed programme theories [24,25], to uncover new theories and differences in women's experiences depending on their characteristics [32]. We utilised existing models of data adequacy [33] to assess acceptable data quality. The initial programme theories relating to access and engagement were

For peer review only - http://bmjopen.bmj.com/site/about/guidelines.xhtml

refined and constructed as a Context + Mechanism= Outcome (CMO) configurations that present specific aspects of the specialist models of care.

Results

Full pregnancy and birth data were collected and analysed from 799 women accessing care across the two service providers. Two hundred and one sets of birth outcome data were missing due to sample drop out, that is that those women stopped receiving care at the service and were therefore excluded from the final analysis. . The total number and reasons given for women with missing outcome data at each hospital did not differ significantly. See Figure 1 for the data collection flowchart.

Demographics of women included in the quantitative data analysis

Table 1 presents the demographic profiles of the 799 women who continued their pregnancies and gave birth at the two services, with significant differences in ethnicity, social and medical risk factors, and model of care received highlighted and adjusted for in the analysis of access and engagement outcomes.

Table 1: Women's demographics at each service provider

**Demographic variable**	**Service A n(%) Total data = 405**	**Service B n(%) Total data= 394**	**TOTAL n(%) Total data=799**	**X^2 p value**
**Ethnicity**				**Pr 0.000**
Asian	37(9)	53(13)	90(11)	
Black African	31(8)	46(12)	77(10)	
Black Caribbean	23(6)	16(4)	39(5)	
Black other	8(2)	14(4)	22(3)	
Mixed	12(3)	7(2)	19(2)	
White British	98(24)	58(15)	156(20)	
White other	80(20)	139(36)	219(27)	
Unknown	116(29)	61(15)	177(22)	
**Age**				Pr 0.356
≤20	6(1)	4(1)	10(1)	
21-24 years	19(5)	32(8)	51(6)	
25-29 years	63(16)	56(14)	119(15)	
30-34 years	134(33)	125(32)	259(32)	
≥35 years	183(45)	177(45)	360(45)	

For peer review only - http://bmjopen.bmj.com/site/about/guidelines.xhtml

**Parity**				Pr 0.167
Primiparous	212(52)	187(47)	399(50)	
Multiparous	193(48)	207(53)	400(50)	
**Social Risk Factor**				
Domestic abuse	23(6)	17(4)	40(5)	Pr 0.377
Common mental health	4(1)	34(9)	38(5)	**Pr 0.000**
Severe mental health	2(<1)	8(2)	10(1)	Pr 0.051
Non-English speaking	16(4)	48(13)	64(8)	**Pr 0.000**
Social care involvement	27(7)	29(7)	56(7)	Pr 0.701
Drug/alcohol abuse	1(<1)	10(3)	11(1)	**Pr 0.005**
Unsupported/single	1(<1)	11(3)	12(2)	**Pr 0.003**
Financial/housing	15(4)	31(8)	46(6)	**Pr 0.012**
Learning disability	6(2)	5(1)	11(1)	Pr 0.797
Sexual abuse/trafficked	4(2)	5(1)	9(1)	Pr 0.677
AS/Refugee	8(2)	7(2)	15(2)	Pr 0.836
FGM	0	11(3)	11(1)	**Pr 0.001**
No recourse to public funds	6(1)	0	6(1)	**Pr 0.015**
**No of social risk factors**				**Pr 0.003**
None	337(83)	279(70)	616(77)	
1	43(11)	61(15)	104(13)	
2	13(3)	26(7)	39(5)	
3	6(1)	15(4)	21(3)	
4	5(1)	9(2)	14(2)	
≥5	1(<1)	4(1)	5(1)	
**Name of model of care**				**Pr 0.000**
Standard Care	256(63)	213(54)	469(59)	
Group Practice	77(19)	144(37)	221(28)	
Specialist	59(15)	21(5)	80(10)	
**Private Care**	13(3)	16 (4)	29(4)	
**Place of model of antenatal care**				**Pr 0.000**
Standard model in hospital	100(25)	212(54)	312(39)	
Standard model in community	156(40)	1(0)	157(20)	
Group practice in community	40(10)	94(24)	134(17)	
Group practice in hospital	37(9)	50(13)	87(11)	
Specialist model in community	59(15)	2(1)	61(8)	
Specialist model in hospital	0	19(5)	19(2)	
Private Care	13(3)	16(4)	29(4)	
**By place of antenatal care only***				**Pr 0.000**
Hospital based	137(35)	281(74)	418(54)	
Community based	255(65)	97(26)	352(46)	

*Excludes private care

Characteristics of the women interviewed

Twenty pregnant women with low socio-economic status and/or educational attainment were recruited- See Table 2. All twenty women received a specialist model of care, eight were first time mothers and the other twelve had between one and eight children. For five of the multiparous women, this was their first pregnancy in the UK. Based on 2019 deprivation scores [27], 19 participants were in the 1st or 2nd most deprived deciles, with one in the 3rd and 4th decile group. Twelve participants were born outside of the UK, and nine did not speak English and required an interpreter. All participants were experiencing between one and seven social risk factors including common or severe mental health issues, domestic violence, drug/alcohol misuse, no support, single motherhood, financial and housing problems, learning disability, sexual abuse, trafficking, female genital mutilation, and no recourse to public funds. Five participants were seeking asylum, had refugee status, or had had an asylum claim refused and nine had social care involvement during their pregnancy.

Table 2: Demographics of women interviewed

Characteristic	Community based model (CBM) n=10	Hospital based model (HBM) n=10	TOTAL n(%) n=20
**Ethnicity and migration status**			
**Born outside the UK:**	7	5	12(60)
Asian	0	2	2(10)
Black African	3	0	3(15)
Black Caribbean	0	1	1(5)
White	4	2	6(30)
Asylum seeker/refugee*	2	3	5(25)
**Born inside the UK:**	3	5	8(40)
Asian British	1	1	2(10)
Black British	2	1	3(15)
White British	0	3	3(15)
**Age**			
18-24	0	3	3(13)
25-29	1	1	2(2)
30-34	5	5	10(50)
>34	4	1	5(25)
**Parity**			
Primiparous	5	3	8(40)

For peer review only - http://bmjopen.bmj.com/site/about/guidelines.xhtml

**IMD Decile (2019)**			
Most deprived 1st +2nd	9	10	19(95)
3rd and 4th	1	0	1(5)
Least deprived 5th-10th	0	0	0
**No of social risk factors**			
1	3	0	3(15)
2	0	2	2(10)
3	2	0	2(10)
4	1	1	2(10)
≥5	4	7	11(55)
**Mental Illness**			
Common	5	9	14(70)
Severe	1	1	2(15)
**Level of education**			
Secondary school only	5	6	11(55)
Completed college	4	3	7(35)
Completed university	1	1	2(15)
**Occupation Status (NS- SEC)**			
8 (long term unemployed)	6	8	14(70)
7 (routine occupations)	0	2	2(15)
6-3 (semi-routine)	4	0	4(20)
**High medical risk at booking**	7	5	12(60)

*Including women whose asylum claim had been refused.

Model of care accessed by deprivation score and risk factors

When merging data for the two service providers Table 3 shows that women in the most deprived deciles were significantly more likely to receive a specialist model of care, and women in the least deprived deciles were less likely to receive community-based antenatal care than hospital-based antenatal care. This reflects the aim of the service providers to offer women in more deprived areas both specialist and/or community-based antenatal care. A statistically significant relationship was found between deprivation score and the number of social risk factors recorded.

Table 3: Maternity care received, and risk factors recorded by deprivation score

**Maternity care and risk factors**	**Least deprived (7th, 8th, 9th +10th)**	**5th and 6th deciles**	**3rd and 4th deciles**	**Most deprived (1st +2nd deciles)**	**Total n(%)**	**X^2 p value**

For peer review only - http://bmjopen.bmj.com/site/about/guidelines.xhtml

**Model of care**	**Total=149**	**Total=158**	**Total=286**	**Total=206**	**N=799**	**Pr 0.000**
Standard Care	81(54)	98(62)	163(57)	127(62)	469(58)	
Group Practice	43(29)	41(26)	85(30)	52(25)	221(28)	
Specialist	3(2)	16(10)	34(12)	27(13)	80(10)	
Private Care	22(15)	3(2)	4(1)	0(0)	29(4)	
**Place of antenatal care***	**Total=127**	**Total=155**	**Total=282**	**Total=206**	**Total=770**	**Pr0.008**
Hospital based	82(65)	91(59)	134(48)	111(54)	418(54)	
Community based	45(35)	64(41)	148(52)	95(46)	352(46)	
**Social Risk Factors**	**Total=149**	**Total=158**	**Total=286**	**Total=206**	**N=799**	**Pr 0.000**
None	133(89)	132(84)	212(74)	139(67)	616(77)	
One	10(7)	17(11)	50(17)	27(13)	104(13)	
Two	5(3)	4(3)	10(4)	20(10)	39(5)	
Three	1(1)	2(1)	10(4)	8(4)	21(3)	
Four or more	0	3(2)	4(1)	12(6)	19(2)	
**Medical Risk**						
High at booking	37(25)	46(29)	85(30)	56(27)	224(28)	Pr 0.743
High at onset of labour	62 (42)	79(50)	138(48)	96(47)	375(47)	Pr 0.475

*Excludes private care

Testing using qualitative data: Women reported different pathways into the specialist models including direct referrals from a GP, midwives, sexual health clinics, social workers, emergency departments, and self-referral. Overall women were pleased to be referred to the specialist model, however they were often not made aware of *why* they had been referred and described a lack of choice.

I don't know if it's because I have these issues, only, or if it's the team or if it's the hospital or, I don't know, or the combination. I have no idea. But I'm super-happy and feeling lucky I ended up like having all this. like how many people get the support we get here? Is it just me because I have mental health?' (CBM10)

Timing of access to maternity services

Quantitative Analysis 1- Model of care

For peer review only - http://bmjopen.bmj.com/site/about/guidelines.xhtml

The quantitative data was analysed to test the hypothesis that the specialist model of care has an impact on the timing of access to maternity care, known as the 'booking appointment'. Table 4 shows that no relationship was found between the model of care received and the gestation at which women attended their booking appointment. The base outcome was set for less than 10 weeks' gestation at the booking appointment to reflect the NICE guidance for women with complex social factors [15]. When adjusting for women's characteristics (see Appendix B for fully adjusted outcome data tables) primiparous women (RR 1.79 CI 1.02-3.12), those with social risk factors (RR 1.93 CI 1.03-3.62), and those with high medical risk status at the booking appointment (RR 2.49 CI 1.18-5.25) were more likely to book for maternity care later than 20 weeks.

Table 4: Gestation at booking appointment in relation to the model of care received

Gestation at booking	Model of Care	Number of women (%)	Unadjusted RR (95% CI)	Model 1 Adjusted RR (95% CI) *	Model 2 Adjusted RR (95% CI) **	Model 3 Adjusted RR (95% CI) ***
<10 weeks	Standard	197(62)	Ref	Ref	Ref	Ref
	Group	87(27)	Ref	Ref	Ref	Ref
	Specialist	35(11)	Ref	Ref	Ref	Ref
10-12	Standard	166(58)	1.01(0.59-1.73)	0.91 (0.52-1.61)	0.77(0.42-1.40)	0.76(0.42-1.38)
	Group	90(32)	1.24 (0.70-2.12)	1.18 (0.65-2.16)	1.09(0.59-2.02)	0.86(0.46-1.63)
	Specialist	29(30)	Ref	Ref	Ref	Ref
13-20	Standard	53(61)	0.94 (0.43-2.02)	1.14 (0.50-2.61)	0.88 (0.37-2.09)	0.89(0.37-2.13)
	Group	24(28)	0.96 (0.41-2.22)	1.14 (0.47-2.77)	1.04 (0.42-2.56)	0.75(0.29-1.94)
	Specialist	10(11)	Ref	Ref	Ref	Ref
> 20 weeks	Standard	53(68)	1.56 (0.62-3.92)	2.19 (0.82-5.80)	1.29(0.46-3.60)	1.27(0.45-3.56)
	Group	19(24)	1.27 (0.46-3.45)	1.75 (0.61-4.99)	1.29 (0.43-3.82)	1.15(0.38-3.44)
	Specialist	6(8)	Ref	Ref	Ref	Ref

* Model 1: Adjusted for demographics ethnicity, age, parity, IMD score, any social and medical risk factors at booking and onset of labour
** Model 2: Model 1 + Adjustment for place of antenatal care (community or hospital)
*** Model 3: Model 2 + Adjustment for service provider attended (A or B)

Quantitative Analysis 2- Place of antenatal care

Table 5 shows that after adjusting for the model of care received and service attended, women receiving hospital-based antenatal care were significantly more likely to attend their booking appointment after 20 weeks' gestation (RR 2.51 CI 1.33-4.70).

Table 5: Gestation at booking appointment in relation to the place of antenatal care[2]

Gestation at booking	Place of antenatal care	Number of women (%)	Unadjusted RR	Model 1 Adjusted RR (95% CI) *	Model 2 Adjusted RR (95% CI) **	Model 3 Adjusted RR (95% CI) ***
<10	Hospital	154(48)	Ref	Ref	Ref	Ref
	Community	165(52)	Ref	Ref	Ref	Ref
10-13weeks	Hospital	154(54)	1.25(0.91-1.73)	1.19(0.85-1.66)	1.30(0.91-1.86)	1.00(0.67-1.49)
	Community	131(46)	Ref	Ref	Ref	Ref
13-20	Hospital	54(62)	1.75(1.07-2.84)	1.59(0.95-2.66)	1.66(0.97-2.87)	1.05(0.54-2.01)
	Community	33(38)	Ref	Ref	Ref	Ref
> 20 weeks	Hospital	56(72)	**2.72(1.58-4.67)**	**2.89(1.65-5.10)**	**2.81(1.56-5.06)**	**2.51(1.33-4.70)**
	Community	22(28)	Ref	Ref	Ref	Ref

* Model 1: Adjusted for demographics ethnicity, age, parity, IMD score, social risk, and medical risk factors
** Model 2: Model 1 plus adjusted for model of care
*** Model 3: Model 2 plus Adjusted for service provider attended

Qualitative data analysis

The qualitative data was analysed to explore why timing of access to maternity care was different for women attending hospital-based antenatal care, primiparous women, and those with high medical risk status and social risk factors. Many women expressed wanting to be seen earlier in pregnancy and described not feeling valued by maternity services until their pregnancy is viable. Delays in accessing maternity services was often due to difficulties in accessing GP services and a convoluted referral pathway between community and hospital services. Community-based care and the ability to self-refer to maternity services appeared to reduce the time women spent waiting for their booking appointment.

'[at 3 weeks gestation] I called the GP to book an appointment. to say that I'm pregnant, and they sent me… to buy a Clearblue to check it because the list is too long. But it take a very long time, to see the GP… to refer me to the midwife, I was waiting a long time…when I saw the midwife the first time I was around 20 weeks. (CBM7)

Engagement - Number of antenatal appointments attended

Quantitative Analysis 1- Model of care

For peer review only

Women's engagement with services was tested through the number of antenatal appointments women attended. Table 6 shows no significant relationship between the model of care received and the number of antenatal appointments attended. Women with social risk factors were significantly more likely to have more than 15 antenatal appointments than those with no social risk factors (RR2.57 CI1.30-5.07), as were women with high medical risk (RR 2.70 CI 1.21-6.03)- See Appendix B.

Table 6:Number of antenatal appointments attended in relation to the model of care accessed

Number of antenatal appointments	Model of Care	Number of women (%)	Unadjusted RR	Model 1 Adjusted RR (95% CI) *	Model 2 Adjusted RR (95% CI) **	Model 3 Adjusted RR (95% CI) ***
1-6	Standard	173(63)	0.58 (0.31-1.09)	0.64 (0.35-1.30)	0.86 (0.44-1.69)	0.87(0.44-1.70)
	Group	63(23)	**0.41 (0.21-0.80)**	**0.42 (0.22-0.93)**	**0.43 (0.21-0.86)**	0.55(0.25-1.15)
	Specialist	37(14)	Ref	Ref	Ref	Ref
7-9****	Standard	135(61)	Ref	Ref	Ref	Ref
	Group	70(31)	Ref	Ref	Ref	Ref
	Specialist	17(8)	Ref	Ref	Ref	Ref
10-14	Standard	120(64)	0.94(0.45-1.95)	1.13(0.53-2.42)	0.80 (0.36-1.78)	0.82(0.37-1.81)
	Group	52(28)	0.78 (0.36-1.70)	0.90(0.40-2.02)	0.82 (0.36-1.86)	0.97(0.42-2.24)
	Specialist	16(8)	Ref	Ref	Ref	Ref
≥15	Standard	38(46)	0.47 (0.20-1.13)	0.68 (0.26-1.74)	0.43 (0.16-1.17)	0.43(0.16-1.14)
	Group	35(42)	0.85 (0.35-2.05)	1.25 (0.47-3.28)	1.02 (0.38-2.75)	1.28(0.47-3.45)
	Specialist	10(12)	Ref	Ref	Ref	Ref

* Model 1: Adjusted for demographics ethnicity, age, parity, IMD score, any social and medical risk factors at booking and onset of labour
** Model 2: Model 1 + Adjustment for place of antenatal care (community or hospital)
*** Model 3: Model 2 + Adjustment for service provider attended (A or B)
**** Set as base as WHO recommends 8 antenatal appointments [1]

Quantitative Analysis 2- Place of antenatal care

Table 7 shows that women receiving hospital-based care were significantly likely to have less than the recommended number of appointments, [1,2,15] (RR0.61 CI 0.38-0.99), and much more likely to have over 15 appointments (RR4.90 CI2.50-9.61) than those receiving community-based care regardless of their medical risk status and other confounding factors.

For peer review only - http://bmjopen.bmj.com/site/about/guidelines.xhtml

Table 7: Number of antenatal appointments attended in relation to the place of antenatal care

Number of antenatal appointments	Place of antenatal care	Number of women (%)	Unadjusted RR	Model 1 Adjusted RR (95% CI) *	Model 2 Adjusted RR (95% CI) **	Model 3 Adjusted RR (95% CI) ***
1-6	Hospital	106(39)	**0.53(0.37-0.77)**	**0.56(0.38-0.82)**	**0.47(0.31-0.71)**	**0.61(0.38-0.99)**
	Community	167(61)	Ref	Ref	Ref	Ref
7-9****	Hospital	120(54)	Ref	Ref	Ref	Ref
	Community	102(46)	Ref	Ref	Ref	Ref
10-14	Hospital	130(69)	**1.90(1.26-2.86)**	**2.22(1.44-3.41)**	**2.27(1.43-3.58)**	**2.70(1.62-4.49)**
	Community	58(31)	Ref	Ref	Ref	Ref
≥15	Hospital	60(72)	**2.21(1.28-3.83)**	**2.92(1.59-5.34)**	**3.36(1.80-6.25)**	**4.90(2.50-9.61)**
	Community	23(28)	Ref	Ref	Ref	Ref

* Model 1: Adjusted for demographics ethnicity, age, parity, IMD score, social risk, and medical risk factors
** Model 2: Model 1 plus adjusted for model of care
*** Model 3: Model 2 plus Adjusted for service provider attended
**** Set as base as WHO recommends 8 antenatal appointments [1]

Qualitative analysis

Flexible care was discussed by women accessing both specialist models, in terms of when and where appointments were scheduled and how long each appointment lasted. The community or home setting was seen as more convenient and supportive, particularly for those who were unfamiliar with UK transport systems, and those with little resource or young children. Women from both specialist models expressed ease of contacting the specialist model midwives through phone call, text messaging or email, felt that it reduced the number of face-to-face appointments they needed and that they could rely on the midwives to remind them of appointments. Women who did not speak fluent English (45%) particularly valued the ability to text their midwife as it gave them the opportunity to use translation technology. This flexibility appeared to encourage women to seek help more readily when they were concerned or needed reassurance. However, women in the hospital-based model did not always feel that the way care was scheduled suited their needs but structured around the hospital protocol and organisational efficiency.

If it's [antenatal care] like near to me it's OK but… I have so much financial problems so when she [HBM midwife] came to my house like, so much easier for me….and at the beginning I didn't know how to use bus... she helped me. (HBM6)

For peer review only - http://bmjopen.bmj.com/site/about/guidelines.xhtml

The quality of relational continuity and missed appointments

Quantitative Analysis 1- Model of care

The quantitative data was analysed to test the hypothesis that the specialist model of care increases the quality of relational continuity through the number of antenatal appointments and care in labour from a known healthcare professional (midwife, GP, or obstetrician), and reduces the number of appointments women miss, or do not attend. Table 8 shows that women receiving group practice and specialist models were significantly more likely to see a known healthcare professional during pregnancy. When adjusting the model for women's characteristics Black African women were the least likely group to see a known healthcare professional more than 5 times (RR0.17 CI 0.03-0.82). Women receiving care in the specialist model were significantly more likely to be looked after in labour by a known healthcare professional than the group practice (RR0.44 CI0.21-0.92).

For peer

.

Table 8: Number of appointments and support in labour by known healthcare professional

**Number of antenatal appointments with a known professional**	**Model of Care**	**Number of women (%)**	**Unadjusted OR**	**Model 1 Adjusted RR (95% CI) ***	**Model 2 Adjusted RR (95% CI) ****	**Model 3 Adjusted RR (95% CI) *****
None	Standard	313(69)	Ref	Ref	Ref	Ref
	Group	84(19)	Ref	Ref	Ref	Ref
	Specialist	53(12)	Ref	Ref	Ref	Ref
2	Standard	108(73)	**6.09(1.86-19.9)**	**4.43(1.30-15.0)**	2.75(0.78-9.67)	2.12(0.57-7.87)
	Group	36(24)	**7.57(2.21-25.8)**	**7.30(2.05-26.0)**	6.81(1.89-24.5)	1.81(0.44-7.45)
	Specialist	3(2)	Ref	Ref	Ref	Ref
3	Standard	36(47)	0.76(0.33-1.72)	0.69(0.27-1.77)	0.47 (0.17-1.29)	0.34(0.11-1.06)
	Group	32(42)	**2.52(1.08-5.89)**	**3.33(1.27-8.75)**	**3.15 (1.18-8.38)**	0.82(0.24-2.75)
	Specialist	8(11)	Ref	Ref	Ref	Ref
4	Standard	7(15)	0.29(0.83-1.04)	**0.23 (0.05-0.89)**	**0.16(0.03-0.67)**	**0.10(0.02-0.53)**
	Group	34(76)	**5.36(1.80-15.9)**	**5.99(1.78-20.1)**	**5.19(1.62-18.7)**	1.72(0.39-1.47)
	Specialist	4(9)	Ref	Ref	Ref	Ref
>5	Standard	5(10)	**0.76(0.02-0.23)**	**0.05(0.01-0.19)**	**0.03 (0.00-0.13)**	**0.02(0.00-0.11)**
	Group	35(68)	2.00(0.93-4.29)	2.50(0.96-6.49)	2.38 (0.90-6.32)	0.82(0.23-2.94)
	Specialist	11(22)	Ref	Ref	Ref	Ref
**Looked after in labour by known midwife**	Standard	235(63)	**0.41(0.24-0.71)**	**0.44(0.24-0.82)**	0.62 (0.32-1.19)	0.59(0.30-1.17)
	Group	81(23)	**0.24(0.13-0.42)**	**0.23 (0.12-0.44)**	**0.24 (0.12-0.47)**	**0.44(0.21-0.92)**
	Specialist	53(14)	Ref	Ref	Ref	Ref

For peer review only - http://bmjopen.bmj.com/site/about/guidelines.xhtml

* Model 1: Adjusted for demographics ethnicity, age, parity, IMD score, any social and medical risk factors at booking and onset of labour
** Model 2: Model 1 + Adjustment for place of antenatal care (community or hospital)
*** Model 3: Model 2 + Adjustment for service attended (A or B)

Quantitative Analysis 2- Place of antenatal care

Table 9 shows that women receiving hospital-based antenatal care were less likely to see a known healthcare professional for their antenatal appointments compared to those receiving community-based care. No significant relationship was found between the place of care and the number of women cared for in labour by their named midwife.

Table 9: Number of appointments with a known healthcare professional in relation to place of care

Number of antenatal appointments with a known professional	Place of antenatal care	Number of women (%)	Unadjusted OR	Model 1 Adjusted RR (95% CI) *	Model 2 Adjusted RR (95% CI) **	Model 3 Adjusted RR (95% CI) ***
None	Hospital<br>Community	215(48)<br>235(52)	Ref<br>Ref	Ref<br>Ref	Ref<br>Ref	Ref<br>Ref
2	Hospital<br>Community	109(74)<br>38(26)	**3.15 (2.08-4.76)**<br>Ref	**2.44(1.56-3.83)**<br>Ref	**2.71(1.63-4.50)**<br>Ref	**0.24(0.10-0.59)**<br>Ref
3	Hospital<br>Community	45(59)<br>31(41)	1.59 (0.97-2.61)<br>Ref	1.23(0.71-2.13)<br>Ref	**2.25(1.19-4.25)**<br>Ref	**0.23(0.08-0.62)**<br>Ref
4	Hospital<br>Community	20(44)<br>25(56)	0.87 (0.47-1.62)<br>Ref	0.61 (0.31-1.22)<br>Ref	2.02 (0.92-1.47)<br>Ref	**0.30(0.11-0.83)**<br>Ref
>5	Hospital<br>Community	28(55)<br>23(45)	1.33 (0.74-2.39)<br>Ref	0.70 (0.35-1.39)<br>Ref	**2.66 (1.24-5.70)**<br>Ref	**0.35(0.12-0.96)**<br>Ref
**Looked after in labour by named midwife**	Hospital<br>Community	153(41)<br>216(59)	**0.34(0.25-0.46)**<br>Ref	**0.46(0.33-0.64)**<br>Ref	**0.39(0.27-0.57)**<br>Ref	0.89(0.59-1.36)<br>Ref

* Model 1: Adjusted for demographics ethnicity, age, parity, IMD score, social risk, and medical risk factors
** Model 2: Model 1 plus adjusted for model of care
*** Model 3: Model 2 plus Adjusted for service attended

For peer review only - http://bmjopen.bmj.com/site/about/guidelines.xhtml

Missed appointments

Quantitative Analysis 1- Model of care

Table 10 shows that no significant relationship was found between model of care and the number of missed appointments. When adjusting for women's characteristics multiparous women were 4 times more likely (RR 4.50 CI 1.13-17.82) to miss 4 or more appointments then primiparous women, and the older a woman was the less likely she was to miss 4 or more appointments (RR0.03 CI 0.00-0.55). Black African women (RR 12.85 CI 2.42-68.07), and women with social risk factors (RR2.26 CI 1.14-4.47) were more likely to miss two or more appointments (See Appendix B). These findings should be viewed with caution due to the wide confidence intervals.

Table 10: Number of missed appointments in relation to model of care received

Number of missed appointments	Model of Care	Number of women (%)	Unadjusted OR	Model 1 Adjusted RR (95% CI) *	Model 2 Adjusted RR (95% CI) **	Model 3 Adjusted RR (95% CI) ***
None	Standard	352(63)	Ref	Ref	Ref	Ref
	Group	147(26)	Ref	Ref	Ref	Ref
	Specialist	62(11)	Ref	Ref	Ref	Ref
1	Standard	62(56)	1.36 (0.62-2.99)	1.58 (0.67-3.67)	1.46 (0.61-3.48)	1.44(0.61-3.49)
	Group	40(37)	2.10 (9.33-4.76)	**2.41 (1.01-5.77)**	2.38 (0.99-5.72)	1.97(0.80-4.84)
	Specialist	8(7)	Ref	Ref	Ref	Ref
	Standard	37(67)	1.62 (0.56-4.73)	2.27 (0.72-7.13)	1.79(0.54-5.84)	1.81(0.53-6.19)
2	Group	14(26)	1.47 (0.46-4.66)	1.83 (0.54-6.20)	1.79 (0.52-6.14)	0.86(0.22-3.33)
	Specialist	4(7)	Ref	Ref	Ref	Ref
	Standard	14(52)	1.23 (0.27-5.55)	2.71 (0.33-22.0)	2.01 (0.23-17.2)	2.02(0.22-18.4)
3	Group	11(41)	2.31 (0.49-10.7)	4.54 (0.54-37.9)	4.41 (0.51-37.6)	2.03(0.25-23.6)
	Specialist	2(7)	Ref	Ref	Ref	Ref
	Standard	4(24)	0.17(0.42-0.72)	**0.20 (0.04-0.98)**	0.22 (0.04-1.14)	0.23(0.04-1.22)
≥4	Group	9(53)	0.94(0.28- 3.19)	0.94 (0.22-4.02)	0.94 (0.21-4.05)	0.49(0.08-2.71)
	Specialist	4(23)	Ref	Ref	Ref	Ref

* Model 1: Adjusted for demographics ethnicity, age, parity, IMD score, any social and medical risk factors at booking and onset of labour
** Model 2: Model 1 + Adjustment for place of antenatal care (community or hospital)
*** Model 3: Model 2 + Adjustment for service provider attended (A or B)

Analysis 2- Place of antenatal care

Table 11 shows no significant relationship was found between the number of missed appointments and the place of antenatal care.

Table 11: Number of missed appointments in relation to place of care

Number of missed appointments	Place of Care	Number of women (%)	Unadjusted OR	Model 1 Adjusted RR (95% CI) *	Model 2 Adjusted RR (95% CI) **	Model 3 Adjusted RR (95% CI) ***
None	Hospital	292(52)	Ref	Ref	Ref	Ref
	Community	269(48)	Ref	Ref	Ref	Ref
1	Hospital	64(58)	1.28 (0.84-1.93)	1.11 (0.71-1.72)	1.21 (0.76-1.94)	0.95(0.56-1.62)
	Community	46(42)	Ref	Ref	Ref	Ref
2	Hospital	38(69)	**2.06 (1.13-3.74)**	**2.07 (1.12-3.88)**	**1.96 (1.01-3.78)**	0.64(0.26-1.58)
	Community	17(31)	Ref	Ref	Ref	Ref
3	Hospital	18(67)	1.84 (0.81-4.17)	1.82 (0.74-4.46)	2.18 (0.83-5.67)	1.02(0.32-3.20)
	Community	9(33)	Ref	Ref	Ref	Ref
≥4	Hospital	6(35)	0.50 (0.18-1.38)	0.48 (0.15-1.48)	0.77(0.23-2.51)	0.36(0.08-1.55)
	Community	11(65)	Ref	Ref	Ref	Ref

* Model 1: Adjusted for demographics ethnicity, age, parity, IMD score, social risk, and medical risk factors at booking
** Model 2: Model 1 plus adjusted for model of care
*** Model 3: Model 2 plus Adjusted for service provider attended

Qualitative analysis

Women accessing the community-based model perceived a high level of continuity of care from the whole team. Women accessing the hospital-based model discussed how they knew their named midwife but not necessarily the rest of the team, impacting on how they sought help. They were also not always aware of the level of continuity offered, expressed more anxiety about their labour care and discussed the impact of midwives needing to cancel appointments. For some, cancelled appointments and a lack of continuity had a significant impact on their support and engagement, leading to social care involvement. Women in both models described feeling comfortable when rebooking appointments they were unable to attend that appeared to reduce the number of 'missed' appointments recorded, seen as a mitigating effect of the specialist model.

For peer review only - http://bmjopen.bmj.com/site/about/guidelines.xhtml

'if I'm running late or something comes up I just text her or give her a call and she'll muddle things around' (HBM9)

Summary of findings

Table 12 summarises the quantitative findings in relation to either the model of care received, or the place of antenatal care, or both. Characteristics of women at disproportionate risk and differences between the services providers is also presented.

Table 12: Overview of qualitative outcomes

Access and Engagement Outcome variable	Characteristics of women at disproportionate risk when adjusting (Appendix B)	Significant effect of specialist model of care	Significant effect of hospital based antenatal care	Significant effect of service
Access to specialist model	Most deprived, social risk factors, Black African ethnicity	↑	↓	=
Late gestation at booking appointment (>20/40)	Primiparous, High medical risk, social risk factors	=	↑	=
Number of antenatal appointments outside of recommendations [1,2]	Social risk factors and high medical risk status (> 15 appointments)	=	↓/↑	B↓
Appointments with a known healthcare professional	Black African (least likely to see a known HCP)	=	↓	B↓
Missed appointments	Multiparous, Black African, social risk	=	=	B↑

↑= Statistically significant increase (Pr < 0.05)

↓ = Statistically significant decrease (Pr < 0.05)

= No significant relationship detected

For peer review only - http://bmjopen.bmj.com/site/about/guidelines.xhtml

'A' and 'B' refer to service providers

Refined programme theory

The qualitative interviews with women enabled the refinement of previously constructed programme theories [7,25], giving insight into specific, underlying mechanisms of improved access and engagement with maternity services. These are detailed in Table 13, providing a framework for those developing future models of care for women with social risk factors.

Table 13: Refined programme theory – Access and engagement with maternity services

Context +	Mechanism =	Outcome
Women who struggle to access services and are at greater risk of booking for maternity care at a late gestation.<br><br>Women who are unfamiliar of the UK health system or have difficulties in registering with health services. These women are often experiencing social risk factors that might lead to chaotic lives, social isolation, lack of resource, lack of support.	M1) If maternity care provision commences when a woman accesses services regardless of her gestation and women have 24/7 access to a small team of midwives whom they have had the opportunity to meet during pregnancy and are encouraged to contact via a phone call, text message or free technology	O1) Then women would not feel unsupported, anxious, and that the service does not value them until they have a viable pregnancy. This might also improve early access to safe abortion and family planning services. Engagement with services will improve through needs-based communication and appropriately timed antenatal appointments. This open access can work both ways through midwives reminding them of appointments, this leads to women feeling more 'cared for'.
	M2) If women are made aware of the possibility and how to self-refer to maternity services and specialist models of care by administrative staff at the first point of contact	O2)Then difficulties trying to access a GP will be overcome, the time spent waiting for a GP appointment reduced, and long referral processes between primary and secondary services will be avoided.
Primiparous women, those with any social risk factor, and high medical risk are more likely to book late for maternity care.	M3) If women can access a community-based service where GP's and midwives regularly communicate with each other	O3) Then the timing of access to a booking appointment with a midwife will be improved and convoluted referral pathways between community and hospital services avoided.
Multiparous women, Black African women and those with social risk factors are more likely to miss appointments. Black African women have less	M4) If women living in areas of deprivation are prioritised to receive continuity of care through community-based models	O4) Then services are likely to identify women with social risk factors who have not previously disclosed these issues with professionals, and care is less likely to be disrupted during pregnancy when a disclosure is made.

For peer review only - http://bmjopen.bmj.com/site/about/guidelines.xhtml

appointments with a known healthcare professional.	M5) If women are informed of the reasons why they have been allocated a continuity of care model or specialist service, or are able to self-refer to them if they feel they are eligible for their care	O5) Then the development of a trusting and open relationship with their healthcare provider will be enabled and feelings of suspicion and surveillance reduced. This transparency may also reduce feelings of discrimination for women in marginalised groups.
	M6) If women who book late for pregnancy care are eligible for specialist models of care where they have a named midwife or small team of midwives	O6) Then the benefits of these models of care and highlighted mechanisms may protect them from the disproportionately poor outcomes associated with late booking.
	M7) If women can reschedule appointments easily, and do not fear judgement or reproach when they miss appointments	O7) then they will perceive the maternity environment as a place of safety and their engagement with flexible services will improve.
	M8)If women have the opportunity to get to know all of the midwives in a small team throughout their pregnancy and the number, time and place of appointments is co-planned with women to meet their individual needs,	O8) then they will not feel disappointment or let down when their named midwife is unable to attend an appointment. Care, information, and responsibility will be shared across the team, women will be better able to engage, not perceive the pressure of time and feel more able to seek help and disclose information thus improving safety.

Discussion

In response to concerns that the most affluent and lowest risk women are often the most likely to receive the highest standards of maternity care [34], a clear example of Tudor Harts Inverse Care Law [35], this study examines a shift change to more proportionate universalism through services targeting those who are at highest risk of poor outcomes. The aims of the specialist models of care- to reach those who are most at risk of poor outcomes and experiences, are being met. This is an important finding as it addresses the aims of the NHS Ten Year plan [36] to improve outcomes and care experiences through the offer of continuity of care to Black and minority ethnic women and those living in deprived areas. The findings suggest that placing continuity of care models in areas of deprivation are likely to identify women who go on to disclose social risk factors during pregnancy. In contrast, strict inclusion criteria to

For peer review only - http://bmjopen.bmj.com/site/about/guidelines.xhtml

hospital-based models risk missing those women who are at increased risk but are yet to disclose social risk factors, leading to fragmented care and further risk of 'falling through the gaps'.

Overall women found the referral to both specialist models of care acceptable, but some expressed a lack of choice, and perceived discrimination about being left in the dark for the reason for referral. There is a wealth of literature around healthcare professionals reluctance to address sensitive issues such as mental health, social risk factors and cultural differences with women [37–3937,39–45]. In addition to this, socioeconomic status and ethnicity are often associated with stigma and discrimination in healthcare services [46–49]. These factors might contribute to midwives' feelings of discomfort around informing women why they have been referred to a specialist model. Perceived discrimination could be avoided through community-based care within areas of deprivation rather than inclusion criteria based on these social risk factors.

The findings showed no differences between the model of care received and timing of access to maternity care or the number of antenatal appointments women attended. Considering women in the specialist models were more likely to have low socioeconomic status and social risk factors and therefore more likely to struggle to access and engage [50–52] this indicates that the inequality in access may have been mitigated by the specialist model of care. This theory would need to be tested with a larger sample. Regardless of the model of care women received, those receiving hospital-based care were more likely to attend their first maternity appointment after 20 weeks' gestation, this was thought to be partly due to convoluted referral systems between the community and hospital. Many women expressed wanting to be seen earlier in pregnancy, challenging the notion that women with social risk factors do not prioritise their maternity care [53]. Women also described difficulties in registering or booking an appointment with their GP when they found out they were pregnant, particularly if they did not speak English, were homeless, or unfamiliar with the system. The wider literature has identified similar barriers to access including; difficulties navigating the health system and service delays in the processing of referrals [54–56]. A UK study [57] of women accessing antenatal care in a multicultural, deprived area found that women want to access care in early pregnancy but perceive antenatal care for viable and continuing pregnancies at a later gestation. If women do not feel that a service is open to them, or that maternity services only value those who carry a viable pregnancy, they may internalise this as a prioritisation of the wellbeing of the fetus over their own emotional, physical, and social needs. As well as impacting on women's engagement with services this can impact on the safety of women with complex needs such as those who are experiencing abuse, or need to discuss a termination of pregnancy.

For peer review only - http://bmjopen.bmj.com/site/about/guidelines.xhtml

A recent review of the literature on how women with social risk factors experience maternity care in the UK found reasons for late access include the denial of services based on a lack of documentation, fear of disclosure to immigration services, language and financial barriers, cultural differences, unfamiliarity, a lack of trust, and a perception that maternity services act as a system of surveillance rather than support [58]. Practice recommendations detailed in the refined programme theory suggest that all women are made aware of the possibility to self-refer directly to maternity services at the first point of contact with health services, using language appropriate information. Strict inclusion criteria for access to specialist models that restrict women who have booked late for maternity care should be reviewed as these women are often high risk and can benefit from the specialist model regardless of their gestation. An evaluation of group antenatal care [59] included women who had booked later in pregnancy with positive feedback and no negative impact on group dynamics. With the national expansion of continuity of care models [18], inclusion criteria's could be relaxed somewhat as the demands on the service are more evenly distributed. Future research should assess the impact of place-based midwifery continuity of care on health inequalities to test the outcomes of this preference.

This study reflects findings in the wider literature [52,60] highlighting service use inequities for women with particular characteristics such as multiparity, age, and ethnicity and presents an opportunity for those designing models of care to focus on these demographics. Where poor engagement with services is often associated with women's priorities and behaviours [7,56,61], the women in this study highlighted system barriers. Interestingly, many women in this study felt that the ability to contact a known midwife anytime reduced the number of face-to-face appointments they needed. Where engagement in this study was measured through the number of antenatal appointments attended, it would be useful for future research to measure other forms of contact between women and healthcare professionals. Women from the hospital-based specialist model discussed the detrimental impact of midwives needing to cancel their appointments. Rather than a place-based issue this seems to relate to how the teams are organised as those in the hospital-based model were allocated one midwife who they saw for most of their appointments, whereas women accessing the community-based model described being cared for by the whole team. Finally, women from both community and hospital-based models expressed a preference for care to be based in the community or home setting as they felt it was more accessible and supportive of their needs.

For peer review only - http://bmjopen.bmj.com/site/about/guidelines.xhtml

The aim of both the group practice and specialist models evaluated appear to be being met with women more likely to receive more antenatal appointments with a known healthcare professional from these models. Women who received hospital-based antenatal care had significantly lower levels of continuity in the antenatal period. Women receiving care in the specialist model were more likely to be looked after in labour by a known healthcare professional compared to the other models. The qualitative data revealed this continuity lessened anxiety, the need to repeat often complex social and medical histories to numerous professionals, and increased disclosure and preparedness for labour and birth, particularly for those who had the opportunity to meet the rest of the team. Multiparous, younger, and Black African women were more likely to miss antenatal appointments, as well as those with social risk factors. Black African women were also less likely to see a known healthcare professional than women of other ethnicities, perhaps reflecting an underlying mechanism of the health inequalities they experience [62]. There is a significant paucity of literature around Black women's experiences of UK maternity care, but a US review [63] concluded that midwifery care can accommodate their specific needs through attentive provider, continuity of care, and empowerment. The review stated that '*researchers must meet, consult with, and listen to Black women and hear their stories to understand the significance of midwifery to them. Only then, in partnership with, and based on suggestions for change from Black women themselves, can health care providers and researchers begin to make changes in the health care system to facilitate improved antenatal care'* [63]. This recommendation is as relevant to the UK context given the known stark inequalities.

Strengths and limitations

Women interviewed during the study may have perceived the study questions to be testing them about their willingness to engage with their care. This limitation may have been lessened through the trust built between the participant and the researcher over the course of the longitudinal interviews [64,65]. The relatively small numbers in each quantitative data group should be taken into consideration due to the significant amount of multiple testing required to establish the separate effects of the model of care, place of care and service attended. This presents a potential limitation as the use of multiple testing can result in a substantial change in statistical power, reducing the probability of detecting effects when they do exist [66]. This could be overcome in future research using larger sample sizes to test the apparent mitigating effects of the specialist models of care on inequalities in access and engagement. Finally, the generalisability of the findings is limited by the urban location of both specialist models of care evaluated. This is particularly significant when reflecting on the outcomes relating to place-based care-what may have yielded significant outcomes in a densely populated, multicultural community, may yield

For peer review only - http://bmjopen.bmj.com/site/about/guidelines.xhtml

very different results elsewhere. Research is needed to test the generalisability of the findings to rural and other community settings.

Conclusion

This research highlights how carefully considered place-based care with a focus on continuity of carer can improve access and engagement with maternity services for women with social risk factors, but it is not a panacea. Hospital-based models of care with strict inclusion criteria may risk excluding women at increased risk who are yet to disclose social risk factors. Rather than the often-assumed 'relaxed maternal care-seeking behaviour' and 'women with social risk factors deprioritising maternity care' explanations for late booking and missed appointments, the findings highlight system barriers. Women want to be able to access care earlier in pregnancy, and there appears to be a lack of information regarding their choices in doing so, particularly for those who do not speak English or are unfamiliar with the system. The identification of specific mechanisms that improve access and engagement with services are outlined in the refined programme theory, which will enable those developing maternity services to structure models of care around women's individual need.

Availability of data and material- full quantitative data analysis attached as additional file or contact the lead author HRJ for qualitative and raw data files that are available upon reasonable request.

Ethical approval- The research was approved by the London Brent Research Ethics Committee (HRA) REC Reference 18-LO-0701.

Competing interests - None declared

Funding- This report is independent research supported by the National Institute for Health Research (NIHR Doctoral Research Fellowship, Mrs Hannah Rayment-Jones, award no.DRF-2017-10-033). Angela Harden is supported by the NIHR Applied Research Collaboration North Thames. Jane Sandall (King's College London) is supported by the NIHR Applied Research Collaboration South London [NIHR ARC South London]. Jane Sandall is also an NIHR Senior Investigator. James Harris is supported by a Post-doctoral Fellowship from Wellbeing of Women (Award Ref PRF006). Kathryn Dalrymple is funded by the MRC (grant number: MR/V005839/1)The views expressed in this article are

For peer review only - http://bmjopen.bmj.com/site/about/guidelines.xhtml

those of the author(s) and not necessarily those of the NHS, the NIHR, the MRC, or the Department of Health and Social Care.

Authors' contributions- Authors HRJ, JH, AH, JS contributed to the conceptualisation of the research question, and methodology. Authors EP and TG organised collection and anonymisation of the quantitative data, HRJ and KD analysed the quantitative data. HRJ collected qualitative data and analysed with JH. All authors interpreted the data analysis, read and approved the final manuscript.

Acknowledgements- The authors would like to thank student midwives Laura Peazold, Mary Newman, Natalie Goodyear and Micaela Anthony for help with data collection and anonymisation, Justin Jagosh for invaluable advice on realist methodology and Sergio Silverio for support with qualitative data analysis.

Reporting checklist- We used the SQUIRE checklist [67] when writing our report. Then cite this checklist as Ogrinc G, Davies L, Goodman D, Batalden P, Davidoff F, Stevens D. SQUIRE 2.0 (Standards for QUality Improvement Reporting Excellence): revised publication guidelines from a detailed consensus process.

Figure legends: Figure 1: Quantitative data collection flowchart

Word count- 4793

For peer review only

For peer review only

For peer review only - http://bmjopen.bmj.com/site/about/guidelines.xhtml

References

1. World Health Organization (WHO). WHO Recommendations on Antenatal Care for a Positive Pregnancy Experience: Summary. *World Health Organization* (2018) doi:10.1186/1742-4755-10-19.5.
2. National Institute for Clinical Excellence (NICE). *Antenatal care. Quality standard [QS22]*. (2012).
3. Nam, J. Y., Cho, E. & Park, E. C. Do severe maternal morbidity and adequate prenatal care affect the delivery cost? A nationwide cohort study for 11 years with follow up. *BJOG: An International Journal of Obstetrics and Gynaecology* (2019) doi:10.1111/1471-0528.15895.
4. Renfrew, M. J. *et al.* Midwifery and quality care: Findings from a new evidence-informed framework for maternal and newborn care. *The Lancet* (2014) doi:10.1016/S0140-6736(14)60789-3.
5. Shaw, D. *et al.* Drivers of maternity care in high-income countries: can health systems support woman-centred care? *The Lancet* (2016) doi:10.1016/S0140-6736(16)31527-6.
6. Lindquist, A., Kurinczuk, J. J., Redshaw, M. & Knight, M. Experiences, utilisation and outcomes of maternity care in England among women from different socio-economic groups: Findings from the 2010 National Maternity Survey. *BJOG: An International Journal of Obstetrics and Gynaecology* (2015) doi:10.1111/1471-0528.13059.
7. Rayment-Jones, H., Harris, J., Harden, A., Khan, Z. & Sandall, J. How do women with social risk factors experience United Kingdom maternity care? A realist synthesis. *Birth* **46**, 461–474 (2019).
8. Downe, S., Finlayson, K., Tunçalp & Metin Gülmezoglu, A. What matters to women: A systematic scoping review to identify the processes and outcomes of antenatal care provision that are important to healthy pregnant women. *BJOG: An International Journal of Obstetrics and Gynaecology* (2016) doi:10.1111/1471-0528.13819.
9. Finlayson, K. & Downe, S. Why Do Women Not Use Antenatal Services in Low- and Middle-Income Countries? A Meta-Synthesis of Qualitative Studies. *PLoS Medicine* (2013) doi:10.1371/journal.pmed.1001373.
10. Arsenault, C. *et al.* Equity in antenatal care quality: an analysis of 91 national household surveys. *The Lancet Global Health* (2018) doi:10.1016/S2214-109X(18)30389-9.
11. Nair, M., Knight, M. & Kurinczuk, J. J. Risk factors and newborn outcomes associated with maternal deaths in the UK from 2009 to 2013: a national case–control study. *BJOG: An International Journal of Obstetrics and Gynaecology* (2016) doi:10.1111/1471-0528.13978.
12. Amjad, S., Chandra, S., Osornio-Vargas, A., Voaklander, D. & Ospina, M. B. Maternal Area of Residence, Socioeconomic Status, and Risk of Adverse Maternal and Birth Outcomes in Adolescent Mothers. *Journal of Obstetrics and Gynaecology Canada* (2019) doi:10.1016/j.jogc.2019.02.126.
13. Knight, Marian., Bunch, Kathryn., Kenyon, Sara., Tuffnell, Derek., Kurinczuk, Jennifer J., Shakespear, Judy., Kotnis, Rohit. *Saving Lives, Improving Mothers' Care. Lessons learned*

For peer review only

to inform maternity care from the UK and Ireland Confidential Enquiries into Maternal Deaths and Morbidity 2015-17. (2019).

14. Ki-Moon, B. Global strategy for women's and children's health. in *Maternal Mortality, Human Rights and Accountability* (2013). doi:10.4324/9780203684214.
15. (UK), N. C. C. for W. and C. H. *Pregnancy and Complex Social Factors. Pregnancy and Complex Social Factors: A Model for Service Provision for Pregnant Women with Complex Social Factors* (2010).
16. Organization, world health. WHO Recommendation on Antenatal care for positive pregnancy experience. *WHO Recommendation on Antenatal care for positive pregnancy experience* (2016) doi:ISBN 978 92 4 154991 2.
17. Kennedy, H. P. *et al.* Asking different questions: research priorities to improve the quality of care for every woman, every child. *The Lancet Global Health* (2016) doi:10.1016/S2214-109X(16)30183-8.
18. NHS England. *The NHS Long Term Plan: Maternity and neonatal services*. (2019).
19. National Maternity Review. *Better Births: Improving outcomes of maternity services in England. National Maternity Review* (2016).
20. Sandall, J., Soltani, H., Gates, S., Shennan, A. & Devane, D. Midwife-led continuity models versus other models of care for childbearing women. *Cochrane Database of Systematic Reviews* (2016) doi:10.1002/14651858.CD004667.pub5.
21. Hollowell, J., Kurinczuk, J. J., Oakley, L., Brocklehurst, P. & Gray, R. A systematic review of the effectiveness of antenatal care programmes to reduce infant mortality and its major causes in socially disadvantaged and vulnerable women. *National Perinatal Epidemiology Unit (NPEU)* (2009).
22. Pawson, R. *The Science of Evaluation: A Realist Manifesto*. *The Science of Evaluation: A Realist Manifesto* (2014). doi:10.4135/9781473913820.
23. Jagosh, J. Realist Synthesis for Public Health: Building an Ontologically Deep Understanding of How Programs Work, For Whom, and In Which Contexts. *Annual Review of Public Health* (2019) doi:10.1146/annurev-publhealth-031816-044451.
24. Rayment-Jones, H., Harris, J., Harden, A., Khan, Z. & Sandall, J. How do women with social risk factors experience United Kingdom maternity care? A realist synthesis. *Birth* (2019) doi:10.1111/birt.12446.
25. Rayment-Jones, H., Silverio, S. A., Harris, J., Harden, A. & Sandall, J. Project 20: Midwives' insight into continuity of care models for women with social risk factors: what works, for whom, in what circumstances, and how. *Midwifery* (2020) doi:10.1016/j.midw.2020.102654.
26. Murray, S. F., Buller, A. M., Bewley, S. & Sandall, J. Metrics for monitoring local inequalities in access to maternity care: Developing a basket of markers from routinely available data. *Quality and Safety in Health Care* (2010) doi:10.1136/qshc.2008.032136.
27. Ministry of Housing, C. & L. G. *English indices of deprivation 2019. National Statistics.* (2019).
28. Office for National Statistics. The English Indices of Deprivation 2015. *Neighbourhoods Statistical Release* (2015) doi:http://dx.doi.org/http://www.communities.gov.uk/publications/corporate/statistics/indices2010technicalreport.

For peer review only

29. Manzano, A. The craft of interviewing in realist evaluation. *Evaluation* (2016) doi:10.1177/1356389016638615.
30. Ministry of Housing Communities and Local Government. English indices of deprivation 2019. *National Statistics* (2019).
31. Prel, J.-B. du, Hommel, G., Röhrig, B. & Blettner, M. Confidence Interval or P-Value? Part 4 of a Series on Evaluation of Scientific Publications. *Deutsches Aerzteblatt Online* (2009) doi:10.3238/arztebl.2009.0335.
32. Gale, N. K., Heath, G., Cameron, E., Rashid, S. & Redwood, S. Using the framework method for the analysis of qualitative data in multi-disciplinary health research. *BMC Medical Research Methodology* (2013) doi:10.1186/1471-2288-13-117.
33. Vasileiou, K., Barnett, J., Thorpe, S. & Young, T. Characterising and justifying sample size sufficiency in interview-based studies: Systematic analysis of qualitative health research over a 15-year period. *BMC Medical Research Methodology* (2018) doi:10.1186/s12874-018-0594-7.
34. Kirkham, M., Stapleton, H., Curtis, P. & Thomas, G. The inverse care law in antenatal midwifery care. *British Journal of Midwifery* (2002) doi:10.12968/bjom.2002.10.8.10600.
35. Tudor Hart, J. THE INVERSE CARE LAW. *The Lancet* (1971) doi:10.1016/S0140-6736(71)92410-X.
36. Charles, A., Ewbank, L., McKenna, H. & Wenzel, L. The NHS long-term plan explained. . *Long read ;* (2019).
37. Fontein-Kuipers, Y. J., Budé, L., Ausems, M., de Vries, R. & Nieuwenhuijze, M. J. Dutch midwives' behavioural intentions of antenatal management of maternal distress and factors influencing these intentions: An exploratory survey. *Midwifery* (2014) doi:10.1016/j.midw.2013.06.010.
38. Everitt, L., Homer, C. & Fenwick, J. Working with Vulnerable Pregnant Women Who Are At Risk of Having their Babies Removed by the Child Protection Agency in New South Wales, Australia. *Child Abuse Review* (2017) doi:10.1002/car.2432.
39. Higgins, A., Carroll, M., Downes, C., Monahan, M., Gill, A., Madden, D., McGoldrick, E. & Nagle, U. Perinatal mental health: an exploration of practices, policies, processes and education needs of nurses and midwives within maternity and primary care services in Ireland. . *Dublin: Health Services Executive* (2017).
40. Baldwin, S. & Griffiths, P. Do specialist community public health nurses assess risk factors for depression, suicide, and self-harm among south asian mothers living in London?: Special features: Clinical concepts. *Public Health Nursing* (2009) doi:10.1111/j.1525-1446.2009.00780.x.
41. Jomeen, J., Glover, L., Jones, C., Garg, D. & Marshall, C. Assessing women's perinatal psychological health: Exploring the experiences of health visitors. *Journal of Reproductive and Infant Psychology* (2013) doi:10.1080/02646838.2013.835038.
42. Jane, R. Midwives, society and childbirth: debates and controversies in the modern period. *Women's History Review* (1999) doi:10.1080/09612029900200394.
43. Everitt, L., Homer, C. & Fenwick, J. Working with Vulnerable Pregnant Women Who Are At Risk of Having their Babies Removed by the Child Protection Agency in New South Wales, Australia. *Child Abuse Review* (2017) doi:10.1002/car.2432.

For peer review only

For peer review only - http://bmjopen.bmj.com/site/about/guidelines.xhtml

44. Mulherin, K., Miller, Y. D., Barlow, F. K., Diedrichs, P. C. & Thompson, R. Weight stigma in maternity care: Women's experiences and care providers' attitudes. *BMC Pregnancy and Childbirth* (2013) doi:10.1186/1471-2393-13-19.
45. Radcliffe, P. Substance-misusing women: Stigma in the maternity setting. *British Journal of Midwifery* (2011) doi:10.12968/bjom.2011.19.8.497.
46. Stepanikova, I. & Oates, G. R. Perceived Discrimination and Privilege in Health Care: The Role of Socioeconomic Status and Race. *American Journal of Preventive Medicine* (2017) doi:10.1016/j.amepre.2016.09.024.
47. Tajeu, G. S. *et al.* "We'll get to you when we get to you": Exploring potential contributions of health care staff behaviors to patient perceptions of discrimination and satisfaction. *American Journal of Public Health* (2015) doi:10.2105/AJPH.2015.302721.
48. Phelan, J. C., Lucas, J. W., Ridgeway, C. L. & Taylor, C. J. Stigma, status, and population health. *Social Science and Medicine* (2014) doi:10.1016/j.socscimed.2013.10.004.
49. Walker, R. *The Shame of Poverty*. *The Shame of Poverty* (2014). doi:10.1093/acprof:oso/9780199684823.001.0001.
50. Hatherall, B. *et al.* Timing of the initiation of antenatal care: An exploratory qualitative study of women and service providers in East London. *Midwifery* (2016) doi:10.1016/j.midw.2016.02.017.
51. Rowe, R. E. *et al.* Social and ethnic differences in attendance for antenatal care in England. *Public Health* (2008) doi:10.1016/j.puhe.2008.05.011.
52. Lindquist, A., Kurinczuk, J. J., Redshaw, M. & Knight, M. Experiences, utilisation and outcomes of maternity care in England among women from different socio-economic groups: Findings from the 2010 National Maternity Survey. *BJOG: An International Journal of Obstetrics and Gynaecology* (2015) doi:10.1111/1471-0528.13059.
53. Haddrill, R., Jones, G. L., Mitchell, C. A. & Anumba, D. O. C. Understanding delayed access to antenatal care: A qualitative interview study. *BMC Pregnancy and Childbirth* (2014) doi:10.1186/1471-2393-14-207.
54. Hollowell, Jennifer., Oakley C., Vigurs, E,. Barnett-Page, J. Kavanagh, and S. Oliver. Increasing the early initiation of antenatal care by black and minority ethnic women in the United Kingdom: a systematic review and mixed methods synthesis of women's views and the literature on intervention effectiveness. *Social Science Research Unit, Institute of Education, University of London* (2012).
55. Cresswell, J. A. *et al.* Predictors of the timing of initiation of antenatal care in an ethnically diverse urban cohort in the UK. *BMC Pregnancy and Childbirth* (2013) doi:10.1186/1471-2393-13-103.
56. Hatherall, B. *et al.* Timing of the initiation of antenatal care: An exploratory qualitative study of women and service providers in East London. *Midwifery* (2016) doi:10.1016/j.midw.2016.02.017.
57. Hatherall, B. *et al.* Timing of the initiation of antenatal care: An exploratory qualitative study of women and service providers in East London. *Midwifery* (2016) doi:10.1016/j.midw.2016.02.017.
58. Rayment-Jones, H., Harris, J., Harden, A., Khan, Z. & Sandall, J. How do women with social risk factors experience United Kingdom maternity care? A realist synthesis. *Birth* (2019) doi:10.1111/birt.12446.

For peer review only - http://bmjopen.bmj.com/site/about/guidelines.xhtml

59. Hunter, L. J. *et al.* Better together: A qualitative exploration of women's perceptions and experiences of group antenatal care. *Women and Birth* (2019) doi:10.1016/j.wombi.2018.09.001.
60. Higginbottom, G. M. A. *et al.* Experience of and access to maternity care in the UK by immigrant women: A narrative synthesis systematic review. *BMJ Open* (2019) doi:10.1136/bmjopen-2019-029478.
61. Docherty, A., Bugge, C. & Watterson, A. Engagement: An indicator of difference in the perceptions of antenatal care for pregnant women from diverse socioeconomic backgrounds. *Health Expectations* (2012) doi:10.1111/j.1369-7625.2011.00684.x.
62. MBRRACE-UK. *Saving Lives, Improving Mother's Care- Lay summary*. *Action against Medical Accidents)* (2018).
63. Yoder, H. & Hardy, L. R. Midwifery and Antenatal Care for Black Women: A Narrative Review. *SAGE Open* (2018) doi:10.1177/2158244017752220.
64. Grinyer, A. & Thomas, C. The value of interviewing on multiple occasions or longitudinally. in *The SAGE Handbook of Interview Research: The Complexity of the Craft* (2012). doi:10.4135/9781452218403.n15.
65. Bhopal, K. & Deuchar, R. *Researching marginalized groups*. *Researching Marginalized Groups* (2015). doi:10.4324/9781315740782.
66. Miller, R. Multiple Comparisons-I. in *Encyclopedia of Statistical Sciences* (2006). doi:10.1002/0471667196.ess1701.pub2.
67. Ogrinc, G. *et al.* SQUIRE 2.0 (Standards for QUality Improvement Reporting Excellence): revised publication guidelines from a detailed consensus process. *BMJ Quality & Safety* **25**, 986–992 (2016).

For peer review only

For peer review only - http://bmjopen.bmj.com/site/about/guidelines.xhtml

For peer review only

For peer review only - http://bmjopen.bmj.com/site/about/guidelines.xhtml

For peer review only

For peer review only - http://bmjopen.bmj.com/site/about/guidelines.xhtml

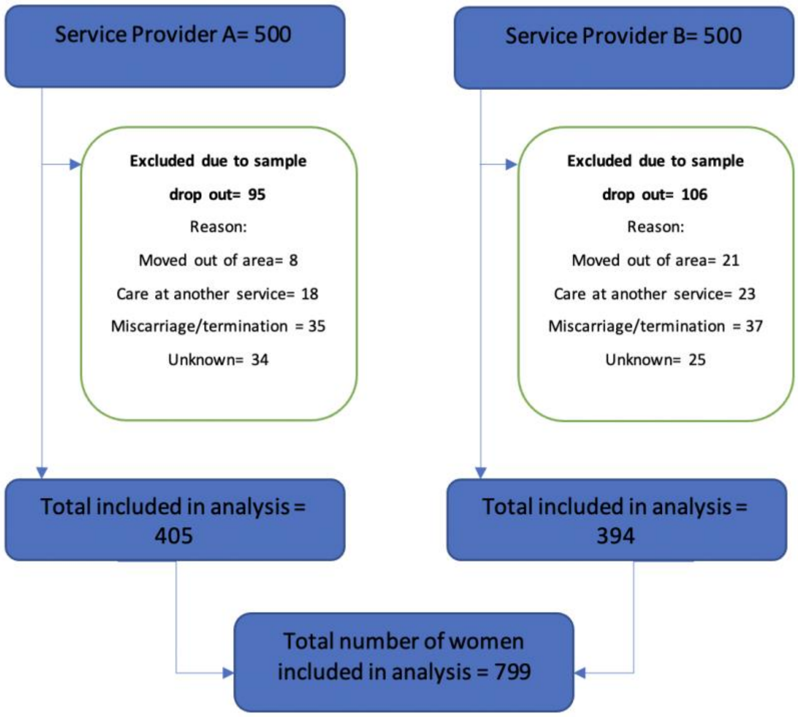

Figure 1: Quantitative data collection flowchart

For peer review only

Appendix 1: Definitions

Table 1: Definitions of the different models of care received by women at the two service providers evaluated

**Service A**	**Standard Care:** Depending on medical risk factors women receive antenatal and postnatal care as set by NICE guidance [2] in either the community setting or hospital setting. For low risk women care is usually provided by a community midwife in a GP surgery or local children's centre. For women at high medical risk care is provided at the hospital and shared between midwives and obstetricians. Although women may be assigned a 'named' healthcare professional, there is no emphasis on the provision of continuity of care.<br><br>**Group Practice:** Women are seen in either the community or hospital setting depending on their medical risk status. There is an aim to provide antenatal and postnatal continuity of care. Women have a named midwife who aims to see them for most of their antenatal and postnatal appointments. Intrapartum care is covered by the hospitals labour ward or birth centre staff. Women planning a homebirth will be looked after by on-call midwives, but this may be from a team not known to the woman.<br><br>**Specialist model: - Community-based Model [CBM]**<br>A team of 6 midwives provide continuity of care to women located in an area of social deprivation. Not all women under their care will have social risk factors. Each woman is assigned a named midwife who coordinates all care, multi-disciplinary communication, and referrals. The named midwife aims to provide most of the clinical care, with others in the team providing care when s/he is not on duty. The midwives are based in a local community health centre and offer antenatal, intrapartum, and postnatal care in the home, community, or hospital setting.
**Service B**	**Standard Care:** Depending on medical risk factors women receive antenatal and postnatal care as set by NICE guidance [2]. If women do not live within the geographical catchment areas of the group practices (see definition below), care is usually provided at the hospital and shared between midwives and obstetricians. Although women may be assigned a 'named' healthcare professional, there is no emphasis on the provision of continuity of care.<br><br>**Group practice** Women are seen in different settings depending on their medical risk status but there is an aim to provide antenatal and postnatal continuity of care. For most women who live within the hospital's geographical catchment area, care is provided in the community setting, often out of children's centres to prevent women from having to travel to the hospital for appointments. Postnatal care is provided at home and in postnatal clinics in the community. Women have a named midwife who aims to see them for most of their antenatal and postnatal appointments. Intrapartum care is covered by the hospitals labour ward or birth centre staff. Women planning a homebirth are looked after by a team of midwives providing on call care, with the aim for the midwives to have met the woman before.<br><br>**Specialist model: - Hospital-based Model [HBM]**<br>A team of 6 midwives provide continuity of care to women with social risk factors only. Women with one or more significant social risk factors (see Appendix A for inclusion criteria) are referred to the team and assigned a named midwife who coordinates all care, multi-disciplinary communication, and referrals. The named midwife aims to provide most of the clinical care, with others in the team providing care when she/he is not on duty. The midwives are based at the hospital site and offer antenatal, intrapartum, and postnatal care in the home or hospital setting.

Table 2: General definitions

Term	Definition
Deprivation score	A composite measure using routine data from the seven domains of deprivation to identify the most disadvantaged areas in England, UK [2]
Ethnicity	Using the ONS 18+ categories of ethnicity
Medical risk status	As recorded by healthcare professional entering routinely collected maternity record data. High medical risk status leads to obstetric-led care although many women will continue to see both obstetricians and midwives during their pregnancy. Low medical risk status leads to midwife-led care throughout pregnancy, birth and the postnatal period if the pregnancy remains low risk/uncomplicated.
Continuity of Care	The midwife is the lead professional in the planning, organisation and delivery of care given to a woman from initial booking to the postnatal period
Social Risk Factors	As recorded by healthcare professional entering routinely collected maternity record data and includes:<br>Domestic abuse- an incident or pattern of incidents of controlling, coercive, threatening, degrading and violent behaviour, including sexual violence, in the majority of cases by a partner or ex-partner, but also by a family member or carer.<br>Common mental health- Includes depression, anxiety, post-traumatic stress disorder, and obsessive-compulsive disorder.<br>Severe mental health-Includes psychological problems that are so debilitating that they effect peoples ability to engage in functional and occupational activities. Includes schizophrenia, bipolar disorder and psychosis<br>Non-English speaking<br>Social care involvement- previous or current involvement with social care<br>Drug/alcohol abuse<br>Unsupported/single<br>Financial/housing<br>Learning disability<br>Sexual abuse/trafficked<br>AS/Refugee<br>FGM- Female genital mutilation<br>No recourse to public funds
Demographics	**Level of education (completed)**<br>Secondary school only<br>Completed college<br>Completed university<br>**Occupation Status (NS- SEC)**<br>8 (long term unemployed)<br>7 (routine occupations)<br>6-3 (semi-routine)

	Recorded as an episode of care each time a woman has a face-to-face appointment with an obstetrician or midwife during their pregnancy
**Number of antenatal appointments**	
**Number of antenatal appointments with a known professional**	Recorded as an episode of care each time a woman has a face-to-face appointment with an obstetrician or midwife that they have previously met during their pregnancy. The maximum number of appointments with one healthcare professional is reported for the purpose of this paper.
**Looked after in labour by known midwife**	Looked after at some point during intrapartum care by a healthcare professional seen at least once during the antenatal period.
**Number of missed appointments**	Recorded as an appointment that was scheduled but not carried out or attended.

For peer review only

Appendix B: Supplementary data- Adjusted and unadjusted data analysis tables and qualitative data

Analysis 1: Model of care accessed by deprivation score and risk factors

Table 1: Maternity care received by deprivation score

Multinomial logistic regression — Number of obs = 765; LR chi2(57) = 153.19; Prob > chi2 = 0.0000; Pseudo R2 = 0.0747

Log likelihood = -948.68392

imd_3_score	RRR	Std. Err.	z	P>|z|	[95% Conf.	Interval]
**most_deprived**						
mod_care_4						
standard	.291514	.1930548	-1.86	0.063	.0796072	1.067497
partial CoC	.1270207	.0863271	-3.04	0.002	.0335253	.481257
ethnicity						
BA	8.688427	6.039025	3.11	0.002	2.224848	33.92985
BC	4.741514	3.985545	1.85	0.064	.91291	24.6267
BO	3.775191	4.386607	1.14	0.253	.3871507	36.81272
M	1.711281	1.391649	0.66	0.509	.3476215	8.424344
U	2.078345	.9214765	1.65	0.099	.8716061	4.955816
WB	.6403515	.2711355	-1.05	0.292	.279258	1.468356
WO	1.002714	.3973854	0.01	0.995	.4611452	2.180302
WP	2.391133	13830.83	0.00	1.000	0	.
age_cat						
20-24	1.37e-06	.0012548	-0.01	0.988	0	.
25-29	1.19e-06	.0010827	-0.01	0.988	0	.
30-34	4.47e-07	.0004081	-0.02	0.987	0	.
greater than 34	2.79e-07	.0002551	-0.02	0.987	0	.
0.parity	.6508242	.1653202	-1.69	0.091	.395589	1.070738
1.any_risk	1.684199	.5775913	1.52	0.129	.8599561	3.298455
2.high_risk_num	2.372965	.9909666	2.07	0.039	1.046704	5.379712
place_hosp_comm						
hospital	.4068348	.1188187	-3.08	0.002	.2295193	.7211356
imp_gstt						
imp	2.764661	.8701099	3.23	0.001	1.491925	5.123148
_cons	1.15e+07	1.05e+10	0.02	0.986	0	.
**3rd_and_4th_deciles**						
mod_care_4						
standard	.2897059	.1877638	-1.91	0.056	.081335	1.031898
partial CoC	.1905813	.1257227	-2.51	0.012	.052307	.6943858
ethnicity						
BA	5.239074	3.616894	2.40	0.016	1.353978	20.27205
BC	2.414152	2.031332	1.05	0.295	.4640247	12.55996
BO	7.223335	7.936432	1.80	0.072	.8385027	62.22588
M	1.297728	1.029289	0.33	0.742	.2741926	6.142027
U	2.248795	.9372376	1.94	0.052	.9935552	5.089884
WB	.689703	.2667646	-0.96	0.337	.3231713	1.471945
WO	1.130883	.4187918	0.33	0.740	.5472657	2.336883
WP	3.09e+07	1.41e+11	0.00	0.997	0	.
age_cat						
20-24	1.58e-06	.001445	-0.01	0.988	0	.
25-29	9.24e-07	.0008435	-0.02	0.988	0	.
30-34	5.30e-07	.0004843	-0.02	0.987	0	.
greater than 34	2.87e-07	.0002617	-0.02	0.987	0	.
0.parity	.66367	.1575855	-1.73	0.084	.4167141	1.056979
1.any_risk	1.480933	.4932381	1.18	0.238	.7709656	2.844695
2.high_risk_num	2.558484	.9732096	2.47	0.014	1.213942	5.392215
place_hosp_comm						
hospital	.4245959	.1120848	-3.25	0.001	.2530904	.7123212
imp_gstt						
imp	1.547321	.4430837	1.52	0.127	.8827429	2.71223
_cons	1.86e+07	1.70e+10	0.02	0.985	0	.
**5th_and_6th_deciles**						
mod_care_4						
standard	.2513724	.1692147	-2.05	0.040	.0671924	.9404055
partial CoC	.1490418	.1026833	-2.76	0.006	.0386249	.575107
ethnicity						
BA	5.558974	4.138049	2.30	0.021	1.292314	23.9123
BC	4.761389	4.20312	1.77	0.077	.843985	26.86164
BO	2.572342	3.305336	0.74	0.462	.2072886	31.92139
M	.9059086	.9060892	-0.10	0.921	.1275596	6.433623
U	2.623069	1.22996	2.06	0.040	1.046359	6.575653
WB	1.151676	.5021643	0.32	0.746	.4899841	2.706942
WO	1.536157	.6463339	1.02	0.308	.6734338	3.504099
WP	1.570376	9622.35	0.00	1.000	0	.
age_cat						
20-24	1.05e-06	.0009586	-0.02	0.988	0	.
25-29	1.12e-06	.0010216	-0.02	0.988	0	.
30-34	6.55e-07	.0005984	-0.02	0.988	0	.
greater than 34	4.74e-07	.0004326	-0.02	0.987	0	.
0.parity	1.061601	.2752384	0.23	0.818	.6386644	1.764615
1.any_risk	.9115363	.3385852	-0.25	0.803	.4401491	1.887766
2.high_risk_num	1.397186	.6138501	0.76	0.446	.5905828	3.305426
place_hosp_comm						
hospital	.6201716	.1818529	-1.63	0.103	.3490713	1.101817
imp_gstt						
imp	2.111408	.6563113	2.40	0.016	1.148112	3.882935
_cons	4769809	4.36e+09	0.02	0.987	0	.
**least_deprived**	(base outcome)					

Table 2: Place of antenatal care recorded by deprivation score

```
Multinomial logistic regression                 Number of obs     =        765
                                                LR chi2(57)       =     153.19
                                                Prob > chi2       =     0.0000
Log likelihood = -948.68392                     Pseudo R2         =     0.0747
```

imd_3_score	RRR	Std. Err.	z	P>|z|	[95% Conf.	Interval]
**most_deprived**						
place_hosp_comm						
hospital	.4068348	.1188187	-3.08	0.002	.2295193	.7211356
ethnicity						
BA	8.688427	6.039025	3.11	0.002	2.224848	33.92985
BC	4.741514	3.985545	1.85	0.064	.91291	24.6267
BO	3.775191	4.386607	1.14	0.253	.3871507	36.81272
M	1.711281	1.391649	0.66	0.509	.3476215	8.424344
U	2.078345	.9214765	1.65	0.099	.8716061	4.955816
WB	.6403515	.2711355	-1.05	0.292	.279258	1.468356
WO	1.002714	.3973854	0.01	0.995	.4611452	2.180302
WP	2.391133	13830.83	0.00	1.000	0	.
age_cat						
20-24	1.37e-06	.0012548	-0.01	0.988	0	.
25-29	1.19e-06	.0010827	-0.01	0.988	0	.
30-34	4.47e-07	.0004081	-0.02	0.987	0	.
greater than 34	2.79e-07	.0002551	-0.02	0.987	0	.
0.parity	.6508242	.1653202	-1.69	0.091	.395589	1.070738
1.any_risk	1.684199	.5775913	1.52	0.129	.8599561	3.298455
2.high_risk_num	2.372965	.9909666	2.07	0.039	1.046704	5.379712
mod_care_4						
standard	.291514	.1930548	-1.86	0.063	.0796072	1.067497
partial CoC	.1270207	.0863271	-3.04	0.002	.0335253	.481257
imp_gstt						
imp	2.764661	.8701099	3.23	0.001	1.491925	5.123148
_cons	1.15e+07	1.05e+10	0.02	0.986	0	.
**3rd_and_4th_deciles**						
place_hosp_comm						
hospital	.4245959	.1120848	-3.25	0.001	.2530904	.7123212
ethnicity						
BA	5.239074	3.616894	2.40	0.016	1.353978	20.27205
BC	2.414152	2.031332	1.05	0.295	.4640247	12.55996
BO	7.223335	7.936432	1.80	0.072	.8385027	62.22588
M	1.297728	1.029289	0.33	0.742	.2741926	6.142027
U	2.248795	.9372376	1.94	0.052	.9935552	5.089884
WB	.689703	.2667646	-0.96	0.337	.3231713	1.471945
WO	1.130883	.4187918	0.33	0.740	.5472657	2.336883
WP	3.09e+07	1.41e+11	0.00	0.997	0	.
age_cat						
20-24	1.58e-06	.001445	-0.01	0.988	0	.
25-29	9.24e-07	.0008435	-0.02	0.988	0	.
30-34	5.30e-07	.0004843	-0.02	0.987	0	.
greater than 34	2.87e-07	.0002617	-0.02	0.987	0	.
0.parity	.66367	.1575855	-1.73	0.084	.4167141	1.056979
1.any_risk	1.480933	.4932381	1.18	0.238	.7709656	2.844695
2.high_risk_num	2.558484	.9732096	2.47	0.014	1.213942	5.392215
mod_care_4						
standard	.2897059	.1877638	-1.91	0.056	.081335	1.031898
partial CoC	.1905813	.1257227	-2.51	0.012	.052307	.6943858
imp_gstt						
imp	1.547321	.4430837	1.52	0.127	.8827429	2.71223
_cons	1.86e+07	1.70e+10	0.02	0.985	0	.
**5th_and_6th_deciles**						
place_hosp_comm						
hospital	.6201716	.1818529	-1.63	0.103	.3490713	1.101817
ethnicity						
BA	5.558974	4.138049	2.30	0.021	1.292314	23.9123
BC	4.761389	4.20312	1.77	0.077	.843985	26.86164
BO	2.572342	3.305336	0.74	0.462	.2072886	31.92139
M	.9059086	.9060892	-0.10	0.921	.1275596	6.433623
U	2.623069	1.22996	2.06	0.040	1.046359	6.575653
WB	1.151676	.5021643	0.32	0.746	.4899841	2.706942
WO	1.536157	.6463339	1.02	0.308	.6734338	3.504099
WP	1.570376	9622.35	0.00	1.000	0	.
age_cat						
20-24	1.05e-06	.0009586	-0.02	0.988	0	.
25-29	1.12e-06	.0010216	-0.02	0.988	0	.
30-34	6.55e-07	.0005984	-0.02	0.988	0	.
greater than 34	4.74e-07	.0004326	-0.02	0.987	0	.
0.parity	1.061601	.2752384	0.23	0.818	.6386644	1.764615
1.any_risk	.9115363	.3385852	-0.25	0.803	.4401491	1.887766
2.high_risk_num	1.397186	.6138501	0.76	0.446	.5905828	3.305426
mod_care_4						
standard	.2513724	.1692147	-2.05	0.040	.0671924	.9404055
partial CoC	.1490418	.1026833	-2.76	0.006	.0386249	.575107
imp_gstt						
imp	2.111408	.6563113	2.40	0.016	1.148112	3.882935
_cons	4769809	4.36e+09	0.02	0.987	0	.
**least_deprived**	(base outcome)					

Table 3: Qualitative quotations relating to women's experiences of referral to the specialist model of care

'I was just sent here. I think it's because of, I have specific needs, but I think he [GP] must have known what he was doing because…this is more like a special care for me rather than just normal midwife care, so I assume he did that knowingly…I do not recall a question, I mean like him asking me if this is what I wanted or not wanted.' (CBM1)
'you always have your own midwife. No I don't think I had any choice, it's just based on your location, you will have your GP, and based on your GP you will have, er, nearby hospital based on your location…I don't think there's any choice out there, it's not given.' (HBM3)
*' I think is because of my vulnerability, the mental health issues that I have…. I was actually quite taken aback that, um, because I didn't have to do anything, I didn't have to chase anyone I didn't have to fight with anyone, it [*the referral*] was just done…so obviously my doctor had done that. There must have been some sort of … I don't know, either procedure on the system or they liaise with each other. But, I thought, thank God, because I felt like it was a moment of euphoria where I myself didn't have to fight. Didn't have to run around. It was just done. (HBM8)*
'I mean the fact that I'm seeing, um, you know [HBM], I don't know if you want to class that as discrimination [laughs], because [midwife name]'s deals with so-called 'vulnerable women'. So you can look at that two ways. I was just referred. I received a letter, um, saying you know, 'You'll be seeing, um, er, a member of the [HBM],' it wasn't kind of like disclosed on the paper you know. It's just something that I kind of gathered myself.' (HBM9)

For peer review only

Analysis 2: Timing of access to maternity services

Table 4: Gestation at booking appointment in relation to the model of care received

Multinomial logistic regression — Number of obs = 764; LR chi2(66) = 118.84; Prob > chi2 = 0.0001; Pseudo R2 = 0.0641

Log likelihood = -867.49667

booking_by_weeks	RRR	Std. Err.	z	P>|z|	[95% Conf.	Interval]
**less_than_10**	(base outcome)					
**10_to_13**						
mod_care_4						
standard	.7668591	.2321091	-0.88	0.380	.4237193	1.387883
partial CoC	.8674172	.2802026	-0.44	0.660	.460536	1.633776
ethnicity						
BA	1.334442	.5151908	0.75	0.455	.6261447	2.843969
BC	.766018	.3445405	-0.59	0.553	.3172375	1.849667
BO	.6923595	.3854517	-0.66	0.509	.232514	2.061646
M	.3655942	.2340446	-1.57	0.116	.1042511	1.282088
U	1.279369	.399387	0.79	0.430	.6938601	2.358955
WB	.9397428	.2956228	-0.20	0.843	.5072702	1.740919
WO	.8957496	.2647394	-0.37	0.710	.5018957	1.598674
WP	5067080	1.20e+10	0.01	0.995	0	.
age_cat						
20-24	1.538972	1.460734	0.45	0.650	.239498	9.889157
25-29	1.753686	1.60286	0.61	0.539	.2923871	10.5183
30-34	1.280188	1.154429	0.27	0.784	.21862	7.496481
greater than 34	1.567427	1.417993	0.50	0.619	.2661565	9.230766
0.parity	.8088913	.1438613	-1.19	0.233	.5708264	1.146242
imd_3_score						
most deprived	.9860824	.2740039	-0.05	0.960	.5719905	1.699956
3rd and 4th deciles	.776939	.2004962	-0.98	0.328	.4685184	1.28839
5th and 6th deciles	.896944	.2518034	-0.39	0.698	.5173727	1.554989
1.any_risk	.7892282	.1783753	-1.05	0.295	.5067813	1.229093
2.high_risk_num	.7087357	.1948708	-1.25	0.211	.4134678	1.214862
place_hosp_comm						
hospital	1.00336	.2024533	0.02	0.987	.6756249	1.490076
imp_gstt						
imp	1.94413	.428977	3.01	0.003	1.261553	2.996022
_cons	.751263	.7661935	-0.28	0.779	.1017827	5.54511
**13_to_20**						
mod_care_4						
standard	.891767	.3978579	-0.26	0.797	.371956	2.138017
partial CoC	.7594591	.3653578	-0.57	0.567	.2958091	1.949832
ethnicity						
BA	1.880764	1.007965	1.18	0.239	.6578797	5.376777
BC	.879995	.6116616	-0.18	0.854	.2253368	3.436594
BO	.6772392	.5960294	-0.44	0.658	.1206731	3.800787
M	.5571623	.5041002	-0.65	0.518	.09459	3.281845
U	1.528332	.7273685	0.89	0.373	.601328	3.884402
WB	1.035115	.5188021	0.07	0.945	.3875826	2.764476
WO	.9787965	.4439433	-0.05	0.962	.4023659	2.381024
WP	.6953945	3549.102	-0.00	1.000	0	.
age_cat						
20-24	.3697443	.350147	-1.05	0.293	.0577851	2.365851
25-29	.3577783	.3203188	-1.15	0.251	.0618776	2.068686
30-34	.1970194	.1730667	-1.85	0.064	.0352202	1.102111
greater than 34	.4102757	.3572851	-1.02	0.306	.0744414	2.261192
0.parity	.7365051	.1987517	-1.13	0.257	.4339826	1.249911
imd_3_score						
most deprived	1.869325	.8781225	1.33	0.183	.7444399	4.693966
3rd and 4th deciles	1.450336	.6606416	0.82	0.414	.5939339	3.541595
5th and 6th deciles	1.924385	.9115327	1.38	0.167	.7605	4.869503
1.any_risk	1.406591	.4298377	1.12	0.264	.7727702	2.560265
2.high_risk_num	.5371098	.2690423	-1.24	0.215	.2012287	1.433628
place_hosp_comm						
hospital	1.05097	.3495657	0.15	0.881	.5476087	2.017022
imp_gstt						
imp	2.732355	.9649809	2.85	0.004	1.367477	5.45952
_cons	.3516654	.4063003	-0.90	0.366	.036533	3.385121
**3**						
mod_care_4						
standard	1.276589	.6689131	0.47	0.641	.4571242	3.565067
partial CoC	1.154034	.644423	0.26	0.798	.3862778	3.447762
ethnicity						
BA	2.23247	1.209553	1.48	0.138	.7719772	6.45605
BC	.3917793	.3365533	-1.09	0.275	.072749	2.109872
BO	6.47e-07	.0004433	-0.02	0.983	0	.
M	.3680627	.4184516	-0.88	0.379	.039645	3.417083
U	1.183814	.5736468	0.35	0.728	.4579438	3.060233
WB	.7463861	.37587	-0.58	0.561	.2781697	2.002706
WO	.8127787	.3744247	-0.45	0.653	.3294938	2.004922
WP	.7171062	3822.002	-0.00	1.000	0	.
age_cat						
20-24	2.274215	2.940942	0.64	0.525	.1803371	28.67993
25-29	1.210863	1.550069	0.15	0.881	.0985013	14.88499
30-34	1.303381	1.621172	0.21	0.831	.1138492	14.92152
greater than 34	1.273376	1.59059	0.19	0.847	.1100805	14.73002
0.parity	1.794035	.509178	2.06	0.039	1.028597	3.129079
imd_3_score						
most deprived	.9455481	.400684	-0.13	0.895	.4120783	2.169639
3rd and 4th deciles	.7027879	.2798035	-0.89	0.376	.3220567	1.533615
5th and 6th deciles	.7430273	.3252906	-0.68	0.497	.3150333	1.75248
1.any_risk	1.938461	.6191795	2.07	0.038	1.036497	3.625319
2.high_risk_num	2.498193	.9487996	2.41	0.016	1.18671	5.259053
place_hosp_comm						
hospital	2.510521	.8052141	2.87	0.004	1.338915	4.707331
imp_gstt						
imp	1.328284	.4706625	0.80	0.423	.6632503	2.660141
_cons	.0502035	.0742011	-2.02	0.043	.0027711	.9095269

Table 5: Gestation at booking appointment in relation to the place of antenatal care

Multinomial logistic regression — Number of obs = 764
LR chi2(66) = 118.84
Prob > chi2 = 0.0001
Log likelihood = -867.49667 — Pseudo R2 = 0.0641

booking_by_weeks	RRR	Std. Err.	z	P>|z|	[95% Conf. Interval]	
**less_than_10**	(base outcome)					
**10_to_13**						
place_hosp_comm						
hospital	1.00336	.2024533	0.02	0.987	.6756249	1.490076
ethnicity						
BA	1.334442	.5151908	0.75	0.455	.6261447	2.843969
BC	.766018	.3445405	-0.59	0.553	.3172375	1.849667
BO	.6923595	.3854517	-0.66	0.509	.232514	2.061646
M	.3655942	.2340446	-1.57	0.116	.1042511	1.282088
U	1.279369	.399387	0.79	0.430	.6938601	2.358955
WB	.9397428	.2956228	-0.20	0.843	.5072702	1.740919
WO	.8957496	.2647394	-0.37	0.710	.5018957	1.598674
WP	5067080	1.20e+10	0.01	0.995	0	.
age_cat						
20-24	1.538972	1.460734	0.45	0.650	.239498	9.889157
25-29	1.753686	1.60286	0.61	0.539	.2923871	10.5183
30-34	1.280188	1.154429	0.27	0.784	.21862	7.496481
greater than 34	1.567427	1.417993	0.50	0.619	.2661565	9.230766
1.parity	1.23626	.2198688	1.19	0.233	.8724162	1.751846
imd_3_score						
3rd and 4th deciles	.7879048	.1724215	-1.09	0.276	.5130983	1.209893
5th and 6th deciles	.9096035	.2323215	-0.37	0.711	.5513749	1.500573
least deprived	1.014114	.2817931	0.05	0.960	.5882506	1.748281
1.any_risk	.7892282	.1783753	-1.05	0.295	.5067813	1.229093
2.high_risk_num	.7087357	.1948708	-1.25	0.211	.4134678	1.214862
mod_care_4						
standard	.7668591	.2321091	-0.88	0.380	.4237193	1.387883
partial CoC	.8674172	.2802026	-0.44	0.660	.460536	1.633776
imp_gstt						
imp	1.94413	.428977	3.01	0.003	1.261553	2.996022
_cons	.5992325	.5848482	-0.52	0.600	.0884762	4.058488
**13_to_20**						
place_hosp_comm						
hospital	1.05097	.3495657	0.15	0.881	.5476087	2.017022
ethnicity						
BA	1.880764	1.007965	1.18	0.239	.6578797	5.376777
BC	.879995	.6116616	-0.18	0.854	.2253368	3.436594
BO	.6772392	.5960294	-0.44	0.658	.1206731	3.800787
M	.5571623	.5041002	-0.65	0.518	.09459	3.281845
U	1.528332	.7273685	0.89	0.373	.601328	3.884402
WB	1.035115	.5188021	0.07	0.945	.3875826	2.764476
WO	.9787965	.4439433	-0.05	0.962	.4023659	2.381024
WP	.6953945	3549.102	-0.00	1.000	0	.
age_cat						
20-24	.3697443	.350147	-1.05	0.293	.0577851	2.365851
25-29	.3577783	.3203188	-1.15	0.251	.0618776	2.068686
30-34	.1970194	.1730667	-1.85	0.064	.0352202	1.102111
greater than 34	.4102757	.3572851	-1.02	0.306	.0744414	2.261192
1.parity	1.357764	.3664033	1.13	0.257	.8000567	2.30424
imd_3_score						
3rd and 4th deciles	.7758606	.2442081	-0.81	0.420	.41866	1.437825
5th and 6th deciles	1.029455	.3671188	0.08	0.935	.5117479	2.070896
least deprived	.5349525	.251296	-1.33	0.183	.2130395	1.343292
1.any_risk	1.406591	.4298377	1.12	0.264	.7727702	2.560265
2.high_risk_num	.5371098	.2690423	-1.24	0.215	.2012287	1.433628
mod_care_4						
standard	.891767	.3978579	-0.26	0.797	.371956	2.138017
partial CoC	.7594591	.3653578	-0.57	0.567	.2958091	1.949832
imp_gstt						
imp	2.732355	.9649809	2.85	0.004	1.367477	5.45952
_cons	.4841614	.5020639	-0.70	0.484	.0634329	3.695438
**3**						
place_hosp_comm						
hospital	2.510521	.8052141	2.87	0.004	1.338915	4.707331
ethnicity						
BA	2.23247	1.209553	1.48	0.138	.7719772	6.45605
BC	.3917793	.3365533	-1.09	0.275	.072749	2.109872
BO	6.47e-07	.0004433	-0.02	0.983	0	.
M	.3680627	.4184516	-0.88	0.379	.039645	3.417083
U	1.183814	.5736468	0.35	0.728	.4579438	3.060233
WB	.7463861	.37587	-0.58	0.561	.2781697	2.002706
WO	.8127787	.3744247	-0.45	0.653	.3294938	2.004922
WP	.7171062	3822.002	-0.00	1.000	0	.
age_cat						
20-24	2.274215	2.940942	0.64	0.525	.1803371	28.67993
25-29	1.210863	1.550069	0.15	0.881	.0985013	14.88499
30-34	1.303381	1.621172	0.21	0.831	.1138492	14.92152
greater than 34	1.273376	1.59059	0.19	0.847	.1100805	14.73002
1.parity	.5574027	.1582005	-2.06	0.039	.3195828	.9721981
imd_3_score						
3rd and 4th deciles	.7432598	.2534893	-0.87	0.384	.3809235	1.450252
5th and 6th deciles	.7858165	.3181927	-0.60	0.552	.3553471	1.737759
least deprived	1.057588	.4481618	0.13	0.895	.4609062	2.426723
1.any_risk	1.938461	.6191795	2.07	0.038	1.036497	3.625319
2.high_risk_num	2.498193	.9487996	2.41	0.016	1.18671	5.259053
mod_care_4						
standard	1.276589	.6689131	0.47	0.641	.4571242	3.565067
partial CoC	1.154034	.644423	0.26	0.798	.3862778	3.447762
imp_gstt						
imp	1.328284	.4706625	0.80	0.423	.6632503	2.660141
_cons	.0851625	.1209387	-1.73	0.083	.0052658	1.377316

Table 6: Qualitative quotations relating to women's experiences of accessing maternity care

'*the main take-away that I have from the whole experience is…the first ten weeks. That you don't have any support from anyone until you have your booking appointment. Like even information on who to call, someone to talk to' (CBM1)*
Husband- *In the beginning she was unhappy because she was telling me like, no one is caring about her…first three months.* Woman - *Yeah, there is no one.* Husband- *there is no services, nothing. No one called her. She didn't do a scan…Before 20 weeks, it's different….you feel like they're not ready for someone who is pregnant before 20 weeks. (CBM6)*
'they [maternity services] weren't really too bothered, it was like, 'well, it's not really important till the 12 weeks and that's when we can start doing what we need to do'.'(HBM9)
'Not easy. You near enough have to be dying to get a [GP] appointment. It is ridiculous to get an appointment there. Sometimes it's just not even worth trying to ring up to make an appointment … it's very hard work. [HBM9)
I couldn't get a GP appointment because I'm still registered at my old GP, I went to my local walk-in centre and they couldn't see me because I was pregnant… So I saw a couple of people at the, is it the maternity urgent care centre there? (CBM9)
'And then they [GP based at community health centre] send me to the [CBM] midwife, they send me a letter to contact the midwife…Very easy.' (CMB3)
'I went to the GP. Um, it was horrible…they gave me a very long [late] appointment like up to one month, so all these things were not done, like I wouldn't have done my scan or anything. Yeah, and then I talked to someone, they said about self-referral. And then I self-referred myself, and then I saw the midwife at around 14 weeks.' (HBM5)
This woman, who had no recourse to public funds, described her experience of accessing standard, hospital-based maternity care in a previous pregnancy, and then her experience of accessing the hospital-based specialist model in her current pregnancy.
Previous pregnancy under standard hospital care: '*We went to the GP when I was about six weeks pregnant, and then we couldn't get [maternity] appointment until I was about four months. So what I did was we went to private*

hospital to do the three-months checks … the blood test and the scan checks. And then the first appointment I had here [at hospital] was when I was four months pregnant with a scan…I don't know maybe within GP to the hospital or the process maybe took long, but I remember phoning them and then asking them, 'When can I get an appointment and see a midwife?' and they said, 'No you can't see at the moment…there's no ready documentation and everything's not ready,' so and then we went to see private hospital. (HBM3)

This pregnancy under specialist model: *I went to GP and they confirmed I was pregnant. And then I received call, or text, I can't remember maybe both, from [HBM midwife], saying that, 'I'm your midwife, can you come and see me on this day?'…Yes it was straightaway, easy process. (HBM3)*

For peer review only

Analysis 3: Engagement - Number of antenatal appointments attended

Table 7: Number of antenatal appointments attended in relation to the model of care accessed

1_6	(base outcome)					
7_9						
mod_care_4						
standard	1.149272	.39513	0.40	0.686	.5858305	2.254621
partial CoC	1.812476	.6855162	1.57	0.116	.863635	3.803772
ethnicity						
BA	.5885425	.2427223	-1.29	0.199	.2622598	1.32076
BC	1.099803	.5676191	0.18	0.854	.3999463	3.024324
BO	4.060241	4.736953	1.20	0.230	.4125555	39.95962
M	1.171773	.7740172	0.24	0.810	.3210558	4.276678
U	.759139	.2641498	-0.79	0.428	.3838287	1.50143
WB	.5519485	.1999764	-1.64	0.101	.2713313	1.122787
WO	.7548208	.2487774	-0.85	0.393	.3956406	1.440081
WP	2.74e+10	5.45e+15	0.00	1.000	0	.
age_cat						
20-24	1.483199	1.397323	0.42	0.676	.2340356	9.399771
25-29	1.624311	1.465304	0.54	0.591	.2772002	9.517979
30-34	1.808432	1.601011	0.67	0.503	.3189559	10.25354
greater than 34	1.769831	1.573776	0.64	0.521	.3097586	10.11208
0.parity	.8018499	.1583715	-1.12	0.264	.5444704	1.180897
imd_3_score						
most deprived	.9755324	.2950882	-0.08	0.935	.5392154	1.764904
3rd and 4th deciles	.900557	.2580345	-0.37	0.715	.5135923	1.579079
5th and 6th deciles	1.030453	.3231764	0.10	0.924	.5572748	1.905402
1.any_risk	.7663696	.1894805	-1.08	0.282	.4720452	1.244208
2.high_risk_num	1.527345	.5028294	1.29	0.198	.8011361	2.911843
place_hosp_comm						
hospital	1.610698	.3915808	1.96	0.050	1.000176	2.593892
imp_gstt						
imp	1.778247	.4527273	2.26	0.024	1.07965	2.928877
_cons	.3449901	.3564553	-1.03	0.303	.045532	2.613946
10_14						
mod_care_4						
standard	.9440856	.3450886	-0.16	0.875	.4611841	1.932629
partial CoC	1.765937	.7013047	1.43	0.152	.8108462	3.846024
ethnicity						
BA	.7315004	.3275001	-0.70	0.485	.3041747	1.759163
BC	.9096089	.5294628	-0.16	0.871	.2906628	2.846557
BO	11.42446	12.8353	2.17	0.030	1.263331	103.3129
M	1.689606	1.169752	0.76	0.449	.43499	6.562836
U	.8829985	.3416791	-0.32	0.748	.4136041	1.885103
WB	.922301	.3626736	-0.21	0.837	.426734	1.993371
WO	.8191682	.3035382	-0.54	0.590	.3962462	1.693484
WP	.6818543	213248.4	-0.00	1.000	0	.
age_cat						
20-24	4.078474	4.889285	1.17	0.241	.3891123	42.74845
25-29	3.71801	4.365364	1.12	0.263	.3723091	37.12935
30-34	3.286795	3.815627	1.02	0.305	.3377661	31.98373
greater than 34	3.223814	3.749286	1.01	0.314	.3299325	31.50031
0.parity	.7469259	.1584339	-1.38	0.169	.492862	1.131957
imd_3_score						
most deprived	1.3555	.4647616	0.89	0.375	.6922249	2.654311
3rd and 4th deciles	1.424572	.4613422	1.09	0.275	.7551377	2.687465
5th and 6th deciles	1.681039	.5879565	1.49	0.138	.8469609	3.336508
1.any_risk	.9725235	.2515335	-0.11	0.914	.5857946	1.614562
2.high_risk_num	2.733911	.8926909	3.08	0.002	1.441603	5.184694
place_hosp_comm						
hospital	4.356843	1.119242	5.73	0.000	2.633324	7.208409
imp_gstt						
imp	1.158933	.3096098	0.55	0.581	.6865297	1.956399
_cons	.0613308	.0796822	-2.15	0.032	.004806	.7826666
15						
mod_care_4						
standard	.4947072	.2332588	-1.49	0.136	.1963356	1.246514
partial CoC	2.32004	1.123352	1.74	0.082	.8981479	5.992984
ethnicity						
BA	.3915834	.2406746	-1.53	0.127	.1173977	1.306137
BC	1.330121	.8859924	0.43	0.668	.3605009	4.907675
BO	14.03244	16.56205	2.24	0.025	1.388309	141.8339
M	.3621583	.4306236	-0.85	0.393	.0352192	3.724071
U	.4462132	.2238845	-1.61	0.108	.1669005	1.192963
WB	.4952449	.2435218	-1.43	0.153	.1889154	1.298293
WO	.3881658	.1850012	-1.99	0.047	.1525217	.9878774
WP	.6593021	273021.6	-0.00	1.000	0	.
age_cat						
20-24	.4101014	.4703021	-0.78	0.437	.0433249	3.881902
25-29	1.199888	1.214551	0.18	0.857	.1650205	8.724565
30-34	.9813159	.9625507	-0.02	0.985	.1435107	6.710166
greater than 34	1.388501	1.361578	0.33	0.738	.203165	9.489499
0.parity	1.135431	.3389281	0.43	0.670	.6325219	2.038196
imd_3_score						
most deprived	.8194501	.3920441	-0.42	0.677	.3208395	2.092942
3rd and 4th deciles	1.249745	.5321265	0.52	0.601	.5424875	2.879074
5th and 6th deciles	1.721278	.7826422	1.19	0.232	.7060268	4.19644
1.any_risk	1.971849	.670567	2.00	0.046	1.012524	3.840095
2.high_risk_num	4.129237	1.634349	3.58	0.000	1.900924	8.969637
place_hosp_comm						
hospital	7.886996	2.707958	6.01	0.000	4.023983	15.45849
imp_gstt						
imp	.5237601	.1927356	-1.76	0.079	.2546264	1.077361
_cons	.1135639	.1406873	-1.76	0.079	.010017	1.287483

Table 8: Number of antenatal appointments attended in relation to the place of antenatal care

ante_appt_cat	RRR	Std. Err.	z	P>|z|	[95% Conf.	Interval]
**1_6**						
place_hosp_comm						
hospital	.6193839	.1505954	-1.97	0.049	.3845926	.9975136
ethnicity						
BA	1.693631	.6982336	1.28	0.201	.7549079	3.799652
BC	.9099711	.469674	-0.18	0.855	.3308926	2.502466
BO	.2459387	.2869793	-1.20	0.229	.0249794	2.421427
M	.8530948	.5635031	-0.24	0.810	.2337463	3.113507
U	1.317993	.4586435	0.79	0.428	.6663566	2.606871
WB	1.817802	.658774	1.65	0.099	.8934492	3.698479
WO	1.325525	.4369186	0.85	0.393	.6947288	2.529067
WP	5.47e-06	.0028133	-0.02	0.981	0	.
age_cat						
20-24	.6736911	.6347487	-0.42	0.675	.1062827	4.270307
25-29	.6160015	.5557416	-0.54	0.591	.1051111	3.610066
30-34	.553214	.4898026	-0.67	0.504	.0975572	3.13709
greater than 34	.5657324	.5031052	-0.64	0.522	.0990007	3.232836
1.parity	.8019261	.1583226	-1.12	0.264	.5446073	1.180824
imd_3_score						
3rd and 4th deciles	1.0851	.2609095	0.34	0.734	.6773299	1.738357
5th and 6th deciles	.942631	.2663404	-0.21	0.834	.5417946	1.640019
least deprived	.9741613	.2946691	-0.09	0.931	.5384622	1.762408
1.any_risk	1.305822	.3228398	1.08	0.280	.8043415	2.119959
2.high_risk_num	.6512724	.2144145	-1.30	0.193	.3416069	1.241648
imp_gstt						
imp	.5628822	.1432485	-2.26	0.024	.3418175	.9269167
mod_care_4						
standard	.8709317	.2994596	-0.40	0.688	.4439239	1.708676
partial CoC	.551143	.2084863	-1.57	0.115	.2625861	1.156796
_cons	3.705105	3.624279	1.34	0.181	.5447133	25.20189
**7_9**	(base outcome)					
**10_14**						
place_hosp_comm						
hospital	2.706802	.7010945	3.84	0.000	1.629239	4.497053
ethnicity						
BA	1.250261	.5609235	0.50	0.619	.5189353	3.01223
BC	.8264228	.4639844	-0.34	0.734	.2749832	2.483696
BO	2.816899	1.91737	1.52	0.128	.7419706	10.69439
M	1.443782	.9667798	0.55	0.583	.3886181	5.363892
U	1.162341	.4419722	0.40	0.692	.5516585	2.449047
WB	1.664648	.6550648	1.30	0.195	.7697707	3.599842
WO	1.084449	.3876004	0.23	0.821	.5382388	2.184957
WP	3.74e-06	.0023281	-0.02	0.984	0	.
age_cat						
20-24	2.750417	3.611257	0.77	0.441	.2097893	36.059
25-29	2.286663	2.944347	0.64	0.521	.1833074	28.5249
30-34	1.815692	2.31376	0.47	0.640	.1493981	22.0668
greater than 34	1.819661	2.322906	0.47	0.639	.1490666	22.21266
1.parity	1.072536	.2310358	0.33	0.745	.7031594	1.635949
imd_3_score						
3rd and 4th deciles	1.137171	.2975716	0.49	0.623	.6809041	1.899178
5th and 6th deciles	1.179217	.3566926	0.54	0.586	.651809	2.133376
least deprived	.720963	.2481584	-0.95	0.342	.3672196	1.415468
1.any_risk	1.26802	.3372343	0.89	0.372	.7529157	2.135532
2.high_risk_num	1.801903	.6252472	1.70	0.090	.9127898	3.557068
imp_gstt						
imp	.6524645	.1786534	-1.56	0.119	.381493	1.115905
mod_care_4						
standard	.8213091	.3325921	-0.49	0.627	.3713723	1.816368
partial CoC	.9744801	.4157976	-0.06	0.952	.4222568	2.248895
_cons	.2299289	.3119432	-1.08	0.279	.0160981	3.28408
**15**						
place_hosp_comm						
hospital	4.907966	1.683372	4.64	0.000	2.50582	9.612873
ethnicity						
BA	.6804993	.4201494	-0.62	0.533	.2029011	2.282291
BC	1.210251	.7847556	0.29	0.769	.3395752	4.313351
BO	3.471839	2.712416	1.59	0.111	.7508414	16.05354
M	.3102073	.3671998	-0.99	0.323	.0304835	3.156746
U	.5870266	.2926949	-1.07	0.285	.2209244	1.559811
WB	.8915576	.4378813	-0.23	0.815	.3404777	2.334587
WO	.5134041	.2397176	-1.43	0.153	.2055976	1.282037
WP	3.62e-06	.0033946	-0.01	0.989	0	.
age_cat						
20-24	.2764452	.3510455	-1.01	0.311	.0229459	3.330521
25-29	.7364513	.8450313	-0.27	0.790	.0777041	6.979816
30-34	.5412187	.6054519	-0.55	0.583	.0604155	4.848384
greater than 34	.7835655	.876771	-0.22	0.827	.0874225	7.02308
1.parity	.7035639	.211548	-1.17	0.242	.3902687	1.268362
imd_3_score						
3rd and 4th deciles	1.649296	.6300238	1.31	0.190	.7800879	3.487016
5th and 6th deciles	2.010318	.8698524	1.61	0.107	.8609046	4.69434
least deprived	1.195039	.5724279	0.37	0.710	.4673623	3.055699
1.any_risk	2.56915	.8901076	2.72	0.006	1.302813	5.066368
2.high_risk_num	2.738223	1.123561	2.45	0.014	1.22518	6.119809
imp_gstt						
imp	.2950832	.1098794	-3.28	0.001	.1422278	.6122156
mod_care_4						
standard	.4299578	.2149768	-1.69	0.091	.1613724	1.145572
partial CoC	1.278626	.6479404	0.49	0.628	.4735869	3.452134
_cons	.3905271	.5050302	-0.73	0.467	.0309654	4.925227

Table 9: Qualitative quotations relating to women's experiences of engaging with the specialist model of care

'I will book my appointment with midwife through text messages like you're texting a friend, and then it's so informal but also very efficient…. sometimes I can't make the appointment, or my midwife can't make the appointment, then we text each other beforehand' (HBM3)
'the absolute amazing thing about the [specialist model] is that I had a phone number I could call…by the end of the first week pretty much knew all of them on a first name basis. Whoever would answer the phone was reassuring, they were able to talk me through things… if we didn't have the like phone number that we could call the first few weeks it would have been a lot worse, a lot more difficult. We would have ended up in A&E a lot more often than we did… which is zero…it's just like every concern, it's kind of like calling a friend…regardless of which midwife was picking up at the time' (Partner of CBM6)
*'the fact that I see someone regularly. I feel like I'm being looked after as well… I can rely on them to look after me, remind me of appointments and stuff like that as I really struggle … [the midwives] text, call, put it in my notes and what-not so …I am remembering … or I do actually go to these appointments… whereas my other midwife appointments [*under standard care*] were just the normal basic appointments…I was visiting hospital more [because] when I did try to get in contact with someone it was impossible, so I just had to keep running to the hospital.'(CBM1)*
'I'll tell [CBM midwife] a convenient time slot for me. Last week it was at home. Sometimes if my husband is not there, it's better to be at home, because travelling with the kids is not easy.' (CBM2)
'it's been very flexible. Um, they have located a lot of time with my appointments because of my circumstances so … that's really really really helpful particularly with the midwives, it is easy.' (CMB5)
'at one point I was a bit worried, so I emailed them, they called me back, they got me in sort of within the day to do some blood tests' (CBM6)
[concerned she had a urine infection] I called up and, again because I've not had like any really serious problems there's always that hesitation like I don't want to bother anyone, but the team are so lovely they're like, 'You can literally just call us to chat.' and she was like 'Someone is in clinic…you could come in today between these hours and also tomorrow'… I literally got a sample, knocked on the door, gave them that, took a seat for five minutes…and when they had a gap came out and just chatted to me.' (CBM9)

For peer review only

'[an appointment] every two weeks…It's too much. Yeah, and um, after the baby's due date they started every week! It was too much. No medical reason, I think it's a requirement… I prefer, um, when I want to see them, like maybe every three weeks. And then if I need to see them then maybe I can call them and then make appointment before three weeks.' (HBM3)

I'm struggling a little bit. Just I think more, I'm feeling a bit low and irritable. I feel a bit, this week I do feel a bit emotional…it's just me, being on my own. I haven't seen [HBM midwife] in probably about two weeks, um, the last time I had an appointment was with a different midwife because she was on holiday. I was meant to see her today but she, I think she's not feeling very well….how I am feeling now I think more [appointments] would be good. I think [antenatal care] follows a schedule. (HBM2)

For peer review only

Analysis 4: The quality of relational continuity and missed appointments

Table 10: Number of appointments and support in labour by known healthcare professional

noofapptswithknown~p	RRR	Std. Err.	z	P>|z|	[95% Conf.	Interval]
**0**	(base outcome)					
**2**						
mod_care_4						
standard	2.12497	1.42054	1.13	0.260	.5732255	7.877349
partial CoC	1.814651	1.307945	0.83	0.408	.4418483	7.452689
ethnicity						
BA	.6897641	.3674707	-0.70	0.486	.2427864	1.959642
BC	1.740153	1.212201	0.80	0.426	.4442582	6.816153
BO	1.674624	1.297217	0.67	0.506	.3669016	7.643372
M	3.514697	3.072361	1.44	0.150	.6335969	19.49678
U	1.185132	.5570245	0.36	0.718	.4717297	2.977422
WB	1.151437	.5773619	0.28	0.779	.4309476	3.076492
WO	1.175301	.507662	0.37	0.708	.5040567	2.740431
WP	2.09e+07	1.20e+11	0.00	0.998	0	.
age_cat						
20-24	2087928	2.43e+09	0.01	0.990	0	.
25-29	5006335	5.83e+09	0.01	0.989	0	.
30-34	5239593	6.10e+09	0.01	0.989	0	.
greater than 34	3618132	4.21e+09	0.01	0.990	0	.
0.parity	1.822294	.481216	2.27	0.023	1.086026	3.057712
imd_3_score						
most deprived	.9973948	.4150319	-0.01	0.995	.4412336	2.25458
3rd and 4th deciles	1.119732	.438289	0.29	0.773	.5199173	2.411538
5th and 6th deciles	1.117686	.4667524	0.27	0.790	.4930066	2.533883
1.any_risk	.8629611	.2652524	-0.48	0.632	.4724473	1.576264
2.high_risk_num	1.664787	1.116654	0.76	0.447	.4471117	6.198709
highriskatbirth						
Y	.8888816	.2524653	-0.41	0.678	.5094256	1.550983
place_hosp_comm						
hospital	.2463731	.11066	-3.12	0.002	.1021577	.5941768
imp_gstt						
imp	130.1612	68.8159	9.21	0.000	46.17993	366.8679
_cons	3.50e-09	4.07e-06	-0.02	0.987	0	.
**3**						
mod_care_4						
standard	.3452801	.1990772	-1.84	0.065	.1115313	1.068923
partial CoC	.8252684	.5081991	-0.31	0.755	.2468462	2.759078
ethnicity						
BA	.4432498	.2848524	-1.27	0.205	.1257847	1.561957
BC	.3192784	.3801943	-0.96	0.338	.0309432	3.294382
BO	.5940215	.7219469	-0.43	0.668	.0548643	6.431527
M	2.919087	2.948978	1.06	0.289	.4030225	21.1429
U	.9494037	.5401176	-0.09	0.927	.3113155	2.89535
WB	1.232423	.7186215	0.36	0.720	.393031	3.864492
WO	1.116556	.5604128	0.22	0.826	.4174958	2.98613
WP	.2176232	3289.857	-0.00	1.000	0	.
age_cat						
20-24	4070954	6.23e+09	0.01	0.992	0	.
25-29	1.60e+07	2.45e+10	0.01	0.991	0	.
30-34	8761015	1.34e+10	0.01	0.992	0	.
greater than 34	1.37e+07	2.10e+10	0.01	0.991	0	.
0.parity	1.645874	.5272247	1.56	0.120	.878477	3.083635
imd_3_score						
most deprived	2.243444	1.183107	1.53	0.125	.7980385	6.306765
3rd and 4th deciles	1.962508	.9973083	1.33	0.185	.724849	5.313436
5th and 6th deciles	1.874744	1.010673	1.17	0.244	.651718	5.392923
1.any_risk	1.249665	.4504208	0.62	0.536	.6165841	2.532765
2.high_risk_num	2.051514	1.692983	0.87	0.384	.4070358	10.3399
highriskatbirth						
Y	.9102559	.3097016	-0.28	0.782	.4672554	1.773261
place_hosp_comm						
hospital	.232326	.1178467	-2.88	0.004	.0859663	.6278666
imp_gstt						
imp	158.5037	106.957	7.51	0.000	42.23336	594.8719
_cons	1.50e-09	2.29e-06	-0.01	0.989	0	.
**4**						
mod_care_4						
standard	.1071422	.087908	-2.72	0.006	.0214569	.5350006
partial CoC	1.723088	1.290399	0.73	0.467	.3970573	7.47759
ethnicity						
BA	.303219	.2529282	-1.43	0.153	.0591197	1.55518
BC	1.391403	1.353061	0.34	0.734	.2068748	9.358329
BO	1.017385	1.394737	0.01	0.990	.0692739	14.94173
M	3.92e-06	.0041128	-0.01	0.991	0	.
U	1.080812	.7921639	0.11	0.916	.256966	4.545951
WB	1.750317	1.226933	0.80	0.425	.4430405	6.914965
WO	.6510023	.4259005	-0.66	0.512	.1805956	2.346702
WP	.8365924	16015.77	-0.00	1.000	0	.
age_cat						
20-24	1.13e+07	2.01e+10	0.01	0.993	0	.
25-29	9275790	1.65e+10	0.01	0.993	0	.
30-34	1.53e+07	2.72e+10	0.01	0.993	0	.
greater than 34	7097596	1.26e+10	0.01	0.993	0	.
0.parity	1.553959	.6429303	1.07	0.287	.6906625	3.496335
imd_3_score						
most deprived	2.797857	1.835835	1.57	0.117	.7732198	10.1239
3rd and 4th deciles	1.791035	1.137307	0.92	0.359	.5159303	6.217517
5th and 6th deciles	1.423641	.9797758	0.51	0.608	.3694775	5.485459
1.any_risk	.9706877	.4723698	-0.06	0.951	.3739863	2.519436
2.high_risk_num	5.072918	6.282963	1.31	0.190	.4477329	57.47735
highriskatbirth						
Y	1.834165	.8026277	1.39	0.166	.777954	4.32437
place_hosp_comm						
hospital	.306421	.1568288	-2.31	0.021	.1123742	.8355462
imp_gstt						
imp	293.5531	331.2947	5.03	0.000	32.14013	2681.178
_cons	3.35e-10	5.95e-07	-0.01	0.990	0	.
**5**						
mod_care_4						
standard	.0254159	.019587	-4.77	0.000	.005612	.1151047
partial CoC	.8291245	.5360572	-0.29	0.772	.2335012	2.944086
ethnicity						
BA	.1758425	.1385612	-2.21	0.027	.037531	.8238682
BC	.5001275	.5111954	-0.68	0.498	.0674593	3.707827
BO	1.5265	1.869733	0.35	0.730	.1383895	16.838
M	2.457489	3.421256	0.65	0.518	.1604981	37.6282
U	.8435105	.6012533	-0.24	0.811	.2086195	3.410562
WB	1.151793	.7796169	0.21	0.835	.3056468	4.340395
WO	.4221212	.2640412	-1.38	0.168	.1238801	1.438376
WP	1.229522	22017.75	0.00	1.000	0	.
age_cat						
20-24	2.161349	3.465587	0.48	0.631	.093299	50.06944
25-29	3.170843	5.019867	0.73	0.466	.1424326	70.58951
30-34	3.298027	5.106225	0.77	0.441	.1586235	68.57108
greater than 34	4.282206	6.658542	0.94	0.350	.2032856	90.20458
0.parity	.8574591	.3721807	-0.35	0.723	.3662257	2.007604
imd_3_score						
most deprived	3.896169	2.551882	2.08	0.038	1.079256	14.06537
3rd and 4th deciles	1.732054	1.141455	0.83	0.405	.475997	6.302582
5th and 6th deciles	1.696077	1.193329	0.75	0.453	.4271259	6.734965
1.any_risk	1.753116	.8077984	1.22	0.223	.7105512	4.325399
2.high_risk_num	3.22637	4.079044	0.93	0.354	.2707415	38.44798
highriskatbirth						
Y	2.248574	.9914991	1.84	0.066	.9474877	5.336308
place_hosp_comm						
hospital	.3523471	.1807112	-2.03	0.042	.128946	.9627944
imp_gstt						
imp	366.4902	422.7924	5.12	0.000	38.20274	3515.848
_cons	.0019683	.0042857	-2.86	0.004	.0000276	.1404198

Table 11: Number of appointments with a known healthcare professional in relation to place of care

deliver_named_mw	RRR	Std. Err.	z	P>|z|	[95% Conf.	Interval]
**yes**						
place_hosp_comm						
hospital	.8999399	.1918592	-0.49	0.621	.5925771	1.366728
ethnicity						
BA	1.107203	.4571936	0.25	0.805	.4928825	2.487202
BC	.8993323	.4435732	-0.22	0.830	.3420474	2.36458
BO	1.875314	1.157879	1.02	0.309	.5591325	6.289745
M	1.222475	.8163165	0.30	0.764	.3302511	4.525181
U	1.193273	.4079359	0.52	0.605	.6105841	2.33203
WB	1.595939	.5570766	1.34	0.181	.8051871	3.163266
WO	.8055546	.2648301	-0.66	0.511	.42292	1.534376
WP	.0000219	.0132068	-0.02	0.986	0	.
age_cat						
20-24	.094338	.0939308	-2.37	0.018	.0134017	.6640707
25-29	.1635071	.157408	-1.88	0.060	.0247791	1.078915
30-34	.1665161	.1580722	-1.89	0.059	.0259071	1.070272
greater than 34	.1411916	.1340099	-2.06	0.039	.0219737	.9072226
1.parity	1.07912	.212864	0.39	0.699	.7331014	1.588456
imd_3_score						
3rd and 4th deciles	.9407191	.2249157	-0.26	0.798	.5887716	1.503049
5th and 6th deciles	.8024294	.2263074	-0.78	0.435	.4616835	1.394663
least deprived	1.089825	.3345874	0.28	0.779	.5970758	1.989227
1.any_risk	1.142474	.2769763	0.55	0.583	.7103706	1.837418
2.high_risk_num	1.267832	.4231819	0.71	0.477	.6590892	2.438816
highriskatbirth						
Y	.4433954	.1015607	-3.55	0.000	.2830229	.6946416
mod_care_4						
standard	.5923736	.206196	-1.50	0.133	.2994372	1.171887
partial CoC	.4508229	.1653001	-2.17	0.030	.2197363	.9249327
imp_gstt						
imp	.0891558	.0219513	-9.82	0.000	.0550266	.1444528
_cons	44.86345	46.44374	3.67	0.000	5.898045	341.2536
**no**	(base outcome)					

For peer review only

noofapptswithknown~p	RRR	Std. Err.	z	P>|z|	[95% Conf.	Interval]
**0**	(base outcome)					
**2**						
place_hosp_comm						
hospital	.2467317	.1107829	−3.12	0.002	.1023374	.5948611
ethnicity						
BA	.6924964	.3690025	−0.69	0.490	.2436956	1.967829
BC	1.738724	1.210712	0.79	0.427	.4441403	6.806772
BO	1.67289	1.295196	0.66	0.506	.3668126	7.6294
M	3.508557	3.064964	1.44	0.151	.6332072	19.44067
U	1.182606	.5555711	0.36	0.721	.4709314	2.969765
WB	1.160279	.5821279	0.30	0.767	.4340132	3.101858
WO	1.174625	.5071569	0.37	0.709	.503946	2.737881
WP	2.05e+07	1.16e+11	0.00	0.998	0	.
age_cat						
20−24	2071967	2.40e+09	0.01	0.990	0	.
25−29	4946104	5.74e+09	0.01	0.989	0	.
30−34	5173676	6.00e+09	0.01	0.989	0	.
greater than 34	3587930	4.16e+09	0.01	0.990	0	.
1.parity	.5471574	.1444873	−2.28	0.022	.3260891	.9180962
imd_3_score						
3rd and 4th deciles	1.127121	.3644407	0.37	0.711	.5980605	2.124203
5th and 6th deciles	1.121483	.4141216	0.31	0.756	.5438459	2.312648
least deprived	1.000974	.4163176	0.00	0.998	.4429937	2.261769
1.any_risk	.8623928	.2650129	−0.48	0.630	.4722057	1.574994
2.high_risk_num	1.668212	1.119368	0.76	0.446	.4478124	6.214504
highriskatbirth						
Y	.8882196	.2522359	−0.42	0.676	.5090926	1.549687
mod_care_4						
standard	2.13658	1.428092	1.14	0.256	.5764682	7.918868
partial CoC	1.819327	1.310825	0.83	0.406	.4432208	7.467951
imp_gstt						
imp	129.3069	68.29544	9.21	0.000	45.92469	364.0801
_cons	6.43e−09	7.46e−06	−0.02	0.987	0	.
**3**						
place_hosp_comm						
hospital	.2324609	.117893	−2.88	0.004	.0860323	.6281138
ethnicity						
BA	.4448746	.2859349	−1.26	0.208	.1262245	1.567948
BC	.3192713	.3800974	−0.96	0.338	.0309593	3.292522
BO	.5933663	.7209856	−0.43	0.668	.0548337	6.420933
M	2.913797	2.942518	1.06	0.290	.4025942	21.08876
U	.9475622	.5389036	−0.09	0.925	.3108185	2.88874
WB	1.240831	.7237501	0.37	0.711	.3955715	3.892245
WO	1.116068	.5599861	0.22	0.827	.4174467	2.983873
WP	.2178736	3262.333	−0.00	1.000	0	.
age_cat						
20−24	4050570	6.18e+09	0.01	0.992	0	.
25−29	1.59e+07	2.43e+10	0.01	0.991	0	.
30−34	8673986	1.32e+10	0.01	0.992	0	.
greater than 34	1.36e+07	2.08e+10	0.01	0.991	0	.
1.parity	.6059389	.1940902	−1.56	0.118	.3234282	1.135219
imd_3_score						
3rd and 4th deciles	.8780057	.3331795	−0.34	0.732	.4173393	1.847164
5th and 6th deciles	.8363101	.3654243	−0.41	0.682	.35517	1.969239
least deprived	.445141	.2346754	−1.54	0.125	.158398	1.250966
1.any_risk	1.249047	.4500963	0.62	0.537	.6163776	2.531108
2.high_risk_num	2.048627	1.690593	0.87	0.385	.4064657	10.32528
highriskatbirth						
Y	.9095566	.3094149	−0.28	0.780	.4669455	1.771713
mod_care_4						
standard	.3472074	.2002005	−1.83	0.067	.1121461	1.074963
partial CoC	.8268466	.5089512	−0.31	0.757	.2474472	2.762914
imp_gstt						
imp	157.4338	106.1708	7.50	0.000	41.98182	590.384
_cons	5.57e−09	8.51e−06	−0.01	0.990	0	.
**4**						
place_hosp_comm						
hospital	.3067113	.1569143	−2.31	0.021	.112526	.8360008
ethnicity						
BA	.3042188	.2537595	−1.43	0.154	.0593156	1.560281
BC	1.390508	1.351873	0.34	0.735	.2068341	9.348128
BO	1.016634	1.393221	0.01	0.990	.0692878	14.9167
M	3.92e−06	.0041111	−0.01	0.991	0	.
U	1.078893	.7905951	0.10	0.917	.2565851	4.536542
WB	1.759525	1.233466	0.81	0.420	.4453323	6.951948
WO	.6506795	.4255932	−0.66	0.511	.1805583	2.344859
WP	.8377351	15882.32	−0.00	1.000	0	.
age_cat						
20−24	1.13e+07	2.00e+10	0.01	0.993	0	.
25−29	9189086	1.63e+10	0.01	0.993	0	.
30−34	1.52e+07	2.68e+10	0.01	0.993	0	.
greater than 34	7048785	1.25e+10	0.01	0.993	0	.
1.parity	.641973	.2655857	−1.07	0.284	.2853466	1.444311
imd_3_score						
3rd and 4th deciles	.6420809	.3098632	−0.92	0.359	.2493483	1.653381
5th and 6th deciles	.5092814	.2955374	−1.16	0.245	.1633064	1.588226
least deprived	.3570741	.2342331	−1.57	0.116	.0987161	1.291602
1.any_risk	.9700013	.4719995	−0.06	0.950	.3737493	2.51747
2.high_risk_num	5.082526	6.295067	1.31	0.189	.4485454	57.59076
highriskatbirth						
Y	1.833258	.8021245	1.39	0.166	.7776577	4.32174
mod_care_4						
standard	.1076106	.0883012	−2.72	0.007	.0215472	.5374261
partial CoC	1.724272	1.290989	0.73	0.467	.3974641	7.480207
imp_gstt						
imp	292.8011	330.4324	5.03	0.000	32.06074	2674.065
_cons	1.47e−09	2.60e−06	−0.01	0.991	0	.
**5**						
place_hosp_comm						
hospital	.3526896	.180827	−2.03	0.042	.1291142	.9634099
ethnicity						
BA	.1763719	.1389716	−2.20	0.028	.0376468	.8262865
BC	.4999369	.5108733	−0.68	0.497	.0674673	3.704565
BO	1.525588	1.867909	0.34	0.730	.1384323	16.81267
M	2.455025	3.417835	0.65	0.519	.1603359	37.59076
U	.8423045	.6002791	−0.24	0.810	.2083768	3.404778
WB	1.157315	.7833931	0.22	0.829	.3070921	4.361488
WO	.4219462	.2638713	−1.38	0.168	.1238636	1.437376
WP	1.23247	21857.73	0.00	1.000	0	.
age_cat						
20−24	2.160438	3.462691	0.48	0.631	.0933811	49.98325
25−29	3.158476	4.997976	0.73	0.467	.1420808	70.21337
30−34	3.283103	5.08069	0.77	0.442	.1581348	68.16189
greater than 34	4.27602	6.645841	0.93	0.350	.2032789	89.94712
1.parity	1.163698	.5050895	0.35	0.727	.4970344	2.724547
imd_3_score						
3rd and 4th deciles	.4457214	.2104513	−1.71	0.087	.1766693	1.124516
5th and 6th deciles	.4357005	.2474089	−1.46	0.143	.1431661	1.325976
least deprived	.256448	.1679203	−2.08	0.038	.0710622	.9254647
1.any_risk	1.752219	.8072836	1.22	0.223	.7102678	4.322695
2.high_risk_num	3.227525	4.080159	0.93	0.354	.2708952	38.45369
highriskatbirth						
Y	2.247512	.9909025	1.84	0.066	.9471462	5.333191
mod_care_4						
standard	.0255008	.0196506	−4.76	0.000	.0056316	.1154723
partial CoC	.8295332	.5361767	−0.29	0.772	.2336962	2.94453
imp_gstt						
imp	365.364	421.434	5.12	0.000	38.09746	3503.93
_cons	.0065914	[illegible]	−2.50	0.012	.0001283	[illegible]

Table 12: Number of missed appointments in relation to model of care received

miss_appt_4_cat	RRR	Std. Err.	z	P>|z|	[95% Conf.	Interval]
0	(base outcome)					
1						
mod_care_4						
standard	1.466618	.6491433	0.87	0.387	.6159775	3.491961
partial CoC	1.976347	.9047997	1.49	0.137	.8056957	4.847919
ethnicity						
BA	2.485515	1.125998	2.01	0.044	1.022826	6.03992
BC	1.94952	1.063413	1.22	0.221	.6692974	5.678536
BO	.8342489	.7124188	-0.21	0.832	.1564588	4.448272
M	.7729222	.6665585	-0.30	0.765	.1425838	4.189878
U	.9045829	.3983115	-0.23	0.820	.3816297	2.144147
WB	.909001	.4042185	-0.21	0.830	.3802318	2.173103
WO	1.45843	.5647411	0.97	0.330	.6827766	3.115247
WP	1.20e-06	.0036594	-0.00	0.996	0	.
age_cat						
20-24	.2842646	.2541574	-1.41	0.159	.0492804	1.639726
25-29	.2027736	.172672	-1.87	0.061	.0382094	1.0761
30-34	.2833981	.2325385	-1.54	0.124	.0567485	1.415269
greater than 34	.2004958	.1662139	-1.94	0.053	.0394864	1.018037
0.parity	.7442038	.1735291	-1.27	0.205	.4712092	1.175358
imd_3_score						
most deprived	1.779518	.6478759	1.58	0.113	.8717703	3.632474
3rd and 4th deciles	.7533867	.2805336	-0.76	0.447	.3631296	1.563055
5th and 6th deciles	1.253867	.4778692	0.59	0.553	.5940794	2.646417
1.any_risk	1.38075	.3691036	1.21	0.227	.8176568	2.331628
2.high_risk_num	.5591242	.2403068	-1.35	0.176	.2408071	1.298217
place_hosp_comm						
hospital	.9538957	.2571112	-0.18	0.861	.5624315	1.617827
imp_gstt						
imp	1.740019	.4995678	1.93	0.054	.99122	3.054485
_cons	.3266529	.3430343	-1.07	0.287	.0417059	2.558439
2						
mod_care_4						
standard	1.819736	1.136977	0.96	0.338	.5347799	6.192154
partial CoC	.8625367	.5949905	-0.21	0.830	.2231548	3.333872
ethnicity						
BA	11.34923	9.569888	2.88	0.004	2.173787	59.25374
BC	3.389846	3.648733	1.13	0.257	.4111224	27.95045
BO	7.72868	7.823851	2.02	0.043	1.062728	56.20676
M	6.162043	6.890638	1.63	0.104	.6884586	55.15332
U	6.547095	5.306048	2.32	0.020	1.337206	32.05524
WB	4.231173	3.552244	1.72	0.086	.8162852	21.93207
WO	3.399138	2.703933	1.54	0.124	.7149213	16.16141
WP	2.12e-06	.0091325	-0.00	0.998	0	.
age_cat						
20-24	.9444105	1.201994	-0.04	0.964	.0779468	11.44257
25-29	.8079029	1.015	-0.17	0.865	.0688591	9.478885
30-34	.3067858	.3842996	-0.94	0.346	.026337	3.573591
greater than 34	.2556753	.3218523	-1.08	0.279	.0216855	3.014454
0.parity	1.016398	.3258644	0.05	0.960	.5422039	1.905307
imd_3_score						
most deprived	.4342183	.2256039	-1.61	0.108	.1568393	1.202157
3rd and 4th deciles	.5644775	.2685964	-1.20	0.229	.2221354	1.434417
5th and 6th deciles	.6803869	.3456319	-0.76	0.448	.251392	1.841452
1.any_risk	2.112226	.7313112	2.16	0.031	1.071595	4.163417
2.high_risk_num	1.848433	1.227859	0.92	0.355	.5027771	6.795662
place_hosp_comm						
hospital	.6361429	.2928467	-0.98	0.326	.2580514	1.568206
imp_gstt						
imp	10.41672	5.60486	4.36	0.000	3.628525	29.90417
_cons	.0143837	.0240619	-2.54	0.011	.0005419	.3817767
3						
mod_care_4						
standard	2.029736	2.288317	0.63	0.530	.22274	18.49613
partial CoC	2.438196	2.827234	0.77	0.442	.2512174	23.66398
ethnicity						
BA	2.626047	2.073478	1.22	0.221	.5587396	12.34229
BC	1.201955	1.499941	0.15	0.883	.1041498	13.87132
BO	1.032836	1.324027	0.03	0.980	.0837234	12.74135
M	1.53e-06	.0016317	-0.01	0.990	0	.
U	.5783578	.5561831	-0.57	0.569	.0878276	3.808574
WB	1.778301	1.417697	0.72	0.470	.3727437	8.483993
WO	1.196887	.8684031	0.25	0.804	.2887104	4.96185
WP	1.60e-06	.0100035	-0.00	0.998	0	.
age_cat						
20-24	946768.2	2.68e+09	0.00	0.996	0	.
25-29	1081410	3.07e+09	0.00	0.996	0	.
30-34	1760311	4.99e+09	0.01	0.996	0	.
greater than 34	472766.8	1.34e+09	0.00	0.996	0	.
0.parity	.7531945	.3418027	-0.62	0.532	.309477	1.833098
imd_3_score						
most deprived	1.474234	1.08819	0.53	0.599	.3469455	6.264289
3rd and 4th deciles	.8275671	.6216565	-0.25	0.801	.1898423	3.60756
5th and 6th deciles	1.433326	1.086795	0.47	0.635	.3242916	6.335112
1.any_risk	2.895147	1.356331	2.27	0.023	1.155836	7.251788
2.high_risk_num	1.272324	1.592764	0.19	0.847	.1094001	14.79715
place_hosp_comm						
hospital	1.023997	.5942124	0.04	0.967	.3283651	3.193303
imp_gstt						
imp	9.324685	7.681576	2.71	0.007	1.85534	46.8646
_cons	2.80e-09	7.93e-06	-0.01	0.994	0	.
4						
mod_care_4						
standard	.2309158	.1969309	-1.72	0.086	.0434038	1.228514
partial CoC	.4939632	.4295315	-0.81	0.417	.0898509	2.715607
ethnicity						
BA	.6189749	.6022693	-0.49	0.622	.0919274	4.167747
BC	.3402144	.4352897	-0.84	0.399	.0277126	4.176653
BO	7.92e-07	.0011195	-0.01	0.992	0	.
M	4.08e-07	.0004059	-0.01	0.988	0	.
U	.7025714	.5593681	-0.44	0.657	.1475663	3.344983
WB	.373482	.3541876	-1.04	0.299	.0582159	2.396058
WO	.2917438	.2447006	-1.47	0.142	.0563709	1.509901
WP	3.24e-06	.0258196	-0.00	0.999	0	.
age_cat						
20-24	.1451364	.2109871	-1.33	0.184	.0084018	2.507158
25-29	.0522817	.0791982	-1.95	0.051	.0026848	1.018076
30-34	.040073	.059397	-2.17	0.030	.0021937	.7320142
greater than 34	.0252698	.0376016	-2.47	0.013	.0013678	.4668686
0.parity	.2194832	.1566993	-2.12	0.034	.0541613	.8894338
imd_3_score						
most deprived	1.402094	1.292939	0.37	0.714	.230061	8.544987
3rd and 4th deciles	.6948283	.6501306	-0.39	0.697	.1110281	4.348325
5th and 6th deciles	.8176601	.901942	-0.18	0.855	.0941092	7.104175
1.any_risk	1.932104	1.203268	1.06	0.290	.5700627	6.548446
2.high_risk_num	2.565955	2.56353	0.94	0.346	.3621195	18.18219
place_hosp_comm						
hospital	.3593408	.2686003	-1.37	0.171	.0830328	1.555118
imp_gstt						
imp	7.903945	7.665181	2.13	0.033	1.181288	52.88495
_cons	1.920439	3.991262	0.31	0.754	.0326846	112.8388

:ew only

Table 13: Number of missed appointments in relation to place of care

miss_appt_4_cat	RRR	Std. Err.	z	P>|z|	[95% Conf.	Interval]
0	(base outcome)					
1						
place_hosp_comm						
hospital	.9569555	.2576891	-0.16	0.870	.564521	1.622196
ethnicity						
BA	2.517118	1.14186	2.03	0.042	1.034586	6.124078
BC	1.946001	1.061045	1.22	0.222	.6683913	5.665725
BO	.8344806	.7122503	-0.21	0.832	.1566369	4.445681
M	.7755741	.6686059	-0.29	0.768	.1431597	4.201709
U	.9020032	.397027	-0.23	0.815	.3806642	2.137342
WB	.9135987	.4062334	-0.20	0.839	.3821793	2.183956
WO	1.456576	.5637853	0.97	0.331	.6821274	3.110291
WP	1.19e-06	.0036291	-0.00	0.996	0	.
age_cat						
20-24	.287718	.2572229	-1.39	0.163	.0498866	1.659396
25-29	.202999	.1727676	-1.87	0.061	.0382875	1.076294
30-34	.2834254	.2324265	-1.54	0.124	.0568068	1.41409
greater than 34	.2015995	.1670383	-1.93	0.053	.0397387	1.02274
1.parity	1.333352	.3112723	1.23	0.218	.8437839	2.106972
imd_3_score						
3rd and 4th deciles	.425283	.1189227	-3.06	0.002	.2458414	.7357007
5th and 6th deciles	.7094975	.2181208	-1.12	0.264	.3883882	1.296092
least deprived	.5615893	.2044222	-1.59	0.113	.275154	1.146204
1.any_risk	1.376409	.3679088	1.20	0.232	.8151256	2.324183
2.high_risk_num	.5711377	.2460416	-1.30	0.194	.2454991	1.328715
mod_care_4						
standard	1.473125	.6518644	0.88	0.381	.6188412	3.506712
partial CoC	1.979531	.9058473	1.49	0.136	.8073215	4.853758
imp_gstt						
imp	1.736336	.4981058	1.92	0.054	.9895738	3.046629
_cons	.4301065	.4174436	-0.87	0.385	.0641852	2.882153
2						
place_hosp_comm						
hospital	.6444555	.296171	-0.96	0.339	.2618232	1.586272
ethnicity						
BA	11.49696	9.698098	2.90	0.004	2.200715	60.06232
BC	3.370232	3.627379	1.13	0.259	.4088011	27.78481
BO	7.69689	7.790642	2.02	0.044	1.058634	55.96093
M	6.204943	6.936276	1.63	0.102	.6937629	55.49636
U	6.504876	5.271353	2.31	0.021	1.328774	31.84394
WB	4.261376	3.577301	1.73	0.084	.8222254	22.08558
WO	3.390012	2.696209	1.53	0.125	.7131934	16.1137
WP	2.10e-06	.0090408	-0.00	0.998	0	.
age_cat						
20-24	.9640915	1.227325	-0.03	0.977	.0795255	11.68773
25-29	.8111337	1.018517	-0.17	0.868	.069225	9.504333
30-34	.3079009	.3854809	-0.94	0.347	.026469	3.581664
greater than 34	.2579331	.3245363	-1.08	0.281	.0219033	3.037422
1.parity	.9754309	.3129515	-0.08	0.938	.5201182	1.829325
imd_3_score						
3rd and 4th deciles	1.306187	.5227048	0.67	0.504	.5961767	2.861777
5th and 6th deciles	1.578365	.7246758	0.99	0.320	.6417928	3.881684
least deprived	2.29793	1.193699	1.60	0.109	.830167	6.360747
1.any_risk	2.099106	.7269789	2.14	0.032	1.06473	4.138368
2.high_risk_num	1.91287	1.273571	0.97	0.330	.5187563	7.053547
mod_care_4						
standard	1.829594	1.142745	0.97	0.333	.5379022	6.22309
partial CoC	.8694436	.5991082	-0.20	0.839	.22527	3.355672
imp_gstt						
imp	10.31898	5.539971	4.35	0.000	3.602889	29.55446
_cons	.0062786	.0099685	-3.19	0.001	.0002795	.1410296
3						
place_hosp_comm						
hospital	1.027215	.5956154	0.05	0.963	.3296895	3.200501
ethnicity						
BA	2.648138	2.091771	1.23	0.218	.5630855	12.45394
BC	1.198983	1.495906	0.15	0.884	.1039478	13.82964
BO	1.033182	1.324002	0.03	0.980	.0838262	12.73427
M	1.54e-06	.0016388	-0.01	0.990	0	.
U	.5769185	.5547314	-0.57	0.567	.0876291	3.798224
WB	1.78182	1.420415	0.72	0.469	.3735172	8.499963
WO	1.194865	.866795	0.25	0.806	.2882896	4.952322
WP	1.59e-06	.0099383	-0.00	0.998	0	.
age_cat						
20-24	941966.5	2.65e+09	0.00	0.996	0	.
25-29	1064254	2.99e+09	0.00	0.996	0	.
30-34	1731111	4.87e+09	0.01	0.996	0	.
greater than 34	466779.2	1.31e+09	0.00	0.996	0	.
1.parity	1.320394	.599513	0.61	0.540	.5422793	3.215022
imd_3_score						
3rd and 4th deciles	.563164	.3030157	-1.07	0.286	.1961723	1.616709
5th and 6th deciles	.976508	.5736932	-0.04	0.968	.3087447	3.088532
least deprived	.6780669	.500478	-0.53	0.599	.1595896	2.880981
1.any_risk	2.886719	1.35232	2.26	0.024	1.15252	7.230371
2.high_risk_num	1.296785	1.62442	0.21	0.836	.1113293	15.10521
mod_care_4						
standard	2.037241	2.296359	0.63	0.528	.2236537	18.55704
partial CoC	2.44362	2.832502	0.77	0.441	.2519827	23.69718
imp_gstt						
imp	9.301962	7.659612	2.71	0.007	1.852085	46.71844
_cons	3.15e-09	8.84e-06	-0.01	0.994	0	.
4						
place_hosp_comm						
hospital	.3605907	.2694494	-1.37	0.172	.0833602	1.559804
ethnicity						
BA	.6230185	.6064412	-0.49	0.627	.0924588	4.198108
BC	.3400471	.4350152	-0.84	0.399	.0277086	4.173147
BO	7.95e-07	.0011212	-0.01	0.992	0	.
M	4.11e-07	.0004077	-0.01	0.988	0	.
U	.70192	.5587515	-0.44	0.657	.1474698	3.340967
WB	.3747134	.3553621	-1.04	0.301	.0584059	2.404042
WO	.2916114	.244571	-1.47	0.142	.0563523	1.509027
WP	3.22e-06	.0256849	-0.00	0.999	0	.
age_cat						
20-24	.1464599	.2129082	-1.32	0.186	.0084787	2.52992
25-29	.052439	.0794202	-1.95	0.052	.0026946	1.020519
30-34	.0401708	.0595274	-2.17	0.030	.0022007	.733278
greater than 34	.025392	.0377773	-2.47	0.014	.001375	.4689034
1.parity	4.534777	3.238673	2.12	0.034	1.11851	18.38535
imd_3_score						
3rd and 4th deciles	.496867	.3204713	-1.08	0.278	.1403553	1.758942
5th and 6th deciles	.5848716	.510162	-0.61	0.539	.1058251	3.232454
least deprived	.7131029	.6575218	-0.37	0.714	.1170295	4.345191
1.any_risk	1.928033	1.200607	1.05	0.292	.5689343	6.533816
2.high_risk_num	2.581789	2.580332	0.95	0.343	.3640824	18.30804
mod_care_4						
standard	.2315544	.197482	-1.72	0.086	.0435214	1.231979
partial CoC	.494682	.4300472	-0.81	0.418	.0900206	2.718381
imp_gstt						
imp	7.884151	7.64327	2.13	0.033	1.179125	52.71693
_cons	.5883176	1.026983	-0.30	0.761	.019219	18.00909

w only

Table 14: Qualitative quotations relating to women's experiences of continuity of care, missing and rescheduling appointments

'I met all of them when I was going for my appointments at [Community centre]. I met all of them before I gave birth…and when I gave birth all of them came to see me at home one by one…. they was taking care of me. Every time when I'm at home I call them, they pick up my call, they know everything about me. Yeah but not the midwife at hospital.' (CBM7)
'There are six of them [CBM midwives]… I met all of them, when I have the baby there going to be one of them… in the hospital, which is I never heard this. Which is good, they know you and then they know all the, the problem or the issues you have' (CBM2)
'sometimes if I was getting anxious and worried, um, I felt like I didn't want to bother or inconvenience [named midwife] even though I had her mobile number and I think in the beginning I did try to utilise it but it, she wasn't either at work or someone else would pick up from the team and say, 'Oh she's not here.' And so that would kind of knock my confidence, so I wouldn't kind of ring her. I just get told that they're rushed off their feet, you have one midwife to six, seven ladies when they're all in active labour, so I don't, I wouldn't think that they would be there for 24 hours, I think the kind of contact is very minimal. the midwife kind of works like nine to five. And I know that she, um, isn't in some days, so any type of concerns like that I'll just rather ring the triage' (HBM8)
'because she [HBM midwife] says she will be off…so I didn't want to call another midwife and explain everything, so I just went straight… to the GP because I was feeling bad, yeah, the GP, yeah she say that I had to go back to the hospital… in the Emergency they say because I was five days after the operation they cannot see me, so they send me to the labour ward. (HBM10)
'That is something that does worry me a little bit is like who's going to come [to provide care at homebirth]?...obviously you don't know when the baby's going to come so you don't know a hundred percent who's going to come round to help you. So that's a bit … daunting. (HBM2)
'she [midwife] cancelled a lot of my appointments….I understand that people have babies. but it meant that it was cancelled appointments again and again and again… I only had maybe one or two midwife appointments during my pregnancy…. it suffered me because I didn't go to antenatal classes, because she didn't really sit there and go, 'Well this is what's available'…. so because of me not having the right sort of preparation for the baby it kind of led to bigger things being involved because they [social care] were scared that I wasn't picking up cues and I wasn't doing this, and I

For peer review only

wasn't doing that… It was an incredibly stressful time for me… And when I sat there and said, 'Look I didn't have any antenatal care,' they were a little bit more understanding of why I wasn't picking up on those cues'(HBM1)

'because of my situation I called them to change it…. if it's not a convenient time, I'm happy to change it.' (CMB1)

For peer review only

Reporting checklist for quality improvement in health care.

Based on the SQUIRE guidelines.

Instructions to authors

Complete this checklist by entering the page numbers from your manuscript where readers will find each of the items listed below.

Your article may not currently address all the items on the checklist. Please modify your text to include the missing information. If you are certain that an item does not apply, please write "n/a" and provide a short explanation.

Upload your completed checklist as an extra file when you submit to a journal.

In your methods section, say that you used the SQUIREreporting guidelines, and cite them as:

Ogrinc G, Davies L, Goodman D, Batalden P, Davidoff F, Stevens D. SQUIRE 2.0 (Standards for QUality Improvement Reporting Excellence): revised publication guidelines from a detailed consensus process

		Reporting Item	Page Number
**Title**			
	#1	Indicate that the manuscript concerns an initiative to improve healthcare (broadly defined to include the quality, safety, effectiveness, patientcenteredness, timeliness, cost, efficiency, and equity of healthcare)	1
**Abstract**			
	#02a	Provide adequate information to aid in searching and indexing	2
	#02b	Summarize all key information from various sections of the text using the abstract format of the intended publication or a structured summary such as: background, local problem, methods, interventions, results, conclusions	2
**Introduction**			
Problem description	#3	Nature and significance of the local problem	3

For peer review only

Available knowledge	#4	Summary of what is currently known about the problem, including relevant previous studies	3
Rationale	#5	Informal or formal frameworks, models, concepts, and / or theories used to explain the problem, any reasons or assumptions that were used to develop the intervention(s), and reasons why the intervention(s) was expected to work	3
Specific aims	#6	Purpose of the project and of this report	3/4
**Methods**			
Context	#7	Contextual elements considered important at the outset of introducing the intervention(s)	n/a - Appendix A
Intervention(s)	#08a	Description of the intervention(s) in sufficient detail that others could reproduce it	n/a - Appendix A
Intervention(s)	#08b	Specifics of the team involved in the work	n/a -Appendix A
Study of the Intervention(s)	#09a	Approach chosen for assessing the impact of the intervention(s)	4
Study of the Intervention(s)	#09b	Approach used to establish whether the observed outcomes were due to the intervention(s)	5
Measures	#10a	Measures chosen for studying processes and outcomes of the intervention(s), including rationale for choosing them, their operational definitions, and their validity and reliability	5
Measures	#10b	Description of the approach to the ongoing assessment of contextual elements that contributed to the success, failure, efficiency, and cost	5
Measures	#10c	Methods employed for assessing completeness and accuracy of data	5
Analysis	#11a	Qualitative and quantitative methods used to draw inferences from the data	5
Analysis	#11b	Methods for understanding variation within the data, including the effects of time as a variable	25
Ethical considerations	#12	Ethical aspects of implementing and studying the intervention(s) and how they were addressed, including, but	4

For peer review only

		not limited to, formal ethics review and potential conflict(s) of interest	
**Results**			
	#13a	Initial steps of the intervention(s) and their evolution over time (e.g., time-line diagram, flow chart, or table), including modifications made to the intervention during the project	n/a -Appendix A
	#13b	Details of the process measures and outcome	11-21
	#13c	Contextual elements that interacted with the intervention(s)	11-21 - described alongside each table and quotation
	#13d	Observed associations between outcomes, interventions, and relevant contextual elements	11-21 - described alongside each table and quotation
	#13e	Unintended consequences such as unexpected benefits, problems, failures, or costs associated with the intervention(s).	25
	#13f	Details about missing data	5, Figure 1, Appendix B
**Discussion**			
Summary	#14a	Key findings, including relevance to the rationale and specific aims	23
Summary	#14b	Particular strengths of the project	26
Interpretation	#15a	Nature of the association between the intervention(s) and the outcomes	23-25
Interpretation	#15b	Comparison of results with findings from other publications	23-25
Interpretation	#15c	Impact of the project on people and systems	23-25
Interpretation	#15d	Reasons for any differences between observed and anticipated outcomes, including the influence of context	23-25
Interpretation	#15e	Costs and strategic trade-offs, including opportunity costs	23-15

For peer review only

Limitations	#16a	Limits to the generalizability of the work	26
Limitations	#16b	Factors that might have limited internal validity such as confounding, bias, or imprecision in the design, methods, measurement, or analysis	26
Limitations	#16c	Efforts made to minimize and adjust for limitations	26
Conclusion	#17a	Usefulness of the work	26
Conclusion	#17b	Sustainability	26
Conclusion	#17c	Potential for spread to other contexts	26
Conclusion	#17d	Implications for practice and for further study in the field	26
Conclusion	#17e	Suggested next steps	26
**Other information**			
Funding	#18	Sources of funding that supported this work. Role, if any, of the funding organization in the design, implementation, interpretation, and reporting	27

Notes:

- 7: n/a - Appendix A
- 08a: n/a - Appendix A
- 08b: n/a -Appendix A
- 13a: n/a -Appendix A
- 13c: 11-21 - described alongside each table and quotation
- 13d: 11-21 - described alongside each table and quotation
- 13f: 5, Figure 1, Appendix B The SQUIRE 2.0 checklist is distributed under the terms of the Creative Commons Attribution License CC BY-NC 4.0. This checklist was completed on 25. April 2022 using https://www.goodreports.org/, a tool made by the EQUATOR Network in collaboration with Penelope.ai

For peer review only

BMJ Open

Project20: Maternity care mechanisms that improve access and engagement for women with social risk factors in the UK. A mixed-methods, realist evaluation

Journal:	*BMJ Open*
Manuscript ID	bmjopen-2022-064291.R1
Article Type:	Original research
Date Submitted by the Author:	05-Dec-2022
Complete List of Authors:	Rayment-Jones, Hannah; King's College London, Women and Children's Health<br>Dalrymple, Kathryn; King's College London, Women and Children's Health<br>Harris, James; University College London Hospitals NHS Foundation Trust, Centre for Nursing, Midwifery and Allied Health Professional Research<br>Harden, Angela; City University of London<br>Parslow, Elidh; North Middlesex University Hospital NHS Trust<br>Georgi, Thomas; King's College London<br>Sandall, Jane; King's College London, Health and Social Care Research Division
**Primary Subject Heading**:	Health services research
Secondary Subject Heading:	Obstetrics and gynaecology, Nursing
Keywords:	Organisation of health services < HEALTH SERVICES ADMINISTRATION & MANAGEMENT, PRIMARY CARE, PUBLIC HEALTH, SOCIAL MEDICINE

SCHOLARONE™
Manuscripts

I, the Submitting Author has the right to grant and does grant on behalf of all authors of the Work (as defined in the below author licence), an exclusive licence and/or a non-exclusive licence for contributions from authors who are: i) UK Crown employees; ii) where BMJ has agreed a CC-BY licence shall apply, and/or iii) in accordance with the terms applicable for US Federal Government officers or employees acting as part of their official duties; on a worldwide, perpetual, irrevocable, royalty-free basis to BMJ Publishing Group Ltd ("BMJ") its licensees and where the relevant Journal is co-owned by BMJ to the co-owners of the Journal, to publish the Work in this journal and any other BMJ products and to exploit all rights, as set out in our licence.

The Submitting Author accepts and understands that any supply made under these terms is made by BMJ to the Submitting Author unless you are acting as an employee on behalf of your employer or a postgraduate student of an affiliated institution which is paying any applicable article publishing charge ("APC") for Open Access articles. Where the Submitting Author wishes to make the Work available on an Open Access basis (and intends to pay the relevant APC), the terms of reuse of such Open Access shall be governed by a Creative Commons licence – details of these licences and which Creative Commons licence will apply to this Work are set out in our licence referred to above.

Other than as permitted in any relevant BMJ Author's Self Archiving Policies, I confirm this Work has not been accepted for publication elsewhere, is not being considered for publication elsewhere and does not duplicate material already published. I confirm all authors consent to publication of this Work and authorise the granting of this licence.

For peer review only

Project20: Maternity care mechanisms that improve access and engagement for women with social risk factors in the UK. A mixed-methods, realist evaluation

For peer review only

Corresponding author:
Hannah Rayment-Jones RM, PhD, PGCE, FHEA
NIHR Post-Doctoral Research Fellow
Department of Women and Children's Health
Faculty of Life Sciences & Medicine, King's College London
10th Floor, North Wing St. Thomas' Hospital
Westminster Bridge Road, London, SE1 7EH
Hannah.rayment-jones@kcl.ac.uk

Kathryn Dalrymple PhD
Post-Doctoral Researcher
Department of Women and Children's Health
Faculty of Life Sciences & Medicine, King's College London
10th Floor, North Wing St. Thomas' Hospital
Westminster Bridge Road, London, SE1 7EH
kathryn.dalrymple@kcl.ac.uk

James Harris, RM, PhD, BSc, PGCE, FHEA
Senior Clinical Lecturer for Evidence Based Midwifery

For peer review only - http://bmjopen.bmj.com/site/about/guidelines.xhtml

Chelsea and Westminster NHS Foundation Trust
Clinical Research Facility, St Stephen’s Centre
252 Fulham Road, London, SW109NA
james.harris4@nhs.net

Angela Harden
Professor of Health Sciences
School of Health Sciences
City, University of London
Northampton Square
London EC1V 0HB
angela.harden@city.ac.uk

Elidh Parslow
Birth Centre Matron
Imperial College NHS Trust
Birth Centre, St Mary's Hospital,
London W2 1NY
Elidh.parslow@nhs.net

Thomas Georgi, MBBS, BA
School of Population Health & Environmental Sciences
Faculty of Life Sciences & Medicine
King’s College London
London, SE1 7EH
Tom.Georgi@nhs.net

Jane Sandall CBE PhD MSc BSc RM HV RN
Professor of Social Science and Women's Health
Department of Women and Children’s Health
Faculty of Life Sciences & Medicine, King’s College London
10th Floor, North Wing St. Thomas' Hospital
Westminster Bridge Road, London, SE1 7EH

For peer review only

jane.sandall@kcl.ac.uk

Abstract

Objectives: To evaluate how women access and engage with different models of maternity care, whether specialist models improve access and engagement for women with social risk factors, and if so, how?

Design: Realist evaluation

Setting: Two UK maternity service providers

Population: Women accessing maternity services in 2019 (n=1020)

Methods: Prospective observational cohort with multinomial regression analysis to compare measures of access and engagement between models and place of antenatal care. Realist informed, longitudinal interviews with women accessing specialist models of care were analysed to identify mechanisms.

Main outcome measures: Measures of access and engagement, healthcare-seeking experiences.

Results: The number of social risk factors women were experiencing increased with deprivation score, with the most deprived more likely to receive a specialist model that provided continuity of care. Women attending hospital-based antenatal care were more likely to access maternity care late (RR 2.51 CI1.33-4.70), less likely to have the recommended number of antenatal appointments (RR0.61 CI0.38-0.99) and more likely to have over 15 appointments (RR4.90 CI2.50-9.61) compared to community-based care. Women accessing standard care (RR0.02, CI0.00-0.11) and Black women (RR0.02, CI0.00-0.11) were less likely to have appointments with a known healthcare professional compared to the specialist model. Qualitative data revealed mechanisms for improved access and engagement including self-referral,

For peer review only - http://bmjopen.bmj.com/site/about/guidelines.xhtml

relational continuity with a small team of midwives, flexibility, and situating services within deprived community settings.

Conclusion: Inequalities in access and engagement with maternity care appears to have been mitigated by the community-based specialist model that provided continuity of care. The findings enabled the refinement of a realist programme theory to inform those developing maternity services in line with current policy.

Keywords: Inequality, maternity care, continuity of care, access and engagement, social risk factors

Strengths and limitations of this study

- The specialist models of care evaluated in this study are situated in the UK's complex maternity system and can be appropriately investigated using a mixed-methods, realist evaluation design.
- Both qualitative and quantitative methods are used to understand not only if access and engagement are improved by specialist models of care, but how and in what context.
- Longitudinal interviews were undertaken to increase trust between participants and the researcher and lessen the perception of surveillance.
- 'The relatively small and varied numbers in each quantitative data group, and the multiple testing required to establish the separate effects of the model of care, place of care and service attended might result in a change in statistical power, reducing the probability of detecting effects when they do exist.'
- The generalisability of the findings is limited by the urban location of both specialist models of care evaluated and the UK health system context.

For peer review only

Introduction

As a core component of maternity care across the globe, the adequacy of antenatal care is measured by the timing of antenatal care access and the number of appointments attended [1]. The routine functions of antenatal care include support, health promotion, screening and diagnosis, disease prevention, and additional care for women at higher risk [2]. When these functions are of high quality and care is well attended, it makes a crucially important contribution to the reduction of health inequalities [3,4]. The World Health Organisation (WHO) [1] recommend a minimum of eight antenatal appointments to reduce perinatal mortality and improve women's experiences. In high-income countries such as the UK, antenatal care coverage is consistently high and correlates with relatively low maternal and infant mortality rates when compared to the LMIC's [5]. Despite this, there are marked inequalities in access, health outcomes and women's experiences with poor antenatal care uptake for women from lower socioeconomic and minority groups [6–10] . The most deprived women in the United Kingdom are 60% less likely to have received any antenatal care when compared to the least deprived women [6].

Factors associated with inequalities in maternal and neonatal outcomes include Black and minority ethnicity, poverty, young motherhood, homelessness, difficulty speaking or understanding English, migrant or refugee status, domestic violence, mental illness and substance abuse [7,11–13]. It is hypothesised that a lack of access and engagement with maternity services is directly linked to health inequalities for women with these social risk factors [14–17]. Therefore, policies to tackle inequalities often focus on improving access to maternity services and continuity of midwifery care [18 19]. There is a strong evidence base documenting improved maternity outcomes and experiences for women who receive continuity models of midwifery care [20] but less is known about what works to improve access and engagement for women with social risk factors [21]. The impact of place-based maternity care is also poorly understood. The specialist models of care being evaluated in this study provide continuity of care across the antenatal, intrapartum and postnatal periods from a one-to-one midwife or small team of midwives whom women have had the opportunity to meet during pregnancy. One model is based in the community setting and provides care to women in the local, deprived catchment area, the other is based in a large teaching hospital and women with significant social risk factors are referred to the model of care early in pregnancy. See supplementary file 1 for full definitions of each type of maternity care women may experience, and those being compared in this study. As part of a wider evaluation these two

For peer review only - http://bmjopen.bmj.com/site/about/guidelines.xhtml

UK-based specialist models, this study aimed to describe and compare service use for women with and without social risk factors by asking:

1) Are women with social risk factors more likely to be offered the specialist models of care over those without? If so, do they find this acceptable?

2) Compared to standard maternity care and group practice models, do specialist models improve access and engagement with maternity services? If so, for whom, in what context, and how?

3) Do specialist models of care increase the quality of relational continuity and reduce the number of appointments women miss?

4) How does the location of a model of care- community or hospital, impact on women's access and engagement with maternity services?

Methods

Study design

Realist methodology is a theoretically informed approach to evaluating complex interventions and often uses mixed methods to understand *how* interventions, in this case models of maternity care, do or do not work in different contexts [22]. This allows those implementing services such as specialist models of care to refine, scale-up, or even withdraw the service [23]. The aims of this study were approached through the testing and refinement of initial programme theories constructed in an earlier synthesis of literature [24] and focus groups with midwives[25] relating to how women with social risk factors access and engage with maternity care- see figure 1 for the theory refinement process. Retroductive theorizing was used to uncover meaningful causal mechanisms, often focusing on how the wider context and human response to different aspects of maternity care leads to specific outcomes [26]. This approach offers an epistemologically and ontologically grounded way of integrating mixed methods, often analysing qualitative data to find the causal relationship behind quantitative findings [27]. Routinely collected pregnancy and birth outcome data for 1000 women accessing different maternity models, including standard care, group practice and specialist models of care at two large, inner-city

For peer review only - http://bmjopen.bmj.com/site/about/guidelines.xhtml
For peer review only

maternity services were prospectively collected and analysed. Longitudinal interviews with 20 women with social risk factors were conducted to uncover the mechanisms for any associations found in the cohort analysis. Supplementary file 1 provides definitions of the two service provider settings and describes the different models of care women might experience at each. Supplementary file 2 demonstrates the detailed, methodological process of programme theory (PT) refinement and development of the context+mechanism=outcome (CMO) configuration and provides adjusted and unadjusted data.

The research was approved by the London Brent Research Ethics Committee (HRA) REC Reference 18-LO-0701. All participants provided written consent by signing a consent form approved by the ethics committee. The quantitative data collected were anonymised before being made available to the research team. A service user panel contributed to the study through external peer review for funding, the research focus, design, and analysis of data. The SQUIRE checklist was utilised to report this new knowledge for use in healthcare services [28].

Patient and Public Involvement

Multiple representative, diverse groups of service users were involved in the planning and development of this research. They were recruited through local community groups, clinicians, and existing patient involvement groups. Using participatory appraisal methods and online engagement events, recent maternity service users provided feedback on the protocol, study materials, interview guides, and refinement of programme theories. They also prioritised outcome measures and reviewed the qualitative data analysis. Training needs were identified by the service users for analysis of quantitative data and further research addressing maternal health inequities [29].

Data collection

Quantitative data collection

A power calculation was based on a previous analysis of UK antenatal care utilisation [6] and validated metrics for monitoring local inequalities in access to care at a service evaluated in this research [30]. We calculated that with 250 women in each group (those receiving standard maternity care and those receiving group practice or a specialist model of care), we would have 90% power to detect a 15%

For peer review only - http://bmjopen.bmj.com/site/about/guidelines.xhtml

difference in timely access to antenatal care (before 12+6 weeks gestation) between the different models of care with 500 anonymised birth records accessed at each trust. Pregnancy and birth outcome data were extracted from computerised records at each service provider. Data collection was prospective; the demographics of the first 500 women booking for maternity care with each service provider in January 2019 were collected, with pregnancy and birth outcomes collected later in the year when all women had been discharged from maternity care. Deprivation deciles calculated using the 2019 English Indices of Deprivation [31] were grouped into four groups of sufficient numbers to enable comparisons between groups of similar numbers.

Semi-structured, longitudinal interviews with 20 women with low socioeconomic status and social risk factors who were receiving specialist care from one of the two service providers were carried out at approximately 28- and 36-weeks' gestation, and 6 weeks post birth. Women were identified by the specialist model midwives providing their care if they met the following inclusion criteria: A deprivation score [32] of higher than 30 and/or secondary school as the highest level of education attained. An interview guide was developed using previous literature [24,25,33] to elicit specific mechanisms explaining how the specialist model might improve access and engagement.

Analysis

The quantitative data were analysed using Stata 16.0. Firstly, women's social risk factors, ethnicity, socioeconomic status and medical characteristics were described using descriptive statistics and stratified by the service provider attended to enable comparisons of differences in the samples between each service. Variables were tested for bivariate association using chi-square tests and *t*-tests, for dichotomous and continuous variables, respectively. Chi-square analyses were also performed to test for associations between socio-economic position by deprivation (IMD) decile [34], as well as social and medical risk factors. Secondly, three regression models were developed: Model 1 adjusted for ethnicity, age, parity, deprivation score, social risk factors and medical risk status, Model 2 included model 1, plus adjustment for the service provider that women attended to consider differences in organisation guidelines, processes and culture and Model 3 included model 2, however, the place of antenatal care (hospital versus community-based antenatal care) was treated as the independent variable. This structured model allowed us to explore the association between access and engagement depending on the model of care received, whilst accounting for interactions between independent variables to predict the dependent variable. Risk ratios and confidence intervals are used to demonstrate statistical significance as well as the

For peer review only - http://bmjopen.bmj.com/site/about/guidelines.xhtml

direction and strength of the effect [35]. Definitions for all demographics and social risk factors are given in supplementary file 1.

The qualitative data were coded using NVivo v.12 and analysed using a thematic framework analysis [36]. This allowed for the organisation of a large qualitative dataset into a coding framework developed using previously constructed programme theories [24,25], to uncover new theories and differences in women's experiences depending on their characteristics [36]. The method suited the longitudinal approach to data collection as changes in women's perceptions and relationships with healthcare providers could be seen over the course of their pregnancy and postnatal period. We utilised existing models of data adequacy [37] to assess acceptable data quality. The initial programme theories relating to access and engagement were refined and constructed as a Context + Mechanism= Outcome (CMO) configurations that present specific aspects of the specialist models of care.

Results

Full pregnancy and birth data were collected and analysed from 799 women accessing care across the two service providers. Two hundred and one sets of birth outcome data were missing due to sample drop out, that is that those women stopped receiving care at the service and were therefore excluded from the final analysis. . The total number and reasons given for women with missing outcome data at each hospital did not differ significantly. See Figure 2 for the data collection flowchart.

Demographics of women included in the quantitative data analysis

Table 1 presents the demographic profiles of the 799 women who continued their pregnancies and gave birth at the two services, with significant differences in ethnicity, social and medical risk factors, and model of care received highlighted and adjusted for in the analysis of access and engagement outcomes. See supplementary file 1 for definitions relating to risk factors and model of care.

For peer review only - http://bmjopen.bmj.com/site/about/guidelines.xhtml

Table 1: Women's demographics at each service provider

Demographic variable	Service A n(%) Total data = 405	Service B n(%) Total data= 394	TOTAL n(%) Total data=799	X^2 p value
**Ethnicity**				**p<0.001**
Asian	37(9)	53(13)	90(11)	
Black African	31(8)	46(12)	77(10)	
Black Caribbean	23(6)	16(4)	39(5)	
Black other	8(2)	14(4)	22(3)	
Mixed	12(3)	7(2)	19(2)	
White British	98(24)	58(15)	156(20)	
White other	80(20)	139(36)	219(27)	
Unknown	116(29)	61(15)	177(22)	
**Age**				p0.356
≤20	6(1)	4(1)	10(1)	
21-24 years	19(5)	32(8)	51(6)	
25-29 years	63(16)	56(14)	119(15)	
30-34 years	134(33)	125(32)	259(32)	
≥35 years	183(45)	177(45)	360(45)	
**Parity**				p0.167
Primiparous	212(52)	187(47)	399(50)	
Multiparous	193(48)	207(53)	400(50)	
**Medical Risk**				
High at booking	118(29)	106(27)	224(28)	p0.496
High at onset of labour	152(38)	223(57)	375(47)	**p<0.001**
**Social Risk Factor**				
Domestic abuse	23(6)	17(4)	40(5)	p0.377
Common mental health	4(1)	34(9)	38(5)	**p<0.001**
Severe mental health	2(<1)	8(2)	10(1)	p0.051
Non-English speaking	16(4)	48(13)	64(8)	**p<0.001**
Social care involvement	27(7)	29(7)	56(7)	p0.701
Drug/alcohol abuse	1(<1)	10(3)	11(1)	**p<0.001**
Unsupported/single	1(<1)	11(3)	12(2)	**p<0.001**
Financial/housing	15(4)	31(8)	46(6)	**p<0.001**
Learning disability	6(2)	5(1)	11(1)	p0.797
Sexual abuse/trafficked	4(2)	5(1)	9(1)	p0.677
AS/Refugee	8(2)	7(2)	15(2)	p0.836
FGM	0	11(3)	11(1)	**p<0.001**
No recourse to public funds	6(1)	0	6(1)	**p<0.001**

For peer review only

For peer review only - http://bmjopen.bmj.com/site/about/guidelines.xhtml

**No of social risk factors**				**p<0.001**
None	337(83)	279(70)	616(77)	
1	43(11)	61(15)	104(13)	
2	13(3)	26(7)	39(5)	
3	6(1)	15(4)	21(3)	
4	5(1)	9(2)	14(2)	
≥5	1(<1)	4(1)	5(1)	
**Name of model of care**				**p<0.001**
Standard Care	256(63)	213(54)	469(59)	
Group Practice	77(19)	144(37)	221(28)	
Specialist	59(15)	21(5)	80(10)	
Private Care	13(3)	16 (4)	29(4)	
**Place of model of antenatal care**				**p<0.001**
Standard model in hospital	100(25)	212(54)	312(39)	
Standard model in community	156(40)	1(0)	157(20)	
Group practice in community	40(10)	94(24)	134(17)	
Group practice in hospital	37(9)	50(13)	87(11)	
Specialist model in community	59(15)	2(1)	61(8)	
Specialist model in hospital	0	19(5)	19(2)	
Private Care	13(3)	16(4)	29(4)	
**By place of antenatal care only***				**p<0.001**
Hospital based	137(35)	281(74)	418(54)	
Community based	255(65)	97(26)	352(46)	

Characteristics of the women interviewed

Twenty pregnant women with low socio-economic status and/or educational attainment were recruited- See Table 2. All twenty women received a specialist model of care, eight were first time mothers and the other twelve had between one and eight children. For five of the multiparous women, this was their first pregnancy in the UK. Based on 2019 deprivation scores [31], 19 participants were in the 1st or 2nd most deprived deciles, with one in the 3rd and 4th decile group. Twelve participants were born outside of the UK, and nine did not speak English and required an interpreter. All participants were experiencing between one and seven social risk factors including common or severe mental health issues, domestic violence, drug/alcohol misuse, no support, single motherhood, financial and housing problems, learning disability, sexual abuse, trafficking, female genital mutilation, and no recourse to public funds. Five

For peer review only - http://bmjopen.bmj.com/site/about/guidelines.xhtml

participants were seeking asylum, had refugee status, or had had an asylum claim refused and nine had social care involvement during their pregnancy.

Table 2: Demographics of women interviewed

Characteristic	Community based model (CBM) n=10	Hospital based model (HBM) n=10	TOTAL n(%) n=20
**Ethnicity and migration status**			
**Born outside the UK:**	7	5	12(60)
Asian	0	2	2(10)
Black African	3	0	3(15)
Black Caribbean	0	1	1(5)
White	4	2	6(30)
Asylum seeker/refugee*	2	3	5(25)
**Born inside the UK:**	3	5	8(40)
Asian British	1	1	2(10)
Black British	2	1	3(15)
White British	0	3	3(15)
**Not proficient in English language/ interpreter required**	5	4	9(45)
**Age**			
18-24	0	3	3(13)
25-29	1	1	2(2)
30-34	5	5	10(50)
>34	4	1	5(25)
**Parity**			
Primiparous	5	3	8(40)
**IMD Decile (2019)**			
Most deprived 1st +2nd	9	10	19(95)
3rd and 4th	1	0	1(5)
Least deprived 5th-10th	0	0	0
**No. of social risk factors**			
1	3	0	3(15)
2	0	2	2(10)
3	2	0	2(10)
4	1	1	2(10)
≥5	4	7	11(55)
**Mental Illness**			
Common	5	9	14(70)

For peer review only - http://bmjopen.bmj.com/site/about/guidelines.xhtml

Severe	1	1	2(15)
**Level of education**			
Secondary school only	5	6	14(70)
Completed college	4	3	2(15)
Completed university	1	1	4(20)
**High medical risk at booking**	7	5	12(60)

*Including women whose asylum claim had been refused.

For peer review only

Model of care accessed by deprivation score and risk factors

When merging data for the two service providers Table 3 shows that women in the most deprived deciles were significantly more likely to receive a specialist model of care, and women in the least deprived deciles were less likely to receive community-based antenatal care than hospital-based antenatal care. This reflects the aim of the service providers to offer women in more deprived areas both specialist and/or community-based antenatal care. A statistically significant relationship was found between deprivation score and the number of social risk factors recorded.

Table 3: Maternity care received, and risk factors recorded by deprivation score

**Maternity care and risk factors**	**Least deprived (7th, 8th, 9th +10th)**	**5th and 6th deciles**	**3rd and 4th deciles**	**Most deprived (1st +2nd deciles)**	**Total n(%)**	**X^2 p value**
**Model of care**	Total=149	Total=158	Total=286	Total=206	N=799	p<0.001
Standard Care	81(54)	98(62)	163(57)	127(62)	469(58)	
Group Practice	43(29)	41(26)	85(30)	52(25)	221(28)	
Specialist	3(2)	16(10)	34(12)	27(13)	80(10)	
Private Care	22(15)	3(2)	4(1)	0(0)	29(4)	
**Place of antenatal care***	Total=127	Total=155	Total=282	Total=206	Total=770	p<0.001
Hospital based	82(65)	91(59)	134(48)	111(54)	418(54)	
Community based	45(35)	64(41)	148(52)	95(46)	352(46)	
**Social Risk Factors**	Total=149	Total=158	Total=286	Total=206	N=799	p<0.001
None	133(89)	132(84)	212(74)	139(67)	616(77)	

One	10(7)	17(11)	50(17)	27(13)	104(13)	
Two	5(3)	4(3)	10(4)	20(10)	39(5)	
Three	1(1)	2(1)	10(4)	8(4)	21(3)	
Four or more	0	3(2)	4(1)	12(6)	19(2)	
**Medical Risk**						
High at booking	37(25)	46(29)	85(30)	56(27)	224(28)	p0.743
High at onset of labour	62 (42)	79(50)	138(48)	96(47)	375(47)	p0.475

*Excludes private care

Testing using qualitative data: Women reported different pathways into the specialist models including direct referrals from a GP, midwives, sexual health clinics, social workers, emergency departments, and self-referral. Overall women were pleased to be referred to the specialist model, however they were often not made aware of *why* they had been referred and described a lack of choice.

I don't know if it's because I have these issues, only, or if it's the team or if it's the hospital or, I don't know, or the combination. I have no idea. But I'm super-happy and feeling lucky I ended up like having all this. like how many people get the support we get here? Is it just me because I have mental health?' (CBM10)

Timing of access to maternity services

Quantitative Analysis 1- Model of care

The quantitative data were analysed to test the hypothesis that the specialist model of care has an impact on the timing of access to maternity care, known as the 'booking appointment'. Table 4 shows that no relationship was found between the model of care received and the gestation at which women attended their booking appointment. The base outcome was set for less than 10 weeks' gestation at the booking appointment to reflect the NICE guidance for women with complex social factors [15]. When adjusting for women's characteristics (see supplementary file 2 for fully adjusted outcome data tables) primiparous women (RR 1.79 CI 1.02-3.12), those with social risk factors (RR 1.93 CI 1.03-3.62), and those with high medical risk status at the booking appointment (RR 2.49 CI 1.18-5.25) were more likely to book for maternity care later than 20 weeks.

For peer review only - http://bmjopen.bmj.com/site/about/guidelines.xhtml

Table 4:Gestation at booking appointment in relation to the model of care received

Gestation at booking	Model of Care	Number of women (%)	Unadjusted RR (95% CI)	Model 1 Adjusted RR (95% CI) *	Model 2 Adjusted RR (95% CI) **	Model 3 Adjusted RR (95% CI) ***
<10 weeks	Standard	197(62)	Ref	Ref	Ref	Ref
	Group	87(27)	Ref	Ref	Ref	Ref
	Specialist	35(11)	Ref	Ref	Ref	Ref
10-12	Standard	166(58)	1.01(0.59-1.73)	0.91 (0.52-1.61)	0.77(0.42-1.40)	0.76(0.42-1.38)
	Group	90(32)	1.24 (0.70-2.12)	1.18 (0.65-2.16)	1.09(0.59-2.02)	0.86(0.46-1.63)
	Specialist	29(30)	Ref	Ref	Ref	Ref
13-20	Standard	53(61)	0.94 (0.43-2.02)	1.14 (0.50-2.61)	0.88 (0.37-2.09)	0.89(0.37-2.13)
	Group	24(28)	0.96 (0.41-2.22)	1.14 (0.47-2.77)	1.04 (0.42-2.56)	0.75(0.29-1.94)
	Specialist	10(11	Ref	Ref	Ref	Ref
> 20 weeks	Standard	53(68)	1.56 (0.62-3.92)	2.19 (0.82-5.80)	1.29(0.46-3.60)	1.27(0.45-3.56)
	Group	19(24)	1.27 (0.46-3.45)	1.75 (0.61-4.99)	1.29 (0.43-3.82)	1.15(0.38-3.44)
	Specialist	6(8)	Ref	Ref	Ref	Ref

* Model 1: Adjusted for demographics ethnicity, age, parity, IMD score, any social and medical risk factors at booking and onset of labour
** Model 2: Model 1 + Adjustment for place of antenatal care (community or hospital)
*** Model 3: Model 2 + Adjustment for service provider attended (A or B)

Quantitative Analysis 2- Place of antenatal care

Table 5 shows that after adjusting for the model of care received and service attended, women receiving hospital-based antenatal care were significantly more likely to attend their booking appointment after 20 weeks' gestation (RR 2.51 CI 1.33-4.70).

Table 5: Gestation at booking appointment in relation to the place of antenatal care

Gestation at booking	Place of antenatal care	Number of women (%)	Unadjusted RR	Model 1 Adjusted RR (95% CI) *	Model 2 Adjusted RR (95% CI) **	Model 3 Adjusted RR (95% CI) ***
<10	Hospital	154(48)	Ref	Ref	Ref	Ref
	Community	165(52)	Ref	Ref	Ref	Ref
10-13weeks	Hospital	154(54)	1.25(0.91-1.73)	1.19(0.85-1.66)	1.30(0.91-1.86)	1.00(0.67-1.49)
	Community	131(46)	Ref	Ref	Ref	Ref
13-20	Hospital	54(62)	1.75(1.07-2.84)	1.59(0.95-2.66)	1.66(0.97-2.87)	1.05(0.54-2.01)
	Community	33(38)	Ref	Ref	Ref	Ref
> 20 weeks	Hospital	56(72)	**2.72(1.58-4.67)**	**2.89(1.65-5.10)**	**2.81(1.56-5.06)**	**2.51(1.33-4.70)**
	Community	22(28)	Ref	Ref	Ref	Ref

* Model 1: Adjusted for demographics ethnicity, age, parity, IMD score, social risk, and medical risk factors at booking and onset of labour
** Model 2: Model 1 plus adjusted for model of care
*** Model 3: Model 2 plus Adjusted for service provider attended

Qualitative data analysis

For peer review only - http://bmjopen.bmj.com/site/about/guidelines.xhtml

The qualitative data were analysed to explore why timing of access to maternity care was different for women attending hospital-based antenatal care, primiparous women, and those with high medical risk status and social risk factors. Many women expressed wanting to be seen earlier in pregnancy and described not feeling valued by maternity services until their pregnancy is viable. Delays in accessing maternity services was often due to difficulties in accessing GP services and a convoluted referral pathway between community and hospital services. Community-based care and the ability to self-refer to maternity services appeared to reduce the time women spent waiting for their booking appointment.

'[at 3 weeks gestation] I called the GP to book an appointment. to say that I'm pregnant, and they sent me… to buy a Clearblue to check it because the list is too long. But it take a very long time, to see the GP… to refer me to the midwife, I was waiting a long time…when I saw the midwife the first time I was around 20 weeks. (CBM7)

Engagement - Number of antenatal appointments attended

Quantitative Analysis 1- Model of care

Women's engagement with services was tested through the number of antenatal appointments women attended. Table 6 shows no significant relationship between the model of care received and the number of antenatal appointments attended. Women with social risk factors were significantly more likely to have more than 15 antenatal appointments than those with no social risk factors (RR2.57 CI1.30-5.07), as were women with high medical risk (RR 2.70 CI 1.21-6.03)- See supplementary file 2.

Table 6: Number of antenatal appointments attended in relation to the model of care accessed

Number of antenatal appointments	Model of Care	Number of women (%)	Unadjusted RR	Model 1 Adjusted RR (95% CI) *	Model 2 Adjusted RR (95% CI) **	Model 3 Adjusted RR (95% CI) ***
1-6	Standard	173(63)	0.58 (0.31-1.09)	0.64 (0.35-1.30)	0.86 (0.44-1.69)	0.87(0.44-1.70)
	Group	63(23)	**0.41 (0.21-0.80)**	**0.42 (0.22-0.93)**	**0.43 (0.21-0.86)**	0.55(0.25-1.15)
	Specialist	37(14)	Ref	Ref	Ref	Ref
7-9****	Standard	135(61)	Ref	Ref	Ref	Ref
	Group	70(31)	Ref	Ref	Ref	Ref
	Specialist	17(8)	Ref	Ref	Ref	Ref
10-14	Standard	120(64)	0.94(0.45-1.95)	1.13(0.53-2.42)	0.80 (0.36-1.78)	0.82(0.37-1.81)
	Group	52(28)	0.78 (0.36-1.70)	0.90(0.40-2.02)	0.82 (0.36-1.86)	0.97(0.42-2.24)

For peer review only - http://bmjopen.bmj.com/site/about/guidelines.xhtml

For peer review only

	Specialist	16(8)	Ref	Ref	Ref	Ref
≥15	Standard	38(46)	0.47 (0.20-1.13)	0.68 (0.26-1.74)	0.43 (0.16-1.17)	0.43(0.16-1.14)
	Group	35(42)	0.85 (0.35-2.05)	1.25 (0.47-3.28)	1.02 (0.38-2.75)	1.28(0.47-3.45)
	Specialist	10(12	Ref	Ref	Ref	Ref

* Model 1: Adjusted for demographics ethnicity, age, parity, IMD score, any social and medical risk factors at booking and onset of labour
** Model 2: Model 1 + Adjustment for place of antenatal care (community or hospital)
*** Model 3: Model 2 + Adjustment for service provider attended (A or B)
**** Set as base as WHO recommends 8 antenatal appointments [1]

Quantitative Analysis 2- Place of antenatal care

Table 7 shows that women receiving hospital-based care were significantly likely to have less than the recommended number of appointments, [1,2,15] (RR0.61 CI 0.38-0.99), and much more likely to have over 15 appointments (RR4.90 CI2.50-9.61) than those receiving community-based care regardless of their medical risk status and other confounding factors.

Table 7: Number of antenatal appointments attended in relation to the place of antenatal care

Number of antenatal appointments	Model of Care	Number of women (%)	Unadjusted RR	Model 1 Adjusted RR (95% CI) *	Model 2 Adjusted RR (95% CI) **	Model 3 Adjusted RR (95% CI) ***
1-6	Hospital	106(39)	**0.53(0.37-0.77)**	**0.56(0.38-0.82)**	**0.47(0.31-0.71)**	**0.61(0.38-0.99)**
	Community	167(61)	Ref	Ref	Ref	Ref
7-9****	Hospital	120(54)	Ref	Ref	Ref	Ref
	Community	102(46)	Ref	Ref	Ref	Ref
10-14	Hospital	130(69)	**1.90(1.26-2.86)**	**2.22(1.44-3.41)**	**2.27(1.43-3.58)**	**2.70(1.62-4.49)**
	Community	58(31)	Ref	Ref	Ref	Ref
≥15	Hospital	60(72)	**2.21(1.28-3.83)**	**2.92(1.59-5.34)**	**3.36(1.80-6.25)**	**4.90(2.50-9.61)**
	Community	23(28)	Ref	Ref	Ref	Ref

* Model 1: Adjusted for demographics ethnicity, age, parity, IMD score, social risk, and medical risk factors at booking and onset of labour
** Model 2: Model 1 plus adjusted for model of care
*** Model 3: Model 2 plus Adjusted for service provider attended
**** Set as base as WHO recommends 8 antenatal appointments [1]

Qualitative analysis

Flexible care was discussed by women accessing both specialist models, in terms of when and where appointments were scheduled and how long each appointment lasted. The community or home setting was seen as more convenient and supportive, particularly for those who were unfamiliar with UK transport systems, and those with little resource or young children. Women from both specialist models

For peer review only - http://bmjopen.bmj.com/site/about/guidelines.xhtml

expressed ease of contacting the specialist model midwives through phone call, text messaging or email, felt that it reduced the number of face-to-face appointments they needed and that they could rely on the midwives to remind them of appointments. Women who did not speak fluent English (45%) particularly valued the ability to text their midwife as it gave them the opportunity to use translation technology. This flexibility appeared to encourage women to seek help more readily when they were concerned or needed reassurance. However, women in the hospital-based model did not always feel that the way care was scheduled suited their needs but structured around the hospital protocol and organisational efficiency.

If it's [antenatal care] like near to me it's OK but… I have so much financial problems so when she [HBM midwife] came to my house like, so much easier for me….and at the beginning I didn't know how to use bus... she helped me. (HBM6)

The quality of relational continuity and missed appointments

Quantitative Analysis 1- Model of care

The quantitative data were analysed to test the hypothesis that the specialist model of care increases the quality of relational continuity through the number of antenatal appointments and care in labour from a known healthcare professional (midwife, GP, or obstetrician), and reduces the number of appointments women miss, or do not attend. Table 8 shows that women receiving group practice and specialist models were significantly more likely to see a known healthcare professional during pregnancy. When adjusting the model for women's characteristics Black African women were the least likely group to see a known healthcare professional more than 5 times (RR0.17 CI 0.03-0.82). Women receiving care in the specialist model were significantly more likely to be looked after in labour by a known healthcare professional than the group practice (RR0.44 CI0.21-0.92).

Table 8: Number of appointments and support in labour by known healthcare professional

Number of antenatal appointments with a known professional	Model of Care	Number of women (%)	Unadjusted OR	Model 1 Adjusted RR (95% CI) *	Model 2 Adjusted RR (95% CI) **	Model 3 Adjusted RR (95% CI) ***
None	Standard	313(69)	Ref	Ref	Ref	Ref
	Group	84(19)	Ref	Ref	Ref	Ref
	Specialist	53(12)	Ref	Ref	Ref	Ref

**1-2**	Standard	108(73)	**6.09(1.86-19.9)**	**4.43(1.30-15.0)**	2.75(0.78-9.67)	2.12(0.57-7.87)
	Group	36(24)	**7.57(2.21-25.8)**	**7.30(2.05-26.0)**	6.81(1.89-24.5)	1.81(0.44-7.45)
	Specialist	3(2)	Ref	Ref	Ref	Ref
**3**	Standard	36(47)	0.76(0.33-1.72)	0.69(0.27-1.77)	0.47 (0.17-1.29)	0.34(0.11-1.06)
	Group	32(42)	**2.52(1.08-5.89)**	**3.33(1.27-8.75)**	**3.15 (1.18-8.38)**	0.82(0.24-2.75)
	Specialist	8(11)	Ref	Ref	Ref	Ref
**4**	Standard	7(15)	0.29(0.83-1.04)	**0.23 (0.05-0.89)**	**0.16(0.03-0.67)**	**0.10(0.02-0.53)**
	Group	34(76)	**5.36(1.80-15.9)**	**5.99(1.78-20.1)**	**5.19(1.62-18.7)**	1.72(0.39-1.47)
	Specialist	4(9)	Ref	Ref	Ref	Ref
**>5**	Standard	5(10)	**0.76(0.02-0.23)**	**0.05(0.01-0.19)**	**0.03 (0.00-0.13)**	**0.02(0.00-0.11)**
	Group	35(68)	2.00(0.93-4.29)	2.50(0.96-6.49)	2.38 (0.90-6.32)	0.82(0.23-2.94)
	Specialist	11(22)	Ref	Ref	Ref	Ref
**Looked after in labour by a known midwife**	Standard	235(63)	**0.41(0.24-0.71)**	**0.44(0.24-0.82)**	0.62 (0.32-1.19)	0.59(0.30-1.17)
	Group	81(23)	**0.24(0.13-0.42)**	**0.23 (0.12-0.44)**	**0.24 (0.12-0.47)**	**0.44(0.21-0.92)**
	Specialist	53(14)	Ref	Ref	Ref	Ref

* Model 1: Adjusted for demographics ethnicity, age, parity, IMD score, any social and medical risk factors at booking and onset of labour

** Model 2: Model 1 + Adjustment for place of antenatal care (community or hospital)

*** Model 3: Model 2 + Adjustment for service attended (A or B)

For peer review only

Quantitative Analysis 2- Place of antenatal care

Table 9 shows that women receiving hospital-based antenatal care were less likely to see a known healthcare professional for their antenatal appointments compared to those receiving community-based care. No significant relationship was found between the place of care and the number of women cared for in labour by their named midwife.

For peer review only - http://bmjopen.bmj.com/site/about/guidelines.xhtml

Table 9: Number of appointments with a known healthcare professional in relation to place of care

Number of antenatal appointments with a known professional	Model of Care	Number of women (%)	Unadjusted OR	Model 1 Adjusted RR (95% CI) *	Model 2 Adjusted RR (95% CI) **	Model 3 Adjusted RR (95% CI) ***
None	Hospital	215(48)	Ref	Ref	Ref	Ref
	Community	235(52)	Ref	Ref	Ref	Ref
1-2	Hospital	109(74)	**3.15 (2.08-4.76)**	**2.44(1.56-3.83)**	**2.71(1.63-4.50)**	**0.24(0.10-0.59)**
	Community	38(26)	Ref	Ref	Ref	Ref
3	Hospital	45(59)	1.59 (0.97-2.61)	1.23(0.71-2.13)	**2.25(1.19-4.25)**	**0.23(0.08-0.62)**
	Community	31(41)	Ref	Ref	Ref	Ref
4	Hospital	20(44)	0.87 (0.47-1.62)	0.61 (0.31-1.22)	2.02 (0.92-1.47)	**0.30(0.11-0.83)**
	Community	25(56)	Ref	Ref	Ref	Ref
>5	Hospital	28(55)	1.33 (0.74-2.39)	0.70 (0.35-1.39)	**2.66 (1.24-5.70)**	**0.35(0.12-0.96)**
	Community	23(45)	Ref	Ref	Ref	Ref
Looked after in labour by a known midwife	Hospital	153(41)	**0.34(0.25-0.46)**	**0.46(0.33-0.64)**	**0.39(0.27-0.57)**	0.89(0.59-1.36)
	Community	216(59)	Ref	Ref	Ref	Ref

* Model 1: Adjusted for demographics ethnicity, age, parity, IMD score, social risk, and medical risk factors at booking and onset of labour
** Model 2: Model 1 plus adjusted for model of care
*** Model 3: Model 2 plus Adjusted for service attended

Missed appointments

Quantitative Analysis 1- Model of care

Table 10 shows that no significant relationship was found between model of care and the number of missed appointments. When adjusting for women's characteristics multiparous women were 4 times more likely (RR 4.50 CI 1.13-17.82) to miss 4 or more appointments then primiparous women, and the older a woman was the less likely she was to miss 4 or more appointments (RR0.03 CI 0.00-0.55). Black African women (RR 12.85 CI 2.42-68.07), and women with social risk factors (RR2.26 CI 1.14-4.47) were more likely to miss two or more appointments (See supplementary file 2). These findings should be viewed with caution due to the wide confidence intervals.

For peer review only - http://bmjopen.bmj.com/site/about/guidelines.xhtml

Table 10: Number of missed appointments in relation to model of care received

Number of missed appointments	Model of Care	Number of women (%)	Unadjusted OR	Model 1 Adjusted RR (95% CI) *	Model 2 Adjusted RR (95% CI) **	Model 3 Adjusted RR (95% CI) ***
None	Standard	352(63)	Ref	Ref	Ref	Ref
	Group	147(26)	Ref	Ref	Ref	Ref
	Specialist	62(11)	Ref	Ref	Ref	Ref
1	Standard	62(56)	1.36 (0.62-2.99)	1.58 (0.67-3.67)	1.46 (0.61-3.48)	1.44(0.61-3.49)
	Group	40(37)	2.10 (9.33-4.76)	**2.41 (1.01-5.77)**	2.38 (0.99-5.72)	1.97(0.80-4.84)
	Specialist	8(7)	Ref	Ref	Ref	Ref
2	Standard	37(67)	1.62 (0.56-4.73)	2.27 (0.72-7.13)	1.79(0.54-5.84)	1.81(0.53-6.19)
	Group	14(26)	1.47 (0.46-4.66)	1.83 (0.54-6.20)	1.79 (0.52-6.14)	0.86(0.22-3.33)
	Specialist	4(7)	Ref	Ref	Ref	Ref
3	Standard	14(52)	1.23 (0.27-5.55)	2.71 (0.33-22.0)	2.01 (0.23-17.2)	2.02(0.22-18.4)
	Group	11(41)	2.31 (0.49-10.7)	4.54 (0.54-37.9)	4.41 (0.51-37.6)	2.03(0.25-23.6)
	Specialist	2(7)	Ref	Ref	Ref	Ref
≥4	Standard	4(24)	0.17(0.42-0.72)	**0.20 (0.04-0.98)**	0.22 (0.04-1.14)	0.23(0.04-1.22)
	Group	9(53)	0.94(0.28-3.19)	0.94 (0.22-4.02)	0.94 (0.21-4.05)	0.49(0.08-2.71)
	Specialist	4(23)	Ref	Ref	Ref	Ref

* Model 1: Adjusted for demographics ethnicity, age, parity, IMD score, any social and medical risk factors at booking and onset of labour

** Model 2: Model 1 + Adjustment for place of antenatal care (community or hospital)

*** Model 3: Model 2 + Adjustment for service provider attended (A or B)

Analysis 2- Place of antenatal care

Table 11 shows no significant relationship was found between the number of missed appointments and the place of antenatal care.

For peer review only - http://bmjopen.bmj.com/site/about/guidelines.xhtml

Table 11: Number of missed appointments in relation to place of care

Number of missed appointments	Place of Care	Number of women (%)	Unadjusted OR	Model 1 Adjusted RR (95% CI) *	Model 2 Adjusted RR (95% CI) **	Model 3 Adjusted RR (95% CI) ***
None	Hospital	292(52)	Ref	Ref	Ref	Ref
	Community	269(48)	Ref	Ref	Ref	Ref
1	Hospital	64(58)	1.28 (0.84-1.93)	1.11 (0.71-1.72)	1.21 (0.76-1.94)	0.95(0.56-1.62)
	Community	46(42)	Ref	Ref	Ref	Ref
2	Hospital	38(69)	**2.06 (1.13-3.74)**	**2.07 (1.12-3.88)**	**1.96 (1.01-3.78)**	0.64(0.26-1.58)
	Community	17(31)	Ref	Ref	Ref	Ref
3	Hospital	18(67)	1.84 (0.81-4.17)	1.82 (0.74-4.46)	2.18 (0.83-5.67)	1.02(0.32-3.20)
	Community	9(33)	Ref	Ref	Ref	Ref
≥4	Hospital	6(35)	0.50 (0.18-1.38)	0.48 (0.15-1.48)	0.77(0.23-2.51)	0.36(0.08-1.55)
	Community	11(65)	Ref	Ref	Ref	Ref

* Model 1: Adjusted for demographics ethnicity, age, parity, IMD score, social risk, and medical risk factors at booking and onset of labour
** Model 2: Model 1 plus adjusted for model of care
*** Model 3: Model 2 plus Adjusted for service provider attended

Qualitative analysis

Women accessing the community-based model perceived a high level of continuity of care from the whole team. Women accessing the hospital-based model discussed how they knew their named midwife but not necessarily the rest of the team, impacting on how they sought help. They were also not always aware of the level of continuity offered, expressed more anxiety about their labour care and discussed the impact of midwives needing to cancel appointments. For some, cancelled appointments and a lack of continuity had a significant impact on their support and engagement, leading to social care involvement. Women in both models described feeling comfortable when rebooking appointments, they were unable to attend that appeared to reduce the number of 'missed' appointments recorded, seen as a mitigating effect of the specialist model.

'if I'm running late or something comes up I just text her or give her a call and she'll muddle things around' (HBM9)

For peer review only - http://bmjopen.bmj.com/site/about/guidelines.xhtml

Summary of findings

Table 12 summarises the quantitative findings in relation to either the model of care received, or the place of antenatal care, or both. Characteristics of women at disproportionate risk and differences between the services providers is also presented.

Table 12: Overview of quantitative outcomes

Access and Engagement Outcome variable	Characteristics of women at disproportionate risk when adjusting (Supplementary file 2)	Significant effect of specialist model of care	Significant effect of hospital based antenatal care	Significant effect of service
Access to specialist model	Most deprived, social risk factors, Black African ethnicity	↑	↓	=
Late gestation at booking appointment (>20/40)	Primiparous, High medical risk, social risk factors	=	↑	=
Number of antenatal appointments outside of recommendations [1,2]	Social risk factors and high medical risk status (> 15 appointments)	=	↓/↑	B↓
Appointments with a known healthcare professional	Black African (least likely to see a known HCP)	=	↓	B↓
Missed appointments	Multiparous, Black African, social risk	=	=	B↑

↑= Statistically significant increase (p< 0.05)

↓ = Statistically significant decrease (p < 0.05)

= No significant relationship detected

'A' and 'B' refer to service providers

Refined programme theory

For peer review only - http://bmjopen.bmj.com/site/about/guidelines.xhtml

The qualitative interviews with women enabled the refinement of previously constructed programme theories [7,25], giving insight into specific, underlying mechanisms of improved access and engagement with maternity services. The process of refinement using the data analysed is detailed in Supplementary file 2. The CMO configurations are detailed in Table 13, providing a framework for those developing future models of care for women with social risk factors.

Table 13: Refined programme theory – Access and engagement with maternity services

**Context +**	**Mechanism =**	**Outcome**
Women who struggle to access services and are at greater risk of booking for maternity care at a late gestation.<br><br>Women who are unfamiliar of the UK health system or have difficulties in registering with health services. These women are often experiencing social risk factors that might lead to chaotic lives, social isolation, lack of resource, lack of support.<br><br>Primiparous women, those with any social risk factor, and high medical risk are more likely to book late for maternity care. Multiparous women, Black African women and those with social risk factors are more likely to miss appointments. Black African women have less appointments with a known healthcare professional.	M1) If maternity care provision commences when a woman accesses services regardless of her gestation and women have 24/7 access to a small team of midwives whom they have had the opportunity to meet during pregnancy and are encouraged to contact via a phone call, text message or free technology	O1) Then women would not feel unsupported, anxious, and that the service does not value them until they have a viable pregnancy. This might also improve early access to safe abortion and family planning services. Engagement with services will improve through needs-based communication and appropriately timed antenatal appointments. This open access can work both ways through midwives reminding them of appointments, this leads to women feeling more 'cared for'.
	M2) If women are made aware of the possibility and how to self-refer to maternity services and specialist models of care by administrative staff at the first point of contact	O2)Then difficulties trying to access a GP will be overcome, the time spent waiting for a GP appointment reduced, and long referral processes between primary and secondary services will be avoided.
	M3) If women can access a community-based service where GP's and midwives regularly communicate with each other	O3) Then the timing of access to a booking appointment with a midwife will be improved and convoluted referral pathways between community and hospital services avoided.
	M4) If women living in areas of deprivation are prioritised to receive continuity of care through community-based models	O4) Then services are likely to provide an enhanced package of care to women with social risk factors who have not previously disclosed these issues with professionals, and care is less likely to be disrupted during pregnancy when a disclosure is made.
	M5) If women are informed of the reasons why they have been allocated a continuity of care model or specialist service, or are able to self-refer to them if they feel they are eligible for their care	O5) Then the development of a trusting and open relationship with their healthcare provider will be enabled and feelings of suspicion and surveillance reduced. This transparency may also reduce feelings of discrimination for women in marginalised groups.
	M6) If women who book late for pregnancy care are eligible for specialist models of care where they have a named midwife or small team of midwives	O6) Then the benefits of these models of care and highlighted mechanisms may protect them from the disproportionately poor outcomes associated with late booking.
	M7) If women can reschedule appointments easily, and do not fear judgement or reproach when they miss appointments	O7) then they will perceive the maternity environment as a place of safety and their engagement with flexible services will improve.

For peer review only - http://bmjopen.bmj.com/site/about/guidelines.xhtml

	M8)If women have the opportunity to get to know all the midwives in a small team throughout their pregnancy and the number, time and place of appointments is co-planned with women to meet their individual needs	O8) then they will not feel disappointment or let down when their named midwife is unable to attend an appointment. Care, information, and responsibility will be shared across the team, women will be better able to engage, not perceive the pressure of time and feel more able to seek help and disclose information thus improving safety.

Discussion

In response to concerns that the most affluent and lowest risk women are often the most likely to receive the highest standards of maternity care [38], a clear example of Tudor Harts Inverse Care Law [39], this study examines a shift change to more proportionate universalism through services targeting those who are at highest risk of poor outcomes. The aims of the specialist models of care- to reach those who are most at risk of poor outcomes and experiences, are being met. This is an important finding as it addresses the aims of the NHS Ten Year plan [40] to improve outcomes and care experiences through the offer of continuity of care to Black and minority ethnic women and those living in deprived areas. The findings suggest that placing continuity of care models in areas of deprivation are likely to identify women who are experiencing social risk factors during pregnancy, thus providing them with an enhanced level of care whether they have disclosed risk factors or not. In contrast, strict inclusion criteria to hospital-based models risk missing those women who are at increased risk but are yet to disclose social risk factors, leading to fragmented care and further risk of 'falling through the gaps'. The wider Project20 evaluation tests the theory that women will feel more able to disclose risk factors when they have developed a trusting relationship with their care provider (paper currently under review; WOMBI-D-22-00309R2)

Overall women found the referral to both specialist models of care acceptable, but some expressed a lack of choice, and perceived discrimination about being left in the dark for the reason for referral. There is a wealth of literature around healthcare professionals reluctance to address sensitive issues such as mental health, social risk factors and cultural differences with women [41–4341,43–49]. In addition to this, socioeconomic status and ethnicity are often associated with stigma and discrimination in healthcare services [50–53]. These factors might contribute to midwives' feelings of discomfort around informing women why they have been referred to a specialist model. Perceived discrimination could be avoided

For peer review only - http://bmjopen.bmj.com/site/about/guidelines.xhtml

through community-based care within areas of deprivation rather than inclusion criteria based on these social risk factors.

The findings showed no differences between the model of care received and timing of access to maternity care or the number of antenatal appointments women attended. Considering women in the specialist models were more likely to have low socioeconomic status and social risk factors and therefore more likely to struggle to access and engage [54–56] this indicates that the inequality in access may have been mitigated by the specialist model of care. This theory would need to be tested with a larger sample. Regardless of the model of care women received, those receiving hospital-based care were more likely to attend their first maternity appointment after 20 weeks' gestation, this was thought to be partly due to convoluted referral systems between the community and hospital. Many women expressed wanting to be seen earlier in pregnancy, challenging the notion that women with social risk factors do not prioritise their maternity care [57]. Women also described difficulties in registering or booking an appointment with their GP when they found out they were pregnant, particularly if they did not speak English, were homeless, or unfamiliar with the system. The wider literature has identified similar barriers to access including difficulties navigating the health system and service delays in the processing of referrals [58–60]. A UK study [61] of women accessing antenatal care in a multicultural, deprived area found that women want to access care in early pregnancy but perceive antenatal care for viable and continuing pregnancies at a later gestation. If women do not feel that a service is open to them, or that maternity services only value those who carry a viable pregnancy, they may internalise this as a prioritisation of the wellbeing of the fetus over their own emotional, physical, and social needs. The wider Project20 evaluation previously tested programme theory relating to interpreter services for pregnant women with social risk factors, finding that despite accessing a specialist model of care women experienced a lack of regulation and access to high-quality interpretation services [62] . As well as impacting on women's engagement with services, these factors can impact on the safety of women with complex needs such as those who are experiencing abuse, poor mental health or need to discuss a termination of pregnancy. Previous research has found a correlation between continuity models of care and increased disclosure and referral to support services [63 64], but it must be acknowledged that under ascertainment of sensitive issues such as mental health and domestic abuse remains likely whilst women perceive services as a form of surveillance and risk [64].

A recent review of the literature on how women with social risk factors experience maternity care in the UK found reasons for late access include the denial of services based on a lack of documentation, fear of

For peer review only - http://bmjopen.bmj.com/site/about/guidelines.xhtml

disclosure to immigration services, language and financial barriers, cultural differences, unfamiliarity, a lack of trust, and a perception that maternity services act as a system of surveillance rather than support [65]. Practice recommendations detailed in the refined programme theory suggest that all women are made aware of the possibility to self-refer directly to maternity services at the first point of contact with health services, using language appropriate information. Strict inclusion criteria for access to specialist models that restrict women who have booked late for maternity care should be reviewed as these women are often high risk and can benefit from the specialist model regardless of their gestation. An evaluation of group antenatal care [66] included women who had booked later in pregnancy with positive feedback and no negative impact on group dynamics. With the national expansion of continuity of care models [18], inclusion criteria's could be relaxed somewhat as the demands on the service are more evenly distributed. Future research should assess the impact of place-based midwifery continuity of care on health inequalities to test the outcomes of this preference.

This study reflects findings in the wider literature [56,67] highlighting service use inequities for women with characteristics such as multiparity, age, and ethnicity and presents an opportunity for those designing models of care to focus on these demographics. Where poor engagement with services is often associated with women's priorities and behaviours [7,60,68], the women in this study highlighted system barriers. Interestingly, many women in this study felt that the ability to contact a known midwife anytime reduced the number of face-to-face appointments they needed. Where engagement in this study was measured through the number of antenatal appointments attended, it would be useful for future research to measure other forms of contact between women and healthcare professionals. Women from the hospital-based specialist model discussed the detrimental impact of midwives needing to cancel their appointments. Rather than a place-based issue this seems to relate to how the teams are organised as those in the hospital-based model were allocated one midwife who they saw for most of their appointments, whereas women accessing the community-based model described being cared for by the whole team. Finally, women from both community and hospital-based models expressed a preference for care to be based in the community or home setting as they felt it was more accessible and supportive of their needs.

The aim of both the group practice and specialist models evaluated appear to be being met with women more likely to receive more antenatal appointments with a known healthcare professional from these models. Women who received hospital-based antenatal care had significantly lower levels of continuity in the antenatal period. Women receiving care in the specialist model were more likely to be looked after in

For peer review only - http://bmjopen.bmj.com/site/about/guidelines.xhtml

labour by a known healthcare professional compared to the other models. The qualitative data revealed this continuity of care lessened anxiety, the need to repeat often complex social and medical histories to numerous professionals, and increased disclosure of social risk factors, and preparedness for labour and birth, particularly for those who had the opportunity to meet the rest of the team. Multiparous, younger, and Black African women were more likely to miss antenatal appointments, as well as those with social risk factors. Black African women were also less likely to see a known healthcare professional than women of other ethnicities, perhaps reflecting an underlying mechanism of the health inequalities they experience [69]. There is a significant paucity of literature around Black women's experiences of UK maternity care, but a US review [70] concluded that midwifery care can accommodate their specific needs through attentive provider, continuity of care, and empowerment. The review stated that '*researchers must meet, consult with, and listen to Black women and hear their stories to understand the significance of midwifery to them. Only then, in partnership with, and based on suggestions for change from Black women themselves, can health care providers and researchers begin to make changes in the health care system to facilitate improved antenatal care'* [70]. This recommendation is as relevant to the UK context given the known stark inequalities.

Strengths and limitations

Women interviewed during the study may have perceived the study questions to be testing them about their willingness to engage with their care. This limitation may have been lessened through the trust built between the participant and the researcher over the course of the longitudinal interviews [71,72]. The small and varied numbers in each quantitative data group should be taken into consideration due to the significant amount of multiple testing required to establish the separate effects of the model of care, place of care and service attended. This presents a potential limitation as the use of multiple testing can result in erroneous inferences, reducing the probability of detecting effects when they do exist [73]. This could be overcome in future research using larger sample sizes, and Bayesian analysis to test the apparent mitigating effects of the specialist models of care on inequalities in access and engagement [74] . That said, the claim to the causality of the mechanisms is strengthened as both qualitative and quantitative data analysis point to a causal link between the mechanism(s) and the outcomes studied [74].

The analysis of quantitative data is also limited by the availability and depth of information routinely recorded in maternity services. It is recommended that more granular ethnicity categories, using the ONS 18+ guidance [75] is used, as well as migration details such as country of birth, national identify, length of time in the UK, visa conditions and language proficiency. Finally, the generalisability of the

For peer review only - http://bmjopen.bmj.com/site/about/guidelines.xhtml

findings is limited by the urban location of both specialist models of care evaluated and the UK's health system context. This is particularly significant when reflecting on the outcomes relating to place-based care- what may have yielded significant outcomes in a densely populated, multicultural community, may yield very different results elsewhere. Research is needed to test the generalisability of the findings to rural and other community settings.

Conclusion

This research highlights how carefully considered place-based care with a focus on continuity of carer can improve access and engagement with maternity services for women with social risk factors, but it is not a panacea. Hospital-based models of care with strict inclusion criteria may risk excluding women at increased risk who are yet to disclose social risk factors. Rather than the often-assumed 'relaxed maternal care-seeking behaviour' and 'women with social risk factors deprioritising maternity care' explanations for late booking and missed appointments, the findings highlight system barriers. Women want to be able to access care earlier in pregnancy, and there appears to be a lack of information regarding their choices in doing so, particularly for those who do not speak English or are unfamiliar with the system. The identification of specific mechanisms that improve access and engagement with services are outlined in the refined programme theory, which will enable those developing maternity services to structure models of care around women's individual need.

Availability of data and material- full quantitative data analysis attached as additional file or contact the lead author HRJ for qualitative and raw data files that are available upon reasonable request.

Ethical approval- The research was approved by the London Brent Research Ethics Committee (HRA) REC Reference 18-LO-0701.

Competing interests - None declared

Funding- This report is independent research supported by the National Institute for Health Research (NIHR Doctoral Research Fellowship, Hannah Rayment-Jones, award no DRF-2017-10-033). Angela Harden is supported by the NIHR Applied Research Collaboration North Thames. Jane Sandall (King's

For peer review only - http://bmjopen.bmj.com/site/about/guidelines.xhtml

College London) is supported by the NIHR Applied Research Collaboration South London [NIHR ARC South London]. Jane Sandall is also an NIHR Senior Investigator. James Harris is supported by a Post-doctoral Fellowship from Wellbeing of Women (Award Ref PRF006). Kathryn Dalrymple is funded by the MRC (grant number: MR/V005839/1)The views expressed in this article are those of the author(s) and not necessarily those of the NHS, the NIHR, the MRC, or the Department of Health and Social Care.

Authors' contributions- Authors HRJ, JH, AH, JS contributed to the conceptualisation of the research question, and methodology. Authors EP and TG organised collection and anonymisation of the quantitative data, HRJ and KD analysed the quantitative data. HRJ collected qualitative data and analysed with JH. All authors interpreted the data analysis, read and approved the final manuscript.

Acknowledgements- The authors would like to thank student midwives Laura Peazold, Mary Newman, Natalie Goodyear and Micaela Anthony for help with data collection and anonymisation, Justin Jagosh for invaluable advice on realist methodology and Sergio Silverio for support with qualitative data analysis.

Figure legends: Figure 1: Programme theory refinement process
Figure 2: Quantitative data collection flowchart

Word count- 4793

For peer review only

References

1. World Health Organization (WHO). WHO Recommendations on Antenatal Care for a Positive Pregnancy Experience: Summary. *World Health Organization* (2018) doi:10.1186/1742-4755-10-19.5.
2. National Institute for Clinical Excellence (NICE). *Antenatal care. Quality standard [QS22].* (2012).
3. Nam, J. Y., Cho, E. & Park, E. C. Do severe maternal morbidity and adequate prenatal care affect the delivery cost? A nationwide cohort study for 11 years with follow up. *BJOG* (2019) doi:10.1111/1471-0528.15895.
4. Renfrew, M. J. *et al.* Midwifery and quality care: Findings from a new evidence-informed framework for maternal and newborn care. *The Lancet* Preprint at https://doi.org/10.1016/S0140-6736(14)60789-3 (2014).
5. Shaw, D. *et al.* Drivers of maternity care in high-income countries: can health systems support woman-centred care? *The Lancet* Preprint at https://doi.org/10.1016/S0140-6736(16)31527-6 (2016).
6. Lindquist, A., Kurinczuk, J. J., Redshaw, M. & Knight, M. Experiences, utilisation and outcomes of maternity care in England among women from different socio-economic groups: Findings from the 2010 National Maternity Survey. *BJOG* (2015) doi:10.1111/1471-0528.13059.
7. Rayment-Jones, H., Harris, J., Harden, A., Khan, Z. & Sandall, J. How do women with social risk factors experience United Kingdom maternity care? A realist synthesis. *Birth* **46**, 461–474 (2019).
8. Downe, S., Finlayson, K., Tunçalp & Metin Gülmezoglu, A. What matters to women: A systematic scoping review to identify the processes and outcomes of antenatal care provision that are important to healthy pregnant women. *BJOG: An International Journal of Obstetrics and Gynaecology* Preprint at https://doi.org/10.1111/1471-0528.13819 (2016).
9. Finlayson, K. & Downe, S. Why Do Women Not Use Antenatal Services in Low- and Middle-Income Countries? A Meta-Synthesis of Qualitative Studies. *PLoS Med* (2013) doi:10.1371/journal.pmed.1001373.
10. Arsenault, C. *et al.* Equity in antenatal care quality: an analysis of 91 national household surveys. *Lancet Glob Health* (2018) doi:10.1016/S2214-109X(18)30389-9.
11. Nair, M., Knight, M. & Kurinczuk, J. J. Risk factors and newborn outcomes associated with maternal deaths in the UK from 2009 to 2013: a national case–control study. *BJOG* (2016) doi:10.1111/1471-0528.13978.
12. Amjad, S., Chandra, S., Osornio-Vargas, A., Voaklander, D. & Ospina, M. B. Maternal Area of Residence, Socioeconomic Status, and Risk of Adverse Maternal and Birth Outcomes in Adolescent Mothers. *Journal of Obstetrics and Gynaecology Canada* (2019) doi:10.1016/j.jogc.2019.02.126.
13. Knight, Marian., Bunch, Kathryn., Kenyon, Sara., Tuffnell, Derek., Kurinczuk, Jennifer J., Shakespear, Judy., Kotnis, Rohit. *Saving Lives, Improving Mothers' Care. Lessons learned*

For peer review only - http://bmjopen.bmj.com/site/about/guidelines.xhtml

to inform maternity care from the UK and Ireland Confidential Enquiries into Maternal Deaths and Morbidity 2015-17. (2019).

14. Ki-Moon, B. Global strategy for women's and children's health. in *Maternal Mortality, Human Rights and Accountability* (2013). doi:10.4324/9780203684214.
15. (UK), N. C. C. for W. and C. H. *Pregnancy and Complex Social Factors. Pregnancy and Complex Social Factors: A Model for Service Provision for Pregnant Women with Complex Social Factors* (2010).
16. Organization, world health. WHO Recommendation on Antenatal care for positive pregnancy experience. *WHO Recommendation on Antenatal care for positive pregnancy experience* (2016) doi:ISBN 978 92 4 154991 2.
17. Kennedy, H. P. *et al.* Asking different questions: research priorities to improve the quality of care for every woman, every child. *The Lancet Global Health* Preprint at https://doi.org/10.1016/S2214-109X(16)30183-8 (2016).
18. NHS England. *The NHS Long Term Plan: Maternity and neonatal services*. (2019).
19. National Maternity Review. *Better Births: Improving outcomes of maternity services in England. National Maternity Review* (2016).
20. Sandall, J., Soltani, H., Gates, S., Shennan, A. & Devane, D. Midwife-led continuity models versus other models of care for childbearing women. *Cochrane Database of Systematic Reviews* Preprint at https://doi.org/10.1002/14651858.CD004667.pub5 (2016).
21. Hollowell, J., Kurinczuk, J. J., Oakley, L., Brocklehurst, P. & Gray, R. A systematic review of the effectiveness of antenatal care programmes to reduce infant mortality and its major causes in socially disadvantaged and vulnerable women. *National Perinatal Epidemiology Unit (NPEU)* (2009).
22. Pawson, R. *The Science of Evaluation: A Realist Manifesto*. *The Science of Evaluation: A Realist Manifesto* (2014). doi:10.4135/9781473913820.
23. Jagosh, J. Realist Synthesis for Public Health: Building an Ontologically Deep Understanding of How Programs Work, For Whom, and In Which Contexts. *Annu Rev Public Health* (2019) doi:10.1146/annurev-publhealth-031816-044451.
24. Rayment-Jones, H., Harris, J., Harden, A., Khan, Z. & Sandall, J. How do women with social risk factors experience United Kingdom maternity care? A realist synthesis. *Birth* (2019) doi:10.1111/birt.12446.
25. Rayment-Jones, H., Silverio, S. A., Harris, J., Harden, A. & Sandall, J. Project 20: Midwives' insight into continuity of care models for women with social risk factors: what works, for whom, in what circumstances, and how. *Midwifery* (2020) doi:10.1016/j.midw.2020.102654.
26. Jagosh, J. Retroductive theorizing in Pawson and Tilley's applied scientific realism. *J Crit Realism* (2020) doi:10.1080/14767430.2020.1723301.
27. Mukumbang, F. C., Kabongo, E. M. & Eastwood, J. G. Examining the Application of Retroductive Theorizing in Realist-Informed Studies. *Int J Qual Methods* **20**, 1–14 (2021).
28. Ogrinc, G. *et al.* SQUIRE 2.0 (Standards for QUality Improvement Reporting Excellence): revised publication guidelines from a detailed consensus process. *BMJ Qual Saf* **25**, 986–992 (2016).

For peer review only - http://bmjopen.bmj.com/site/about/guidelines.xhtml

29. Fernandez Turienzo, C. *et al.* Addressing inequities in maternal health among women living in communities of social disadvantage and ethnic diversity. *BMC Public Health* **21**, 1–5 (2021).
30. Murray, S. F., Buller, A. M., Bewley, S. & Sandall, J. Metrics for monitoring local inequalities in access to maternity care: Developing a basket of markers from routinely available data. *Qual Saf Health Care* (2010) doi:10.1136/qshc.2008.032136.
31. Ministry of Housing, C. & L. G. *English indices of deprivation 2019. National Statistics.* (2019).
32. Office for National Statistics. The English Indices of Deprivation 2015. *Neighbourhoods Statistical Release* (2015) doi:http://dx.doi.org/http://www.communities.gov.uk/publications/corporate/statistics/indices2010technicalreport.
33. Manzano, A. The craft of interviewing in realist evaluation. *Evaluation* (2016) doi:10.1177/1356389016638615.
34. Ministry of Housing Communities and Local Government. English indices of deprivation 2019. *National Statistics* (2019).
35. Prel, J.-B. du, Hommel, G., Röhrig, B. & Blettner, M. Confidence Interval or P-Value? Part 4 of a Series on Evaluation of Scientific Publications. *Deutsches Aerzteblatt Online* (2009) doi:10.3238/arztebl.2009.0335.
36. Gale, N. K., Heath, G., Cameron, E., Rashid, S. & Redwood, S. Using the framework method for the analysis of qualitative data in multi-disciplinary health research. *BMC Med Res Methodol* (2013) doi:10.1186/1471-2288-13-117.
37. Vasileiou, K., Barnett, J., Thorpe, S. & Young, T. Characterising and justifying sample size sufficiency in interview-based studies: Systematic analysis of qualitative health research over a 15-year period. *BMC Med Res Methodol* (2018) doi:10.1186/s12874-018-0594-7.
38. Kirkham, M., Stapleton, H., Curtis, P. & Thomas, G. The inverse care law in antenatal midwifery care. *Br J Midwifery* (2002) doi:10.12968/bjom.2002.10.8.10600.
39. Tudor Hart, J. THE INVERSE CARE LAW. *The Lancet* (1971) doi:10.1016/S0140-6736(71)92410-X.
40. Charles, A., Ewbank, L., McKenna, H. & Wenzel, L. The NHS long-term plan explained. . *Long read ;* (2019).
41. Fontein-Kuipers, Y. J., Budé, L., Ausems, M., de Vries, R. & Nieuwenhuijze, M. J. Dutch midwives' behavioural intentions of antenatal management of maternal distress and factors influencing these intentions: An exploratory survey. *Midwifery* (2014) doi:10.1016/j.midw.2013.06.010.
42. Everitt, L., Homer, C. & Fenwick, J. Working with Vulnerable Pregnant Women Who Are At Risk of Having their Babies Removed by the Child Protection Agency in New South Wales, Australia. *Child Abuse Review* (2017) doi:10.1002/car.2432.
43. Higgins, A., Carroll, M., Downes, C., Monahan, M., Gill, A., Madden, D., McGoldrick, E. & Nagle, U. Perinatal mental health: an exploration of practices, policies, processes and education needs of nurses and midwives within maternity and primary care services in Ireland. . *Dublin: Health Services Executive* (2017).
44. Baldwin, S. & Griffiths, P. Do specialist community public health nurses assess risk factors for depression, suicide, and self-harm among south asian mothers living in London?:

For peer review only - http://bmjopen.bmj.com/site/about/guidelines.xhtml

Special features: Clinical concepts. *Public Health Nurs* (2009) doi:10.1111/j.1525-1446.2009.00780.x.
45. Jomeen, J., Glover, L., Jones, C., Garg, D. & Marshall, C. Assessing women's perinatal psychological health: Exploring the experiences of health visitors. *J Reprod Infant Psychol* (2013) doi:10.1080/02646838.2013.835038.
46. Jane, R. Midwives, society and childbirth: debates and controversies in the modern period. *Womens Hist Rev* (1999) doi:10.1080/09612029900200394.
47. Everitt, L., Homer, C. & Fenwick, J. Working with Vulnerable Pregnant Women Who Are At Risk of Having their Babies Removed by the Child Protection Agency in New South Wales, Australia. *Child Abuse Review* (2017) doi:10.1002/car.2432.
48. Mulherin, K., Miller, Y. D., Barlow, F. K., Diedrichs, P. C. & Thompson, R. Weight stigma in maternity care: Women's experiences and care providers' attitudes. *BMC Pregnancy Childbirth* (2013) doi:10.1186/1471-2393-13-19.
49. Radcliffe, P. Substance-misusing women: Stigma in the maternity setting. *Br J Midwifery* (2011) doi:10.12968/bjom.2011.19.8.497.
50. Stepanikova, I. & Oates, G. R. Perceived Discrimination and Privilege in Health Care: The Role of Socioeconomic Status and Race. *Am J Prev Med* (2017) doi:10.1016/j.amepre.2016.09.024.
51. Tajeu, G. S. *et al.* 'We'll get to you when we get to you': Exploring potential contributions of health care staff behaviors to patient perceptions of discrimination and satisfaction. *Am J Public Health* (2015) doi:10.2105/AJPH.2015.302721.
52. Phelan, J. C., Lucas, J. W., Ridgeway, C. L. & Taylor, C. J. Stigma, status, and population health. *Soc Sci Med* (2014) doi:10.1016/j.socscimed.2013.10.004.
53. Walker, R. *The Shame of Poverty*. *The Shame of Poverty* (2014). doi:10.1093/acprof:oso/9780199684823.001.0001.
54. Hatherall, B. *et al.* Timing of the initiation of antenatal care: An exploratory qualitative study of women and service providers in East London. *Midwifery* (2016) doi:10.1016/j.midw.2016.02.017.
55. Rowe, R. E. *et al.* Social and ethnic differences in attendance for antenatal care in England. *Public Health* (2008) doi:10.1016/j.puhe.2008.05.011.
56. Lindquist, A., Kurinczuk, J. J., Redshaw, M. & Knight, M. Experiences, utilisation and outcomes of maternity care in England among women from different socio-economic groups: Findings from the 2010 National Maternity Survey. *BJOG* (2015) doi:10.1111/1471-0528.13059.
57. Haddrill, R., Jones, G. L., Mitchell, C. A. & Anumba, D. O. C. Understanding delayed access to antenatal care: A qualitative interview study. *BMC Pregnancy Childbirth* (2014) doi:10.1186/1471-2393-14-207.
58. Hollowell, Jennifer., Oakley C., Vigurs, E,. Barnett-Page, J. Kavanagh, and S. Oliver. Increasing the early initiation of antenatal care by black and minority ethnic women in the United Kingdom: a systematic review and mixed methods synthesis of women's views and the literature on intervention effectiveness. *Social Science Research Unit, Institute of Education, University of London* (2012).

For peer review only - http://bmjopen.bmj.com/site/about/guidelines.xhtml

59. Cresswell, J. A. *et al.* Predictors of the timing of initiation of antenatal care in an ethnically diverse urban cohort in the UK. *BMC Pregnancy Childbirth* (2013) doi:10.1186/1471-2393-13-103.
60. Hatherall, B. *et al.* Timing of the initiation of antenatal care: An exploratory qualitative study of women and service providers in East London. *Midwifery* (2016) doi:10.1016/j.midw.2016.02.017.
61. Hatherall, B. *et al.* Timing of the initiation of antenatal care: An exploratory qualitative study of women and service providers in East London. *Midwifery* (2016) doi:10.1016/j.midw.2016.02.017.
62. Rayment-Jones, H. *et al.* Project20: interpreter services for pregnant women with social risk factors in England: what works, for whom, in what circumstances, and how? *Int J Equity Health* **20**, 1–11 (2021).
63. Rayment-Jones, H., Murrells, T. & Sandall, J. An investigation of the relationship between the caseload model of midwifery for socially disadvantaged women and childbirth outcomes using routine data – A retrospective, observational study. *Midwifery* **31**, 409–417 (2015).
64. Rayment-Jones, H., Harris, J., Harden, A., Turienzo, C. F. & Sandall, J. Project20: Maternity care mechanisms that improve (or exacerbate) health inequalities. A realist evaluation. *Women and Birth* (2022) doi:10.1016/J.WOMBI.2022.11.006.
65. Rayment-Jones, H., Harris, J., Harden, A., Khan, Z. & Sandall, J. How do women with social risk factors experience United Kingdom maternity care? A realist synthesis. *Birth* (2019) doi:10.1111/birt.12446.
66. Hunter, L. J. *et al.* Better together: A qualitative exploration of women's perceptions and experiences of group antenatal care. *Women and Birth* (2019) doi:10.1016/j.wombi.2018.09.001.
67. Higginbottom, G. M. A. *et al.* Experience of and access to maternity care in the UK by immigrant women: A narrative synthesis systematic review. *BMJ Open* Preprint at https://doi.org/10.1136/bmjopen-2019-029478 (2019).
68. Docherty, A., Bugge, C. & Watterson, A. Engagement: An indicator of difference in the perceptions of antenatal care for pregnant women from diverse socioeconomic backgrounds. *Health Expectations* (2012) doi:10.1111/j.1369-7625.2011.00684.x.
69. MBRRACE-UK. *Saving Lives, Improving Mother's Care- Lay summary*. *Action against Medical Accidents)* (2018).
70. Yoder, H. & Hardy, L. R. Midwifery and Antenatal Care for Black Women: A Narrative Review. *Sage Open* (2018) doi:10.1177/2158244017752220.
71. Grinyer, A. & Thomas, C. The value of interviewing on multiple occasions or longitudinally. in *The SAGE Handbook of Interview Research: The Complexity of the Craft* (2012). doi:10.4135/9781452218403.n15.
72. Bhopal, K. & Deuchar, R. *Researching marginalized groups*. *Researching Marginalized Groups* (2015). doi:10.4324/9781315740782.
73. Miller, R. Multiple Comparisons-I. in *Encyclopedia of Statistical Sciences* (2006). doi:10.1002/0471667196.ess1701.pub2.
74. Ravn, R. Testing mechanisms in large-N realistic evaluations. *https://doi.org/10.1177/1356389019829164* **25**, 171–188 (2019).

For peer review only - http://bmjopen.bmj.com/site/about/guidelines.xhtml

75. Ethnic group, national identity and religion - Office for National Statistics. https://www.ons.gov.uk/methodology/classificationsandstandards/measuringequality/ethnicgroupnationalidentityandreligion.

For peer review only

For peer review only

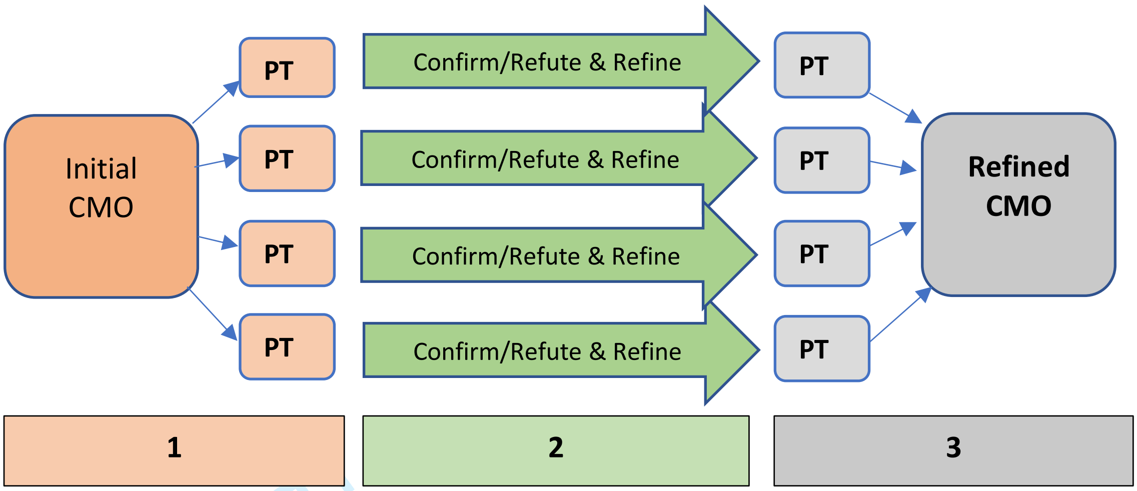

Figure 1: Programme theory (PT) refinement process leading to Context+Mechanism=Outcome (CMO) configuration

For peer review only

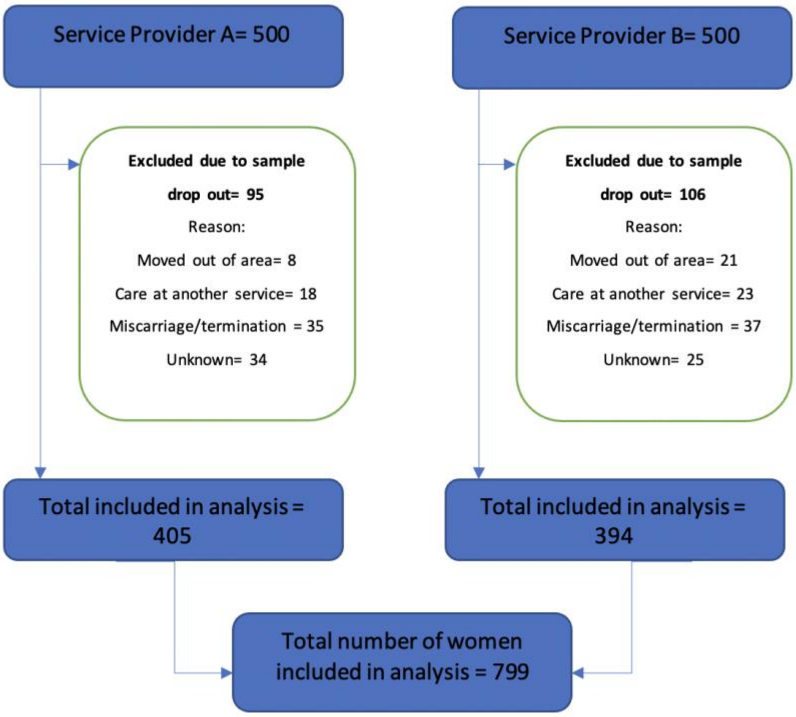

Figure 2: Quantitative data collection flowchart

Appendix 1: Definitions

Table 1: Definitions of the different models of care received by women at the two service providers evaluated

**Service A**	**Standard Care:** Depending on medical risk factors women receive antenatal and postnatal care as set by NICE guidance [2] in either the community setting or hospital setting. For low risk women care is usually provided by a community midwife in a GP surgery or local children's centre. For women at high medical risk care is provided at the hospital and shared between midwives and obstetricians. Although women may be assigned a 'named' healthcare professional, there is no emphasis on the provision of continuity of care.<br><br>**Group Practice:** Women are seen in either the community or hospital setting depending on their medical risk status. There is an aim to provide antenatal and postnatal continuity of care. Women have a named midwife who aims to see them for most of their antenatal and postnatal appointments. Intrapartum care is covered by the hospitals labour ward or birth centre staff. Women planning a homebirth will be looked after by on-call midwives, but this may be from a team not known to the woman.<br><br>**Specialist model: - Community-based Model [CBM]**<br>A team of 6 midwives provide continuity of care to women located in an area of social deprivation. Not all women under their care will have social risk factors. Each woman is assigned a named midwife who coordinates all care, multi-disciplinary communication, and referrals. The named midwife aims to provide most of the clinical care, with others in the team providing care when s/he is not on duty. The midwives are based in a local community health centre and offer antenatal, intrapartum, and postnatal care in the home, community, or hospital setting.
**Service B**	**Standard Care:** Depending on medical risk factors women receive antenatal and postnatal care as set by NICE guidance [2]. If women do not live within the geographical catchment areas of the group practices (see definition below), care is usually provided at the hospital and shared between midwives and obstetricians. Although women may be assigned a 'named' healthcare professional, there is no emphasis on the provision of continuity of care.<br><br>**Group practice** Women are seen in different settings depending on their medical risk status but there is an aim to provide antenatal and postnatal continuity of care. For most women who live within the hospital's geographical catchment area, care is provided in the community setting, often out of children's centres to prevent women from having to travel to the hospital for appointments. Postnatal care is provided at home and in postnatal clinics in the community. Women have a named midwife who aims to see them for most of their antenatal and postnatal appointments. Intrapartum care is covered by the hospitals labour ward or birth centre staff. Women planning a homebirth are looked after by a team of midwives providing on call care, with the aim for the midwives to have met the woman before.<br><br>**Specialist model: - Hospital-based Model [HBM]**<br>A team of 6 midwives provide continuity of care to women with social risk factors only. Women with one or more significant social risk factors (see Appendix A for inclusion criteria) are referred to the team and assigned a named midwife who coordinates all care, multi-disciplinary communication, and referrals. The named midwife aims to provide most of the clinical care, with others in the team providing care when she/he is not on duty. The midwives are based at the hospital site and offer antenatal, intrapartum, and postnatal care in the home or hospital setting.

Table 2: General definitions

Term	Definition
Deprivation score	A composite measure using routine data from the seven domains of deprivation to identify the most disadvantaged areas in England, UK [2]
Ethnicity	Using the ONS 18+ categories of ethnicity
Medical risk status	As recorded by healthcare professional entering routinely collected maternity record data. High medical risk status leads to obstetric-led care although many women will continue to see both obstetricians and midwives during their pregnancy. Low medical risk status leads to midwife-led care throughout pregnancy, birth and the postnatal period if the pregnancy remains low risk/uncomplicated.
Continuity of Care	The midwife is the lead professional in the planning, organisation and delivery of care given to a woman from initial booking to the postnatal period
Social Risk Factors	As recorded by healthcare professional entering routinely collected maternity record data* and includes:<br>Domestic abuse- an incident or pattern of incidents of controlling, coercive, threatening, degrading and violent behaviour, including sexual violence, in the majority of cases by a partner or ex-partner, but also by a family member or carer.<br>Common mental health- Includes depression, anxiety, post-traumatic stress disorder, and obsessive-compulsive disorder.<br>Severe mental health-Includes psychological problems that are so debilitating that they effect peoples ability to engage in functional and occupational activities. Includes schizophrenia, bipolar disorder and psychosis<br>Non-English speaking<br>Social care involvement- previous or current involvement with social care<br>Drug/alcohol abuse<br>Unsupported/single<br>Financial/housing<br>Learning disability<br>Sexual abuse/trafficked<br>AS/Refugee<br>FGM- Female genital mutilation<br>No recourse to public funds<br>*These risk factors could be ascertained through self-reporting
Demographics	**Level of education (completed)**<br>Secondary school only<br>Completed college<br>Completed university<br>**Occupation Status (NS- SEC)**<br>8 (long term unemployed)

	7 (routine occupations)<br>6-3 (semi-routine)
	Recorded as an episode of care each time a woman has a face-to-face appointment with an obstetrician or midwife during their pregnancy
**Number of antenatal appointments**	
**Number of antenatal appointments with a known professional**	Recorded as an episode of care each time a woman has a face-to-face appointment with an obstetrician or midwife that they have previously met during their pregnancy. The maximum number of appointments with one healthcare professional is reported for the purpose of this paper.
**Looked after in labour by known midwife**	Looked after at some point during intrapartum care by a healthcare professional seen at least once during the antenatal period.
**Number of missed appointments**	Recorded as an appointment that was scheduled but not carried out or attended.

For peer review only

Supplementary file 2: Programme theory refinement and supplementary data (Adjusted and unadjusted data analysis tables and qualitative data)

This file will detail the process of theory refinement. Initial programme theories and CMO configurations constructed by Rayment-Jones et al [1,2] to gain further insight into how and why specialist models of care work, or do not work, for women with low socioeconomic status and social risk factors. Each initial CMO configuration was tested through a three-step process- see Figure 1below for an explanatory diagram of the process:

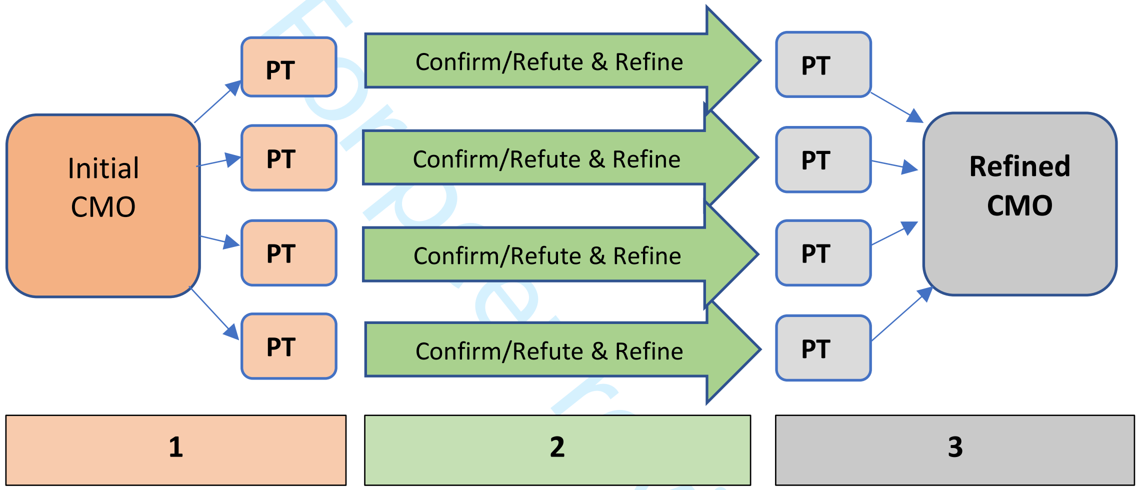

Figure 1: Programme theory refinement CMO configuration development process

This process is described below with the numbers correlating to the order of the diagram:

1) The initial CMO configuration is presented and broken down into smaller testable programme theories. Rival, or conflicting theories, and new theories are presented alongside these programme theories. These rival theories were identified throughout the project including the realist synthesis, focus groups with service providers, patient and public involvement group liaison, supervisory and advisory group discussions.
2) The programme theories are individually tested by drawing on the relevant quantitative data presented in Chapter 7, and qualitative longitudinal interview data to confirm or refute the programme theory before refining them to better reflect the findings.
3) The refined programme theories relating to the initial CMO configuration are compiled in a new, refined CMO configuration that represents the findings of the research.

For peer review only

The above three step process gives way to middle-range theories, indicating how the specialist model of care activates mechanisms amongst whom and in what conditions to bring about different outcomes. The refined CMO configurations summarised in Table 13 of the main manuscript will inform the development of a theoretically informed model of maternity care for women with social risk factors.

Analysis 1: Model of care accessed by deprivation score and risk factors

Initial Programme Theory: If women living in areas of deprivation are prioritised to receive continuity of care through community-based models then services are likely to identify women with social risk factors who have not previously disclosed these issues with professionals, and care is less likely to be disrupted during pregnancy when a disclosure is made. This may also avoid women feeling discriminated against due to specific social risk factors that they may feel do not place them at higher risk or may place them at a higher risk of increased surveillance.

Rival Theory: Placing specialist models of care that include increased levels of continuity in deprived communities does not protect those services from becoming used by more affluent women who are not at such high risk of poor outcomes and experiences and are able to coordinate the system. This would result in those women with low SES and social risk factors having to seek care elsewhere, perhaps outside of their local communities.

Testing using quantitative data:

Firstly, the quantitative data was used to test the hypothesis that women with low socioeconomic status and social risk factors are most likely to receive specialist models of care. The data presented in Table 1 shows that women receiving specialist models of care were more likely to be in the more deprived deciles even after adjusting for women's characteristics, the service provider attended and the place of antenatal care. These findings suggest that the aims of the specialist models- to reach the most deprived women, are being met. Further analysis shows that the specialist models of care based in areas of deprivation were indeed caring for more women with low SES and social risk factors, therefore refuting the rival theory. However, Table 2 also highlighted a significant relationship between the place of antenatal care and the model of care received, with women in the highest deprivation deciles attending hospital based antenatal care being less likely to be cared for by the specialist model. This supports the initial programme theory that community-based models are more likely to identify women with low SES who are more likely to be experiencing social risk factors.

For peer review only

Table 1: Maternity care received by deprivation score

Multinomial logistic regression — Number of obs = 765; LR chi2(57) = 153.19; Prob > chi2 = 0.0000; Log likelihood = -948.68392; Pseudo R2 = 0.0747

imd_3_score	RRR	Std. Err.	z	P>|z|	[95% Conf.	Interval]
**most_deprived**						
mod_care_4						
standard	.291514	.1930548	-1.86	0.063	.0796072	1.067497
partial CoC	.1270207	.0863271	-3.04	0.002	.0335253	.481257
ethnicity						
BA	8.688427	6.039025	3.11	0.002	2.224848	33.92985
BC	4.741514	3.985545	1.85	0.064	.91291	24.6267
BO	3.775191	4.386607	1.14	0.253	.3871507	36.81272
M	1.711281	1.391649	0.66	0.509	.3476215	8.424344
U	2.078345	.9214765	1.65	0.099	.8716061	4.955816
WB	.6403515	.2711355	-1.05	0.292	.279258	1.468356
WO	1.002714	.3973854	0.01	0.995	.4611452	2.180302
WP	2.391133	13830.83	0.00	1.000	0	.
age_cat						
20-24	1.37e-06	.0012548	-0.01	0.988	0	.
25-29	1.19e-06	.0010827	-0.01	0.988	0	.
30-34	4.47e-07	.0004081	-0.02	0.987	0	.
greater than 34	2.79e-07	.0002551	-0.02	0.987	0	.
0.parity	.6508242	.1653202	-1.69	0.091	.395589	1.070738
1.any_risk	1.684199	.5775913	1.52	0.129	.8599561	3.298455
2.high_risk_num	2.372965	.9909666	2.07	0.039	1.046704	5.379712
place_hosp_comm						
hospital	.4068348	.1188187	-3.08	0.002	.2295193	.7211356
imp_gstt						
imp	2.764661	.8701099	3.23	0.001	1.491925	5.123148
_cons	1.15e+07	1.05e+10	0.02	0.986	0	.
**3rd_and_4th_deciles**						
mod_care_4						
standard	.2897059	.1877638	-1.91	0.056	.081335	1.031898
partial CoC	.1905813	.1257227	-2.51	0.012	.052307	.6943858
ethnicity						
BA	5.239074	3.616894	2.40	0.016	1.353978	20.27205
BC	2.414152	2.031332	1.05	0.295	.4640247	12.55996
BO	7.223335	7.936432	1.80	0.072	.8385027	62.22588
M	1.297728	1.029289	0.33	0.742	.2741926	6.142027
U	2.248795	.9372376	1.94	0.052	.9935552	5.089884
WB	.689703	.2667646	-0.96	0.337	.3231713	1.471945
WO	1.130883	.4187918	0.33	0.740	.5472657	2.336883
WP	3.09e+07	1.41e+11	0.00	0.997	0	.
age_cat						
20-24	1.58e-06	.001445	-0.01	0.988	0	.
25-29	9.24e-07	.0008435	-0.02	0.988	0	.
30-34	5.30e-07	.0004843	-0.02	0.987	0	.
greater than 34	2.87e-07	.0002617	-0.02	0.987	0	.
0.parity	.66367	.1575855	-1.73	0.084	.4167141	1.056979
1.any_risk	1.480933	.4932381	1.18	0.238	.7709656	2.844695
2.high_risk_num	2.558484	.9732096	2.47	0.014	1.213942	5.392215
place_hosp_comm						
hospital	.4245959	.1120848	-3.25	0.001	.2530904	.7123212
imp_gstt						
imp	1.547321	.4430837	1.52	0.127	.8827429	2.71223
_cons	1.86e+07	1.70e+10	0.02	0.985	0	.
**5th_and_6th_deciles**						
mod_care_4						
standard	.2513724	.1692147	-2.05	0.040	.0671924	.9404055
partial CoC	.1490418	.1026833	-2.76	0.006	.0386249	.575107
ethnicity						
BA	5.558974	4.138049	2.30	0.021	1.292314	23.9123
BC	4.761389	4.20312	1.77	0.077	.843985	26.86164
BO	2.572342	3.305336	0.74	0.462	.2072886	31.92139
M	.9059086	.9060892	-0.10	0.921	.1275596	6.433623
U	2.623069	1.22996	2.06	0.040	1.046359	6.575653
WB	1.151676	.5021643	0.32	0.746	.4899841	2.706942
WO	1.536157	.6463339	1.02	0.308	.6734338	3.504099
WP	1.570376	9622.35	0.00	1.000	0	.
age_cat						
20-24	1.05e-06	.0009586	-0.02	0.988	0	.
25-29	1.12e-06	.0010216	-0.02	0.988	0	.
30-34	6.55e-07	.0005984	-0.02	0.988	0	.
greater than 34	4.74e-07	.0004326	-0.02	0.987	0	.
0.parity	1.061601	.2752384	0.23	0.818	.6386644	1.764615
1.any_risk	.9115363	.3385852	-0.25	0.803	.4401491	1.887766
2.high_risk_num	1.397186	.6138501	0.76	0.446	.5905828	3.305426
place_hosp_comm						
hospital	.6201716	.1818529	-1.63	0.103	.3490713	1.101817
imp_gstt						
imp	2.111408	.6563113	2.40	0.016	1.148112	3.882935
_cons	4769809	4.36e+09	0.02	0.987	0	.
**least_deprived**	(base outcome)					

Table 2: Place of antenatal care recorded by deprivation score

Multinomial logistic regression

Number of obs	=	765	
LR chi2(57)	=	153.19	
Prob > chi2	=	0.0000	
Log likelihood = -948.68392	Pseudo R2	=	0.0747

imd_3_score	RRR	Std. Err.	z	P>|z|	[95% Conf.	Interval]
**most_deprived**						
place_hosp_comm						
hospital	.4068348	.1188187	-3.08	0.002	.2295193	.7211356
ethnicity						
BA	8.688427	6.039025	3.11	0.002	2.224848	33.92985
BC	4.741514	3.985545	1.85	0.064	.91291	24.6267
BO	3.775191	4.386607	1.14	0.253	.3871507	36.81272
M	1.711281	1.391649	0.66	0.509	.3476215	8.424344
U	2.078345	.9214765	1.65	0.099	.8716061	4.955816
WB	.6403515	.2711355	-1.05	0.292	.279258	1.468356
WO	1.002714	.3973854	0.01	0.995	.4611452	2.180302
WP	2.391133	13830.83	0.00	1.000	0	.
age_cat						
20-24	1.37e-06	.0012548	-0.01	0.988	0	.
25-29	1.19e-06	.0010827	-0.01	0.988	0	.
30-34	4.47e-07	.0004081	-0.02	0.987	0	.
greater than 34	2.79e-07	.0002551	-0.02	0.987	0	.
0.parity	.6508242	.1653202	-1.69	0.091	.395589	1.070738
1.any_risk	1.684199	.5775913	1.52	0.129	.8599561	3.298455
2.high_risk_num	2.372965	.9909666	2.07	0.039	1.046704	5.379712
mod_care_4						
standard	.291514	.1930548	-1.86	0.063	.0796072	1.067497
partial CoC	.1270207	.0863271	-3.04	0.002	.0335253	.481257
imp_gstt						
imp	2.764661	.8701099	3.23	0.001	1.491925	5.123148
_cons	1.15e+07	1.05e+10	0.02	0.986	0	.
**3rd_and_4th_deciles**						
place_hosp_comm						
hospital	.4245959	.1120848	-3.25	0.001	.2530904	.7123212
ethnicity						
BA	5.239074	3.616894	2.40	0.016	1.353978	20.27205
BC	2.414152	2.031332	1.05	0.295	.4640247	12.55996
BO	7.223335	7.936432	1.80	0.072	.8385027	62.22588
M	1.297728	1.029289	0.33	0.742	.2741926	6.142027
U	2.248795	.9372376	1.94	0.052	.9935552	5.089884
WB	.689703	.2667646	-0.96	0.337	.3231713	1.471945
WO	1.130883	.4187918	0.33	0.740	.5472657	2.336883
WP	3.09e+07	1.41e+11	0.00	0.997	0	.
age_cat						
20-24	1.58e-06	.001445	-0.01	0.988	0	.
25-29	9.24e-07	.0008435	-0.02	0.988	0	.
30-34	5.30e-07	.0004843	-0.02	0.987	0	.
greater than 34	2.87e-07	.0002617	-0.02	0.987	0	.
0.parity	.66367	.1575855	-1.73	0.084	.4167141	1.056979
1.any_risk	1.480933	.4932381	1.18	0.238	.7709656	2.844695
2.high_risk_num	2.558484	.9732096	2.47	0.014	1.213942	5.392215
mod_care_4						
standard	.2897059	.1877638	-1.91	0.056	.081335	1.031898
partial CoC	.1905813	.1257227	-2.51	0.012	.052307	.6943858
imp_gstt						
imp	1.547321	.4430837	1.52	0.127	.8827429	2.71223
_cons	1.86e+07	1.70e+10	0.02	0.985	0	.
**5th_and_6th_deciles**						
place_hosp_comm						
hospital	.6201716	.1818529	-1.63	0.103	.3490713	1.101817
ethnicity						
BA	5.558974	4.138049	2.30	0.021	1.292314	23.9123
BC	4.761389	4.20312	1.77	0.077	.843985	26.86164
BO	2.572342	3.305336	0.74	0.462	.2072886	31.92139
M	.9059086	.9060892	-0.10	0.921	.1275596	6.433623
U	2.623069	1.22996	2.06	0.040	1.046359	6.575653
WB	1.151676	.5021643	0.32	0.746	.4899841	2.706942
WO	1.536157	.6463339	1.02	0.308	.6734338	3.504099
WP	1.570376	9622.35	0.00	1.000	0	.
age_cat						
20-24	1.05e-06	.0009586	-0.02	0.988	0	.
25-29	1.12e-06	.0010216	-0.02	0.988	0	.
30-34	6.55e-07	.0005984	-0.02	0.988	0	.
greater than 34	4.74e-07	.0004326	-0.02	0.987	0	.
0.parity	1.061601	.2752384	0.23	0.818	.6386644	1.764615
1.any_risk	.9115363	.3385852	-0.25	0.803	.4401491	1.887766
2.high_risk_num	1.397186	.6138501	0.76	0.446	.5905828	3.305426
mod_care_4						
standard	.2513724	.1692147	-2.05	0.040	.0671924	.9404055
partial CoC	.1490418	.1026833	-2.76	0.006	.0386249	.575107
imp_gstt						
imp	2.111408	.6563113	2.40	0.016	1.148112	3.882935
_cons	4769809	4.36e+09	0.02	0.987	0	.
**least_deprived**	(base outcome)					

The qualitative data was then used to explore women's experiences of being referred to continuity models of care, focusing on the 'how' and 'why' women with low SES were more likely to receive specialist models

at the two services evaluated, and why those with low SES were less likely to receive antenatal care in the hospital setting, despite model of care received.

Testing using qualitative data: Women reported different pathways into the specialist models including direct referrals from their GP, midwives working in other models, sexual health clinics, social workers, accident and emergency departments, and self-referral. This indicates the specialist models are known to local services and have open referral pathways. However, the qualitative findings suggest that most women in the hospital based model were not aware the model existed. When women were referred to the model of care by other healthcare professionals, they were often not aware why they had been referred, or what the aim of the model of care was.

Table 3: Qualitative quotations relating to women's experiences of referral to the specialist model of care

'I was just sent here. I think it's because of, I have specific needs, but I think he [GP] must have known what he was doing because…this is more like a special care for me rather than just normal midwife care, so I assume he did that knowingly…I do not recall a question, I mean like him asking me if this is what I wanted or not wanted.' (CBM1)
'you always have your own midwife. No I don't think I had any choice, it's just based on your location, you will have your GP, and based on your GP you will have, er, nearby hospital based on your location…I don't think there's any choice out there, it's not given.' (HBM3)
*' I think is because of my vulnerability, the mental health issues that I have…. I was actually quite taken aback that, um, because I didn't have to do anything, I didn't have to chase anyone I didn't have to fight with anyone, it [*the referral*] was just done…so obviously my doctor had done that. There must have been some sort of … I don't know, either procedure on the system or they liaise with each other. But, I thought, thank God, because I felt like it was a moment of euphoria where I myself didn't have to fight. Didn't have to run around. It was just done. (HBM8)*
'I mean the fact that I'm seeing, um, you know [HBM], I don't know if you want to class that as discrimination [laughs], because [midwife name]'s deals with so-called 'vulnerable women'. So you can look at that two ways. I was just referred. I received a letter, um, saying you know, 'You'll be seeing, um, er, a member of the [HBM],' it wasn't kind of like disclosed on the paper you know. It's just something that I kind of gathered myself.' (HBM9)

For peer review only

The qualitative and quantitative data appear to come to the same conclusion that community-based models of care in areas of deprivation are likely to identify women who are experiencing social risk factors that increase their chances of poor birth outcomes. The rival theory is therefore refuted in the context of this evaluation. Although the hospital-based model cared for women with at least once social risk factor, the data suggests that only those women who have a known social risk factor are cared for by the team, and these women are not necessarily in the highest deprivation centile. This may mean that the service is not identifying women who are at increased risk but are yet to disclose sensitive, often difficult social risk factors.

For peer review only

Analysis 2: Timing of access to maternity services

Initial programme theory: If maternity care incorporated early pregnancy care (from conception/confirmation of pregnancy), then women would not view it as a package of care for viable and continuing pregnancies and therefore see value of accessing care early in pregnancy to seek support and advice regardless of whether or not they intend to continue the pregnancy

Rival PT: If maternity care incorporated early pregnancy care (from conception/confirmation of pregnancy), then women might perceive this as added surveillance and increased importance placed on the wellbeing of the fetus, this may result in less women accessing appropriately timed abortion services.

Testing using quantitative data: Table 4 and 5 show that the majority of women booked with maternity services later than 10 weeks' gestation, the recommended time at which to book, particularly those with social and medical risk factors. No significant relationship was found between the model of care and the gestation at which women attended the booking appointment. Women attending their booking appointment after 12 weeks were significantly more likely to be receiving hospital based care.

This data contributes to the testing of the theory by revealing the context that women with social and medical risk factors are not accessing maternity services early in pregnancy. The qualitative data will go on to explore if this is due to their preferences and behaviours or system barriers.

Table 4: Gestation at booking appointment in relation to the model of care received

For peer review only

Multinomial logistic regression		Number of obs	=	764
		LR chi2(66)	=	118.84
		Prob > chi2	=	0.0001
Log likelihood = -867.49667		Pseudo R2	=	0.0641

booking_by_weeks	RRR	Std. Err.	z	P>|z|	[95% Conf.	Interval]
**less_than_10**	(base outcome)					
**10_to_13**						
mod_care_4						
standard	.7668591	.2321091	-0.88	0.380	.4237193	1.387883
partial CoC	.8674172	.2802026	-0.44	0.660	.460536	1.633776
ethnicity						
BA	1.334442	.5151908	0.75	0.455	.6261447	2.843969
BC	.766018	.3445405	-0.59	0.553	.3172375	1.849667
BO	.6923595	.3854517	-0.66	0.509	.232514	2.061646
M	.3655942	.2340446	-1.57	0.116	.1042511	1.282088
U	1.279369	.399387	0.79	0.430	.6938601	2.358955
WB	.9397428	.2956228	-0.20	0.843	.5072702	1.740919
WO	.8957496	.2647394	-0.37	0.710	.5018957	1.598674
WP	5067080	1.20e+10	0.01	0.995	0	.
age_cat						
20-24	1.538972	1.460734	0.45	0.650	.239498	9.889157
25-29	1.753686	1.60286	0.61	0.539	.2923871	10.5183
30-34	1.280188	1.154429	0.27	0.784	.21862	7.496481
greater than 34	1.567427	1.417993	0.50	0.619	.2661565	9.230766
0.parity	.8088913	.1438613	-1.19	0.233	.5708264	1.146242
imd_3_score						
most deprived	.9860824	.2740039	-0.05	0.960	.5719905	1.699956
3rd and 4th deciles	.776939	.2004962	-0.98	0.328	.4685184	1.28839
5th and 6th deciles	.896944	.2518034	-0.39	0.698	.5173727	1.554989
1.any_risk	.7892282	.1783753	-1.05	0.295	.5067813	1.229093
2.high_risk_num	.7087357	.1948708	-1.25	0.211	.4134678	1.214862
place_hosp_comm						
hospital	1.00336	.2024533	0.02	0.987	.6756249	1.490076
imp_gstt						
imp	1.94413	.428977	3.01	0.003	1.261553	2.996022
_cons	.751263	.7661935	-0.28	0.779	.1017827	5.54511
**13_to_20**						
mod_care_4						
standard	.891767	.3978579	-0.26	0.797	.371956	2.138017
partial CoC	.7594591	.3653578	-0.57	0.567	.2958091	1.949832
ethnicity						
BA	1.880764	1.007965	1.18	0.239	.6578797	5.376777
BC	.879995	.6116616	-0.18	0.854	.2253368	3.436594
BO	.6772392	.5960294	-0.44	0.658	.1206731	3.800787
M	.5571623	.5041002	-0.65	0.518	.09459	3.281845
U	1.528332	.7273685	0.89	0.373	.601328	3.884402
WB	1.035115	.5188021	0.07	0.945	.3875826	2.764476
WO	.9787965	.4439433	-0.05	0.962	.4023659	2.381024
WP	.6953945	3549.102	-0.00	1.000	0	.
age_cat						
20-24	.3697443	.350147	-1.05	0.293	.0577851	2.365851
25-29	.3577783	.3203188	-1.15	0.251	.0618776	2.068686
30-34	.1970194	.1730667	-1.85	0.064	.0352202	1.102111
greater than 34	.4102757	.3572851	-1.02	0.306	.0744414	2.261192
0.parity	.7365051	.1987517	-1.13	0.257	.4339826	1.249911
imd_3_score						
most deprived	1.869325	.8781225	1.33	0.183	.7444399	4.693966
3rd and 4th deciles	1.450336	.6606416	0.82	0.414	.5939339	3.541595
5th and 6th deciles	1.924385	.9115327	1.38	0.167	.7605	4.869503
1.any_risk	1.406591	.4298377	1.12	0.264	.7727702	2.560265
2.high_risk_num	.5371098	.2690423	-1.24	0.215	.2012287	1.433628
place_hosp_comm						
hospital	1.05097	.3495657	0.15	0.881	.5476087	2.017022
imp_gstt						
imp	2.732355	.9649809	2.85	0.004	1.367477	5.45952
_cons	.3516654	.4063003	-0.90	0.366	.036533	3.385121
**3**						
mod_care_4						
standard	1.276589	.6689131	0.47	0.641	.4571242	3.565067
partial CoC	1.154034	.644423	0.26	0.798	.3862778	3.447762
ethnicity						
BA	2.23247	1.209553	1.48	0.138	.7719772	6.45605
BC	.3917793	.3365533	-1.09	0.275	.072749	2.109872
BO	6.47e-07	.0004433	-0.02	0.983	0	.
M	.3680627	.4184516	-0.88	0.379	.039645	3.417083
U	1.183814	.5736468	0.35	0.728	.4579438	3.060233
WB	.7463861	.37587	-0.58	0.561	.2781697	2.002706
WO	.8127787	.3744247	-0.45	0.653	.3294938	2.004922
WP	.7171062	3822.002	-0.00	1.000	0	.
age_cat						
20-24	2.274215	2.940942	0.64	0.525	.1803371	28.67993
25-29	1.210863	1.550069	0.15	0.881	.0985013	14.88499
30-34	1.303381	1.621172	0.21	0.831	.1138492	14.92152
greater than 34	1.273376	1.59059	0.19	0.847	.1100805	14.73002
0.parity	1.794035	.509178	2.06	0.039	1.028597	3.129079
imd_3_score						
most deprived	.9455481	.400684	-0.13	0.895	.4120783	2.169639
3rd and 4th deciles	.7027879	.2798035	-0.89	0.376	.3220567	1.533615
5th and 6th deciles	.7430273	.3252906	-0.68	0.497	.3150333	1.75248
1.any_risk	1.938461	.6191795	2.07	0.038	1.036497	3.625319
2.high_risk_num	2.498193	.9487996	2.41	0.016	1.18671	5.259053
place_hosp_comm						
hospital	2.510521	.8052141	2.87	0.004	1.338915	4.707331
imp_gstt						
imp	1.328284	.4706625	0.80	0.423	.6632503	2.660141
_cons	.0502035	.0742011	-2.02	0.043	.0027711	.9095269

Table 5: Gestation at booking appointment in relation to the place of antenatal care

Multinomial logistic regression

Number of obs	=	764	
LR chi2(66)	=	118.84	
Prob > chi2	=	0.0001	
Pseudo R2	=	0.0641	

Log likelihood = -867.49667

booking_by_weeks	RRR	Std. Err.	z	P>|z|	[95% Conf.	Interval]
**less_than_10**	(base outcome)					
**10_to_13**						
place_hosp_comm						
hospital	1.00336	.2024533	0.02	0.987	.6756249	1.490076
ethnicity						
BA	1.334442	.5151908	0.75	0.455	.6261447	2.843969
BC	.766018	.3445405	-0.59	0.553	.3172375	1.849667
BO	.6923595	.3854517	-0.66	0.509	.232514	2.061646
M	.3655942	.2340446	-1.57	0.116	.1042511	1.282088
U	1.279369	.399387	0.79	0.430	.6938601	2.358955
WB	.9397428	.2956228	-0.20	0.843	.5072702	1.740919
WO	.8957496	.2647394	-0.37	0.710	.5018957	1.598674
WP	5067080	1.20e+10	0.01	0.995	0	.
age_cat						
20-24	1.538972	1.460734	0.45	0.650	.239498	9.889157
25-29	1.753686	1.60286	0.61	0.539	.2923871	10.5183
30-34	1.280188	1.154429	0.27	0.784	.21862	7.496481
greater than 34	1.567427	1.417993	0.50	0.619	.2661565	9.230766
1.parity	1.23626	.2198688	1.19	0.233	.8724162	1.751846
imd_3_score						
3rd and 4th deciles	.7879048	.1724215	-1.09	0.276	.5130983	1.209893
5th and 6th deciles	.9096035	.2323215	-0.37	0.711	.5513749	1.500573
least deprived	1.014114	.2817931	0.05	0.960	.5882506	1.748281
1.any_risk	.7892282	.1783753	-1.05	0.295	.5067813	1.229093
2.high_risk_num	.7087357	.1948708	-1.25	0.211	.4134678	1.214862
mod_care_4						
standard	.7668591	.2321091	-0.88	0.380	.4237193	1.387883
partial CoC	.8674172	.2802026	-0.44	0.660	.460536	1.633776
imp_gstt						
imp	1.94413	.428977	3.01	0.003	1.261553	2.996022
_cons	.5992325	.5848482	-0.52	0.600	.0884762	4.058488
**13_to_20**						
place_hosp_comm						
hospital	1.05097	.3495657	0.15	0.881	.5476087	2.017022
ethnicity						
BA	1.880764	1.007965	1.18	0.239	.6578797	5.376777
BC	.879995	.6116616	-0.18	0.854	.2253368	3.436594
BO	.6772392	.5960294	-0.44	0.658	.1206731	3.800787
M	.5571623	.5041002	-0.65	0.518	.09459	3.281845
U	1.528332	.7273685	0.89	0.373	.601328	3.884402
WB	1.035115	.5188021	0.07	0.945	.3875826	2.764476
WO	.9787965	.4439433	-0.05	0.962	.4023659	2.381024
WP	.6953945	3549.102	-0.00	1.000	0	.
age_cat						
20-24	.3697443	.350147	-1.05	0.293	.0577851	2.365851
25-29	.3577783	.3203188	-1.15	0.251	.0618776	2.068686
30-34	.1970194	.1730667	-1.85	0.064	.0352202	1.102111
greater than 34	.4102757	.3572851	-1.02	0.306	.0744414	2.261192
1.parity	1.357764	.3664033	1.13	0.257	.8000567	2.30424
imd_3_score						
3rd and 4th deciles	.7758606	.2442081	-0.81	0.420	.41866	1.437825
5th and 6th deciles	1.029455	.3671188	0.08	0.935	.5117479	2.070896
least deprived	.5349525	.251296	-1.33	0.183	.2130395	1.343292
1.any_risk	1.406591	.4298377	1.12	0.264	.7727702	2.560265
2.high_risk_num	.5371098	.2690423	-1.24	0.215	.2012287	1.433628
mod_care_4						
standard	.891767	.3978579	-0.26	0.797	.371956	2.138017
partial CoC	.7594591	.3653578	-0.57	0.567	.2958091	1.949832
imp_gstt						
imp	2.732355	.9649809	2.85	0.004	1.367477	5.45952
_cons	.4841614	.5020639	-0.70	0.484	.0634329	3.695438
**3**						
place_hosp_comm						
hospital	2.510521	.8052141	2.87	0.004	1.338915	4.707331
ethnicity						
BA	2.23247	1.209553	1.48	0.138	.7719772	6.45605
BC	.3917793	.3365533	-1.09	0.275	.072749	2.109872
BO	6.47e-07	.0004433	-0.02	0.983	0	.
M	.3680627	.4184516	-0.88	0.379	.039645	3.417083
U	1.183814	.5736468	0.35	0.728	.4579438	3.060233
WB	.7463861	.37587	-0.58	0.561	.2781697	2.002706
WO	.8127787	.3744247	-0.45	0.653	.3294938	2.004922
WP	.7171062	3822.002	-0.00	1.000	0	.
age_cat						
20-24	2.274215	2.940942	0.64	0.525	.1803371	28.67993
25-29	1.210863	1.550069	0.15	0.881	.0985013	14.88499
30-34	1.303381	1.621172	0.21	0.831	.1138492	14.92152
greater than 34	1.273376	1.59059	0.19	0.847	.1100805	14.73002
1.parity	.5574027	.1582005	-2.06	0.039	.3195828	.9721981
imd_3_score						
3rd and 4th deciles	.7432598	.2534893	-0.87	0.384	.3809235	1.450252
5th and 6th deciles	.7858165	.3181927	-0.60	0.552	.3553471	1.737759
least deprived	1.057588	.4481618	0.13	0.895	.4609062	2.426723
1.any_risk	1.938461	.6191795	2.07	0.038	1.036497	3.625319
2.high_risk_num	2.498193	.9487996	2.41	0.016	1.18671	5.259053
mod_care_4						
standard	1.276589	.6689131	0.47	0.641	.4571242	3.565067
partial CoC	1.154034	.644423	0.26	0.798	.3862778	3.447762
imp_gstt						
imp	1.328284	.4706625	0.80	0.423	.6632503	2.660141
_cons	.0851625	.1209387	-1.73	0.083	.0052658	1.377316

Testing using qualitative data: When interviewing women about when they accessed maternity care many spoke about wanting to be seen earlier in pregnancy, and the impact that late booking, particularly after 12 weeks, had on their emotional wellbeing- see Table 6.

Table 6: Qualitative quotations relating to women's experiences of accessing maternity care

'the main take-away that I have from the whole experience is…the first ten weeks. That you don't have any support from anyone until you have your booking appointment. Like even information on who to call, someone to talk to' (CBM1)
Husband- *In the beginning she was unhappy because she was telling me like, no one is caring about her…first three months.* Woman - *Yeah, there is no one.* Husband- *there is no services, nothing. No one called her. She didn't do a scan…Before 20 weeks, it's different….you feel like they're not ready for someone who is pregnant before 20 weeks. (CBM6)*
'they [maternity services] weren't really too bothered, it was like, 'well, it's not really important till the 12 weeks and that's when we can start doing what we need to do'.'(HBM9)
'Not easy. You near enough have to be dying to get a [GP] appointment. It is ridiculous to get an appointment there. Sometimes it's just not even worth trying to ring up to make an appointment … it's very hard work. [HBM9)
I couldn't get a GP appointment because I'm still registered at my old GP, I went to my local walk-in centre and they couldn't see me because I was pregnant… So I saw a couple of people at the, is it the maternity urgent care centre there? (CBM9)
'And then they [GP based at community health centre] send me to the [CBM] midwife, they send me a letter to contact the midwife…Very easy.' (CMB3)
'I went to the GP. Um, it was horrible…they gave me a very long [late] appointment like up to one month, so all these things were not done, like I wouldn't have done my scan or anything. Yeah, and then I talked to someone, they said about self-referral. And then I self-referred myself, and then I saw the midwife at around 14 weeks.' (HBM5)

For peer review only

This woman, who had no recourse to public funds, described her experience of accessing standard, hospital-based maternity care in a previous pregnancy, and then her experience of accessing the hospital-based specialist model in her current pregnancy.

Previous pregnancy under standard hospital care: '*We went to the GP when I was about six weeks pregnant, and then we couldn't get [maternity] appointment until I was about four months. So what I did was we went to private hospital to do the three-months checks … the blood test and the scan checks. And then the first appointment I had here [at hospital] was when I was four months pregnant with a scan…I don't know maybe within GP to the hospital or the process maybe took long, but I remember phoning them and then asking them, 'When can I get an appointment and see a midwife?' and they said, 'No you can't see at the moment…there's no ready documentation and everything's not ready,' so and then we went to see private hospital. (HBM3)*

This pregnancy under specialist model: *I went to GP and they confirmed I was pregnant. And then I received call, or text, I can't remember maybe both, from [HBM midwife], saying that, 'I'm your midwife, can you come and see me on this day?'…Yes it was straightaway, easy process. (HBM3)*

The data confirms the initial programme theory and challenges the notion that women with social risk factors do not prioritise their maternity care. The qualitative data did not support the rival theory.

Refined PT (demonstrating CMO configuration): If maternity care provision commences when a woman accesses services (M) regardless of her gestation (C) , even if this is in the form of a phone number for advice (M), then women would not feel unsupported (O) , anxious (O), and that the service does not value them until they have a viable pregnancy (O). This might also improve early access to safe abortion and family planning services (O).

For peer review only

Analysis 3: Engagement - Number of antenatal appointments attended

Initial programme theory 1): If women can access a known midwife 24/7 via a phone call or text message, then they will be better able to engage with services, care will be more personalised, they will feel more cared for, and are less likely to have to repeat their history and experience a variation of responses/advice. Additionally, if those women who have few resources, such as no phone credit, have direct, easy access to a midwife through a free phone number, or free technology such as WhatsApp, skype, etc, then their anxieties will be allayed and engagement with services improved.

Rival theory 1): If women can access a known midwife throughout their pregnancy they might become overly reliant on the midwife and misuse the service, leading to inappropriate women-midwife relationships and overburdening the midwife.

Rival theory 2): If women feel they can contact a known midwife for every concern or question they have, then they may become more anxious and less able to seek out information of their own accord, leading to disempowerment and an inability to seek readily available information provided by reliable agencies when they are no longer cared for by their known midwife. Care could also become overmedicalized if midwives feel they have to investigate every concern that women might have prioritised less if they were under standard care and only had access to information through an unknown healthcare professional.

Testing using quantitative data: Before looking to the specific programme theories related to continuity of care, relationships and trust, it is important to test the hypothesis that the specialist models of care improve women's engagement with maternity care, and if so, to what extent the level of continuity and other factors such as the place of antenatal care have an impact. It is important to remember that increased engagement is not nesesarily a 'better' outcome, for example more antenatal appointments may be too many and burdensome for the woman, or lead to overmedicalisation or inappropriate intervention. The desired outcome here is a level of engagement that is approporiate to the womans needs.

Engagement with services is tested in tables 7 and 8 through the number of antenatal appointments women attended. Table 8 showed that women accessing the specialist model of care attended a similar number of appointments to those women in the standard model of care and group practices. Women with high medical risk and any social risk factor were more likely to have more than 15 antenatal

For peer review only

appointments. Given that the specialist models provide care for significantly more women with social risk factors, the similar findings across model of care groups suggests that specialist model might be mitigating the affects of inequality through improved engagement with maternity care for women with low socioeconomic status and social risk factors has been levelled by the specialist model of care. Women at Service A were less likely to have more than 15 antenatal appointments. A possible explanation for this could be due to the disproportionately high numbers of women at Service B having high medical risk status at the onset of labour and therefore requiring more appointments during pregnancy. This theory will be further explored in the qualitative data and discussion section.

When analysing the effect of place of antenatal care, Table 8 showed that women receiving hospital-based antenatal care were significantly less likely to attend the recommended number of appointments compared to those attending community based care, despite the model of care received.

Table 7: Number of antenatal appointments attended in relation to the model of care accessed

For peer review only

1_6	(base outcome)					
7_9						
mod_care_4						
standard	1.149272	.39513	0.40	0.686	.5858305	2.254621
partial CoC	1.812476	.6855162	1.57	0.116	.863635	3.803772
ethnicity						
BA	.5885425	.2427223	-1.29	0.199	.2622598	1.32076
BC	1.099803	.5676191	0.18	0.854	.3999463	3.024324
BO	4.060241	4.736953	1.20	0.230	.4125555	39.95962
M	1.171773	.7740172	0.24	0.810	.3210558	4.276678
U	.759139	.2641498	-0.79	0.428	.3838287	1.50143
WB	.5519485	.1999764	-1.64	0.101	.2713313	1.122787
WO	.7548208	.2487774	-0.85	0.393	.3956406	1.440081
WP	2.74e+10	5.45e+15	0.00	1.000	0	.
age_cat						
20-24	1.483199	1.397323	0.42	0.676	.2340356	9.399771
25-29	1.624311	1.465304	0.54	0.591	.2772002	9.517979
30-34	1.808432	1.601011	0.67	0.503	.3189559	10.25354
greater than 34	1.769831	1.573776	0.64	0.521	.3097586	10.11208
0.parity	.8018499	.1583715	-1.12	0.264	.5444704	1.180897
imd_3_score						
most deprived	.9755324	.2950882	-0.08	0.935	.5392154	1.764904
3rd and 4th deciles	.900557	.2580345	-0.37	0.715	.5135923	1.579079
5th and 6th deciles	1.030453	.3231764	0.10	0.924	.5572748	1.905402
1.any_risk	.7663696	.1894805	-1.08	0.282	.4720452	1.244208
2.high_risk_num	1.527345	.5028294	1.29	0.198	.8011361	2.911843
place_hosp_comm						
hospital	1.610698	.3915808	1.96	0.050	1.000176	2.593892
imp_gstt						
imp	1.778247	.4527273	2.26	0.024	1.07965	2.928877
_cons	.3449901	.3564553	-1.03	0.303	.045532	2.613946
10_14						
mod_care_4						
standard	.9440856	.3450886	-0.16	0.875	.4611841	1.932629
partial CoC	1.765937	.7013047	1.43	0.152	.8108462	3.846024
ethnicity						
BA	.7315004	.3275001	-0.70	0.485	.3041747	1.759163
BC	.9096089	.5294628	-0.16	0.871	.2906628	2.846557
BO	11.42446	12.8353	2.17	0.030	1.263331	103.3129
M	1.689606	1.169752	0.76	0.449	.43499	6.562836
U	.8829985	.3416791	-0.32	0.748	.4136041	1.885103
WB	.922301	.3626736	-0.21	0.837	.426734	1.993371
WO	.8191682	.3035382	-0.54	0.590	.3962462	1.693484
WP	.6818543	213248.4	-0.00	1.000	0	.
age_cat						
20-24	4.078474	4.889285	1.17	0.241	.3891123	42.74845
25-29	3.71801	4.365364	1.12	0.263	.3723091	37.12935
30-34	3.286795	3.815627	1.02	0.305	.3377661	31.98373
greater than 34	3.223814	3.749286	1.01	0.314	.3299325	31.50031
0.parity	.7469259	.1584339	-1.38	0.169	.492862	1.131957
imd_3_score						
most deprived	1.3555	.4647616	0.89	0.375	.6922249	2.654311
3rd and 4th deciles	1.424572	.4613422	1.09	0.275	.7551377	2.687465
5th and 6th deciles	1.681039	.5879565	1.49	0.138	.8469609	3.336508
1.any_risk	.9725235	.2515335	-0.11	0.914	.5857946	1.614562
2.high_risk_num	2.733911	.8926909	3.08	0.002	1.441603	5.184694
place_hosp_comm						
hospital	4.356843	1.119242	5.73	0.000	2.633324	7.208409
imp_gstt						
imp	1.158933	.3096098	0.55	0.581	.6865297	1.956399
_cons	.0613308	.0796822	-2.15	0.032	.004806	.7826666
15						
mod_care_4						
standard	.4947072	.2332588	-1.49	0.136	.1963356	1.246514
partial CoC	2.32004	1.123352	1.74	0.082	.8981479	5.992984
ethnicity						
BA	.3915834	.2406746	-1.53	0.127	.1173977	1.306137
BC	1.330121	.8859924	0.43	0.668	.3605009	4.907675
BO	14.03244	16.56205	2.24	0.025	1.388309	141.8339
M	.3621583	.4306236	-0.85	0.393	.0352192	3.724071
U	.4462132	.2238845	-1.61	0.108	.1669005	1.192963
WB	.4952449	.2435218	-1.43	0.153	.1889154	1.298293
WO	.3881658	.1850012	-1.99	0.047	.1525217	.9878774
WP	.6593021	273021.6	-0.00	1.000	0	.
age_cat						
20-24	.4101014	.4703021	-0.78	0.437	.0433249	3.881902
25-29	1.199888	1.214551	0.18	0.857	.1650205	8.724565
30-34	.9813159	.9625507	-0.02	0.985	.1435107	6.710166
greater than 34	1.388501	1.361578	0.33	0.738	.203165	9.489499
0.parity	1.135431	.3389281	0.43	0.670	.6325219	2.038196
imd_3_score						
most deprived	.8194501	.3920441	-0.42	0.677	.3208395	2.092942
3rd and 4th deciles	1.249745	.5321265	0.52	0.601	.5424875	2.879074
5th and 6th deciles	1.721278	.7826422	1.19	0.232	.7060268	4.19644
1.any_risk	1.971849	.670567	2.00	0.046	1.012524	3.840095
2.high_risk_num	4.129237	1.634349	3.58	0.000	1.900924	8.969637
place_hosp_comm						
hospital	7.886996	2.707958	6.01	0.000	4.023983	15.45849
imp_gstt						
imp	.5237601	.1927356	-1.76	0.079	.2546264	1.077361
_cons	.1135639	.1406873	-1.76	0.079	.010017	1.287483

Table 8: Number of antenatal appointments attended in relation to the place of antenatal care

ante_appt_cat	RRR	Std. Err.	z	P>|z|	[95% Conf.	Interval]
**1_6**						
place_hosp_comm						
hospital	.6193839	.1505954	-1.97	0.049	.3845926	.9975136
ethnicity						
BA	1.693631	.6982336	1.28	0.201	.7549079	3.799652
BC	.9099711	.469674	-0.18	0.855	.3308926	2.502466
BO	.2459387	.2869793	-1.20	0.229	.0249794	2.421427
M	.8530948	.5635031	-0.24	0.810	.2337463	3.113507
U	1.317993	.4586435	0.79	0.428	.6663566	2.606871
WB	1.817802	.658774	1.65	0.099	.8934492	3.698479
WO	1.325525	.4369186	0.85	0.393	.6947288	2.529067
WP	5.47e-06	.0028133	-0.02	0.981	0	.
age_cat						
20-24	.6736911	.6347487	-0.42	0.675	.1062827	4.270307
25-29	.6160015	.5557416	-0.54	0.591	.1051111	3.610066
30-34	.553214	.4898026	-0.67	0.504	.0975572	3.13709
greater than 34	.5657324	.5031052	-0.64	0.522	.0990007	3.232836
1.parity	.8019261	.1583226	-1.12	0.264	.5446073	1.180824
imd_3_score						
3rd and 4th deciles	1.0851	.2609095	0.34	0.734	.6773299	1.738357
5th and 6th deciles	.942631	.2663404	-0.21	0.834	.5417946	1.640019
least deprived	.9741613	.2946691	-0.09	0.931	.5384622	1.762408
1.any_risk	1.305822	.3228398	1.08	0.280	.8043415	2.119959
2.high_risk_num	.6512724	.2144145	-1.30	0.193	.3416069	1.241648
imp_gstt						
imp	.5628822	.1432485	-2.26	0.024	.3418175	.9269167
mod_care_4						
standard	.8709317	.2994596	-0.40	0.688	.4439239	1.708676
partial CoC	.551143	.2084863	-1.57	0.115	.2625861	1.156796
_cons	3.705105	3.624279	1.34	0.181	.5447133	25.20189
**7_9**	(base outcome)					
**10_14**						
place_hosp_comm						
hospital	2.706802	.7010945	3.84	0.000	1.629239	4.497053
ethnicity						
BA	1.250261	.5609235	0.50	0.619	.5189353	3.01223
BC	.8264228	.4639844	-0.34	0.734	.2749832	2.483696
BO	2.816899	1.91737	1.52	0.128	.7419706	10.69439
M	1.443782	.9667798	0.55	0.583	.3886181	5.363892
U	1.162341	.4419722	0.40	0.692	.5516585	2.449047
WB	1.664648	.6550648	1.30	0.195	.7697707	3.599842
WO	1.084449	.3876004	0.23	0.821	.5382388	2.184957
WP	3.74e-06	.0023281	-0.02	0.984	0	.
age_cat						
20-24	2.750417	3.611257	0.77	0.441	.2097893	36.059
25-29	2.286663	2.944347	0.64	0.521	.1833074	28.5249
30-34	1.815692	2.31376	0.47	0.640	.1493981	22.0668
greater than 34	1.819661	2.322906	0.47	0.639	.1490666	22.21266
1.parity	1.072536	.2310358	0.33	0.745	.7031594	1.635949
imd_3_score						
3rd and 4th deciles	1.137171	.2975716	0.49	0.623	.6809041	1.899178
5th and 6th deciles	1.179217	.3566926	0.54	0.586	.651809	2.133376
least deprived	.720963	.2481584	-0.95	0.342	.3672196	1.415468
1.any_risk	1.26802	.3372343	0.89	0.372	.7529157	2.135532
2.high_risk_num	1.801903	.6252472	1.70	0.090	.9127898	3.557068
imp_gstt						
imp	.6524645	.1786534	-1.56	0.119	.381493	1.115905
mod_care_4						
standard	.8213091	.3325921	-0.49	0.627	.3713723	1.816368
partial CoC	.9744801	.4157976	-0.06	0.952	.4222568	2.248895
_cons	.2299289	.3119432	-1.08	0.279	.0160981	3.28408
**15**						
place_hosp_comm						
hospital	4.907966	1.683372	4.64	0.000	2.50582	9.612873
ethnicity						
BA	.6804993	.4201494	-0.62	0.533	.2029011	2.282291
BC	1.210251	.7847556	0.29	0.769	.3395752	4.313351
BO	3.471839	2.712416	1.59	0.111	.7508414	16.05354
M	.3102073	.3671998	-0.99	0.323	.0304835	3.156746
U	.5870266	.2926949	-1.07	0.285	.2209244	1.559811
WB	.8915576	.4378813	-0.23	0.815	.3404777	2.334587
WO	.5134041	.2397176	-1.43	0.153	.2055976	1.282037
WP	3.62e-06	.0033946	-0.01	0.989	0	.
age_cat						
20-24	.2764452	.3510455	-1.01	0.311	.0229459	3.330521
25-29	.7364513	.8450313	-0.27	0.790	.0777041	6.979816
30-34	.5412187	.6054519	-0.55	0.583	.0604155	4.848384
greater than 34	.7835655	.876771	-0.22	0.827	.0874225	7.02308
1.parity	.7035639	.211548	-1.17	0.242	.3902687	1.268362
imd_3_score						
3rd and 4th deciles	1.649296	.6300238	1.31	0.190	.7800879	3.487016
5th and 6th deciles	2.010318	.8698524	1.61	0.107	.8609046	4.69434
least deprived	1.195039	.5724279	0.37	0.710	.4673623	3.055699
1.any_risk	2.56915	.8901076	2.72	0.006	1.302813	5.066368
2.high_risk_num	2.738223	1.123561	2.45	0.014	1.22518	6.119809
imp_gstt						
imp	.2950832	.1098794	-3.28	0.001	.1422278	.6122156
mod_care_4						
standard	.4299578	.2149768	-1.69	0.091	.1613724	1.145572
partial CoC	1.278626	.6479404	0.49	0.628	.4735869	3.452134
_cons	.3905271	.5050302	-0.73	0.467	.0309654	4.925227

The qualitative data was interrogated to explore mechanisms that may lead to increased engagement: for example, did women find the number of appointments appropriate for their individual, holistic needs? If they missed an appointment, did they feel they could rebook it without reproach? These were then explored in relation to the level of continuity of care received.

Testing using qualitative data:

Women from both specialist models of care expressed ease of contacting and booking appointments with their midwife or a midwife from the team, particularly when comparing this to their experiences of standard care or other healthcare services. This might be through phone call, text messaging or emailing. This was also valued by women's family members who felt able to contact the specialist model. This women's partner described the benefit of being able to contact a known midwife, and described how this interaction was enhanced by the fact the team communicated with one another and were knowledgeable about their situation. Women who do not speak English particularly valued the ability to text their midwife as it gave them the opportunity to use translation technology. Those who had multiple services involved in their care and needed to attend numerous appointments commented on how the regular care she received from the team made them feel looked after and that they could rely on them to remind her of her appointments:

Table 9: Qualitative quotations relating to women's experiences of engaging with the specialist model of care

'I will book my appointment with midwife through text messages like you're texting a friend, and then it's so informal but also very efficient…. sometimes I can't make the appointment, or my midwife can't make the appointment, then we text each other beforehand' (HBM3) <br>*'the absolute amazing thing about the [specialist model] is that I had a phone number I could call…by the end of the first week pretty much knew all of them on a first name basis. Whoever would answer the phone was reassuring, they were able to talk me through things… if we didn't have the like phone number that we could call the first few weeks it would have been a lot worse, a lot more difficult. We would have ended up in A&E a lot more often than we did… which is zero…it's just like every concern, it's kind of like calling a friend…regardless of which midwife was picking up at the time' (Partner of CBM6)* <br>*'the fact that I see someone regularly. I feel like I'm being looked after as well… I can rely on them to look after me, remind me of appointments and stuff like that as I really struggle … [the midwives] text, call, put it in my notes and*

For peer review only

*what-not so …I am remembering … or I do actually go to these appointments… whereas my other midwife appointments [*under standard care*] were just the normal basic appointments…I was visiting hospital more [because] when I did try to get in contact with someone it was impossible, so I just had to keep running to the hospital.'(CBM1)*

'I'll tell [CBM midwife] a convenient time slot for me. Last week it was at home. Sometimes if my husband is not there, it's better to be at home, because travelling with the kids is not easy.' (CBM2)

'it's been very flexible. Um, they have located a lot of time with my appointments because of my circumstances so … that's really really really helpful particularly with the midwives, it is easy.' (CMB5)

'at one point I was a bit worried, so I emailed them, they called me back, they got me in sort of within the day to do some blood tests' (CBM6)

[concerned she had a urine infection] I called up and, again because I've not had like any really serious problems there's always that hesitation like I don't want to bother anyone, but the team are so lovely they're like, 'You can literally just call us to chat.' and she was like 'Someone is in clinic…you could come in today between these hours and also tomorrow'… I literally got a sample, knocked on the door, gave them that, took a seat for five minutes…and when they had a gap came out and just chatted to me.' (CBM9)

'[an appointment] every two weeks…It's too much. Yeah, and um, after the baby's due date they started every week! It was too much. No medical reason, I think it's a requirement… I prefer, um, when I want to see them, like maybe every three weeks. And then if I need to see them then maybe I can call them and then make appointment before three weeks.' (HBM3)

I'm struggling a little bit. Just I think more, I'm feeling a bit low and irritable. I feel a bit, this week I do feel a bit emotional…it's just me, being on my own. I haven't seen [HBM midwife] in probably about two weeks, um, the last time I had an appointment was with a different midwife because she was on holiday. I was meant to see her today but she, I think she's not feeling very well….how I am feeling now I think more [appointments] would be good. I think [antenatal care] follows a schedule. (HBM2)

For peer review only

Given both the qualitative and quantitative findings above there is no evidence to support the rival theories put forward, therefore these have been refuted. The initial programme theory has been refined to provide more detailed contexts and mechanisms, and how they trigger outcomes.

Refined theory: If women have 24/7 access to a small team of midwives whom they have had the opportunity to meet during pregnancy and are encouraged to contact midwives via a phone call, text message or free technology, then their engagement with services will improve through needs-based communication and appropriately timed antenatal appointments. For women who have multiple social and medical needs, this open access can work both ways through midwives reminding them of appointments, this leads to women feeling more 'cared for'.

For peer review only

Analysis 4: The quality of relational continuity and missed appointments

Initial programme theory: If women feel they have a continued supportive presence throughout pregnancy and the perinatal period, either with a midwife, GP or other healthcare professional, then they will feel better supported and have reduced feelings of anxiety, increased sense of control, and enhanced self-beliefs and wellbeing.

Rival theory: If women have continued supportive presence from a known healthcare professional, then they will become overly reliant on that person and feel anxious when they are not on duty. it is more important that the whole service is perceived as safe, respectful, understanding, and kind, rather than one trusted HCP in a wider toxic environment.

Initial programme theory If women have a level of trust and confidence in their HCP's and do not fear judgement, for example their concerns are listened to on an individual level, they receive meaningful information, and they are able to rebook missed appointments with ease and without reproach, then they will perceive the maternity environment as a place of safety and their engagement with flexible services will improve.

Additional related programme theory identified in focus groups with healthcare professionals: If midwives are able to work flexibly, then they are able to meet women's individual needs and increase safety through spending time care planning and coordinating support that may not be available on demand (for example during an allocated appointment time in the standard maternity care model).

Rival theory: If women feel they can rebook appointments at will then they will not prioritise their maternity care appointments and have less engagement.

Testing using quantitative data: In order to test these programme theories the quality of continuity of care was analysed first through the number of antenatal appointments women received with a known healthcare professional, and whether women were supported in labour by a known healthcare professional. Table 10 shows a significant relationship between the model of care and the number of appointments with a known healthcare professional. The aim of both the group practice and specialist models of care appear to be being met with women more likely to receive more antenatal appointments with a known healthcare professional if they experienced one of these models. Conversely, the fully

For peer review only

adjusted results show that women accessing standard maternity care, Black women, and those in the most deprived deciles were the least likely to see a known healthcare professional for their antenatal appointments. Women receiving care in the specialist model were more likely to be looked after in labour by a known healthcare professional compared to the group practice model. After adjusting for the service provider attended, we found that women in the group practice models were the least likely group to know the person looking after them in labour. This is unsurprising given that these models of care are often set in the community with midwives not working in intrapartum settings. Table 11 shows that women receiving antenatal care based in the hospital were less likely to see a known healthcare professional for their antenatal appointments than those based in the community setting. Importantly, place of antenatal care made no difference the number of women who knew the person looking after them in labour.

No relationship was found between model of care, the place of antenatal care, and the number of missed appointments. Again, this suggests the specialist model of care, that is more likely to care for women with low SES who are known to struggle to enagage, could be mitigating the effects of inequality by reducing the number of appointments women miss

Table 10: Number of appointments and support in labour by known healthcare professional

For peer review only

noofapptswithknown~p	RRR	Std. Err.	z	P>|z|	[95% Conf.	Interval]
0	(base outcome)					
2						
mod_care_4						
standard	2.12497	1.42054	1.13	0.260	.5732255	7.877349
partial CoC	1.814651	1.307945	0.83	0.408	.4418483	7.452689
ethnicity						
BA	.6897641	.3674707	-0.70	0.486	.2427864	1.959642
BC	1.740153	1.212201	0.80	0.426	.4442582	6.816153
BO	1.674624	1.297217	0.67	0.506	.3669016	7.643372
M	3.514697	3.072361	1.44	0.150	.6335969	19.49678
U	1.185132	.5570245	0.36	0.718	.4717297	2.977422
WB	1.151437	.5773619	0.28	0.779	.4309476	3.076492
WO	1.175301	.507662	0.37	0.708	.5040567	2.740431
WP	2.09e+07	1.20e+11	0.00	0.998	0	.
age_cat						
20-24	2087928	2.43e+09	0.01	0.990	0	.
25-29	5006335	5.83e+09	0.01	0.989	0	.
30-34	5239593	6.10e+09	0.01	0.989	0	.
greater than 34	3618132	4.21e+09	0.01	0.990	0	.
0.parity	1.822294	.481216	2.27	0.023	1.086026	3.057712
imd_3_score						
most deprived	.9973948	.4150319	-0.01	0.995	.4412336	2.25458
3rd and 4th deciles	1.119732	.438289	0.29	0.773	.5199173	2.411538
5th and 6th deciles	1.117686	.4667524	0.27	0.790	.4930066	2.533883
1.any_risk	.8629611	.2652524	-0.48	0.632	.4724473	1.576264
2.high_risk_num	1.664787	1.116654	0.76	0.447	.4471117	6.198709
highriskatbirth						
Y	.8888816	.2524653	-0.41	0.678	.5094256	1.550983
place_hosp_comm						
hospital	.2463731	.11066	-3.12	0.002	.1021577	.5941768
imp_gstt						
imp	130.1612	68.8159	9.21	0.000	46.17993	366.8679
_cons	3.50e-09	4.07e-06	-0.02	0.987	0	.
3						
mod_care_4						
standard	.3452801	.1990772	-1.84	0.065	.1115313	1.068923
partial CoC	.8252684	.5081991	-0.31	0.755	.2468462	2.759078
ethnicity						
BA	.4432498	.2848524	-1.27	0.205	.1257847	1.561957
BC	.3192784	.3801943	-0.96	0.338	.0309432	3.294382
BO	.5940215	.7219469	-0.43	0.668	.0548643	6.431527
M	2.919087	2.948978	1.06	0.289	.4030225	21.1429
U	.9494037	.5401176	-0.09	0.927	.3113155	2.89535
WB	1.232423	.7186215	0.36	0.720	.393031	3.864492
WO	1.116556	.5604128	0.22	0.826	.4174958	2.98613
WP	.2176232	3289.857	-0.00	1.000	0	.
age_cat						
20-24	4070954	6.23e+09	0.01	0.992	0	.
25-29	1.60e+07	2.45e+10	0.01	0.991	0	.
30-34	8761015	1.34e+10	0.01	0.992	0	.
greater than 34	1.37e+07	2.10e+10	0.01	0.991	0	.
0.parity	1.645874	.5272247	1.56	0.120	.878477	3.083635
imd_3_score						
most deprived	2.243444	1.183107	1.53	0.125	.7980385	6.306765
3rd and 4th deciles	1.962508	.9973083	1.33	0.185	.724849	5.313436
5th and 6th deciles	1.874744	1.010673	1.17	0.244	.651718	5.392923
1.any_risk	1.249665	.4504208	0.62	0.536	.6165841	2.532765
2.high_risk_num	2.051514	1.692983	0.87	0.384	.4070358	10.3399
highriskatbirth						
Y	.9102559	.3097016	-0.28	0.782	.4672554	1.773261
place_hosp_comm						
hospital	.232326	.1178467	-2.88	0.004	.0859663	.6278666
imp_gstt						
imp	158.5037	106.957	7.51	0.000	42.23336	594.8719
_cons	1.50e-09	2.29e-06	-0.01	0.989	0	.
4						
mod_care_4						
standard	.1071422	.087908	-2.72	0.006	.0214569	.5350006
partial CoC	1.723088	1.290399	0.73	0.467	.3970573	7.47759
ethnicity						
BA	.303219	.2529282	-1.43	0.153	.0591197	1.55518
BC	1.391403	1.353061	0.34	0.734	.2068748	9.358329
BO	1.017385	1.394737	0.01	0.990	.0692739	14.94173
M	3.92e-06	.0041128	-0.01	0.991	0	.
U	1.080812	.7921639	0.11	0.916	.256966	4.545951
WB	1.750317	1.226933	0.80	0.425	.4430405	6.914965
WO	.6510023	.4259005	-0.66	0.512	.1805956	2.346702
WP	.8365924	16015.77	-0.00	1.000	0	.
age_cat						
20-24	1.13e+07	2.01e+10	0.01	0.993	0	.
25-29	9275790	1.65e+10	0.01	0.993	0	.
30-34	1.53e+07	2.72e+10	0.01	0.993	0	.
greater than 34	7097596	1.26e+10	0.01	0.993	0	.
0.parity	1.553959	.6429303	1.07	0.287	.6906625	3.496335
imd_3_score						
most deprived	2.797857	1.835835	1.57	0.117	.7732198	10.1239
3rd and 4th deciles	1.791035	1.137307	0.92	0.359	.5159303	6.217517
5th and 6th deciles	1.423641	.9797758	0.51	0.608	.3694775	5.485459
1.any_risk	.9706877	.4723698	-0.06	0.951	.3739863	2.519436
2.high_risk_num	5.072918	6.282963	1.31	0.190	.4477329	57.47735
highriskatbirth						
Y	1.834165	.8026277	1.39	0.166	.777954	4.32437
place_hosp_comm						
hospital	.306421	.1568288	-2.31	0.021	.1123742	.8355462
imp_gstt						
imp	293.5531	331.2947	5.03	0.000	32.14013	2681.178
_cons	3.35e-10	5.95e-07	-0.01	0.990	0	.
5						
mod_care_4						
standard	.0254159	.019587	-4.77	0.000	.005612	.1151047
partial CoC	.8291245	.5360572	-0.29	0.772	.2335012	2.944086
ethnicity						
BA	.1758425	.1385612	-2.21	0.027	.037531	.8238682
BC	.5001275	.5111954	-0.68	0.498	.0674593	3.707827
BO	1.5265	1.869733	0.35	0.730	.1383895	16.838
M	2.457489	3.421256	0.65	0.518	.1604981	37.6282
U	.8435105	.6012533	-0.24	0.811	.2086195	3.410562
WB	1.151793	.7796169	0.21	0.835	.3056468	4.340395
WO	.4221212	.2640412	-1.38	0.168	.1238801	1.438376
WP	1.229522	22017.75	0.00	1.000	0	.
age_cat						
20-24	2.161349	3.465587	0.48	0.631	.093299	50.06944
25-29	3.170843	5.019867	0.73	0.466	.1424326	70.58951
30-34	3.298027	5.106225	0.77	0.441	.1586235	68.57108
greater than 34	4.282206	6.658542	0.94	0.350	.2032856	90.20458
0.parity	.8574591	.3721807	-0.35	0.723	.3662257	2.007604
imd_3_score						
most deprived	3.896169	2.551882	2.08	0.038	1.079256	14.06537
3rd and 4th deciles	1.732054	1.141455	0.83	0.405	.475997	6.302582
5th and 6th deciles	1.696077	1.193329	0.75	0.453	.4271259	6.734965
1.any_risk	1.753116	.8077984	1.22	0.223	.7105512	4.325399
2.high_risk_num	3.22637	4.079044	0.93	0.354	.2707415	38.44798
highriskatbirth						
Y	2.248574	.9914991	1.84	0.066	.9474877	5.336308
place_hosp_comm						
hospital	.3523471	.1807112	-2.03	0.042	.128946	.9627944
imp_gstt						
imp	366.4902	422.7924	5.12	0.000	38.20274	3515.848
_cons	.0019683	.0042857	-2.86	0.004	.0000276	.1404198

Table 11: Number of appointments with a known healthcare professional in relation to place of care

deliver_named_mw	RRR	Std. Err.	z	P>|z|	[95% Conf.	Interval]
**yes**						
place_hosp_comm						
hospital	.8999399	.1918592	-0.49	0.621	.5925771	1.366728
ethnicity						
BA	1.107203	.4571936	0.25	0.805	.4928825	2.487202
BC	.8993323	.4435732	-0.22	0.830	.3420474	2.36458
BO	1.875314	1.157879	1.02	0.309	.5591325	6.289745
M	1.222475	.8163165	0.30	0.764	.3302511	4.525181
U	1.193273	.4079359	0.52	0.605	.6105841	2.33203
WB	1.595939	.5570766	1.34	0.181	.8051871	3.163266
WO	.8055546	.2648301	-0.66	0.511	.42292	1.534376
WP	.0000219	.0132068	-0.02	0.986	0	.
age_cat						
20-24	.094338	.0939308	-2.37	0.018	.0134017	.6640707
25-29	.1635071	.157408	-1.88	0.060	.0247791	1.078915
30-34	.1665161	.1580722	-1.89	0.059	.0259071	1.070272
greater than 34	.1411916	.1340099	-2.06	0.039	.0219737	.9072226
1.parity	1.07912	.212864	0.39	0.699	.7331014	1.588456
imd_3_score						
3rd and 4th deciles	.9407191	.2249157	-0.26	0.798	.5887716	1.503049
5th and 6th deciles	.8024294	.2263074	-0.78	0.435	.4616835	1.394663
least deprived	1.089825	.3345874	0.28	0.779	.5970758	1.989227
1.any_risk	1.142474	.2769763	0.55	0.583	.7103706	1.837418
2.high_risk_num	1.267832	.4231819	0.71	0.477	.6590892	2.438816
highriskatbirth						
Y	.4433954	.1015607	-3.55	0.000	.2830229	.6946416
mod_care_4						
standard	.5923736	.206196	-1.50	0.133	.2994372	1.171887
partial CoC	.4508229	.1653001	-2.17	0.030	.2197363	.9249327
imp_gstt						
imp	.0891558	.0219513	-9.82	0.000	.0550266	.1444528
_cons	44.86345	46.44374	3.67	0.000	5.898045	341.2536
**no**	(base outcome)					

noofapptswithknown~p	RRR	Std. Err.	z	P>|z|	[95% Conf.	Interval]
0	(base outcome)					
2						
place_hosp_comm						
hospital	.2467317	.1107829	-3.12	0.002	.1023374	.5948611
ethnicity						
BA	.6924964	.3690025	-0.69	0.490	.2436956	1.967829
BC	1.738724	1.210712	0.79	0.427	.4441403	6.806772
BO	1.67289	1.295196	0.66	0.506	.3668126	7.6294
M	3.508557	3.064964	1.44	0.151	.6332072	19.44067
U	1.182606	.5555711	0.36	0.721	.4709314	2.969765
WB	1.160279	.5821279	0.30	0.767	.4340132	3.101858
WO	1.174625	.5071569	0.37	0.709	.503946	2.737881
WP	2.05e+07	1.16e+11	0.00	0.998	0	.
age_cat						
20-24	2071967	2.40e+09	0.01	0.990	0	.
25-29	4946104	5.74e+09	0.01	0.989	0	.
30-34	5173676	6.00e+09	0.01	0.989	0	.
greater than 34	3587930	4.16e+09	0.01	0.990	0	.
1.parity	.5471574	.1444873	-2.28	0.022	.3260891	.9180962
imd_3_score						
3rd and 4th deciles	1.127121	.3644407	0.37	0.711	.5980605	2.124203
5th and 6th deciles	1.121483	.4141216	0.31	0.756	.5438459	2.312648
least deprived	1.000974	.4163176	0.00	0.998	.4429937	2.261769
1.any_risk	.8623928	.2650129	-0.48	0.630	.4722057	1.574994
2.high_risk_num	1.668212	1.119368	0.76	0.446	.4478124	6.214504
highriskatbirth						
Y	.8882196	.2522359	-0.42	0.676	.5090926	1.549687
mod_care_4						
standard	2.13658	1.428092	1.14	0.256	.5764682	7.918868
partial CoC	1.819327	1.310825	0.83	0.406	.4432208	7.467951
imp_gstt						
imp	129.3069	68.29544	9.21	0.000	45.92469	364.0801
_cons	6.43e-09	7.46e-06	-0.02	0.987	0	.
3						
place_hosp_comm						
hospital	.2324609	.117893	-2.88	0.004	.0860323	.6281138
ethnicity						
BA	.4448746	.2859349	-1.26	0.208	.1262245	1.567948
BC	.3192713	.3800974	-0.96	0.338	.0309593	3.292522
BO	.5933663	.7209856	-0.43	0.668	.0548337	6.420933
M	2.913797	2.942518	1.06	0.290	.4025942	21.08876
U	.9475622	.5389036	-0.09	0.925	.3108185	2.88874
WB	1.240831	.7237501	0.37	0.711	.3955715	3.892245
WO	1.116068	.5599861	0.22	0.827	.4174467	2.983873
WP	.2178736	3262.333	-0.00	1.000	0	.
age_cat						
20-24	4050570	6.18e+09	0.01	0.992	0	.
25-29	1.59e+07	2.43e+10	0.01	0.991	0	.
30-34	8673986	1.32e+10	0.01	0.992	0	.
greater than 34	1.36e+07	2.08e+10	0.01	0.991	0	.
1.parity	.6059389	.1940902	-1.56	0.118	.3234282	1.135219
imd_3_score						
3rd and 4th deciles	.8780057	.3331795	-0.34	0.732	.4173393	1.847164
5th and 6th deciles	.8363101	.3654243	-0.41	0.682	.35517	1.969239
least deprived	.445141	.2346754	-1.54	0.125	.158398	1.250966
1.any_risk	1.249047	.4500963	0.62	0.537	.6163776	2.531108
2.high_risk_num	2.048627	1.690593	0.87	0.385	.4064657	10.32528
highriskatbirth						
Y	.9095566	.3094149	-0.28	0.780	.4669455	1.771713
mod_care_4						
standard	.3472074	.2002005	-1.83	0.067	.1121461	1.074963
partial CoC	.8268466	.5089512	-0.31	0.757	.2474472	2.762914
imp_gstt						
imp	157.4338	106.1708	7.50	0.000	41.98182	590.384
_cons	5.57e-09	8.51e-06	-0.01	0.990	0	.
4						
place_hosp_comm						
hospital	.3067113	.1569143	-2.31	0.021	.112526	.8360008
ethnicity						
BA	.3042188	.2537595	-1.43	0.154	.0593156	1.560281
BC	1.390508	1.351873	0.34	0.735	.2068341	9.348128
BO	1.016634	1.393221	0.01	0.990	.0692878	14.9167
M	3.92e-06	.0041111	-0.01	0.991	0	.
U	1.078893	.7905951	0.10	0.917	.2565851	4.536542
WB	1.759525	1.233466	0.81	0.420	.4453323	6.951948
WO	.6506795	.4255932	-0.66	0.511	.1805583	2.344859
WP	.8377351	15882.32	-0.00	1.000	0	.
age_cat						
20-24	1.13e+07	2.00e+10	0.01	0.993	0	.
25-29	9189086	1.63e+10	0.01	0.993	0	.
30-34	1.52e+07	2.68e+10	0.01	0.993	0	.
greater than 34	7048785	1.25e+10	0.01	0.993	0	.
1.parity	.641973	.2655857	-1.07	0.284	.2853466	1.444311
imd_3_score						
3rd and 4th deciles	.6420809	.3098632	-0.92	0.359	.2493483	1.653381
5th and 6th deciles	.5092814	.2955374	-1.16	0.245	.1633064	1.588226
least deprived	.3570741	.2342331	-1.57	0.116	.0987161	1.291602
1.any_risk	.9700013	.4719995	-0.06	0.950	.3737493	2.51747
2.high_risk_num	5.082526	6.295067	1.31	0.189	.4485454	57.59076
highriskatbirth						
Y	1.833258	.8021245	1.39	0.166	.7776577	4.32174
mod_care_4						
standard	.1076106	.0883012	-2.72	0.007	.0215472	.5374261
partial CoC	1.724272	1.290989	0.73	0.467	.3974641	7.480207
imp_gstt						
imp	292.8011	330.4324	5.03	0.000	32.06074	2674.065
_cons	1.47e-09	2.60e-06	-0.01	0.991	0	.
5						
place_hosp_comm						
hospital	.3526896	.180827	-2.03	0.042	.1291142	.9634099
ethnicity						
BA	.1763719	.1389716	-2.20	0.028	.0376468	.8262865
BC	.4999369	.5108733	-0.68	0.497	.0674673	3.704565
BO	1.525588	1.867909	0.34	0.730	.1384323	16.81267
M	2.455025	3.417835	0.65	0.519	.1603359	37.59076
U	.8423045	.6002791	-0.24	0.810	.2083768	3.404778
WB	1.157315	.7833931	0.22	0.829	.3070921	4.361488
WO	.4219462	.2638713	-1.38	0.168	.1238636	1.437376
WP	1.23247	21857.73	0.00	1.000	0	.
age_cat						
20-24	2.160438	3.462691	0.48	0.631	.0933811	49.98325
25-29	3.158476	4.997976	0.73	0.467	.1420808	70.21337
30-34	3.283103	5.08069	0.77	0.442	.1581348	68.16189
greater than 34	4.27602	6.645841	0.93	0.350	.2032789	89.94712
1.parity	1.163698	.5050895	0.35	0.727	.4970344	2.724547
imd_3_score						
3rd and 4th deciles	.4457214	.2104513	-1.71	0.087	.1766693	1.124516
5th and 6th deciles	.4357005	.2474089	-1.46	0.143	.1431661	1.325976
least deprived	.256448	.1679203	-2.08	0.038	.0710622	.9254647
1.any_risk	1.752219	.8072836	1.22	0.223	.7102678	4.322695
2.high_risk_num	3.227525	4.080159	0.93	0.354	.2708952	38.45369
highriskatbirth						
Y	2.247512	.9909025	1.84	0.066	.9471462	5.333191
mod_care_4						
standard	.0255008	.0196506	-4.76	0.000	.0056316	.1154723
partial CoC	.8295332	.5361767	-0.29	0.772	.2336962	2.94453
imp_gstt						
imp	365.364	421.434	5.12	0.000	38.09746	3503.93
_cons	.0065914	.0132469	-2.50	0.012	.0001283	[illegible]

only

Table 12: Number of missed appointments in relation to model of care received

For peer review only

miss_appt_4_cat	RRR	Std. Err.	z	P>|z|	[95% Conf.	Interval]
0	(base outcome)					
1						
mod_care_4						
standard	1.466618	.6491433	0.87	0.387	.6159775	3.491961
partial CoC	1.976347	.9047997	1.49	0.137	.8056957	4.847919
ethnicity						
BA	2.485515	1.125998	2.01	0.044	1.022826	6.03992
BC	1.94952	1.063413	1.22	0.221	.6692974	5.678536
BO	.8342489	.7124188	-0.21	0.832	.1564588	4.448272
M	.7729222	.6665585	-0.30	0.765	.1425838	4.189878
U	.9045829	.3983115	-0.23	0.820	.3816297	2.144147
WB	.909001	.4042185	-0.21	0.830	.3802318	2.173103
WO	1.45843	.5647411	0.97	0.330	.6827766	3.115247
WP	1.20e-06	.0036594	-0.00	0.996	0	.
age_cat						
20-24	.2842646	.2541574	-1.41	0.159	.0492804	1.639726
25-29	.2027736	.172672	-1.87	0.061	.0382094	1.0761
30-34	.2833981	.2325385	-1.54	0.124	.0567485	1.415269
greater than 34	.2004958	.1662139	-1.94	0.053	.0394864	1.018037
0.parity	.7442038	.1735291	-1.27	0.205	.4712092	1.175358
imd_3_score						
most deprived	1.779518	.6478759	1.58	0.113	.8717703	3.632474
3rd and 4th deciles	.7533867	.2805336	-0.76	0.447	.3631296	1.563055
5th and 6th deciles	1.253867	.4778692	0.59	0.553	.5940794	2.646417
1.any_risk	1.38075	.3691036	1.21	0.227	.8176568	2.331628
2.high_risk_num	.5591242	.2403068	-1.35	0.176	.2408071	1.298217
place_hosp_comm						
hospital	.9538957	.2571112	-0.18	0.861	.5624315	1.617827
imp_gstt						
imp	1.740019	.4995678	1.93	0.054	.99122	3.054485
_cons	.3266529	.3430343	-1.07	0.287	.0417059	2.558439
2						
mod_care_4						
standard	1.819736	1.136977	0.96	0.338	.5347799	6.192154
partial CoC	.8625367	.5949905	-0.21	0.830	.2231548	3.333872
ethnicity						
BA	11.34923	9.569888	2.88	0.004	2.173787	59.25374
BC	3.389846	3.648733	1.13	0.257	.4111224	27.95045
BO	7.72868	7.823851	2.02	0.043	1.062728	56.20676
M	6.162043	6.890638	1.63	0.104	.6884586	55.15332
U	6.547095	5.306048	2.32	0.020	1.337206	32.05524
WB	4.231173	3.552244	1.72	0.086	.8162852	21.93207
WO	3.399138	2.703933	1.54	0.124	.7149213	16.16141
WP	2.12e-06	.0091325	-0.00	0.998	0	.
age_cat						
20-24	.9444105	1.201994	-0.04	0.964	.0779468	11.44257
25-29	.8079029	1.015	-0.17	0.865	.0688591	9.478885
30-34	.3067858	.3842996	-0.94	0.346	.026337	3.573591
greater than 34	.2556753	.3218523	-1.08	0.279	.0216855	3.014454
0.parity	1.016398	.3258644	0.05	0.960	.5422039	1.905307
imd_3_score						
most deprived	.4342183	.2256039	-1.61	0.108	.1568393	1.202157
3rd and 4th deciles	.5644775	.2685964	-1.20	0.229	.2221354	1.434417
5th and 6th deciles	.6803869	.3456319	-0.76	0.448	.251392	1.841452
1.any_risk	2.112226	.7313112	2.16	0.031	1.071595	4.163417
2.high_risk_num	1.848433	1.227859	0.92	0.355	.5027771	6.795662
place_hosp_comm						
hospital	.6361429	.2928467	-0.98	0.326	.2580514	1.568206
imp_gstt						
imp	10.41672	5.60486	4.36	0.000	3.628525	29.90417
_cons	.0143837	.0240619	-2.54	0.011	.0005419	.3817767
3						
mod_care_4						
standard	2.029736	2.288317	0.63	0.530	.22274	18.49613
partial CoC	2.438196	2.827234	0.77	0.442	.2512174	23.66398
ethnicity						
BA	2.626047	2.073478	1.22	0.221	.5587396	12.34229
BC	1.201955	1.499941	0.15	0.883	.1041498	13.87132
BO	1.032836	1.324027	0.03	0.980	.0837234	12.74135
M	1.53e-06	.0016317	-0.01	0.990	0	.
U	.5783578	.5561831	-0.57	0.569	.0878276	3.808574
WB	1.778301	1.417697	0.72	0.470	.3727437	8.483993
WO	1.196887	.8684031	0.25	0.804	.2887104	4.96185
WP	1.60e-06	.0100035	-0.00	0.998	0	.
age_cat						
20-24	946768.2	2.68e+09	0.00	0.996	0	.
25-29	1081410	3.07e+09	0.00	0.996	0	.
30-34	1760311	4.99e+09	0.01	0.996	0	.
greater than 34	472766.8	1.34e+09	0.00	0.996	0	.
0.parity	.7531945	.3418027	-0.62	0.532	.309477	1.833098
imd_3_score						
most deprived	1.474234	1.08819	0.53	0.599	.3469455	6.264289
3rd and 4th deciles	.8275671	.6216565	-0.25	0.801	.1898423	3.60756
5th and 6th deciles	1.433326	1.086795	0.47	0.635	.3242916	6.335112
1.any_risk	2.895147	1.356331	2.27	0.023	1.155836	7.251788
2.high_risk_num	1.272324	1.592764	0.19	0.847	.1094001	14.79715
place_hosp_comm						
hospital	1.023997	.5942124	0.04	0.967	.3283651	3.193303
imp_gstt						
imp	9.324685	7.681576	2.71	0.007	1.85534	46.8646
_cons	2.80e-09	7.93e-06	-0.01	0.994	0	.
4						
mod_care_4						
standard	.2309158	.1969309	-1.72	0.086	.0434038	1.228514
partial CoC	.4939632	.4295315	-0.81	0.417	.0898509	2.715607
ethnicity						
BA	.6189749	.6022693	-0.49	0.622	.0919274	4.167747
BC	.3402144	.4352897	-0.84	0.399	.0277126	4.176653
BO	7.92e-07	.0011195	-0.01	0.992	0	.
M	4.08e-07	.0004059	-0.01	0.988	0	.
U	.7025714	.5593681	-0.44	0.657	.1475663	3.344983
WB	.373482	.3541876	-1.04	0.299	.0582159	2.396058
WO	.2917438	.2447006	-1.47	0.142	.0563709	1.509901
WP	3.24e-06	.0258196	-0.00	0.999	0	.
age_cat						
20-24	.1451364	.2109871	-1.33	0.184	.0084018	2.507158
25-29	.0522817	.0791982	-1.95	0.051	.0026848	1.018076
30-34	.040073	.059397	-2.17	0.030	.0021937	.7320142
greater than 34	.0252698	.0376016	-2.47	0.013	.0013678	.4668686
0.parity	.2194832	.1566993	-2.12	0.034	.0541613	.8894338
imd_3_score						
most deprived	1.402094	1.292939	0.37	0.714	.230061	8.544987
3rd and 4th deciles	.6948283	.6501306	-0.39	0.697	.1110281	4.348325
5th and 6th deciles	.8176601	.901942	-0.18	0.855	.0941092	7.104175
1.any_risk	1.932104	1.203268	1.06	0.290	.5700627	6.548446
2.high_risk_num	2.565955	2.56353	0.94	0.346	.3621195	18.18219
place_hosp_comm						
hospital	.3593408	.2686003	-1.37	0.171	.0830328	1.555118
imp_gstt						
imp	7.903945	7.665181	2.13	0.033	1.181288	52.88495
_cons	1.920439	3.991262	0.31	0.754	.0326846	112.8388

iew only

Table 13: Number of missed appointments in relation to place of care

miss_appt_4_cat	RRR	Std. Err.	z	P>|z|	[95% Conf.	Interval]
0	(base outcome)					
1						
place_hosp_comm						
hospital	.9569555	.2576891	-0.16	0.870	.564521	1.622196
ethnicity						
BA	2.517118	1.14186	2.03	0.042	1.034586	6.124078
BC	1.946001	1.061045	1.22	0.222	.6683913	5.665725
BO	.8344806	.7122503	-0.21	0.832	.1566369	4.445681
M	.7755741	.6686059	-0.29	0.768	.1431597	4.201709
U	.9020032	.397027	-0.23	0.815	.3806642	2.137342
WB	.9135987	.4062334	-0.20	0.839	.3821793	2.183956
WO	1.456576	.5637853	0.97	0.331	.6821274	3.110291
WP	1.19e-06	.0036291	-0.00	0.996	0	.
age_cat						
20-24	.287718	.2572229	-1.39	0.163	.0498866	1.659396
25-29	.202999	.1727676	-1.87	0.061	.0382875	1.076294
30-34	.2834254	.2324265	-1.54	0.124	.0568068	1.41409
greater than 34	.2015995	.1670383	-1.93	0.053	.0397387	1.02274
1.parity	1.333352	.3112723	1.23	0.218	.8437839	2.106972
imd_3_score						
3rd and 4th deciles	.425283	.1189227	-3.06	0.002	.2458414	.7357007
5th and 6th deciles	.7094975	.2181208	-1.12	0.264	.3883882	1.296092
least deprived	.5615893	.2044222	-1.59	0.113	.275154	1.146204
1.any_risk	1.376409	.3679088	1.20	0.232	.8151256	2.324183
2.high_risk_num	.5711377	.2460416	-1.30	0.194	.2454991	1.328715
mod_care_4						
standard	1.473125	.6518644	0.88	0.381	.6188412	3.506712
partial CoC	1.979531	.9058473	1.49	0.136	.8073215	4.853758
imp_gstt						
imp	1.736336	.4981058	1.92	0.054	.9895738	3.046629
_cons	.4301065	.4174436	-0.87	0.385	.0641852	2.882153
2						
place_hosp_comm						
hospital	.6444555	.296171	-0.96	0.339	.2618232	1.586272
ethnicity						
BA	11.49696	9.698098	2.90	0.004	2.200715	60.06232
BC	3.370232	3.627379	1.13	0.259	.4088011	27.78481
BO	7.69689	7.790642	2.02	0.044	1.058634	55.96093
M	6.204943	6.936276	1.63	0.102	.6937629	55.49636
U	6.504876	5.271353	2.31	0.021	1.328774	31.84394
WB	4.261376	3.577301	1.73	0.084	.8222254	22.08558
WO	3.390012	2.696209	1.53	0.125	.7131934	16.1137
WP	2.10e-06	.0090408	-0.00	0.998	0	.
age_cat						
20-24	.9640915	1.227325	-0.03	0.977	.0795255	11.68773
25-29	.8111337	1.018517	-0.17	0.868	.069225	9.504333
30-34	.3079009	.3854809	-0.94	0.347	.026469	3.581664
greater than 34	.2579331	.3245363	-1.08	0.281	.0219033	3.037422
1.parity	.9754309	.3129515	-0.08	0.938	.5201182	1.829325
imd_3_score						
3rd and 4th deciles	1.306187	.5227048	0.67	0.504	.5961767	2.861777
5th and 6th deciles	1.578365	.7246758	0.99	0.320	.6417928	3.881684
least deprived	2.29793	1.193699	1.60	0.109	.830167	6.360747
1.any_risk	2.099106	.7269789	2.14	0.032	1.06473	4.138368
2.high_risk_num	1.91287	1.273571	0.97	0.330	.5187563	7.053547
mod_care_4						
standard	1.829594	1.142745	0.97	0.333	.5379022	6.22309
partial CoC	.8694436	.5991082	-0.20	0.839	.22527	3.355672
imp_gstt						
imp	10.31898	5.539971	4.35	0.000	3.602889	29.55446
_cons	.0062786	.0099685	-3.19	0.001	.0002795	.1410296
3						
place_hosp_comm						
hospital	1.027215	.5956154	0.05	0.963	.3296895	3.200501
ethnicity						
BA	2.648138	2.091771	1.23	0.218	.5630855	12.45394
BC	1.198983	1.495906	0.15	0.884	.1039478	13.82964
BO	1.033182	1.324002	0.03	0.980	.0838262	12.73427
M	1.54e-06	.0016388	-0.01	0.990	0	.
U	.5769185	.5547314	-0.57	0.567	.0876291	3.798224
WB	1.78182	1.420415	0.72	0.469	.3735172	8.499963
WO	1.194865	.866795	0.25	0.806	.2882896	4.952322
WP	1.59e-06	.0099383	-0.00	0.998	0	.
age_cat						
20-24	941966.5	2.65e+09	0.00	0.996	0	.
25-29	1064254	2.99e+09	0.00	0.996	0	.
30-34	1731111	4.87e+09	0.01	0.996	0	.
greater than 34	466779.2	1.31e+09	0.00	0.996	0	.
1.parity	1.320394	.599513	0.61	0.540	.5422793	3.215022
imd_3_score						
3rd and 4th deciles	.563164	.3030157	-1.07	0.286	.1961723	1.616709
5th and 6th deciles	.976508	.5736932	-0.04	0.968	.3087447	3.088532
least deprived	.6780669	.500478	-0.53	0.599	.1595896	2.880981
1.any_risk	2.886719	1.35232	2.26	0.024	1.15252	7.230371
2.high_risk_num	1.296785	1.62442	0.21	0.836	.1113293	15.10521
mod_care_4						
standard	2.037241	2.296359	0.63	0.528	.2236537	18.55704
partial CoC	2.44362	2.832502	0.77	0.441	.2519827	23.69718
imp_gstt						
imp	9.301962	7.659612	2.71	0.007	1.852085	46.71844
_cons	3.15e-09	8.84e-06	-0.01	0.994	0	.
4						
place_hosp_comm						
hospital	.3605907	.2694494	-1.37	0.172	.0833602	1.559804
ethnicity						
BA	.6230185	.6064412	-0.49	0.627	.0924588	4.198108
BC	.3400471	.4350152	-0.84	0.399	.0277086	4.173147
BO	7.95e-07	.0011212	-0.01	0.992	0	.
M	4.11e-07	.0004077	-0.01	0.988	0	.
U	.70192	.5587515	-0.44	0.657	.1474698	3.340967
WB	.3747134	.3553621	-1.04	0.301	.0584059	2.404042
WO	.2916114	.244571	-1.47	0.142	.0563523	1.509027
WP	3.22e-06	.0256849	-0.00	0.999	0	.
age_cat						
20-24	.1464599	.2129082	-1.32	0.186	.0084787	2.52992
25-29	.052439	.0794202	-1.95	0.052	.0026946	1.020519
30-34	.0401708	.0595274	-2.17	0.030	.0022007	.733278
greater than 34	.025392	.0377773	-2.47	0.014	.001375	.4689034
1.parity	4.534777	3.238673	2.12	0.034	1.11851	18.38535
imd_3_score						
3rd and 4th deciles	.496867	.3204713	-1.08	0.278	.1403553	1.758942
5th and 6th deciles	.5848716	.510162	-0.61	0.539	.1058251	3.232454
least deprived	.7131029	.6575218	-0.37	0.714	.1170295	4.345191
1.any_risk	1.928033	1.200607	1.05	0.292	.5689343	6.533816
2.high_risk_num	2.581789	2.580332	0.95	0.343	.3640824	18.30804
mod_care_4						
standard	.2315544	.197482	-1.72	0.086	.0435214	1.231979
partial CoC	.494682	.4300472	-0.81	0.418	.0900206	2.718381
imp_gstt						
imp	7.884151	7.64327	2.13	0.033	1.179125	52.71693
_cons	.5883176	1.026983	-0.30	0.761	.019219	18.00909

w only

Testing using qualitative data: Firstly we explored why women receiving care in the hospital-based model might have less appointments with a known healthcare professional than those in the community-based model. One explanation for this appeared to be that women in the hospital-based model were referred to the specialist model after disclosing social risk factors during their pregnancy. This would have an impact on the number of appointments they had with a known healthcare professional depending on their gestation at the time of referral to the specialist model. Although it was often seen as a positive referral, only women in the hospital-based model described care being disrupted through a referral to the specialist model. Flexibility was discussed though women's ability to rebook missed appointments easily and without reproach. Women in both models described feeling comfortable when rebooking appointments. This ability to reschedule may reduce the number of 'missed' appointments recorded, seen as a mitigating effect of the specialist model.

Table 14: Qualitative quotations relating to women's experiences of continuity of care, missing and rescheduling appointments

'I met all of them when I was going for my appointments at [Community centre]. I met all of them before I gave birth…and when I gave birth all of them came to see me at home one by one…. they was taking care of me. Every time when I'm at home I call them, they pick up my call, they know everything about me. Yeah but not the midwife at hospital.' (CBM7)<br><br>*'There are six of them [CBM midwives]… I met all of them, when I have the baby there going to be one of them… in the hospital, which is I never heard this. Which is good, they know you and then they know all the, the problem or the issues you have'* (CBM2)<br><br>*'sometimes if I was getting anxious and worried, um, I felt like I didn't want to bother or inconvenience [named midwife] even though I had her mobile number and I think in the beginning I did try to utilise it but it, she wasn't either at work or someone else would pick up from the team and say, 'Oh she's not here.' And so that would kind of knock my confidence, so I wouldn't kind of ring her. I just get told that they're rushed off their feet, you have one midwife to six, seven ladies when they're all in active labour, so I don't, I wouldn't think that they would be there for 24 hours, I think the kind of contact is very minimal. the midwife kind of works like nine to five. And I know that she, um, isn't in some days, so any type of concerns like that I'll just rather ring the triage'* (HBM8)<br><br>*'because she [HBM midwife] says she will be off…so I didn't want to call another midwife and explain everything, so I just went straight… to the GP because I was feeling bad, yeah, the GP, yeah she say that I had to go back to the*

For peer review only

hospital… in the Emergency they say because I was five days after the operation they cannot see me, so they send me to the labour ward. (HBM10)

'That is something that does worry me a little bit is like who's going to come [to provide care at homebirth]?...obviously you don't know when the baby's going to come so you don't know a hundred percent who's going to come round to help you. So that's a bit … daunting. (HBM2)

'she [midwife] cancelled a lot of my appointments….I understand that people have babies. but it meant that it was cancelled appointments again and again and again… I only had maybe one or two midwife appointments during my pregnancy…. it suffered me because I didn't go to antenatal classes, because she didn't really sit there and go, 'Well this is what's available'…. so because of me not having the right sort of preparation for the baby it kind of led to bigger things being involved because they [social care] were scared that I wasn't picking up cues and I wasn't doing this, and I wasn't doing that… It was an incredibly stressful time for me… And when I sat there and said, 'Look I didn't have any antenatal care,' they were a little bit more understanding of why I wasn't picking up on those cues'(HBM1)

'because of my situation I called them to change it…. if it's not a convenient time, I'm happy to change it.' (CMB1)

'because they say you have to come, I tried to squeeze my plan, to be attending as much as I can…just now because of my situation I called them to change it…. if it's not a convenient time, I'm happy to change it.' (CMB1)

'I like her [CBM midwife], I feel open to talk all to her, yeah. Because the first appointment I missed so she said, 'OK I can, if you are free on Saturday I come on Saturday,' And she came to see me... at home.' (CBM7)

'I think there was one week where, because I was going out of London back to [region].. I was like, 'I can't do this appointment, could we do it a week early?' And they did and it was fine. Um, which was obviously really helpful' (CBM9)

She's perfect when it comes to, um, appointments or anything like that, and if I'm running late or something comes up I just text her or give her a call and she'll muddle things around (HBM9)

'she [midwife] cancelled a lot of my appointments….I understand that people have babies. but it meant that it was cancelled appointments again and again and again… I only had maybe one or two midwife appointments during my pregnancy…. it suffered me because I didn't go to antenatal classes, because she didn't really sit there and go, 'Well this

For peer review only

is what's available'…. so because of me not having the right sort of preparation for the baby it kind of led to bigger things being involved because they [social care] were scared that I wasn't picking up cues and I wasn't doing this, and I wasn't doing that… It was an incredibly stressful time for me… And when I sat there and said, 'Look I didn't have any antenatal care,' they were a little bit more understanding of why I wasn't picking up on those cues…'(HBM1)

The data confirmed aspects of both the initial programme theories and the rival theories. Therefore the refined theory aims to reflect these conflicting ideas:

Refined theory: If a small team of midwives provide continued supportive presence throughout pregnancy and the perinatal period and prepare women for labour and birth through education and gaining familiarity of birth settings, then they will feel better supported and able to seek help and exercise choice, resulting in improved safety and reduced feelings of anxiety. When a named midwife is unable to be present for labour care, women with social risk factors should be cared for by a healthcare professional who is aware of their circumstances and individual needs to avoid disrespectful care and the risk of triggering previous experiences of trauma.

Women's insights around missed appointments confirmed the initial programme theory and enabled the development of a new programme theory relating to how midwives in specialist models share workloads.

Refined theory: If women are able to reschedule appointments easily, and do not fear judgement or reproach when they miss appointments then they will perceive the maternity environment as a place of safety and their engagement with flexible services will improve.

Refined theory: If women have the opportunity to get to know the all midwives in a small team throughout their pregnancy, then they will not feel disappointment or let down when their named midwife is unable to attend an appointment, and care, information and responsibility will be shared across the team, thus improving safety.

For peer review only

Reporting checklist for quality improvement in health care.

Based on the SQUIRE guidelines.

Instructions to authors

Complete this checklist by entering the page numbers from your manuscript where readers will find each of the items listed below.

Your article may not currently address all the items on the checklist. Please modify your text to include the missing information. If you are certain that an item does not apply, please write "n/a" and provide a short explanation.

Upload your completed checklist as an extra file when you submit to a journal.

In your methods section, say that you used the SQUIREreporting guidelines, and cite them as:

Ogrinc G, Davies L, Goodman D, Batalden P, Davidoff F, Stevens D. SQUIRE 2.0 (Standards for QUality Improvement Reporting Excellence): revised publication guidelines from a detailed consensus process

		Reporting Item	Page Number
**Title**			
	#1	Indicate that the manuscript concerns an initiative to improve healthcare (broadly defined to include the quality, safety, effectiveness, patientcenteredness, timeliness, cost, efficiency, and equity of healthcare)	1
**Abstract**			
	#02a	Provide adequate information to aid in searching and indexing	2
	#02b	Summarize all key information from various sections of the text using the abstract format of the intended publication or a structured summary such as: background, local problem, methods, interventions, results, conclusions	2
**Introduction**			
Problem description	#3	Nature and significance of the local problem	3

For peer review only

Available knowledge	#4	Summary of what is currently known about the problem, including relevant previous studies	3
Rationale	#5	Informal or formal frameworks, models, concepts, and / or theories used to explain the problem, any reasons or assumptions that were used to develop the intervention(s), and reasons why the intervention(s) was expected to work	3
Specific aims	#6	Purpose of the project and of this report	3/4
**Methods**			
Context	#7	Contextual elements considered important at the outset of introducing the intervention(s)	n/a - Appendix A
Intervention(s)	#08a	Description of the intervention(s) in sufficient detail that others could reproduce it	n/a - Appendix A
Intervention(s)	#08b	Specifics of the team involved in the work	n/a -Appendix A
Study of the Intervention(s)	#09a	Approach chosen for assessing the impact of the intervention(s)	4
Study of the Intervention(s)	#09b	Approach used to establish whether the observed outcomes were due to the intervention(s)	5
Measures	#10a	Measures chosen for studying processes and outcomes of the intervention(s), including rationale for choosing them, their operational definitions, and their validity and reliability	5
Measures	#10b	Description of the approach to the ongoing assessment of contextual elements that contributed to the success, failure, efficiency, and cost	5
Measures	#10c	Methods employed for assessing completeness and accuracy of data	5
Analysis	#11a	Qualitative and quantitative methods used to draw inferences from the data	5
Analysis	#11b	Methods for understanding variation within the data, including the effects of time as a variable	25
Ethical considerations	#12	Ethical aspects of implementing and studying the intervention(s) and how they were addressed, including, but	4

For peer review only

		not limited to, formal ethics review and potential conflict(s) of interest	
**Results**			
	#13a	Initial steps of the intervention(s) and their evolution over time (e.g., time-line diagram, flow chart, or table), including modifications made to the intervention during the project	n/a -Appendix A
	#13b	Details of the process measures and outcome	11-21
	#13c	Contextual elements that interacted with the intervention(s)	11-21 - described alongside each table and quotation
	#13d	Observed associations between outcomes, interventions, and relevant contextual elements	11-21 - described alongside each table and quotation
	#13e	Unintended consequences such as unexpected benefits, problems, failures, or costs associated with the intervention(s).	25
	#13f	Details about missing data	5, Figure 1, Appendix B
**Discussion**			
Summary	#14a	Key findings, including relevance to the rationale and specific aims	23
Summary	#14b	Particular strengths of the project	26
Interpretation	#15a	Nature of the association between the intervention(s) and the outcomes	23-25
Interpretation	#15b	Comparison of results with findings from other publications	23-25
Interpretation	#15c	Impact of the project on people and systems	23-25
Interpretation	#15d	Reasons for any differences between observed and anticipated outcomes, including the influence of context	23-25
Interpretation	#15e	Costs and strategic trade-offs, including opportunity costs	23-15

For peer review only

Limitations	#16a	Limits to the generalizability of the work	26
Limitations	#16b	Factors that might have limited internal validity such as confounding, bias, or imprecision in the design, methods, measurement, or analysis	26
Limitations	#16c	Efforts made to minimize and adjust for limitations	26
Conclusion	#17a	Usefulness of the work	26
Conclusion	#17b	Sustainability	26
Conclusion	#17c	Potential for spread to other contexts	26
Conclusion	#17d	Implications for practice and for further study in the field	26
Conclusion	#17e	Suggested next steps	26
**Other information**			
Funding	#18	Sources of funding that supported this work. Role, if any, of the funding organization in the design, implementation, interpretation, and reporting	27

Notes:

- 7: n/a - Appendix A
- 08a: n/a - Appendix A
- 08b: n/a -Appendix A
- 13a: n/a -Appendix A
- 13c: 11-21 - described alongside each table and quotation
- 13d: 11-21 - described alongside each table and quotation
- 13f: 5, Figure 1, Appendix B The SQUIRE 2.0 checklist is distributed under the terms of the Creative Commons Attribution License CC BY-NC 4.0. This checklist was completed on 25. April 2022 using https://www.goodreports.org/, a tool made by the EQUATOR Network in collaboration with Penelope.ai

For peer review only
